# Unlocking the Versatility of Linalool Dehydratase Reactivity: Tunable Stereochemical Control in Olefin Formation via Organic Synthesis

**DOI:** 10.1021/acscatal.5c02204

**Published:** 2025-09-09

**Authors:** Jianing Yang, Florian Walkling, Harald Gröger

**Affiliations:** Chair of Industrial Organic Chemistry and Biotechnology, Faculty of Chemistry, 9167 Bielefeld University, Universitätsstr. 25, Bielefeld 33615, Germany

**Keywords:** alkene synthesis, dehydration, enzymatic kinetic resolution, linalool dehydratase isomerase, whole-cell catalysis

## Abstract

Dehydration of α-allyl methyl alcohols catalyzed by linalool dehydratase isomerase (LinD) provides a straightforward route to alkene synthesis. While previous studies have shown that LinD exhibits high enantioselectivity, exclusively converting the *S*-enantiomer to β-myrcene as the sole alkene product, our findings reveal that this selectivity is restricted to the early stages of the preparative enzymatic synthesis. As the reaction proceeds, we observe the formation of the thermodynamically favored Saytzeff olefin, indicating that LinD’s catalytic profile is more versatile than previously recognized. Molecular dynamics (MD) simulations rationalize the observed enantioselectivity of the enzyme and further support the shift in selectivity during the reaction. A comprehensive synthetic approach demonstrates that this phenomenon enables selective access to both Saytzeff and Hofmann alkenes using the same enzyme. Overall, this study showcases the practical utility of LinD for selective alkene formation, including from non-natural substrates, and challenges the prevailing understanding of its catalytic properties and stereochemical selectivity. By integrating insights from biochemistry to large-scale synthesis for applications, these results reveal the potential for unexpected enzymatic behaviors and suggest that the current understanding of LinD should be expanded to incorporate additional perspectives.

## Graphical abstract

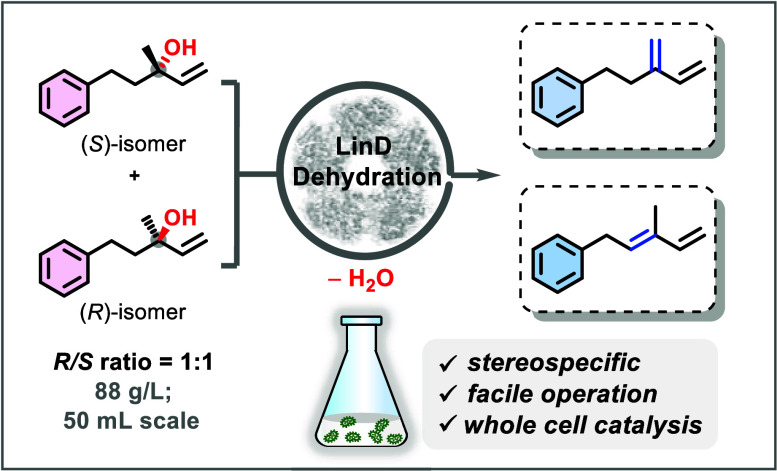

## Introduction

Olefins are crucial in the industry for polymer synthesis and serve as platform chemicals.^1–4^ Alcohol dehydration offers a direct route to valuable olefins, with various catalytic approaches available.^5–11^ However, dehydration of tertiary alcohols, which occurs at lower temperatures than that of primary and secondary alcohols, presents challenges due to competing side reactions and regioselectivity issues.^1,12,13^ Conventional acid–based chemocatalysis typically requires harsh reaction conditions, leading to limited selectivity and complex product mixtures that are challenging to separate.^1,14^ Consequently, biocatalysis has gained increasing attention as a more selective and sustainable approach to olefin production.^15–18^

Linalool dehydratase isomerase (LinD) from *Castellaniella defragrans* represents a promising and straightforward biocatalyst for dehydration chemistry.^19^ The bifunctional enzyme efficiently catalyzes *in vitro* the enantioselective dehydration of (*S*)-linalool to β-myrcene and its isomerization to geraniol.^19–21^ Its synthetic potential was recently demonstrated in a microbial conversion of glycerol to myrcene on a 20 mL scale, yielding 1.25 g/L of product.^22^ In this regard, the catalytic properties of LinD were extensively characterized by Hauer and coworkers.^19^ Among the key findings, LinD was shown to exhibit high enantioselectivity, exclusively converting the (*S*)-enantiomer to Hofmann olefin (**2–1**) as the sole alkene product (Figure 1A).^19^

**Figure 1:**
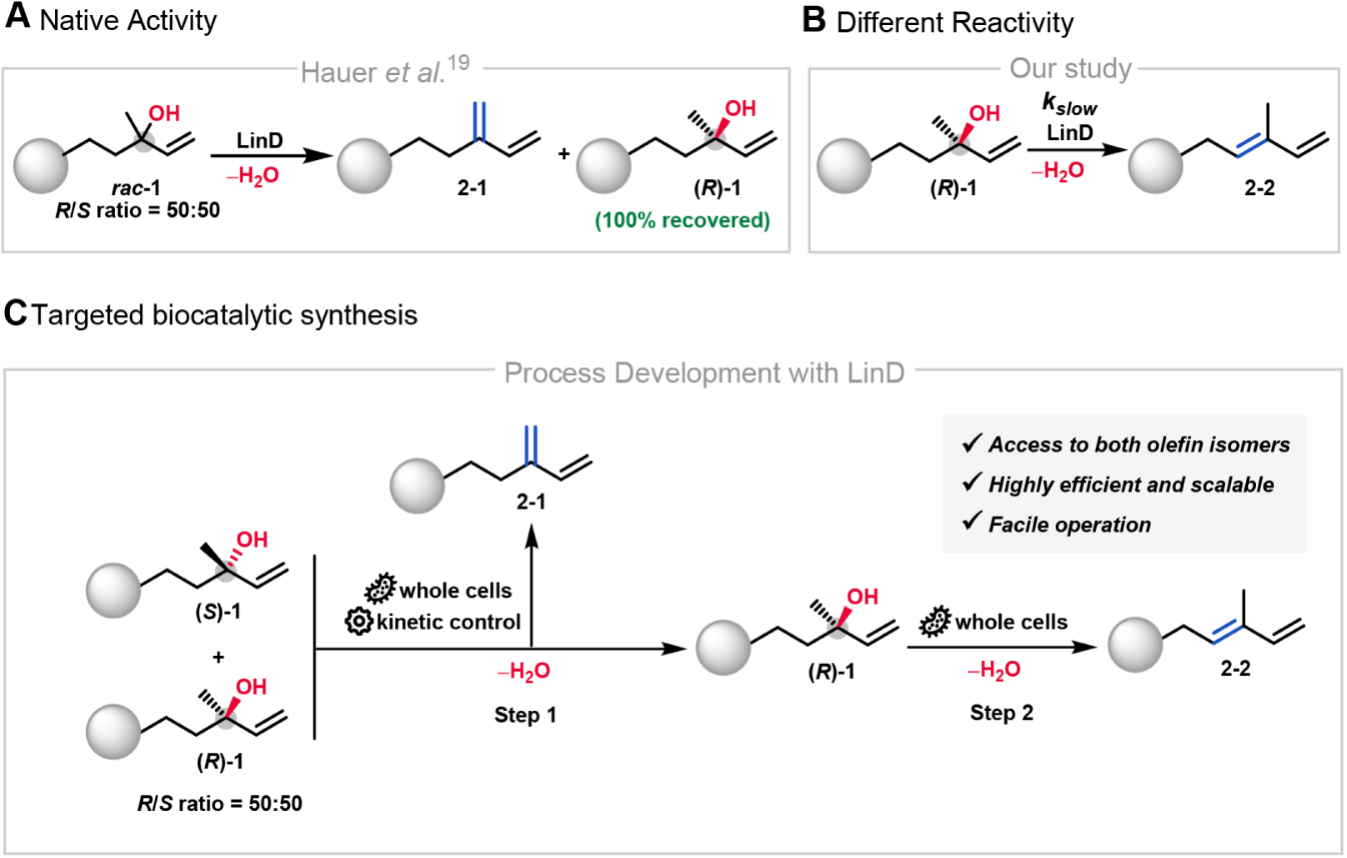
(A) LinD-catalyzed enantioselective water elimination as reported by Hauer et al.,^19^ yielding exclusively the Hofmann olefin (**2–1**). (B) Our observation: LinD exhibits promiscuous activity with (*R*)-alcohol, leading to additional Saytzeff product (**2–2**) formation, which complicates product isolation. (C) Application of LinD: a sequential dehydration process of *rac*-alcohols via LinD whole-cell catalysis enables stereoselective control, facilitating the tailored synthesis of Hofmann (**2–1**) and Saytzeff products (**2–2**), as well as (*R*)-alcohol (**(** *R* **)-1**).

When attempting to exploit the reported enantiopreference of LinD for the selective organic-synthetic resolution of various α-methyl allyl alcohols, we observed significantly broader catalytic activity than previously described, highlighting unexpected promiscuity with implications for practical biocatalysis. Olefin yields exceeding 50% suggest isomerization, noncatalytic alkene cleavage, or nonexclusive resolution of the alcohol, with the enzyme also exhibiting reactivity toward the *R*-enantiomer. Through detailed structural analysis and computational studies, we identified that LinD does not exclusively produce a single alkene but instead generates stereoselectively both Saytzeff and Hofmann products, with the product distribution depending on the absolute configuration of the substrate (Figure 1B). Within this context, we report that after process development this effect can be exploited at the preparative scale to selectively produce both alkenes using the same enzyme, as shown in Figure 1C. These results significantly broaden the current understanding of LinD’s catalytic properties and indicate that its originally proposed stereochemical pathway requires reconsideration.

## Results and Discussion

### Exploration of Stereoselective Olefin Formation via LinD-Catalyzed Dehydration

To explore LinD for process development in the enantioselective dehydration, aiming for exclusive formation of the β-myrcene analogue, we chose the non-natural α-methyl allyl alcohol *rac*-**1a** as the substrate and utilized LinD as whole cells for biocatalysis—a well-established strategy known for its efficiency and cost-effectiveness, as demonstrated by numerous technical examples across various enzyme classes (Figure 2). Our choice of this substrate was guided by a prior study by Hauer and coworkers,^19^ which demonstrated the enantioselective dehydration of the (*S*)-isomer of **1a** by isolated LinD on an analytical scale. This prior finding enabled a direct comparison with our whole-cell and preparative-scale experiments. In addition, the UV absorbance of the benzene moiety facilitated convenient detection and quantification. To ensure accurate enzyme dosing and achieve reproducibility as well as comparability across experiments, the whole-cell specific activity toward the natural substrate *rac*-linalool (*rac*-**1b**) was determined prior to conducting the reactions (see the Supporting Information for details).

**Figure 2:**
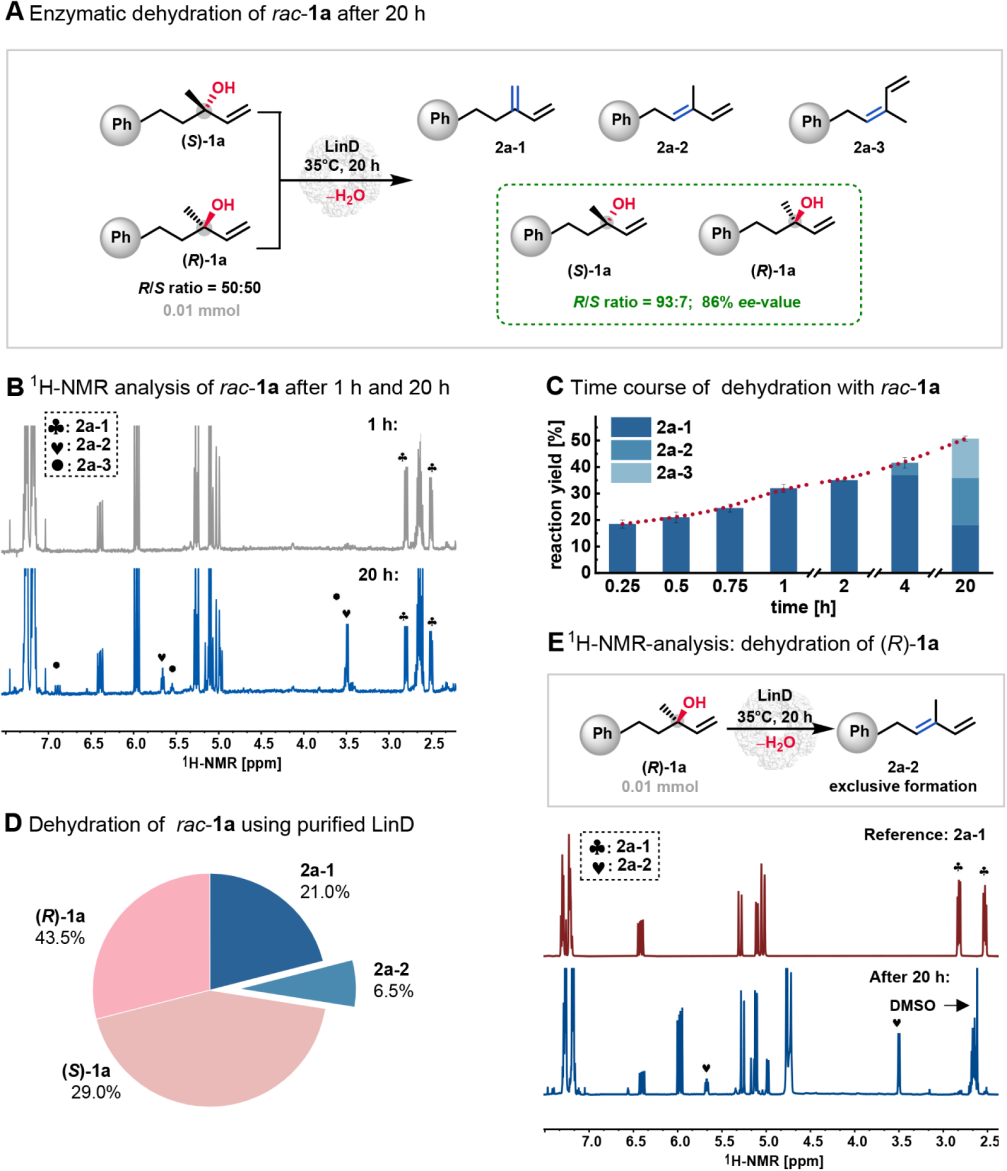
(A) Whole-cell catalysis of *rac*-**1a** formed three olefin isomers after 20 h. Enantioselectivity was assessed by chiral HPLC. Negative controls were performed without enzymes or using *E. coli* cells. (B) ^1^H NMR spectra (500 MHz, CDCl_3_): a comparison for dehydration of *rac*-**1a** (10 mM) after 1 and 20 h on a 1 mL scale. (C) Dehydration of *rac*-**1a** with varying reaction times using 0.2 mol % whole-cell LinD. (D) Dehydration of *rac*-**1a** (10 mM) using 0.3 mol % purified LinD for 10 h on a 1 mL scale. (E) LinD-catalyzed dehydration of (*R*)-**1a**. Reaction conditions: 10 mM (*R*)-**1a**, 0.3 mol % purified LinD, 1 mL scale, 20 h, 850 rpm. ^1^ H NMR spectroscopic analysis (500 MHz, CDCl_3_) showed a 28% reaction yield to **2a-2**. The spectrum of the Hofmann product (**2a-1**) was included for comparison.

Dehydration of 10 mM *rac* **-1a** was conducted on a 1 mL scale using LinD as a wet whole-cell catalyst and monitored over 20 h by chiral HPLC and^1^ H NMR spectroscopy (Figure 2A). Unexpectedly, we obtained not only the anticipated terminal alkene **2a-1** ^19^ but also the internal alkenes **2a-2** and **2a-3**: following the reaction over time revealed exclusive formation of the terminal alkene **2a-1** within the first 2 h. However, while **2a-2**, an internal alkene, was detected after 4 h, a decrease in the reaction yield of **2a-1** and the appearance of **2a-3** were observed after 20 h (Figure 2C). The ^1^H NMR spectra of the product mixtures after 1 and 20 h are shown in Figure 2B.

This result deviates significantly from the findings of Hauer and coworkers,^19^ which demonstrated sole conversion of (*S*)-**1a** in a racemic mixture of **1a** to the Hofmann product **2a-1** with excellent selectivity using isolated LinD. To rule out the possibility that the internal alkenes originated from components of the whole-cell catalyst, the reaction was repeated using purified LinD. After 10 h, a mixture of (*R*)-**1a**, (*S*)-**1a**, **2a-1**, and **2a-2** was obtained in a ratio of 43.5:29:21:6.5, as shown in Figure 2D. This outcome is comparable to those obtained with whole-cell catalysis, thereby excluding the correlation between the formation of minor products and the use of the whole-cell catalyst instead of isolated LinD (Figure 2D).

Given that the reducing agent may influence both the reactivity and selectivity of LinD, we systematically varied the type (e.g., DTT, TCEP) and concentration (2–50 mM) of reducing agents used. Testing up to 50 mM DTT revealed no significant impact on reaction yields or selectivity, confirming that 2 mM is sufficient. Alternative reducing agents, such as TCEP, provided no additional benefit, supporting our choice of DTT for this study.

Since the formation of the Saytzeff product has not been described for LinD, its appearance in the biocatalytic reaction was surprising and prompted several key questions about the reaction mechanism. Two plausible pathways were proposed for the formation of the Saytzeff product **2a-2**—the second product observed in LinD-catalyzed dehydration: (1) direct enzymatic dehydration of (*R*)-**1a** or (2) postisomerization of the Hofmann product **2a-1** because the content of (*R*)-**1a** and the Hofmann product **2a-1** decreased after 4 h. To investigate both possibilities, pure (*R*)-enantiomer (*R*)-**1a** and Hofmann product **2a-1** were separately treated with LinD.

Reactions of 10 mM **(** *R* **)-1a** using whole-cell LinD (1 mL scale) exclusively yielded the Saytzeff product **2a-2**, regardless of the catalyst loading from 0.2 to 0.8 mol % (for details, see Figures 2E and S2). The same reaction using purified LinD was performed in triplicate and yielded **2a-2** in 28%–30% in 20 h; the reaction rate was significantly slower than the formation of the Hofmann product **2a-1** from (*S*)-**1a**. These results confirm the hypothesis that the dehydration of (*R*)-**1a** by LinD yields the Saytzeff product **2a-2**.

Biotransformation of Hofmann product **2a-1** using whole-cell LinD on a 1 mL scale resulted in significant isomerization, yielding internal alkene **2a-3** (Figure 3B). Additionally, a hydration reaction forming (*S*)-**1a** as an alcohol was detected. Consequently, these results explain the origin of the internal alkene **2a-3** observed in previous experiments with racemic alcohol *rac*-**1a**: the Hofmann product **2a-1** undergoes isomerization to form **2a-3**. Negative control reactions using *E. coli* cells without the LinD insert confirmed the absence of detectable enzyme activity.

**Figure 3:**
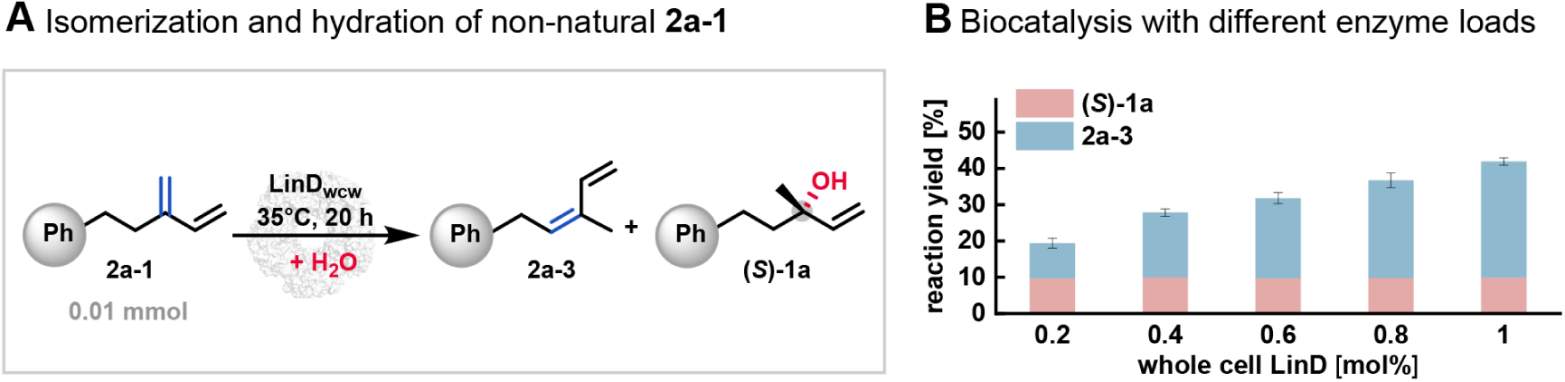
(A) Schematic illustration of the isomerization and hydration of **2a-1** catalyzed by LinD. (B) Results of whole-cell biocatalysis of **2a-1** with varying enzyme loads over 20 h. Reaction conditions: **2a-1** (10 mM), whole-cell LinD, 1 mL scale, 20 h, 850 rpm.

Based on these findings, the dehydration of the natural substrate *rac*-linalool *rac*-**1b** was further investigated using LinD. We also observed the formation of Hofmann and Saytzeff products by using the native substrate racemic linalool *rac*-**1b** in the biotransformation using whole-cells with LinD, consistent with our previous findings for the non-natural substrate *rac*-**1a** but contrary to previous reports.^19^ Notably, in reactions with *rac*-**1b** using whole-cells with LinD, the Saytzeff product (**2b-2**) was formed in yields below 50%, indicating significant biocatalytic activity toward this olefin type, as shown in Figure 4A. Furthermore, the formation of the previously unreported Saytzeff product **2b-2** was found to be strongly dependent on catalyst loading (Figure 4A). Increasing the catalyst-to-substrate ratio from 0.01 to 0.6 mol % led to a rise in the Saytzeff product **2b-2** yield from 0% to 14%.

**Figure 4:**
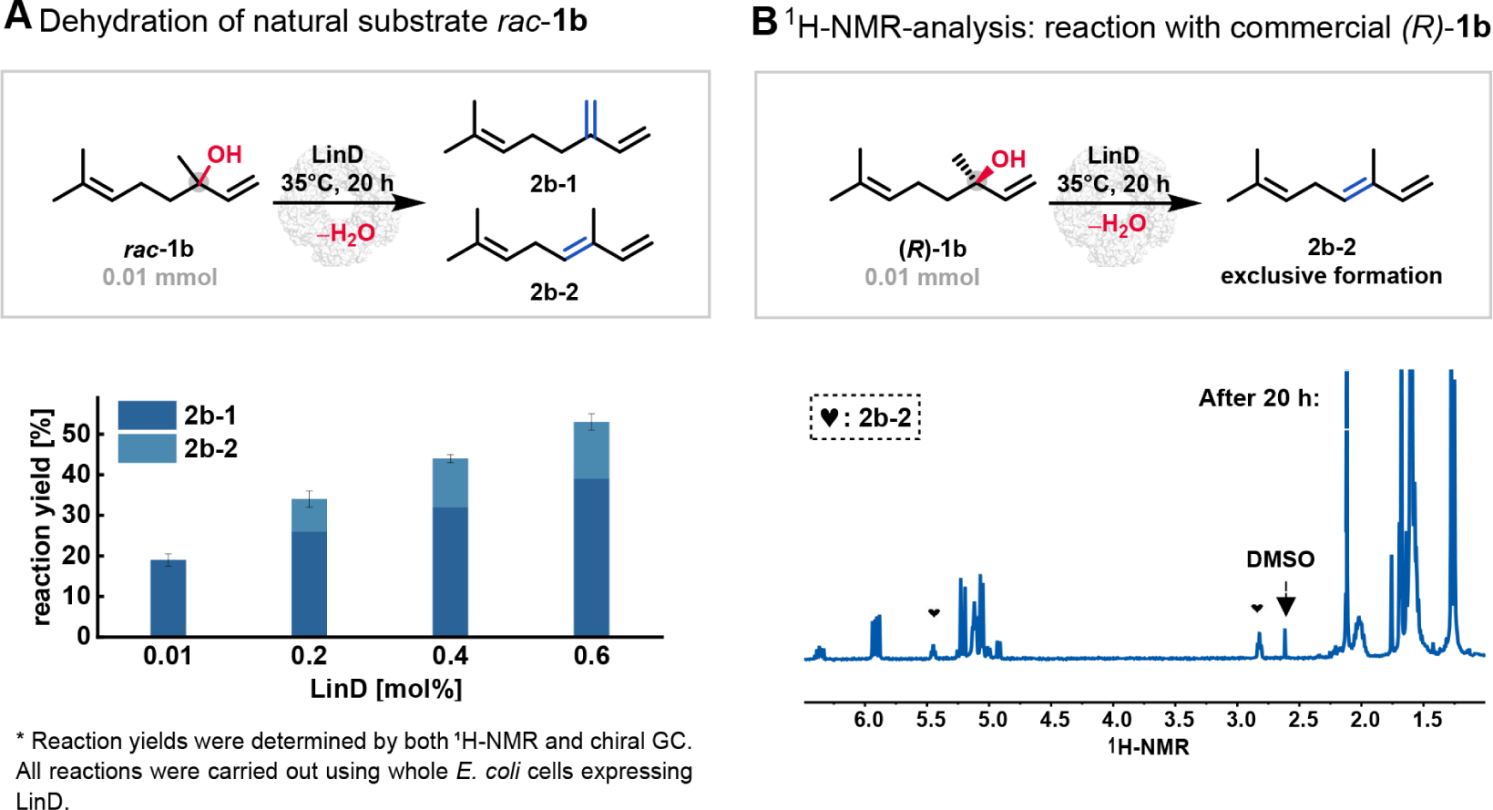
(A) Whole-cell catalysis of *rac*-**1b** (10 mM) resulted in two olefin isomers after 20 h on a 1 mL scale. Reaction yields are averages of three replicates; details are in the Supporting Information. (B) Dehydration of (*R*)-linalool ((*R*)-**1b**) using purified LinD on a 1 mL scale. A cutout of ^1^H NMR spectra (500 MHz, CDCl_3_) of dehydration of *(R*)-**1b** (10 mM, 95% purity) after 20 h using 0.3 mol % purified LinD. The formation of **2b-2** with a reaction yield of 23% could be identified.

To rule out that the formation of the unexpected Saytzeff olefin is due to the use of a whole-cell catalyst and a resulting “background reaction”, we conducted biocatalytic dehydration using purified LinD. ^1^H NMR spectroscopic and GC analysis yielded similar results, confirming the presence of both Hofmann and Saytzeff olefins (for detailed structural data, see the Supporting Information).

Consistent with our previous findings for (*R*)-**1a**, comparable results for the formation of the Saytzeff product **2b-2** were observed when using the natural substrate (*R*)-**1b** with LinD, in both whole-cell and purified forms (Figures 4B and S3 and S4). No racemization of (*R*)-**1b** could be detected in all cases. Even more remarkable is the discovery that the nature of the olefin product—whether Saytzeff or Hofmann—is dictated by the absolute configuration of the substrate’s enantiomer and can be selectively controlled using the same enzyme. This phenomenon, in which the absolute configuration of the substrate determines the regioselectivity (as here) or diastereoselectivity of the product formation while employing the same biocatalyst, is exceptionally rare in enzymatic transformations; related examples have been reported earlier for nitrile formation^23^ and lactone synthesis.^24,25^

Accordingly, our results indicate that independent of the substrate structure, LinD-type enzymes have in general the capability to form Hofmann- and Saytzeff-products in the dehydration of both non-native and natural α-methyl allyl alcohol substrates of type *rac*-**1**, which opens up a valuable synthetic perspective not only for kinetic resolution of such tertiary alcohols but also to get preparative access to both types of olefins, Hofmann- and Saytzeff-olefin products, even when utilizing the same enzyme.

### Computational Analysis of Stereoselective Olefin Formation Driven by Substrate Enantiomer Configuration

To shed light on the molecular basis for the observed control of the formed olefin stereoisomer by the absolute configuration of the substrate when using the same LinD enzyme, we performed computational modeling using (*S*)-**1a** and (*R*)-**1a** as substrates. The geometry of both isomers was optimized by means of density functional theory (DFT) at the PBE0 D3BJ def2-TZVP def2/J level of theory.^26,27^ *In silico* analysis using structure-based molecular docking and molecular dynamics (MD) simulations revealed an appropriate binding mode for both enantiomers in the active site of LinD (PDB: 5H1R), which can enable the dehydration step. The MD simulations illustrate significant differences in the binding behavior of (*S*)-**1a** and (*R*)-**1a** enantiomers with the LinD protein, as depicted by their respective free energy landscapes (FELs) and binding interactions within the active site (Figure 5). In our modeling, the key active-site residues H128, Y44, D38, C179, and C170, which interact with the substrates, are consistent with previous literature findings.^20,28^ However, the structural comparison highlight the different interactions between each enantiomer and key active-site residues.

**Figure 5:**
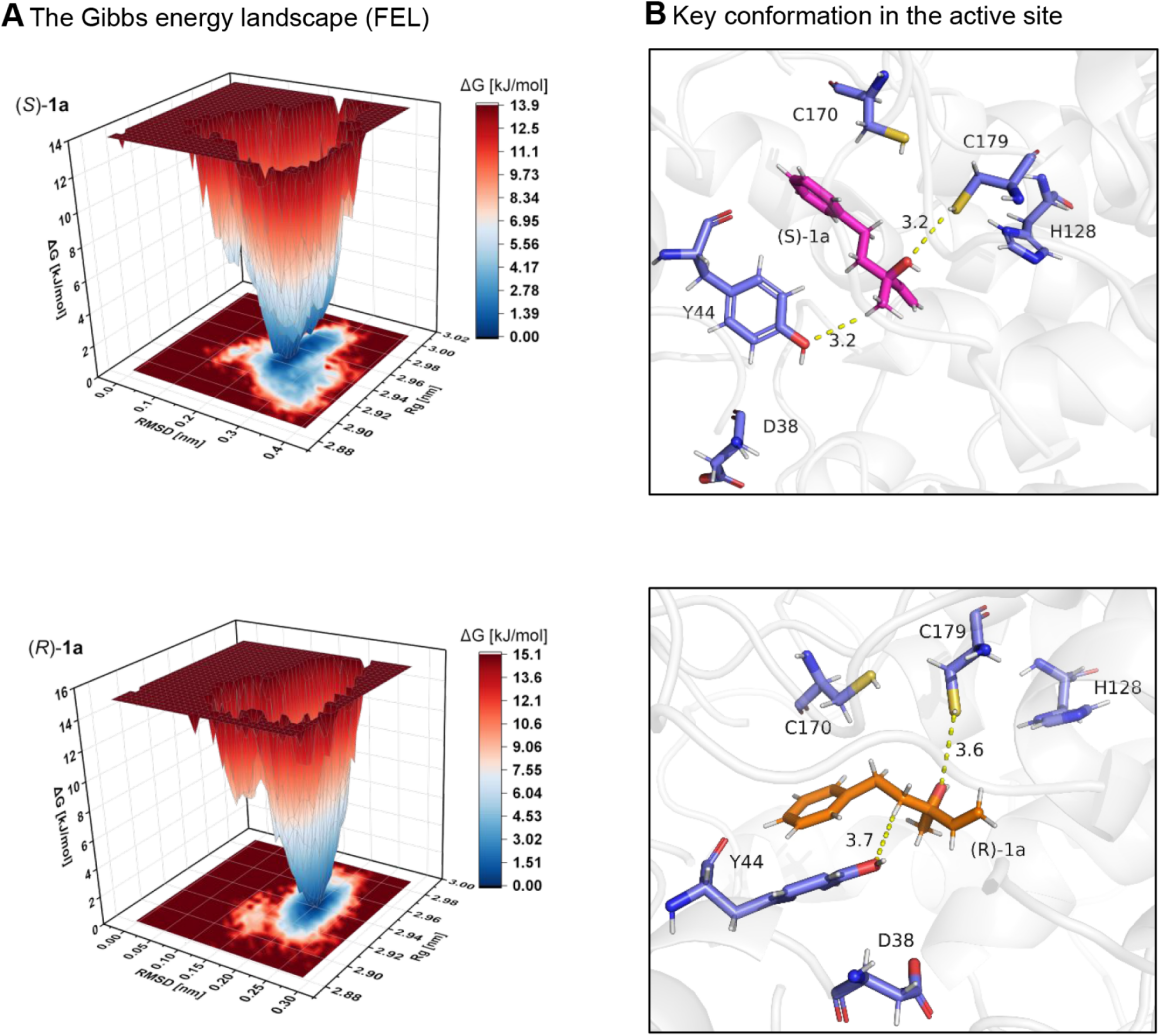
(A) Gibbs energy landscapes were obtained during 100 ns MD simulations for (*S*)-**1a** and (*R*)-**1a** using the crystal structure from LinD as a dimer (PDB: 5H1R). (B) Binding mode analysis of (*S*)-**1a** (magenta) and (*R*)-**1a** (orange) in the active site of LinD. Essential amino acid residues^20^ are shown in the blue stick format.

To investigate the stereochemical basis of LinD selectivity, free energy landscape (FEL) analyses were performed for (*S*)- and (*R*)-**1a** based on RMSD and the radius of gyration (Rg) within the active site. The FEL of the *S*-isomer reveals a well-defined, deep free energy minimum centered on it, suggesting a stable and conformationally favored binding mode within the enzyme pocket. In contrast, the *R*-isomer exhibits a narrower and shallower energy well, with increased energetic dispersion and a higher global minimum (Δ*G* = 15.1 kJ/mol vs 13.9 kJ/mol for the *S*-isomer). These findings indicate stronger conformational stabilization of the *S*-isomer in the active site, consistent with the experimentally observed preference for Hofmann-type olefin formation from the *S*-configured intermediate. As shown in Figure 5A, the complex of (*S*)-**1a** in LinD displays a key conformation at the energy minima, in which the hydroxyl group of (*S*)-**1a** and one of the methyl protons are positioned antiparallel to each other. Simultaneously, the hydroxy group interacts with the thiol group of C179, while the methyl proton engages in close contact with the phenol group of Y44, both with effective distances of 3.2 Å.

This conformation facilitates a favorable β-elimination, leading to the Hofmann product **2a-1.** In contrast, the complex of (*R*)-**1a** in LinD displays a conformation at the energy minimum in which the benzyl group is antiparallel to the methyl group, while the hydroxy group is antiparallel to one of the methylene protons. Within this geometry, the hydroxy group interacts with the thiol of C179, and the methylene proton engages with the phenol group of Y44, with effective distances of 3.6 and 3.7 Å, respectively. This conformation promotes β-elimination to (*E*)-Saytzeff product **2a-2.** The shorter interaction distances observed in the complex of (*S*)-**1a** with LinD suggest stronger activation compared to the key conformation of (*R*)-**1a** in LinD. This difference in activation likely accounts for the distinct dehydration rates observed for (*S*)-**1a** and (*R*)-**1a** obtained using LinD as the biocatalyst.

### Exploring the Synthetic Space of LinD: Advancements in Whole-Cell Catalysis

To showcase the utility of LinD as a whole-cell catalyst in organic synthesis, we explored its application scope for synthesizing a broad range of Hofmann and Saytzeff olefins, both of which are highly valuable in organic chemistry.^29,30^ These olefins serve as valuable substrates, e.g., for Diels–Alder reactions, thus facilitating efficient formation of six-membered carbocycles.^29^ The resulting cyclohexenes are prevalent structural motifs found in numerous natural products and pharmaceuticals.^29,31^ In order to investigate the substrate scope of LinD-catalyzed Hofmann and Saytzeff olefin formation, we synthesized a range of racemic alcohols of type *rac*-**1x** via Grignard reactions, which were then subjected to biocatalytic dehydration using LinD, as shown in Figure 6A. LinD was overexpressed in *E. coli*, and reproducible activity when used as a whole-cell catalyst was confirmed for the natural substrate *rac*-**1b** (see the Supporting Information). The desired olefin isomers were identified by ^1^H NMR spectroscopy, and the enantiomeric excess of the remaining alcohol mixture was determined via chiral HPLC and chiral GC. Additionally, no background reaction was detected when using *E. coli* cells lacking recombinant LinD, thus confirming that these transformations are catalyzed by LinD.

**Figure 6:**
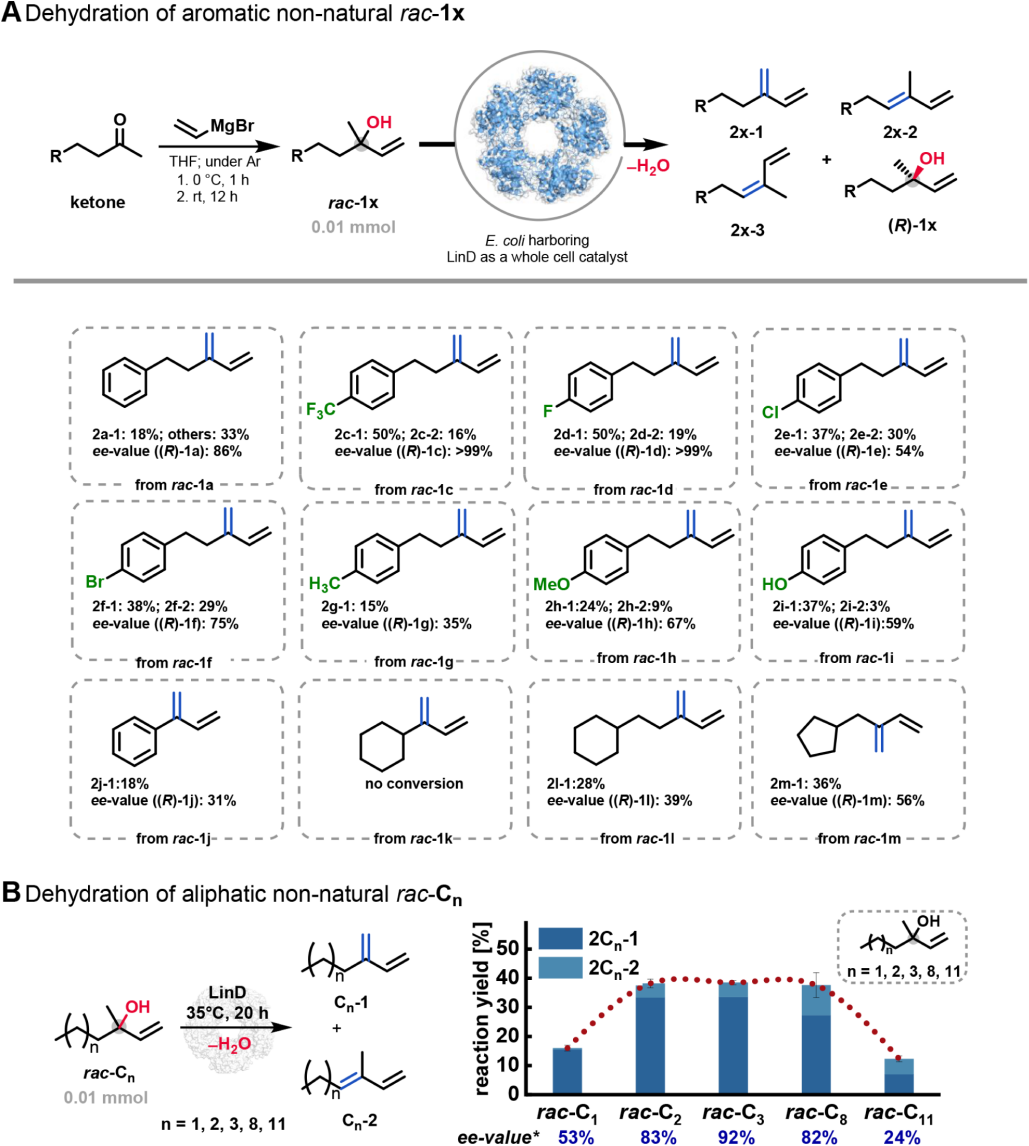
(A) Substrate scope study of biocatalytic dehydration with LinD for aromatic α-methyl allyl alcohols and cycloalkyl-substituted analogs. (B) Dehydration of aliphatic olefins using LinD as a whole-cell catalyst. Reaction conditions: *E. coli* whole cells (0.2 mol %, 60 mg wcw mL^–1^ ≙ 2.77 mU/mg_wcw_), 10 mM *rac*-alcohol, 2 mM DTT, and 5% (v/v) DMSO in citrate-buffer (50 mM, pH = 6), 20 h, 35 °C, and 850 rpm on a 1 mL-scale. All reactions were performed in triplicates. Negative control reactions were conducted using *E. coli* cells without the LinD insert, as well as reactions without any biocatalyst. The *ee*-value of (*R*)-isomers’ analysis of the residual alcohol was determined via chiral GC analysis.

To our delight, the substrate screening revealed that nearly all tested α-methyl allylic alcohols of general type *rac*-**1x** were effectively accepted and converted by LinD, as shown in Figure 6A. When exploring halogen-substituted aromatic alcohols (with F-, Cl-, and Br-substituents) for dehydration, F-substituted alcohols showed excellent activity and selectivity, allowing the isolation of (*R*)-isomers ((*R*)-**1c** and (*R*)-**1d**). For example, in the case of substrate *rac*-**1d**, (*S*)-**1d** was fully converted to Hofmann olefin **2d-1**, while a minor amount of (*R*)-**1d** was converted to **2d-2**. Chiral-HPLC analysis of the remaining (*R*)-**1d** revealed an excellent *ee* value of >99%. This approach not only provided efficient access to the nonconverted tertiary alcohol (*R*)-**1d** but also led to an overall yield of 69% for all synthesized olefin isomers, including both Hofmann and *E*-Saytzeff olefins.

Notably, the resulting fluorine-containing Hofmann olefins offer promising potential for synthetic applications in the life sciences due to the beneficial effect of fluorine.^32^ Additionally, the unique reactivity and versatility of chlorine- or bromine-substituted olefins makes them suitable for further utilization in halogen-metal exchange or cross-coupling reactions.^33^ However, although the yields of these chloro- and bromo-containing substrates *rac*-**1e** and *rac*-**1f** exceeded 60%, the enantioselectivity of the reaction was comparatively low with a maximum *ee*-value of 75%. LinD also exhibited activity in the presence of electron-donating substituents at the aromatic ring, such as CH_3_ or OCH_3_ groups, or when the polarity was enhanced by introducing a hydroxy group at the *para*-position of the aromatic ring (Figure 6A).

Based on these findings, we further investigated the dehydration of the alcohol substrates *rac*-**1k** and *rac*-**1j**, which have similar structural characteristics but exhibit reduced flexibility. However, in contrast to their more flexible counterparts *rac*-**1a** and *rac*-**1l** bearing also a phenyl and cyclohexyl moiety, respectively, the sterically more rigid analogous alcohols *rac*-**1j** and *rac*-**1k** showed decreased or even no conversion after 20 h.

Our observations prompt several hypotheses: first, at least some conformational flexibility, as observed for *rac*-**1a** and *rac*-**1l**, appears to be advantageous for achieving high activity; second, the greater flexibility of substrate *rac*-**1j**, combined with its planar aromatic ring, likely promotes a tighter fit within the active site, potentially enhancing its interaction with LinD. In contrast, the bulkier, chair-like configuration of the cyclohexyl moiety makes this substrate *rac*-**1k** sterically more demanding and difficult to fit well into the active site, resulting in reduced activity. Furthermore, we discovered that LinD could convert not only aromatic α-methyl allylic alcohols but also analogous cycloalkane-substituted tertiary alcohols, such as *rac*-**1l** and *rac*-**1m,** into the corresponding alkene products. A similar trend is observed when comparing substrates *rac*-**1l** and *rac*-**1m**, further supporting the idea that substrate flexibility and steric rigidity play a crucial role in the biocatalytic dehydration process, as shown in Figure 6A.

Having demonstrated the suitability of LinD to convert a broad range of aryl- or cycloalkanol-substituted alcohols into the desired olefins, we next turned our attention to the synthesis of aliphatic olefins using LinD for dehydration. To systematically assess the influence of chain length on enzymatic activity and selectivity, we synthesized a series of aliphatic α-methyl allyl alcohols (*rac*-C_n_) with varying chain lengths. Interestingly, the *S*-enantiomer of these aliphatic alcohols *rac*-**C**_**n**_ was consistently converted into Hofmann **C**_**n**_ **-1** and *E*-Saytzeff-olefins **C**_**n**_ **-2** in all cases within 20 h (Figure 6B). Starting from the C_6_ substrate, the reaction yields raised up with increased chain lengths up to C_13_. This demonstrates that the active site is sufficiently accommodating even more sterically demanding substrates. Since the theoretical yield for *S*-selective dehydration is limited to 50%, the achieved 38% reaction yield for the C_13_ alcohol represents a promising starting point for future process optimization. However, the olefin yields decreased significantly when a C_16_ chain length alcohol was employed, and for the aliphatic substrates with a carbon chain length of ≥C_11_, enantioselectivity was generally very low (Figure 6B). Additional substrate moieties that were not accepted by LinD are summarized in Figure S8, which indicates the narrow structural tolerance of LinD.

### Bioprocess Development: LinD-Catalyzed Dehydration at a High Substrate Loading (88 g/L)

While classical chemocatalytic alternatives, such as sulfuric acid, are commonly employed for the dehydration of tertiary alcohols, the question arises as to what advantages enzyme catalysis offers over traditional chemocatalysts. To address this, we compared the performance of the enzyme LinD to that of sulfuric acid, a classic Brønsted acid, under comparable reaction conditions (e.g., room temperature). The results highlight the superior performance of enzyme catalysis, particularly in terms of selective olefin formation. As shown in Figure 7A, in the presence of sulfuric acid no reaction occurred at 35 °C, and the reaction yield remained low even at 65 °C. At the elevated temperature of 80 °C, a complex mixture of Hofmann and Saytzeff olefin products was formed, likely due to the thermodynamically controlled nature of the reaction at this temperature. This underscores the challenge in achieving selective olefin formation with classical chemocatalysts.

In contrast, LinD whole-cell catalysis operates under milder conditions, offering more precise control over the reaction outcome. Building on the structural insights obtained from our findings, we next focused on the development of a practical preparative process to access both the enantiomerically pure *R*-enantiomer of the substrate *rac*-**1a** and the Hofmann product **2a-1** (Figures 7B and S5 and S7). Accordingly, we carried out the biocatalytic dehydration of *rac*-**1a** in an organic-synthetic biotransformation on a 5 mL scale. By adjusting the amount of wet biomass and appropriate chromatographic separations, we finally successfully optimized reaction conditions to prevent Saytzeff **2a-2** formation and achieved a 47% reaction yield for the Hofmann product **2a-1**.

**Figure 7:**
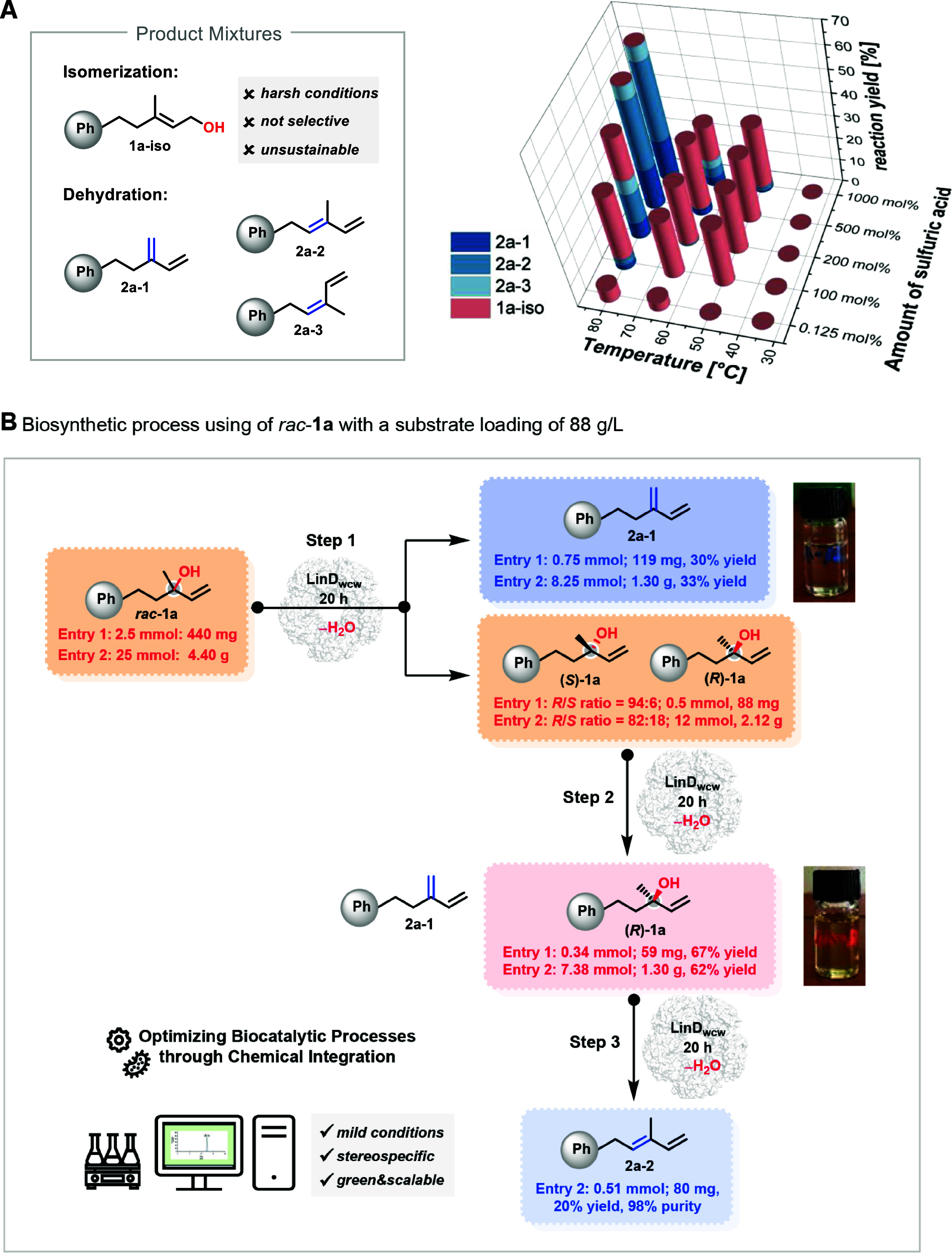
(A) Limitations of the chemocatalytic dehydration of *rac*-**1a** in water using H_2_SO_4_ as a catalyst. Various reaction conditions were screened, and results were analyzed by ^1^H NMR spectroscopy in CDCl_3_ after 20 h. (B) An overview of the biosynthetic concept for the isolation of pure Hofmann-product **2a-1** and (*R*)-**1a** with >99% ee using kinetic racemate resolution as a key methodology on a preparative scale.

When taking into account the theoretical yield limitation of a kinetic resolution to 50%, the reaction yield achieved at this stage is at a good to excellent level. Additionally, since the reaction is performed in aqueous media and both the product and substrate are insoluble in water, removal of DMSO as a cosolvent can be achieved by simple washing steps after workup with an organic solvent. In the second step, the remaining alcohol (*R*/*S* = 94:6) was further dehydrated under biocatalytic conditions using an optimized enzyme amount, resulting in the residual isomer (*R*)-**1a** with 94% reaction yield (Figure 7B). This strategy was further successfully applied for the gram-scale synthesis of (*R*)-**1a** and **2a-1,** as shown in Figure 7B (see the Supporting Information for details).

Both components were isolated via column chromatography and characterized using ^1^H NMR spectroscopy and chiral-HPLC (see the Supporting Information for details). This comparison clearly demonstrates that biocatalysis with LinD not only offers superior selectivity but also provides a robust and efficient alternative to classical chemocatalytic methods. It is further noteworthy that, in contrast to the low substrate concentrations (typically 10 mM or even less) of LinD processes reported in the literature,^22^ we were able to run our biotransformation under optimized conditions at 500 mM substrate concentration (corresponding to a substrate loading of 88 g/L; see the Supporting Information for details), thus already fulfilling the criteria for a technically feasible process.^34–36^

While LinD is primarily studied here for its catalytic efficiency in olefin formation, this enzyme can also be used for enantioselective resolution of tertiary alcohols. Note that for such resolution of tertiary alcohols, only very few alternatives exist and even the scope of lipases for such substrates is very narrow (with lipase CAL-A as one of the rare catalysts for such a transformation).^37,38^ Furthermore, our studies demonstrated that LinD-containing whole-cell catalysts can be effectively utilized in both immobilized and lyophilized forms (Figure S6). Overall, we envision that this approach can be further developed into a versatile strategy for the direct synthesis of olefins as well as enantiomerically pure tertiary alcohols such as, e.g., aromatic α-methyl allyl alcohols, significantly streamlining synthetic efforts for such molecules and expanding the scope of biocatalytic transformations.

## Conclusions

In summary, we present a refined understanding of the catalytic properties of linalool dehydratase isomerase (LinD), an enzyme of growing interest in the stereoselective transformation of tertiary alcohols and alkene synthesis. Our findings reveal that LinD exhibits broader reactivity than previously assumed, challenging the notion of strict stereoselectivity and exclusive Hofmann selectivity. A biosynthetic approach establishes LinD as a versatile biocatalyst for alkene synthesis, where the ratio of formed olefins is dynamically modulated by the reaction time and enzyme concentration. This dual reactivity enables controlled access to Hofmann and Saytzeff olefins, expanding the enzymatic repertoire for stereoselective alkene formation. Overall, our studies demonstrate that the enzymatic dehydration catalyzed by LinD proceeds stereoselectively and depends on the absolute configuration of the alcohol substrates: (*S*)-Alcohols are selectively converted into Hofmann olefins, while (*R*)-alcohols gradually yield Saytzeff olefin products over time. This behavior enables the controlled formation of complementary alkenes using a single enzyme. Moreover, LinD tolerates high substrate concentration (up to 500 mM) and accepts a broad range of non-natural aromatic alcohols as substrates, thus reinforcing its potential for preparative applications. Beyond its intrinsic reactivity, LinD as a whole-cell catalyst offers operational advantages over traditional chemocatalysts, providing enhanced selectivity under mild conditions. This expanded catalytic profile not only complements existing enzymatic strategies but also underscores LinD’s potential as a versatile biocatalyst for tailored olefin formation. We believe that these insights pave the way for further mechanistic and engineering studies to harness LinD for efficient selective synthesis of olefins and enantiomerically pure tertiary alcohols under mild conditions.

## Methods

### Materials

All chemicals and solvents, unless otherwise described, were purchased from commercial suppliers (TCI, abcr, Sigma-Aldrich, BLDpharm, Thermo Scientific, and Enamine) and applied without further purification. Isopropyl-β-D-thiogalactopyranoside (IPTG), kanamycin, and LB media were purchased from Carl Roth. The *E. coli* cells were sourced from Twist Bioscience. The pET28a­(+) vector containing a C-terminal His_6_-tag was used for LinD.

#### Enzyme Preparation

LinD was constructed with the pET28a­(+) vector and transformed into *E. coli* BL21­(DE3), followed by the preparation of glycerol stocks stored at −80 °C. For inoculation, 5 mL LB cultures containing kanamycin as an antibiotic (50 μg/mL) were prepared from single colonies obtained from agar plates or less amount of glycerol stocks and then incubated overnight at 37 °C and 180 rpm. Protein expression was carried out in 2 L culture flasks containing 400 mL of LB media and kanamycin (50 μg/mL). Main cultures were inoculated with the overnight precultures to a starting concentration of 1% (v/v) and allowed to grow to an OD_600_ value between 0.6 and 0.8 at 37 °C and 150 rpm. Afterward, induction was initiated by adding IPTG to a final concentration of 0.05 mM, followed by further incubation for 5 h at 37 °C. Finally, cells were harvested by centrifugation (30 min, 4000 × g, 4 °C) and stored at −20 °C for subsequent applications.

#### Protein Purification

Frozen cell pellets were thawed on ice and resuspended in citrate buffer (50 mM, pH = 6) at a concentration of 300 mg/mL. These cell suspensions were sonicated (Bandelin Sonoplus UW2070) three times each on ice for 3 min each, with 5 cycles at 20% power. Cell debris was removed by centrifugation (12,000 rpm, 4 °C for 15 min), and the soluble crude extract fraction was filtered through 0.2 μM filters before being purified using a HisTrap HP 5 mL column loaded with Ni^2+^. Elution of the crude extract fractions was carried out with an imidazole gradient, starting with 20 mM (4 cv), followed by 40 mM (4 cv), 70 mM (4 cv), 100 mM (4 cv), and 300 mM (5 cv) imidazole concentrations. The enzyme-containing fractions were identified by BCA color formation, combined, and desalted with PD-10 desalting columns packed with Sephadex G-25 resin. The desalted protein solution was concentrated using a centrifugal filter tube (10 kDa). Purity was assessed by 12% SDS-PAGE analysis, and the concentration was determined using a NanoDrop spectrophotometer. Aliquots of purified proteins were stored at −80 °C until further use.

#### Screening of Biocatalytic Dehydration with LinD

All reactions were conducted in 2 mL micro-reaction vessels and performed in triplicate. In general, tertiary alcohol *rac*-1 (10 mM) was typically dissolved in DMSO (5%, *v*/*v*), 2 mM DTT, and LinD as a whole-cell catalyst (wet cell weight, 60 mg ≙ 166 mU ≙ 0.2 mol %, determined by activity tests using *rac*-linalool) was added. The reaction mixture was then incubated for 20 h at 35 °C and 850 rpm in a thermoshaker. Afterward, the sample was extracted with organic solvents (800 μL cyclohexane or CDCl_3_), and the resulting organic fraction was washed with dd. H_2_O (3 × 800 μL). Product formation was analyzed by ^1^H NMR spectroscopy, while enantiomeric excess was determined by chiral HPLC or GC.

#### Kinetic Resolution of *rac*-**1a** with LinD (5 mL Scale)

Frozen cells were dissolved in citrate-buffer (50 mM, pH = 6) to a concentration of 300 mg/mL, and their activity was tested using *rac*-**1b** (10 mM) as the natural substrate. 0.004 mol % of LinD was utilized as a whole-cell catalyst for the biocatalytic dehydration on a 5 mL scale. The reaction was carried out using 500 mM *rac*-**1a** in DMSO (5%, v/v) and DTT (2 mM) for 20 h at 35 °C with 850 rpm. Subsequently, the sample was worked up with ethyl acetate (3 x 5 mL), and the product formation was verified by ^1^H NMR spectroscopy. The enantiomeric excess was determined by HPLC (Chiralpak IC (4.6 mm ID × 250 mm)) with a mobile phase of *n*-hexane/2-propanol (98:2) at a flow rate of 1.0 mL/min and a detection wavelength of λ = 250 nm. Isolation of Hofmann-olefin was carried out using automated column chromatography (Büchi, column: FP ECOFLEX Si 25 g, cyclohexane: DCM = 9:1). For the isolation of *R*-alcohol (*R*)-**1a**, we used 0.004 mol % LinD under the same reaction condition for the dehydration of residual alcohol with an *R*/*S* isomer ratio of 94:6, followed by isolation of *R*-alcohols using column chromatography and analysis via chiral HPLC and ^1^H NMR spectroscopy.

#### Molecular Modeling

Geometry optimizations of (*R*)-**1a** and (*S*)-**1a** were performed using DFT calculations with the ORCA 5.0.3 software package.^39–41^ All minima were characterized at the PBE0 D3BJ def2-TZVP def2/J level of theory^26,27^ in the gas phase. Docking experiments with substrates (*R*)-**1a** and (*S*)-**1a** in the active site of LinD (PDB ID: 5H1R) were conducted using AutoDock Vina 1.2.0.^42,43^ Molecular dynamics (MD) simulations and analyses were carried out with the GROMACS 2023 ^44^ simulation package. Visualization was performed using Avogadro^45^ and Open-Source PyMOL.^46^

## Supplementary Material



## Notes

Experimental procedures, characterization data, ^1^H and ^1^C NMR spectra for all synthesized compounds, HPLC and GC chromatograms, the MD simulation setup, and additional figures referenced in the main text (PDF)
