# Supplementary material for: Unlocking the Versatility of Linalool Dehydratase Reactivity: Tunable Stereochemical Control in Olefin Formation via Organic Synthesis
Source: ACS Catal. 2025 Sep 9;15(18):16369–79. doi: 10.1021/acscatal.5c02204 (PMC12455561; doi:10.1021/acscatal.5c02204)
Supplement: Supplementary file 1 [file cs5c02204_si_001.pdf]

Supporting Information

Unlocking the Versatility of Linalool Dehydratase Reactivity: Tunable Stereochemical Control in Olefin Formation via Organic Synthesis

Jianing Yang,[a] *Florian Walkling,*[a] *Harald Gröger**[a]

[a] Chair of Industrial Organic Chemistry and Biotechnology, Faculty of Chemistry,

Bielefeld University, Universitätsstr. 25, 33615 Bielefeld, Germany

*E-mail: harald.groeger@uni-bielefeld.de

TABLE OF CONTENTS

1. Material and methods S1
   1.1 NMR spectroscopy S1
   1.2 HPLC-Analysis of enantioselectivity S1
   1.3 Column Chromatography S1
   1.4 Gas Chromatography S1
   1.5 Chemicals, cells, media and genes S2
   1.6 Protein transformation and expression S2
   1.7 Protein purification S3
2. General procedure (GP) S3
   2.1 GP-(A): Activity assay for whole-cell LinD using *rac*-linalool (*rac*-1b) S3
   2.2 GP-(B): Screening of biocatalytic dehydration at a 1-mL scale S4
   2.3 GP-(C): Kinetic resolution of *rac*-1a with LinD S4
   2.4 GP-(D): Chemical dehydration of *rac*-1a using H_2SO_4 as Brønsted acid S6
   2.5 GP-(E): Biocatalytic dehydration of *rac*-1a using LinD as purified protein S6
   2.6 GP-(F): Synthesis of tertiary alcohols using Grignard-reagent S6
   2.7 GP-(G): Determination of the concentration of Grignard-reagent S7
3. Supporting figures S8
   3.1 Figure S1 .... S8
   3.2 Figure S2 .... S9
   3.3 Figure S3 .... S11
   3.4 Figure S4 .... S15
   3.5 Figure. S5 .... S18
   3.6 Figure S6 .... S20
   3.7 Figure S7 .... S21
   3.8 Figure S8 .... S22
   3.9 Figure. S9 .... S23
4. Calibration curves and Chromatograms S23
5. Synthetic Procedures S28
6. NMR spectra S44
7. DNA/AA sequences S92
8. Computations S96
9. References S103

1. MATERIAL AND METHODS

1.1 NMR spectroscopy

^{1}H and ^{13}C NMR spectra were recorded on a Bruker Avance III 500 HD spectrometer working at a frequency of 500 MHz (protons) with 128 scans using CDCl3 as solvent. Chemical shifts (δ) are given in ppm and referenced to the residual solvent peak. Coupling constants (Hz) and signal multiplicity (S = singlet, d = doublet, dd =doublet of doublets, dt = doublet of triplets, ddt = doublet of doublets of triplets, t = triplets, m = multiplets) are noted in the conventional form.

1.2 HPLC-Analysis of enantioselectivity

Enantiomeric ratios were determined by chiral high performance liquid chromatography on a LC2000SFC-HPLC system from Knauer (pumps P 6.1 L, automatic back pressure regulator BP-2080plus, column thermostat CO-2060plus, multi-wavelength detector DAD 2.1 L and autosampler AS 6.1 L). The samples were separated with Chiralpak® IC, OD-H and OJ-H columns from Daicel using hexane/isopropanol as mobile phase, from DAICEL. The chromatograms were evaluated with the software Galaxy Chromatography Data.

1.3 Column Chromatography

Automated preparative flash column chromatography with UV detection at 254 nm and ELSD-detector was performed on a Pure C-850 FlashPrep instrument (Büchi® Labortechnik) and Silica/Celite containing pre-packed columns were utilized for the purification. TLC was performed on Macherey-Nagel's ALUGRAM® SIL G/UV254 TLC sheets (0.2 mm) before.

1.4 Gas Chromatography

Gas chromatography-mass spectrometry measurements were carried out on an AGILENT GC 8860 instrument coupled with an AGILENT 5977B Series mass selective detector. The chiral analyses of the generated alcohols were carried out using an AGILENT CP-Chirasil-Dex CB column (30 m x 250 μm x 0.25 μm) and helium as the carrier gas.

Alternatively, enantiomeric ratios were determined on a SHIMADZU GC-2030 with FID-detector on a BGB-174 (30 m x 250 µm x 0.25 µm) chiral column from the BGB Analytik AG company with N_2 as a carrier gas. The activity test of LinD using *rac*-alcohols was analyzed on a SHIMADZU GC-2030 with FID-detector using Phenomenex ZB-5MSi (30 m x 250 µm x 0.25 µm) as column and N_2 as carrier gas using calibration curves. The GCMS-analysis was performed on a SHIMADZU single quadrupole GCMS-QP2020 NX gas chromatograph-mass spectrometer using Phenomenex ZB-5MSi (30 m x 250 µm x 0.25 µm) as column with H_2 as a carrier gas.

1.5 Chemicals, cells, media and genes

All chemicals and solvents, unless otherwise described, were purchased from commercial suppliers (TCI, abcr, SIGMA ALDRICH, BLDPHARM, THERMO SCIENTIFIC, ENAMINE) and applied without further purification. Isopropyl-ß-D-thiogalactopyranosid (IPTG), Kanamycin and LB media were purchased from CARL ROTH. The *E. coli* cells were sourced from TWIST BIOSCIENCE. The pET28a(+) vector, containing a C-terminal His_6-tag was used for LinD.

1.6 Protein transformation and expression

LinD was constructed with pET28a(+) vector and transformed into *E. coli* BL21(DE3), followed by the preparation of glycerol stocks stored at −80°C. For inoculation, 5 mL LB cultures containing kanamycin as antibiotics (50µg/mL) were prepared from single colonies obtained agar plates or less amount of glycerol stocks, and then incubated overnight at 37°C and 180 rpm. Protein expression was carried out in 2 L culture flasks containing 400 mL LB media and kanamycin (50 µg/mL). Main cultures were inoculated with the overnight pre-cultures to a starting concentration of 1% (v/v) and allowed to grow to an OD_{600} value between 0.6-0.8 at 37 °C and 150 rpm. Afterwards, induction was initiated by adding IPTG to a final concentration of 0.05 mM, followed by further incubation for 5 hours at 37°C. Finally, cells were harvested by centrifugation (30 min, 4000 xg, 4°C) and stored at −20°C for subsequent applications.

1.7 Protein purification

Frozen cell pellets were thawed on ice and resuspended in citrate buffer (50 mM, pH = 6) at a concentration of 300 mg/mL. These cell suspensions were sonicated (Bandelin Sonoplus UW2070) three times each on ice for 3 minutes each, with 5 cycles at 20% power. Cell debris was removed by centrifugation (12,000 rpm, 4°C for 15 min) and the soluble crude extract fraction was filtered through 0.2µM filters before being purified using a HisTrap™ HP 5 mL column loaded with Ni^{2+}. Elution of the crude extract fractions was carried out with an imidazole gradient, starting with 20 mM (4 cv), followed by 40 mM (4 cv), 70 mM (4 cv), 100 mM (4 cv), and 300 mM (5 cv) imidazole concentrations. The enzyme-containing fractions were identified by BCA color formation, combined, and desalted with PD-10 desalting columns packed with SEPHADEX G-25 resin. The desalted protein solution was concentrated using a centrifugal filter tube (10 kDa). Purity was assessed by 12% SDS-PAGE analysis, and concentration was determined using a NANODROP spectrophotometer. Aliquots of purified proteins were stored at −80°C until further use.

2. GENERAL PROCEDURE (GP)

2.1 GP-(A): Activity assay for whole-cell LinD using *rac*-linalool (*rac*-1b)

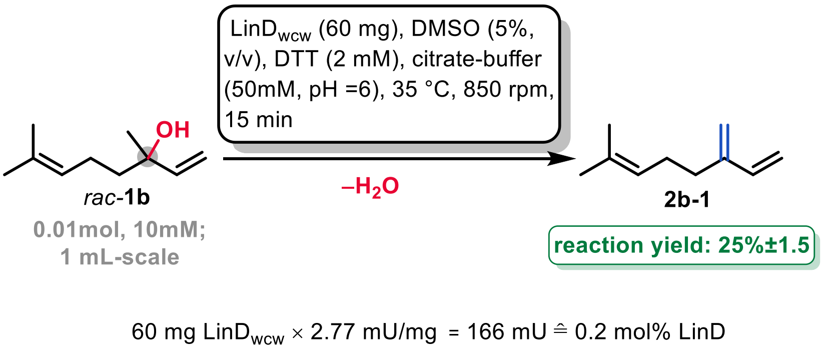

The prepared whole cell catalyst solution (300 mg/ mL,200 µL) was incubated with citrate-buffer (50 mM, pH = 6), DTT (20 mM-stock solution, 100 µL) and *rac*-linalool (*rac*-**1b**, 200 mM-stock solution in DMSO, 50 µL) at 35 °C for 15 min. Meanwhile the reaction was provided on a 1 mL- scale in triplicates. After the 15 min, cyclohexane (800 µL) was added to the mixture and the sample was centrifuged at 20.000g for 2 min.

The organic layer was analyzed by using GC and the purity of olefins was determined by comparison with a calibration curve.

2.2 GP-(B): Screening of biocatalytic dehydration at a 1-mL scale

All reactions were conducted in 2 mL micro reaction vessels and performed in triplicate. In general, tertiary alcohol *rac*-1 (10 mM) was typically dissolved in DMSO (5%, *v*/*v*), 2 mM DTT and LinD as whole cell catalyst (166 mU = 60 mg wet cell weight ≙ 0.2 mol% LinD), determined by activity tests using *rac*-**1b**) was added. The reaction mixture was then incubated for 20 hours at 35 °C and 850 rpm in a thermoshaker. Afterwards, the sample was extracted with organic solvents (800 µL cyclohexane or $CDCl_3$), and the resulting organic fraction was washed with dd. H_2O (3x800 µL), dried with Na_2SO_4. Results were analyzed by ^{1}H-NMR spectroscopy, while enantiomeric excess was determined by chiral HPLC or GC.

2.3 GP-(C): Kinetic resolution of *rac*-1a with LinD (According to the reactions given in Figure 7B)

(A) Preparative-Scale Synthesis (5 mL-scale)

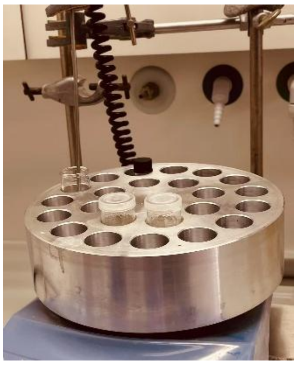

Frozen cells were resuspended in citrate buffer (50 mM, pH 6.0) to a concentration of 300 mg/mL, and their activity was tested using *rac*-**1b** (10 mM) as the natural substrate. Whole-cell LinD (733 mU; 265 mg wet cell weight; 0.004 mol%) was used to catalyze the dehydration of *rac*-**1a** (500 mM, 2.5 mmol) on a 5 mL scale. The reaction was carried out in the presence of DMSO (5%, v/v) and DTT (2 mM) at 35 °C and 850 rpm for 20 h.

Following extraction with ethyl acetate (3 x 5 mL) and solvent removal, the reaction yield of the crude product was determined by ^{1}H NMR spectroscopy. The enantiomeric excess (*ee*) was analyzed by chiral HPLC (CHIRALPAK® IC, 4.6 mm × 250 mm) using a mobile phase of *n*-hexane/2-propanol (98:2), a flow rate of 1.0 mL/min, and UV detection at 250 nm.

The Hofmann olefin was isolated by automated column chromatography (BÜCHI; column: FP ECOFLEX Si 25 g; eluent: cyclohexane/DCM = 9:1).

For the isolation of (*R*)-**1a**, LinD (146 mU; 53 mg wet cell weight; 0.004 mol%) was applied under the same reaction conditions for the dehydration of residual alcohol (100 mM, 0.5 mmol; *R*/*S* = 94:6) on a 5 mL scale. After extraction with ethyl acetate (3 x 5 mL) and solvent removal, the *R*-alcohol was isolated by column chromatography (BÜCHI; column: FP ECOFLEX Si 25 g cyclohexane/DCM = 9:1) and analyzed by chiral HPLC and ^{1}H NMR spectroscopy.

(B) Preparative-Scale Synthesis (50 mL-scale)

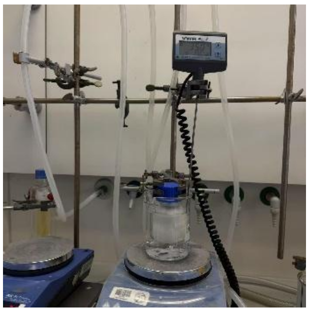

Frozen cells were resuspended in citrate buffer (50 mM, pH 6.0) to a concentration of 300 mg/mL, and their activity was tested using *rac*-**1b** (10 mM) as the natural substrate. Whole-cell LinD (7330 mU; 2.650 g wet cell weight; 0.004 mol%) was used to catalyze the dehydration of *rac*-**1a** (500 mM, 25 mmol) on a 50 mL scale. The reaction was carried out in the presence of DMSO (5%, v/v) and DTT (2 mM) at 35 °C and 850 rpm for 20 h.

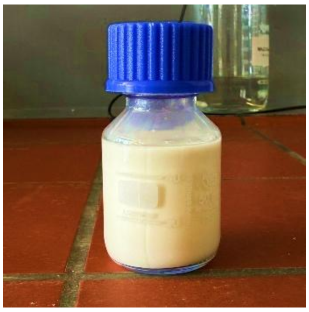

After extraction with ethyl acetate (3 x 50 mL) and solvent removal, the reaction yield was determined by ^{1}H NMR spectroscopy. The enantiomeric excess (*ee*) was analyzed by chiral HPLC (CHIRALPAK® IC, 4.6 mm × 250 mm) using a mobile phase of *n*-hexane/2-propanol (98:2) at a flow rate of 1.0 mL/min and a detection wavelength of 250 nm (λ = 250 nm). The Hofmann product **2a-1** was isolated by automated column chromatography (BÜCHI; column: FP ECOFLEX Si 80 g; eluent: cyclohexane/EtOAc = 9:1).

For the isolation of (*R*)-**1a**, LinD (3500 mU; 1.265 g wet cell weight; 0.004 mol%) was used under the same reaction conditions to dehydrate residual alcohol (240 mM, 12 mmol; *R*/*S* ratio = 85:15) on a 50 mL scale, followed by extraction with ethyl acetate (3 x 50 mL) and isolation via column chromatography (BÜCHI; column: FP ECOFLEX Si 40 g cyclohexane/EtOAc = 9:1) and analysis by chiral HPLC and ^{1}H NMR spectroscopy.

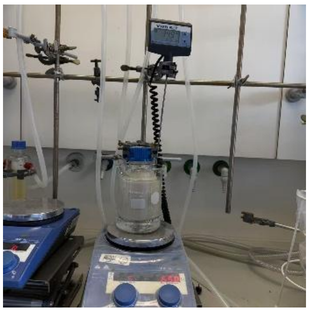

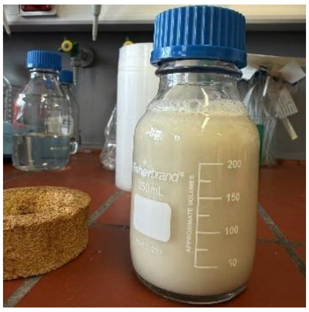

For the isolation of the Saytzeff product **2a-2**, LinD (41.55 U; 15.000 g wet cell weight; 0.2 mol%) was applied under the same conditions to dehydrate (*R*)-**1a** (10 mM, 2.5 mmol) on a 250 mL scale. After extraction with ethyl acetate (4 x 200 mL) and solvent removal, **2a-2** was purified by column chromatography (FP ECOFLEX Si 25 g, cyclohexane/EtOAC = 9:1) and characterized by ^{1}H NMR spectroscopy.

2.4 GP-(D): Chemical dehydration of *rac*-1a using H_2SO_4 as Brønsted acid

Different amount of sulfuric acid (X mol%) was incubated with citrate-buffer (50 mM, pH = 6), DTT (20 mM-stock solution, 100 µL) and *rac*-**1a** 10 mM) with DMSO (5%, v/v) at 35 °C for 20 h. Meanwhile the reaction was provided on a 1 mL- scale in triplicates. After the reaction time, $CDCl_3$ as deuterated solvent (800 µL) was added to the mixture and the sample was centrifuged at 20.000g for 2 min. The organic layer was washed with water (3 x 800 µL) and product formation was analyzed via quantitative ^{1}H-NMR.

2.5 GP-(E): Biocatalytic dehydration of *rac*-1a using LinD as purified protein

Different amount of protein (X mol%) was incubated with citrate-buffer (50 mM, pH = 6) or toluene-d_8, DTT (20 mM-stock solution, 100 µL) and ***rac*-1a** 10 mM) with DMSO (5%, v/v) at 35 °C for Y h. Meanwhile the reaction was provided on a 1 mL- scale in triplicates. After the reaction time, $CDCl_3$ as deuterated solvent (800 µL) was added to the mixture and the sample was centrifuged at 20.000g for 2 min. The organic layer was washed with water (3 x 800 µL) and product formation was analyzed via quantitative ^{1}H-NMR.

2.6 GP-(F): Synthesis of tertiary alcohols using Grignard-reagent

A solution of vinylmagnesium bromide in THF (20 mmol) under Argon atmosphere was prepared. Ketone (18 mmol) was dissolved in fresh distilled THF (dry, 10 mL) and added dropwise under stirring at 0°C. After stirring at room temperature for 12 hours, the reaction mixture was quenched with a saturated ammonia chloride solution.

Subsequently, the aqueous layer was extracted with diethyl ether (three times with 25 mL). The combined extracts were dried over $MgSO_4$ and after removing the solvent *in vacuo*, crude product was purified by using column chromatography (cyclohexane/ ethyl acetate 9:1).

2.7 GP-(G): Determination of the concentration of Grignard-reagent

A mixture of vinylmagnesium bromide in THF (2 mL), 1,10-phenanthroline (2 mg) as an indicator as well as THF (dry, 10 mL) was suspended in a Schlenk flask under Argon and stirred using a magnetic stirrer. Subsequently, a solution of 2-butanol in toluene (1M) was prepared in a 3 mL-syringe for titration. The alcohol solution was dropwise added until a color change from purple to yellow could be observed and the required volume was recorded for the calculation of the Grignard-concentration as shown below. The reaction is performed in triplicate.

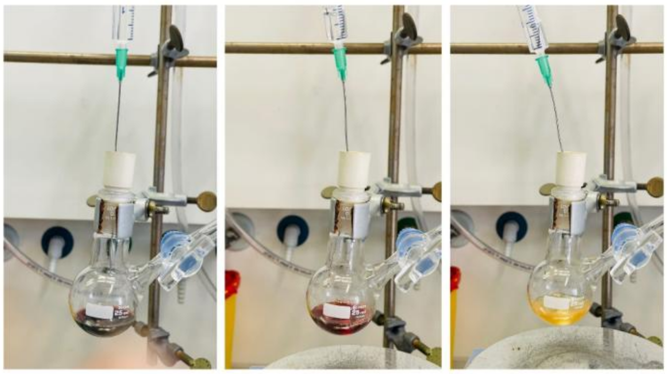

Step 1 **Step 2** **Step 3**

3. SUPPORTING FIGURES

3.1 Figure S1

Different synthetic pathways for alcohol (de)-hydrations are shown below.

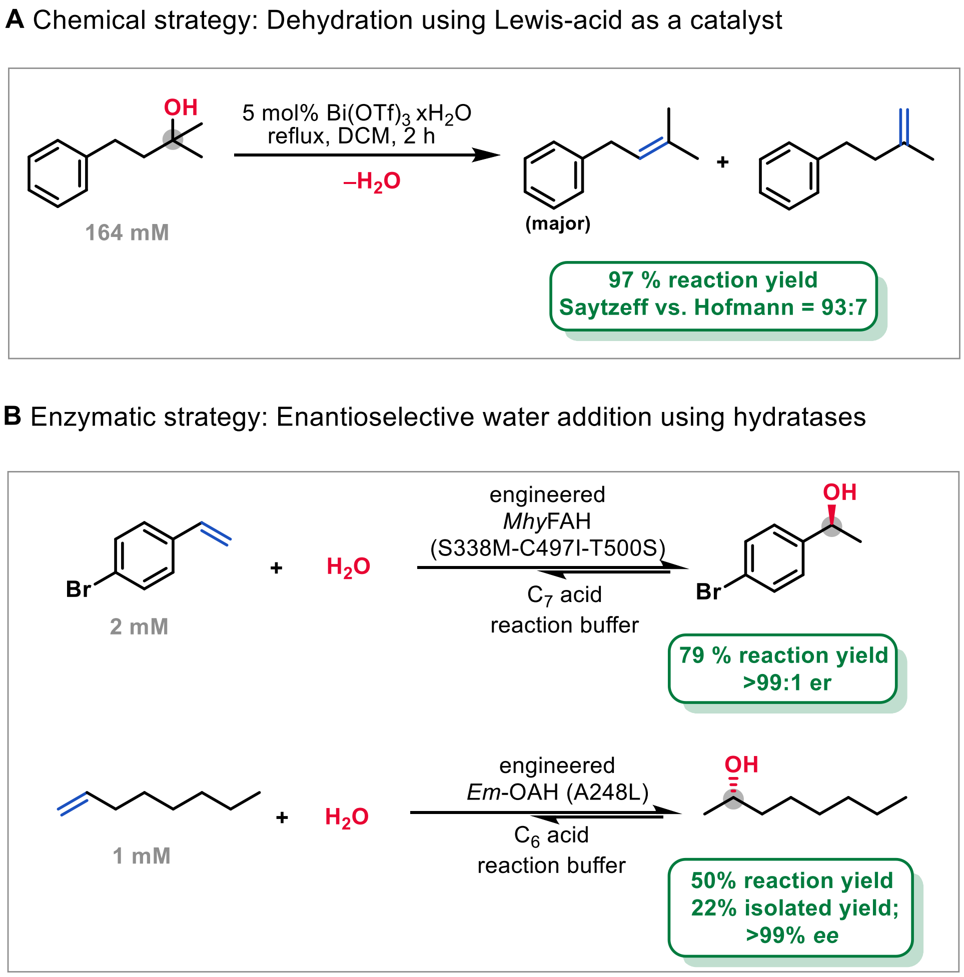

Figure S1. Synthetic routes for hydration and dehydration reactions. (**A**) Synthetic approach to obtain olefin using chemocatalyst is the acid-catalyzed elimination reaction. $Bi(OTf)_3{\bullet}xH_2O$ serves as a Lewis-acid for the dehydration of tertiary alcohols into Saytzeff-alkenes using dichloromethane (DCM) as solvent.[1] (**B**) Asymmetric olefin hydration of alcohols via engineered hydratases.[2,3]

3.2 Figure S2

Dehydration of (*R*)-**1a** (10 mM) was carried out using whole-cell LinD (166 mU; 0.2 mol%) for 20 hours. Enzyme activity was determined using *rac*-linalool (*rac*-**1b**) as a reference substrate. Reactions were conducted at 35°C with shaking at 850 rpm for 20 hours and subsequently quenched by adding 800 µL of $CDCl_3$. The organic phase was subsequently washed with ddH_2O (3 × 800 µL) and analyzed via ^{1}H-NMR. Additional control experiments, including reactions without enzymes or with *E. coli* cells lacking the LinD insert, were prepared in triplicate. No racemization of (*R*)-**1a** was observed in control experiments using *E. coli* cells without the LinD insert. Significant signals corresponding to the formation of the Saytzeff product **2a-2** are marked with '♥'. By comparison with the negative control experiments, only the Saytzeff product **2a-2** was detected.

A Dehydration of (*R*)-1a with whole-cell LinD

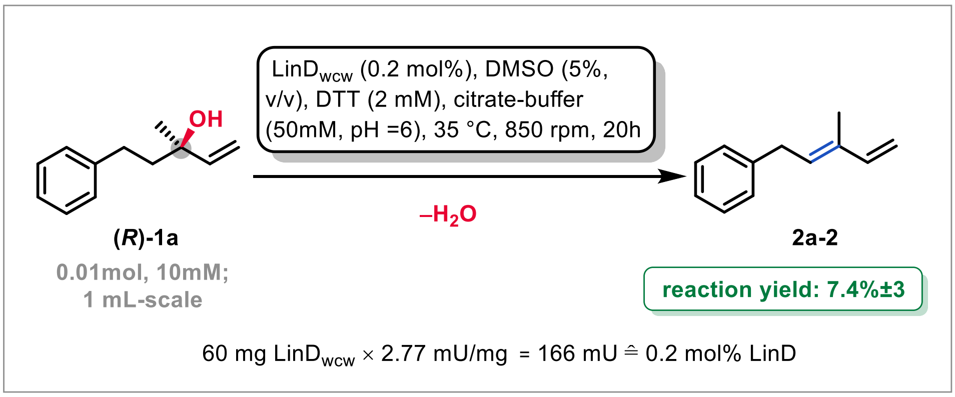

B ^{1}H-NMR: comparison of control and dehydration reactions

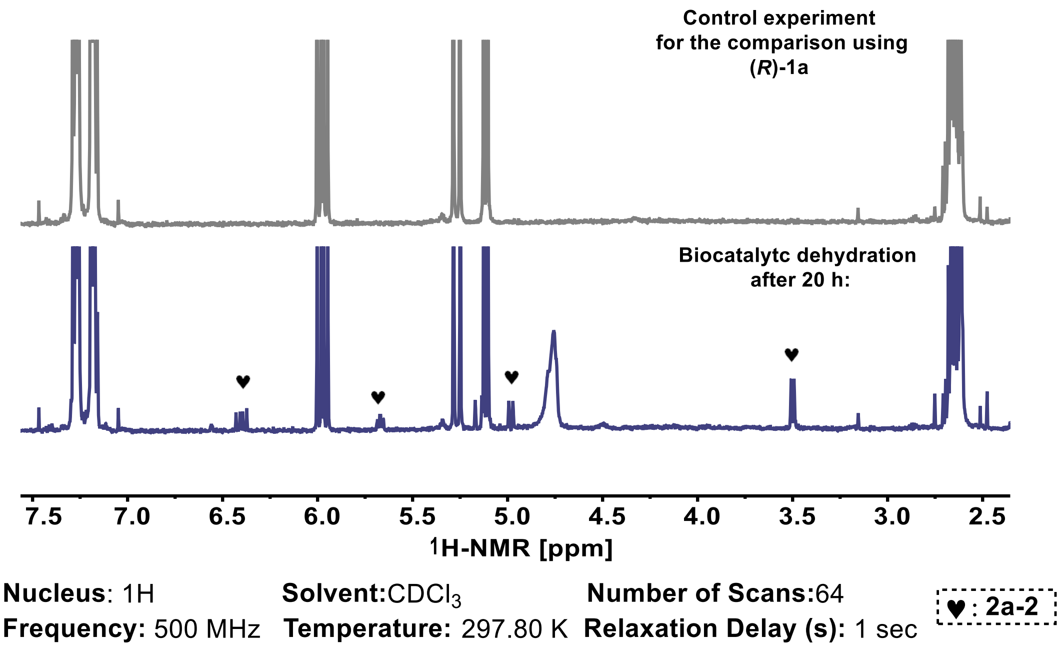

C ^{1}H-NMR: reference spectra of compound **2a-1**

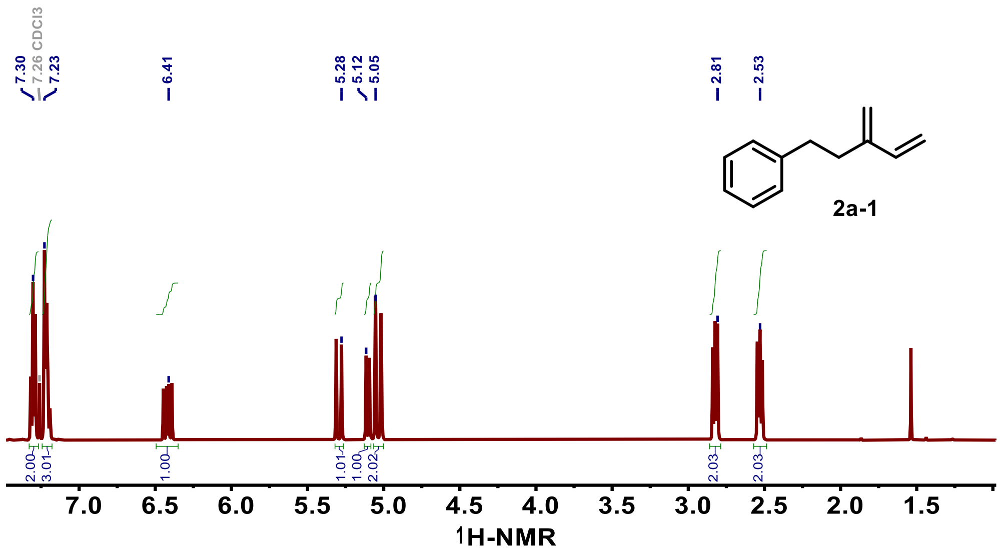

2a-1: ^{1}H NMR (500 MHz, $CDCl_3$): δ 7.30 (t, 2H), 7.24 (m, 3H), 6.41 (dd, 1H), 5.28 (dd, 1H), 5.12 (dd, 1H), 5.05 (m, 2H), 2.81 (m, 2H), 2.53 (m, 2H).

D ^{1}H-NMR: reference spectra of compound **2a-2**

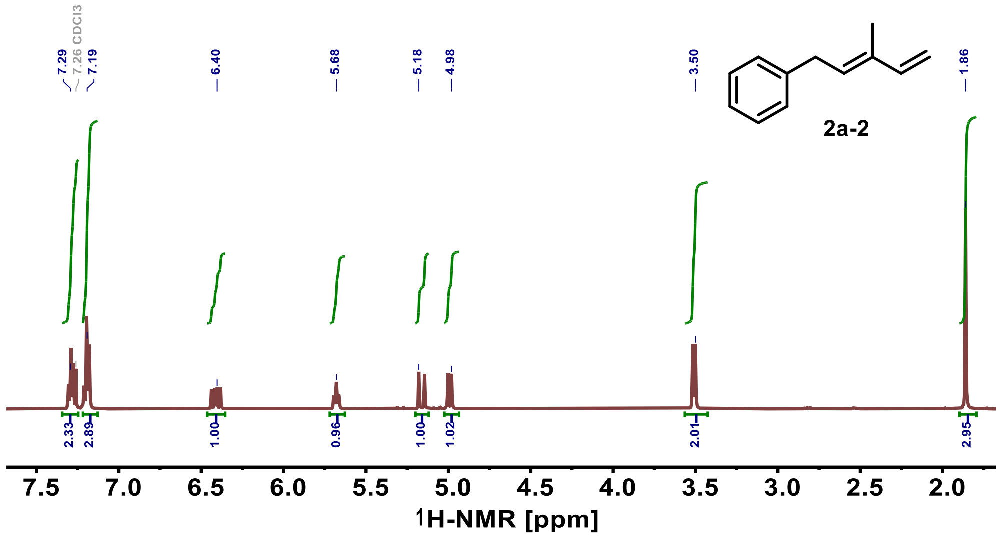

2a-2: ^{1}H NMR (500 MHz, $CDCl_3$): δ 7.29 (m, 2H), 7.19 (m, 3H), 6.40 (dd, 1H), 5.68 (t, 1H), 5.18 (dd, 1H), 4.98 (dd, 1H), 3.50 (d, 2H), 1.86 (s, 3H).

Figure S2. Evaluation of LinD-catalyzed dehydration of (*R*)-**1a** with whole-cell catalysts.

Reference Data[18]**:**

2a-3: ^{1}H NMR (500 MHz, $CDCl_3$): δ 7.31 – 7.15 (m, 5H), 6.91 (dd, 1H), 5.56 (t, 1H), 5.28 (d, 1H), 5.09 (d, 1H), 3.52 (d, 2H), 1.87 (d, 3H).

3.3 Figure S3

(*R*)-Linalool was purchased from Thermo Scientific Fisher® and is commercially available with a purity of 95%. LinD-catalyzed dehydration of 10 mM (*R*)-**1b** was performed using 166 mU (equivalent to 0.2 mol% LinD) of whole-cell catalyst for 20 hours. Enzyme activity was determined using *rac*-linalool (*rac*-**1b**) as a reference substrate. Reactions were conducted at 35°C with shaking at 850 rpm, and product formation was analyzed by chiral GC and ^{1}H-NMR.

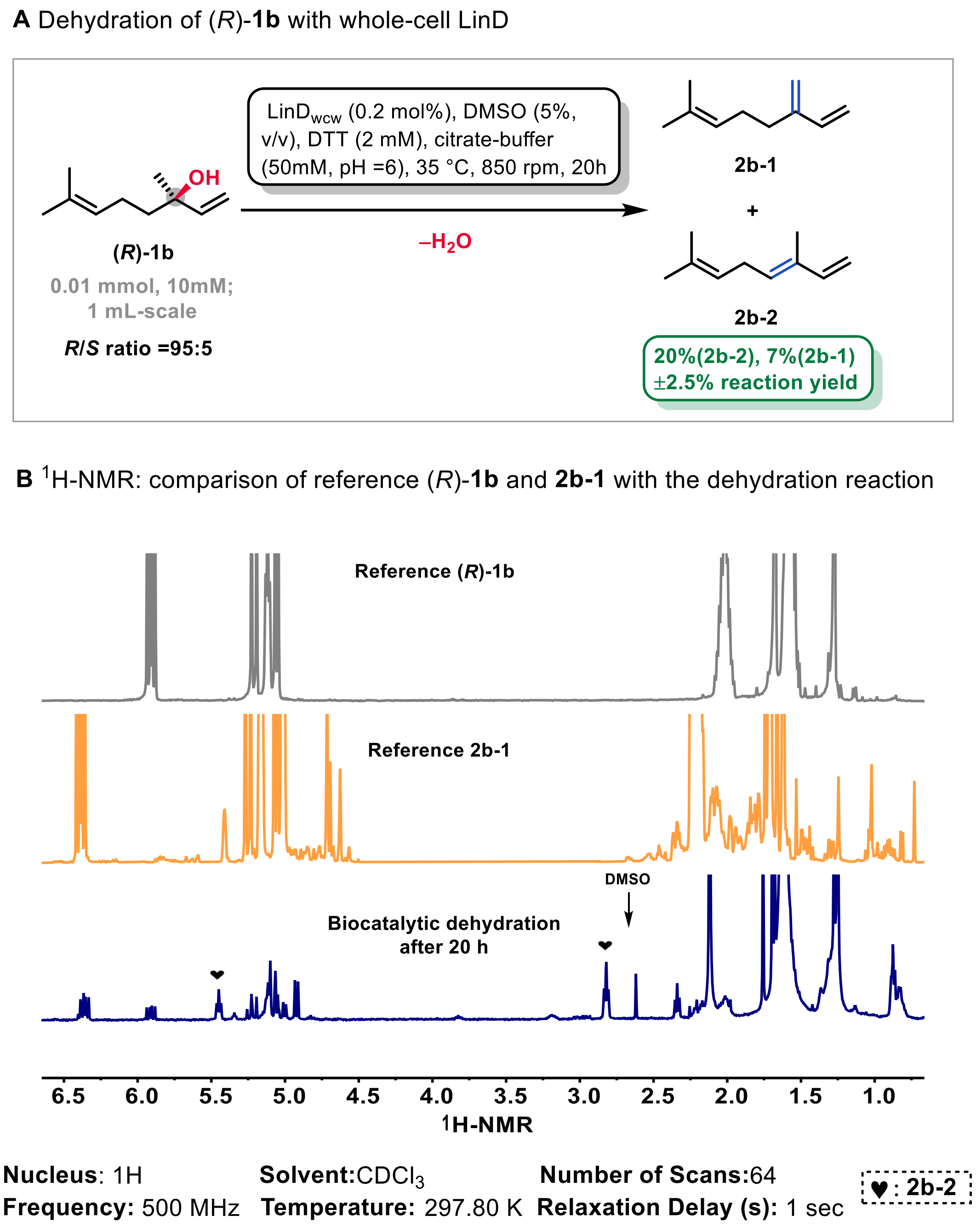

Figure S3-1. Evaluation of whole-cell LinD-catalyzed dehydration of (*R*)-**1b** via ^{1}H-NMR.

Negative control experiments using *E. coli* cells lacking the LinD insert confirmed that dehydration was strictly enzyme dependent. No detectable racemization of (*R*)-**1b** was observed in control experiments using *E. coli* cells lacking the LinD insert. All reactions were performed in triplicate. Significant signals corresponding to the formation of the Saytzeff product **2b-2** are marked with '♥'. A representative section of the ^{1}H-NMR spectra is shown below. By comparison with the reference spectra of substrate (*R*)-**1b** and β-myrcene (**2b-1**), the formation of the Saytzeff product **2b-2** as the main product was confirmed. Building on these findings, we extended our approach to the dehydration of (*R*)-**1b** using purified LinD (0.3 mol%) under identical conditions for 20 h. Product analysis by ^{1}H-NMR and chiral GC confirmed the formation of the (*E*)-Saytzeff product (**2b-2**) as the major product, with a reaction yield of 24%, as demonstrated by the representative ^{1}H-NMR spectrum. Reference data for **2b-1** and **2b-2** from the literature are included as additional information. Furthermore, the (*Z*)-Saytzeff product was not detected. Reference spectra of commercial **2b-1** and (*Z*)-β-ocimene, as the (*Z*)-Saytzeff isomer, are provided below.

A Dehydration of (*R*)-**1b** with purified LinD

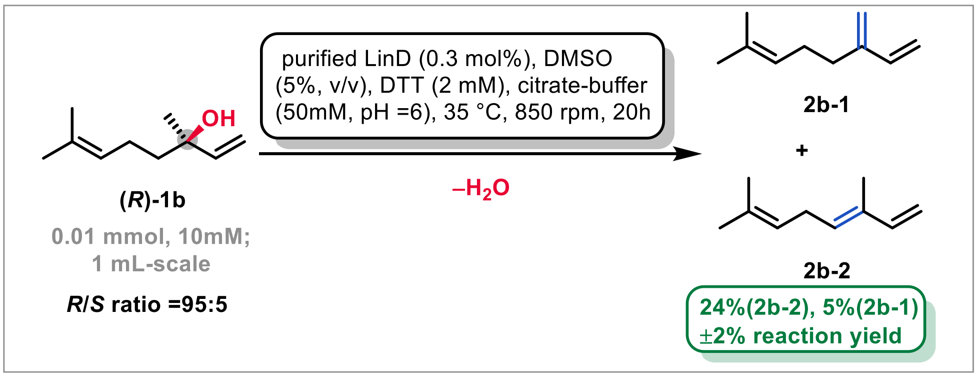

B ^{1}H-NMR: Dehydration of (*R*)-**1b** with purified LinD

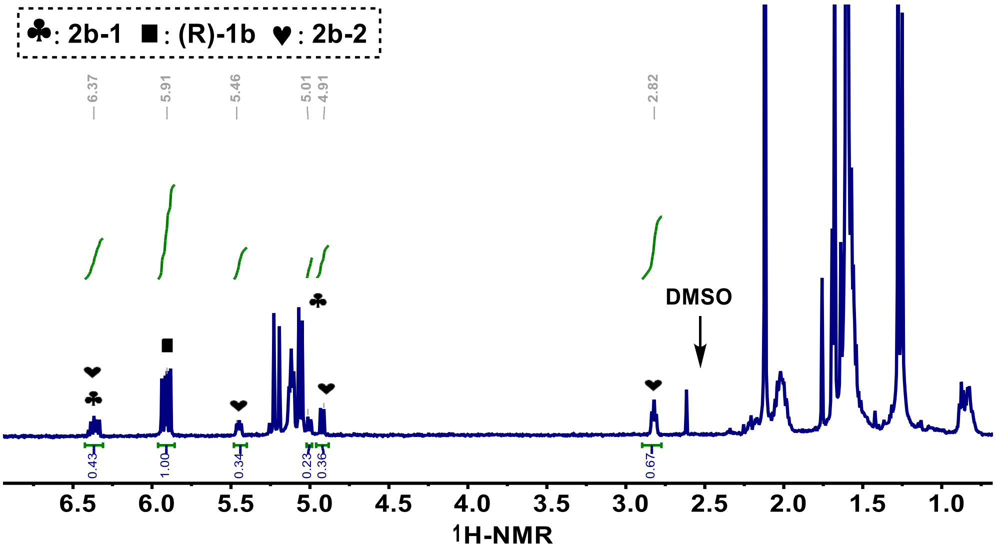

Nucleus: 1H **Solvent:**$CDCl_3$ **Number of Scans:**64
Frequency: 500 MHz **Temperature:** 297.80 K **Relaxation Delay (s):** 1 sec

Reference Data:

2b-1: ^{1}H NMR (500 MHz, $CDCl_3$): δ 6.39 (dd, 1H), 5.27 (dd, 1H), 5.16 (m, 1H), 5.02 (m, 3H), 2.20 (m, 4H), 1.72 (s, 3H), 1.62(s, 3H).

2b-2[19]: ^{1}H-NMR (300 MHz, $CDCl_3$) δ 6.37 (dd, 1H), 5.46 (t, 1H), 5.16–5.07 (m, 2H), 4.94 (d, 1H), 2.84 (t, 2H), 1.77 (s, 3H), 1.71 (s, 3H), 1.65 (s, 3H).

C ^{1}H-NMR: reference spectra of compound (*Z*)-β-ocimene

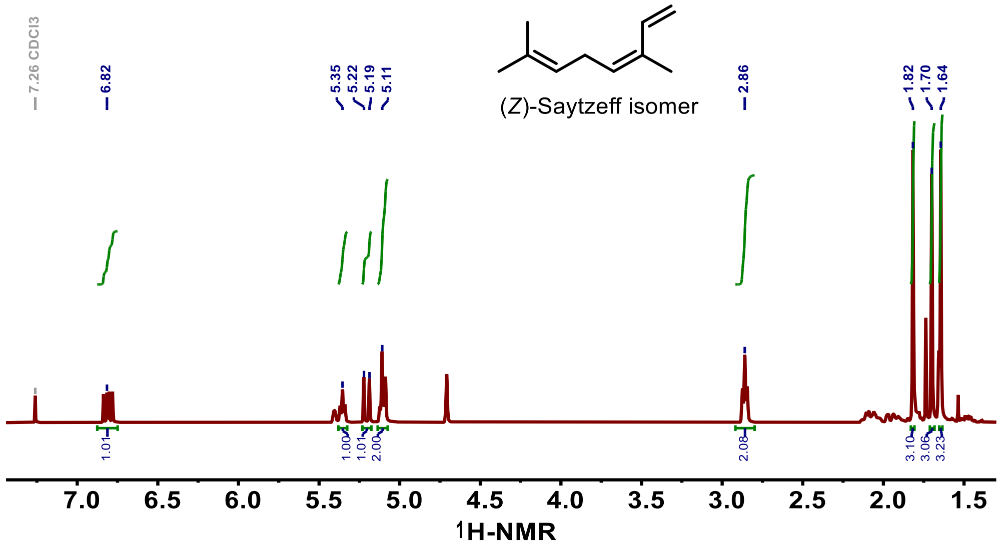

(*Z*)-Saytzeff product: ^{1}H NMR (500 MHz, $CDCl_3$): δ 6.82 (dd, 1H), 5.35 (t, 1H), 5.22-5.19 (dd, 1H), 5.11 (m, 2H), 2.86 (t, 2H), 1.82 (s, 3H), 1.70 (s, 3H), 1.64 (s, 3H).

D ^{1}H-NMR: reference spectra of compound **2b-1**

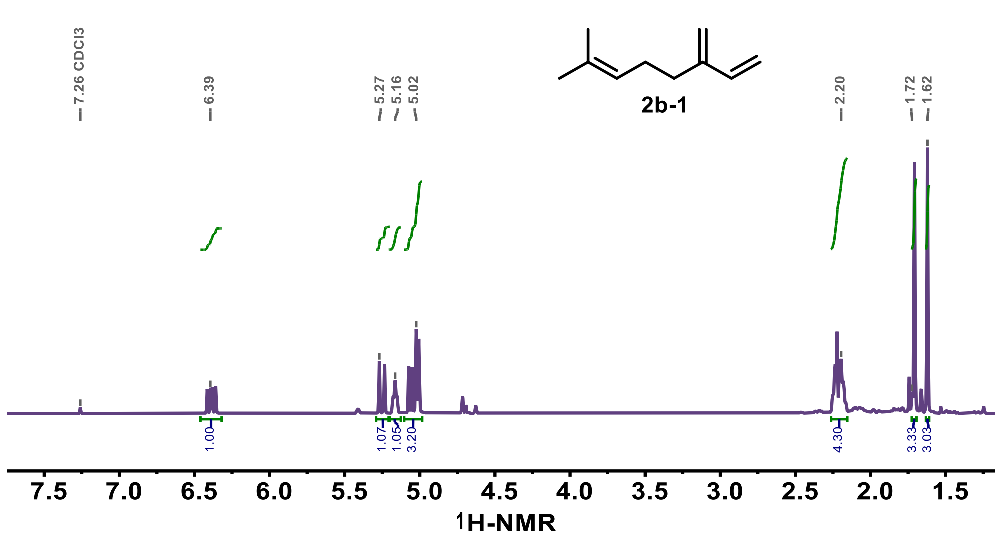

2b-1: ^{1}H NMR (500 MHz, $CDCl_3$): δ 6.39 (m, 1H), 5.27 (dd, 1H), 5.16 (m, 1H), 5.02 (m, 3H), 2.20 (m, 4H), 1.72 (s, 3H), 1.62(s, 3H).

Figure S3-2. Evaluation of purified LinD-catalyzed dehydration with (*R*)-**1b**. ^{1}H-NMR reference spectra of (*Z*)-β-ocimene and β-myrcene (**2b-1**). The reference data of (*Z*)-β-ocimene are in agreement with the literature[20].

Furthermore, chiral-GC analysis confirmed our findings. Chromatogram A displayed the reference spectrum of commercially sourced (*R*)-**1b** with an enantiomeric ratio of 95:5, showing retention times of t_R((*R*)-**1b**) = 35.04 min and t_R((*S*)-**1b**) = 37.02 min. Chromatogram B presented the reference spectrum of commercially obtained **2b-1**. Chromatogram C demonstrated the biocatalytic dehydration catalyzed by purified LinD, yielding the Saytzeff product at t_R (**2b-2**) =10.69 min, along with a minor fraction of the Hofmann product at t_R (**2b-1**) =7.10 min.

A Chiral GC Chromatogram of Reference (*R*)-**1b** (Purity 95%)

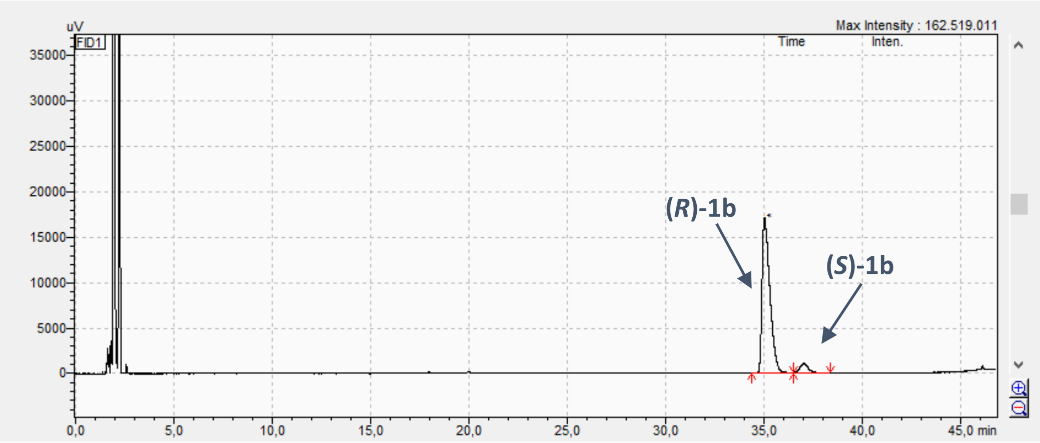

Results View - Peak Table

Peak Table | Compound | Group | Calibration Curve

Peak#	Ret. Time	Area	Height	Mark	Conc.	Unit	ID	N	Area%
1	35,039	470303	17093	M	0,000				94,268
2	37,016	28596	1022	M	0,000				5,732
Total		498899	18115		0,000				100,000

B Chiral GC Chromatogram of Reference **2b-1**

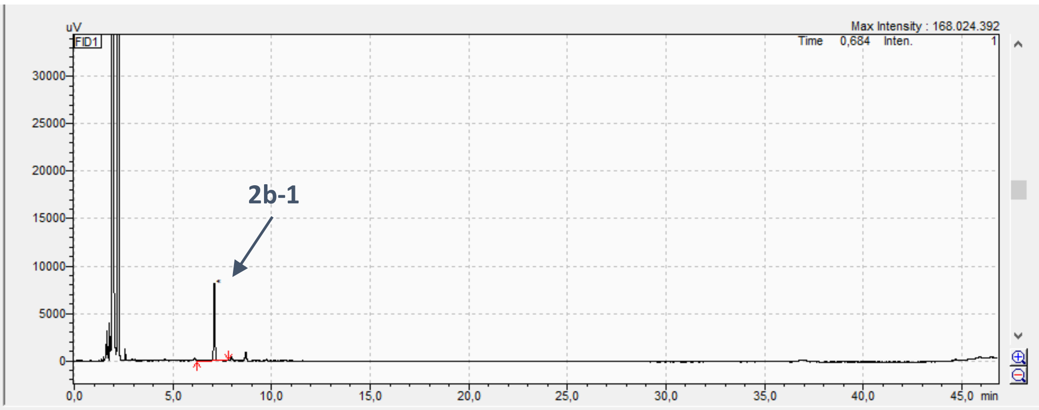

Results View - Peak Table

Peak Table | Compound | Group | Calibration Curve

Peak#	Ret. Time	Area	Height	Mark	Conc.	Unit	ID	N	Area%
1	7,101	38006	8133	M	0,000				100,000
Total		38006	8133		0,000				100,000

C Example: Chiral GC Chromatogram of dehydration of **(*R*)-1b** (purity 95%)

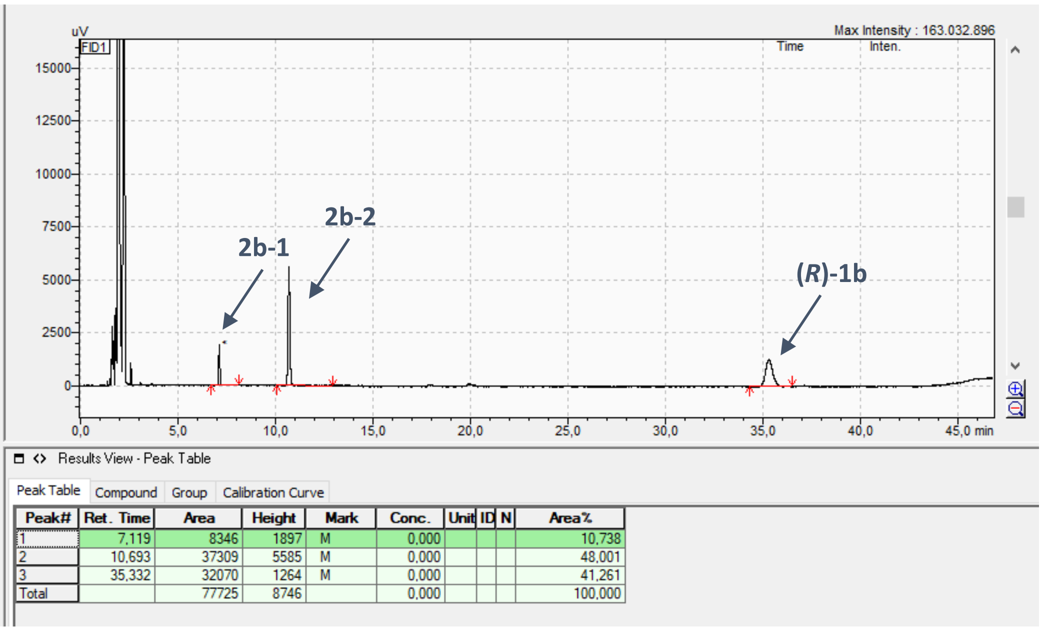

Peak#	Ret. Time	Area	Height	Mark	Conc.	Unit	ID	N	Area%
1	7,119	8346	1897	M	0,000				10,738
2	10,693	37309	5585	M	0,000				48,001
3	35,332	32070	1264	M	0,000				41,261
Total		77725	8746		0,000				100,000

Figure S3-3. Chiral GC chromatograms of (*R*)-**1b** and **2b-1** as reference as well as the biotransformation of (*R*)-**1b** using LinD.

3.4 Figure S4

We further investigated the biotransformation using purified LinD as the biocatalyst. Initially, the dehydration reaction was carried out with 0.03 mol% enzyme. After 24 hours, both the Hofmann product (**2b-1**) and (*R*)-linalool ((*R*)-**1b**) were detected via chiral GC analysis. To study the reaction kinetics, an additional 0.03 mol% enzyme was introduced, and the transformation was monitored over time. Notably, through kinetic resolution, we confirmed that (*R*)-linalool ((*R*)-**1b**) could also be dehydrated by LinD. With the optimal enzyme load, the reaction exclusively yielded the Hofmann olefin after 24 hours, reaching the peak of the kinetic resolution. Upon introducing more purified LinD, the formation of the Saytzeff product (**2b-2**) was observed within two hours, and its yield progressively increased over time, supporting our hypothesis of a shift in product distribution with varying protein concentrations. Chiral GC Chromatogram reveals that upon adding additional portion of purified LinD after 24 hours, the Saytzeff product (t_R(**2b-2**) = 10.67 min) was detected within 6 hours, confirming the formation of the Saytzeff product **2b-2**.

This approach further demonstrates the high activity of purified LinD. Even with the addition of a minimal amounts (0.03 mol%) of purified protein, the Saytzeff product **2b-2** was detectable within 6 hours of the reaction.

A Dehydration of *rac*-**1b** with purified LinD

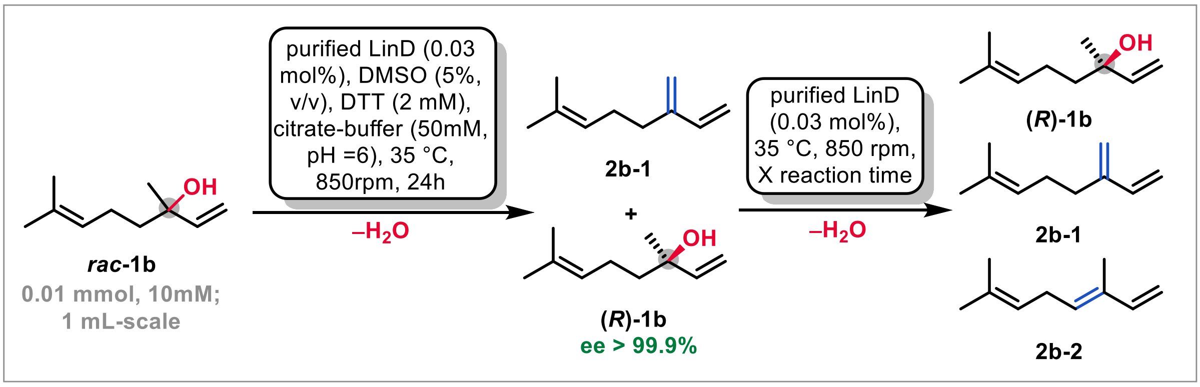

B Time course of dehydration with *rac*-**1b**

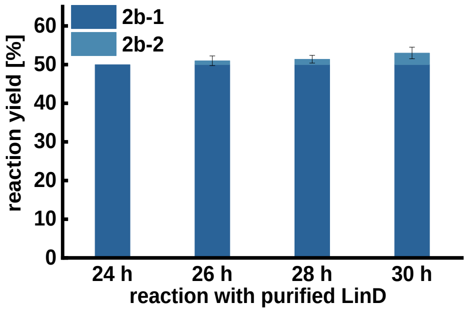

C Example: Chiral GC Chromatogram of reaction with ***rac*-1b** after addition of LinD (t = 24h+6h)

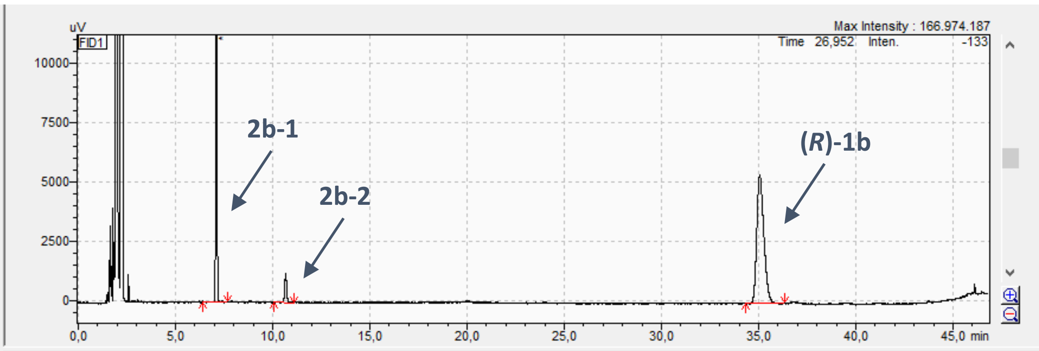

Results View - Peak Table

Peak Table | Compound | Group | Calibration Curve

Peak#	Ret. Time	Area	Height	Mark	Conc.	Unit	ID	N	Area%
1	7,103	72583	16636	M	0,000				33,186
2	10,668	8288	1190	M	0,000				3,789
3	35,045	137843	5404	M	0,000				63,024
Total		218713	23230		0,000				100,000

Figure S4-1. Evaluation of purified LinD-catalyzed dehydration with *rac*-**1b** following enzyme addition.

Additionally, instead of adding 0.03 mol% purified LinD in two steps, a single addition of 0.06 mol% purified LinD resulted in predominant formation of the Hofmann product **2b-1** after 24 hours. Minor amounts of (*S*)-**1b** and the Saytzeff product **2b-2** were also detected by GC, as shown below.

A Dehydration of *rac*-**1b** with purified LinD

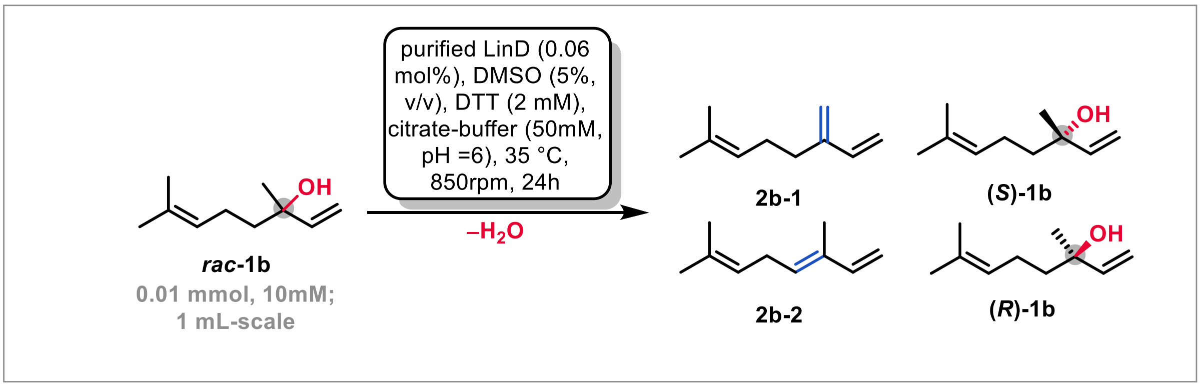

B Example: Chiral GC Chromatogram of reaction with *rac*-**1b** after 24h

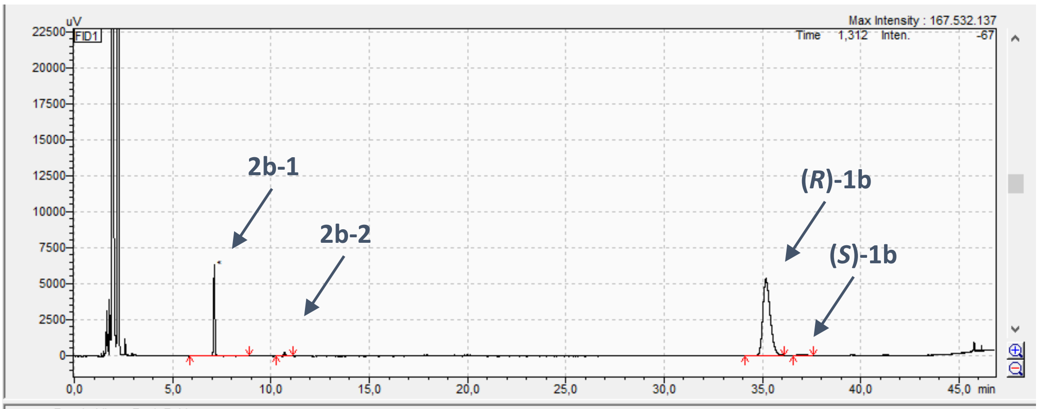

Results View - Peak Table

Peak Table | Compound | Group | Calibration Curve

Peak#	Ret. Time	Area	Height	Mark	Conc.	Unit	ID	N	Area%
1	7,118	30082	6353	M	0,000				17,797
2	10,702	1545	226	M	0,000				0,914
3	35,195	136119	5367	M	0,000				80,529
4	37,050	1285	64	M	0,000				0,760
Total		169032	12010		0,000				100,000

Figure S4-2. Evaluation of 0.06 mol% purified LinD-catalyzed dehydration with *rac*-**1b** for 24 hours.

3.5 Figure. S5

We carried out the biocatalytic dehydration of *rac*-**1a** on a 5 mL scale using 5% DMSO as a co-solvent and 2 mM DTT as a reducing agent. By reducing the amount of wet biomass, suitable reaction conditions were identified to avoid the formation of the (*E*)-Saytzeff product (**2a-2**), achieving >47% reaction yield to the Hofmann product **2a-1**. Considering the theoretical maximum of 50% reaction yield for kinetic resolution, this result represents a good to excellent outcome. As the reaction was performed in aqueous media and both the substrate and product are water-insoluble, DMSO could be readily removed by aqueous washing following extraction with organic solvent. The product **2a-1** was isolated via column chromatography in 30% isolated yield. In the second step, the remaining alcohol with an *R*/*S* ratio of 94:6 was subjected to the same biocatalytic dehydration on a 5 mL scale. After purification by column chromatography, (*R*)-**1a** was isolated in 67% yield.

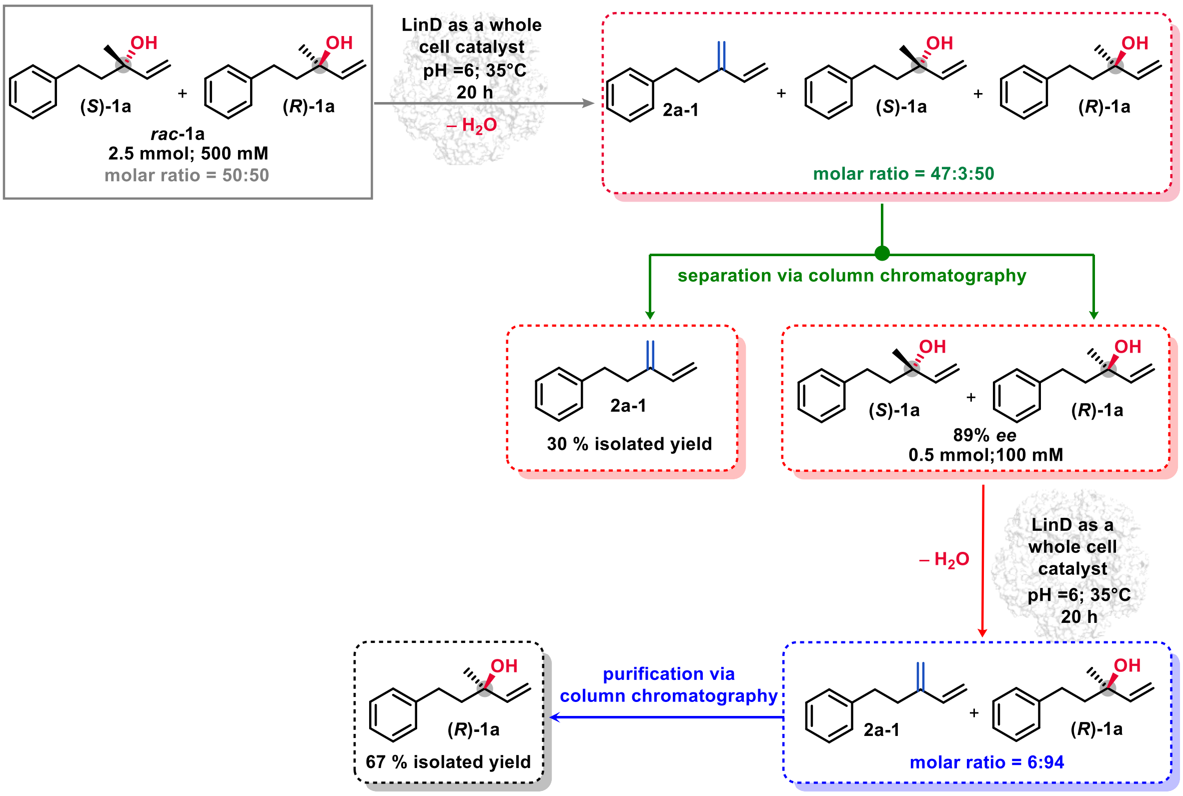

Figure S5-1. An overview of the biosynthetic concept for the isolation of **2a-1** and (*R*)-**1a** using kinetic racemate resolution as a key methodology on a preparative scale. DMSO 5% was performed as co-solvent and 2 mM DTT was utilized as reducing agent.

Based on these encouraging results, we performed the biocatalytic dehydration of *rac*-**1a** on a 50 mL scale using 5% DMSO as a co-solvent and 2 mM DTT as a reducing agent. Applying the same strategy as used for the 5 mL-scale experiment, formation of the (*E*)-Saytzeff product (**2a-2**) was effectively prevented, and a reaction yield of 41% for the Hofmann product **2a-1** was achieved. DMSO was removed by simple aqueous washing following organic solvent extraction, and **2a-1** was isolated by column chromatography in 33% isolated yield. In the second step, the remaining alcohol with an *R*/*S* ratio of 82:18 was subjected to the same dehydration conditions on a 50 mL scale, affording (*R*)-**1a** in 62% isolated yield after purification by column chromatography. In the third step, the dehydration of (*R*)-**1a** was carried out on a 250 mL scale using 5% DMSO and 2 mM DTT for 20 h. A 33% reaction yield of **2a-2** was obtained, and **2a-2** was isolated in 20% isolated yield by column chromatography.

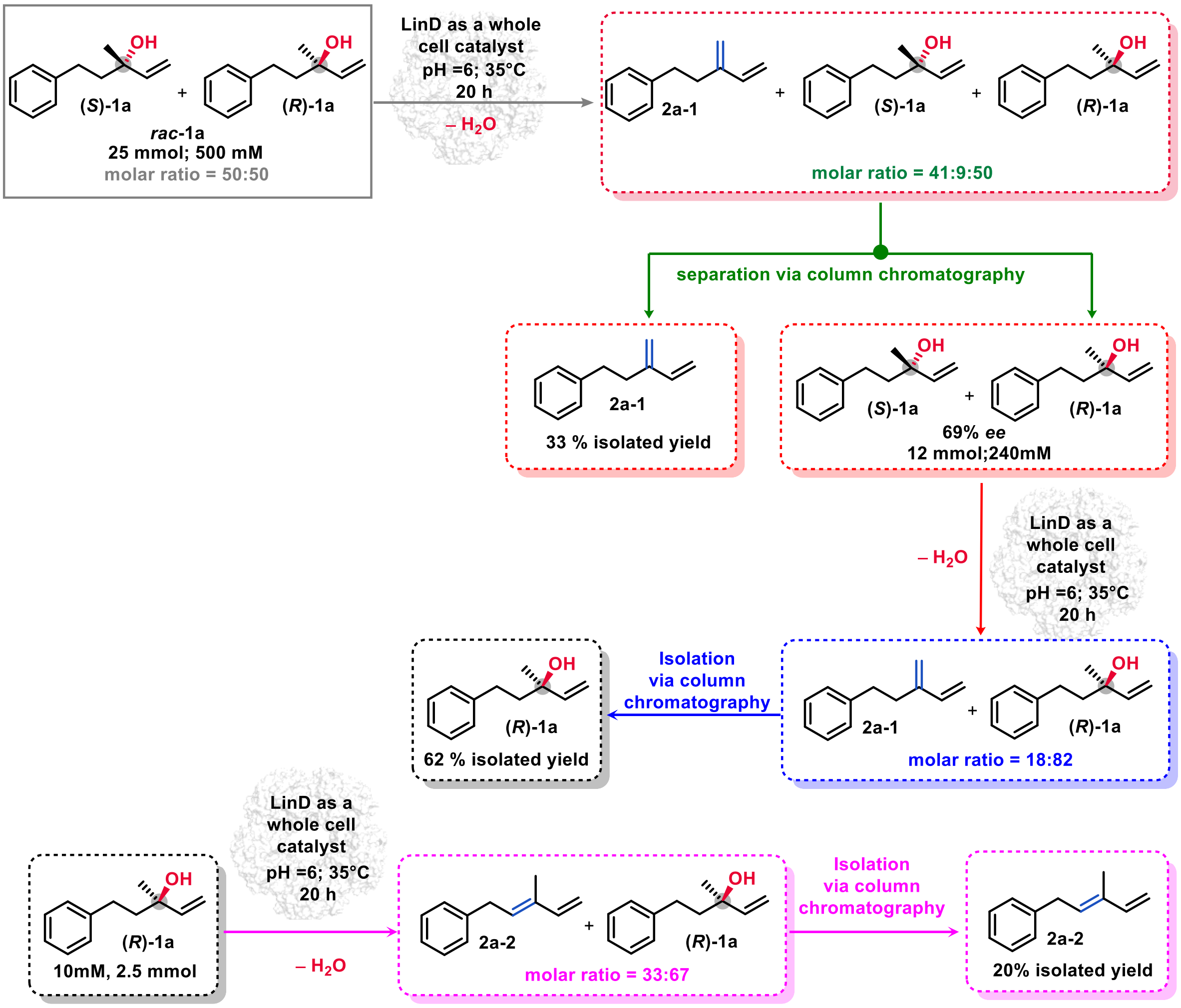

Figure S5-2. Overview of the biosynthetic strategy for the preparative-scale isolation of **2a-1**, **2a-2**, and (*R*)-**1a** using kinetic resolution of the racemate as the key methodology. DMSO (5%) was used as co-solvent, and DTT (2 mM) was employed as a reducing agent. Both **2a-2** and (*R*)-**1a** were isolated on the gram scale.

3.6 Figure S6

For further applications, we tested LinD as a whole-cell catalyst in both lyophilized and immobilized forms using the superabsorber FAVOR®, achieving promising results. Superabsorbent polymers were employed for the immobilization of LinD in this study. These specialized polymer gels are capable of absorbing significant amounts of liquid, typically consisting of crosslinked polyelectrolyte networks with carboxylate anions and sodium cations, as depicted below.[4] Specifically, we utilized the superabsorbent polymer FAVOR® (EVONIK), a polyacrylate copolymer, for whole-cell immobilization.

For the immobilization process, 2 mg of FAVOR® superabsorbent polymer was combined with wet biomass. All reactions were conducted on a 5 mL scale with 100 mM ***rac*-1a** as the substrate, performed in duplicate. The reactions ran for 20 hours at 35 °C, with 5% DMSO as a co-solvent and 2 mM DTT as a reducing agent. Employing whole-cell LinD in different preparations resulted in comparable catalytic performance after 20 h. Therefore, we believe that these findings demonstrate that whole-cell LinD maintains robust activity regardless of the preparation method, highlighting its operational flexibility. This consistency across different catalyst preparations positions whole-cell LinD as a promising biocatalyst for scalable and sustainable terminal alkene synthesis.

A General description of the immobilization strategy using whole-cell LinD

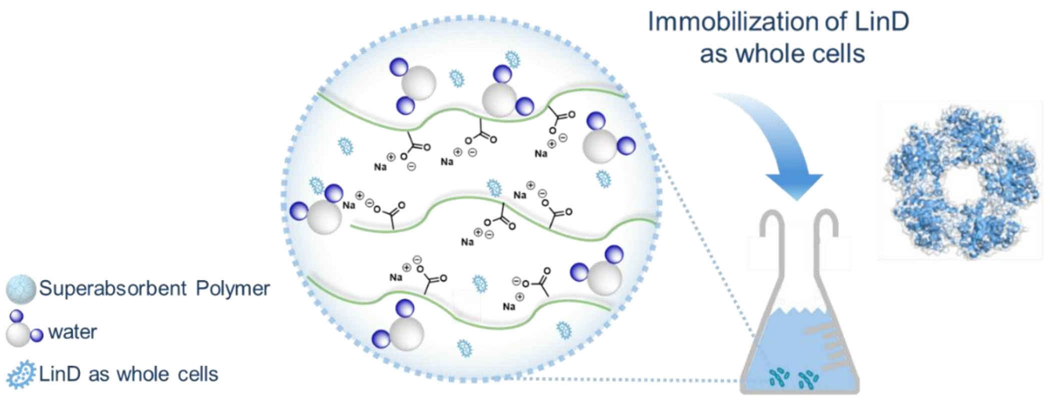

B General conditions for the dehydration of *rac*-**1a** using whole-cell LinD in different preparations

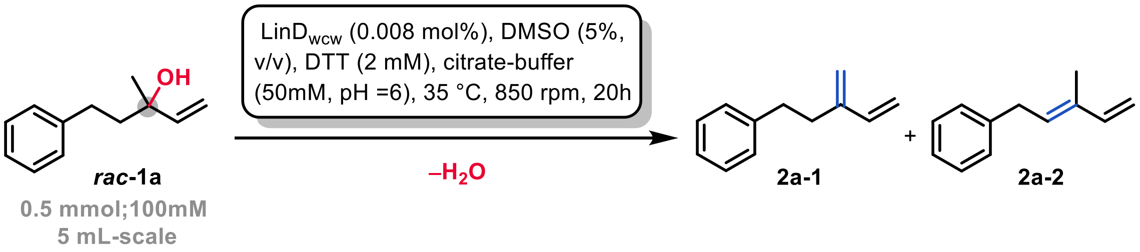

106 mg LinD$_{wcw}$ × 2.77 mU/mg ≙ 293 mU ≙ 0.008 mol%

C Performance evaluation of whole-cell LinD in free, lyophilized, and immobilized forms

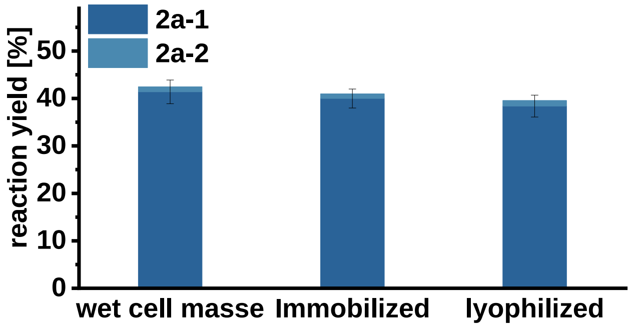

Figure S6. Biocatalytic dehydration of *rac*-**1a** (100 mM) comparing free, lyophilized, and immobilized preparations of whole-cell LinD.

3.7 Figure S7

As demonstrated in Figure 7A, chemocatalytic dehydration, typically catalyzed by strong acids such as sulfuric acid, offers rapid reactions but suffers from low selectivity, harsh conditions, and challenging separations. In contrast, biocatalytic dehydration using enzymes like LinD provides superior selectivity and operates under milder, aqueous conditions, making it a more sustainable approach. However, a thorough understanding of enzyme properties is crucial for optimizing the process. A general comparison of both approaches is presented below:

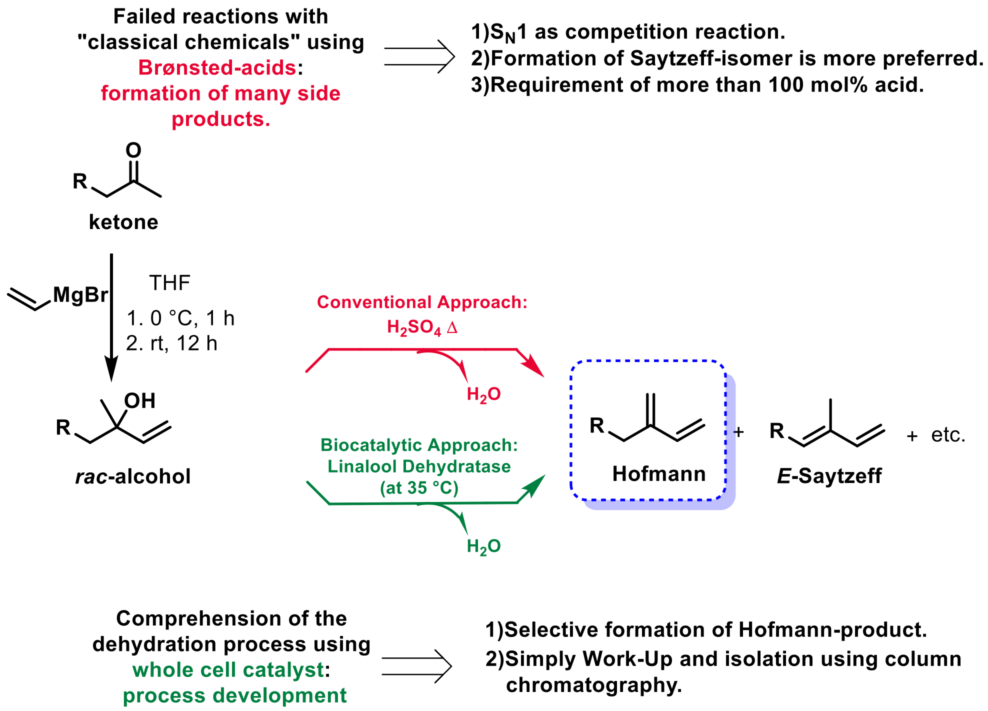

Figure. S7. Comparison of chemo- vs. biocatalytic dehydration: Advantages and Disadvantages.

3.8 Figure S8

In addition to α-methyl-substituted allylic alcohols, we also evaluated structurally distinct analogues, including secondary alcohols and tertiary alcohols that deviate from the typical methyl- and vinyl-substituted motifs, as shown below. No or only minimal product formation was observed in these cases, underscoring the high structural specificity of LinD and providing a clearer picture of its substrate limitations.

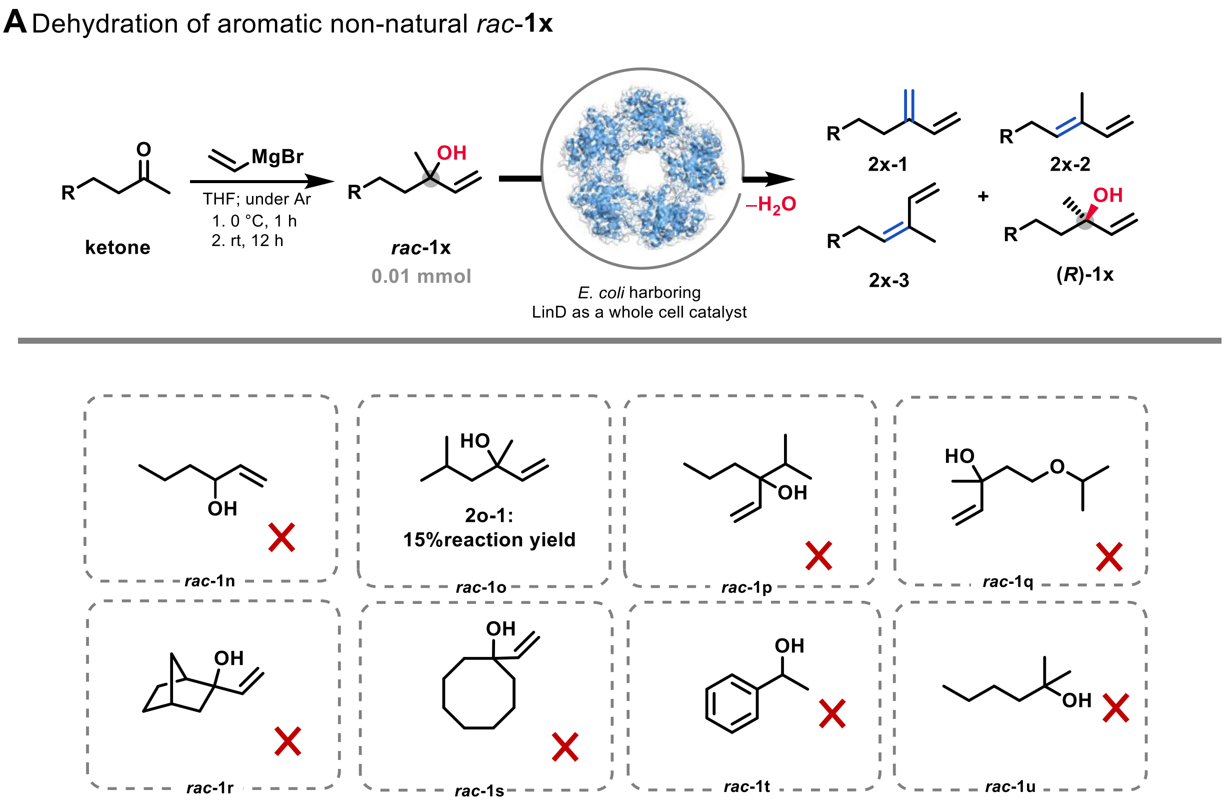

Figure S8. List of substrates that were not converted or were only weakly converted by whole-cell LinD. Reaction conditions: *E. coli* whole cells (0.2 mol%, 60 mg wcw mL^{-1}≙2.77 mU/mg_{wcw}), 10 mM *rac*-alcohol, 2 mM DTT and 5% (v/v) DMSO in citrate-buffer (50 mM, pH=6), 20 h, 35°C and 850 rpm on a 1mL-scale.

3.9 Figure. S9

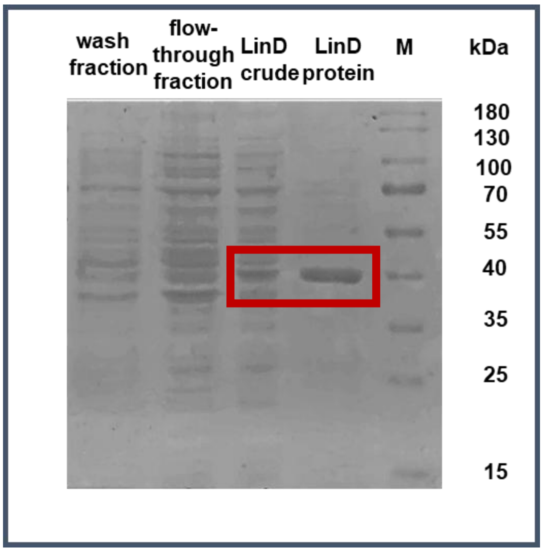

Figure. S9. SDS-Analysis of the purified LinD. Marker (M) was the PageRuler Plus prestained Protein Ladder from THERMO SCIENTIFIC™.

4. CALIBRATION CURVES AND CHROMATOGRAMS

4.1 Calibration Curve for GC Quantification of

Rac-linalool (*rac*-**1b**) and β-myrcene (**2b-1**) were analyzed by GC based on calibration curves for each compound.

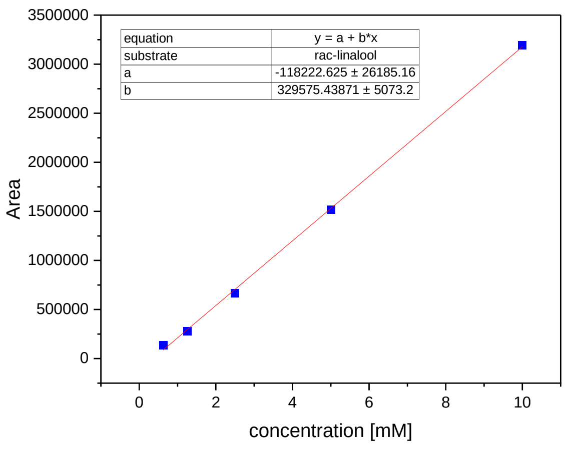

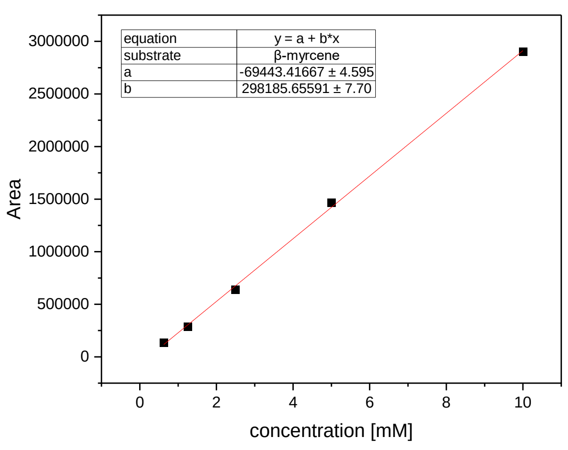

4.2 GC-Chromatogram

The activity test of LinD using *rac*-alcohols was analyzed on a SHIMADZU GC-2030 with FID-detector using Phenomenex ZB-5MSi (30 m x 250 µm x 0.25 µm) as column with N_2 as a carrier gas in constant flow of 1.03 mL/min., 10:1 split ration, 1µL injection volume.

The oven program for the analysis: starting at the oven program for the analysis: starting at 100°C with 1.50 min hold time and then with an immediate gradient of 40°C/min to155°C and hold 1.50 min, subsequently to a 40°C/min gradient to 210°C and hold 2min. The retention time t_R of *rac*-**1b** was observed at 3.33 min, while the retention time t_R of **2b-1** was recorded at 2.79 min.

A Analytical results from the LinD-catalyzed dehydration of *rac*-Linalool (*rac*-**1b**) to β-myrcene (**2b-1**)

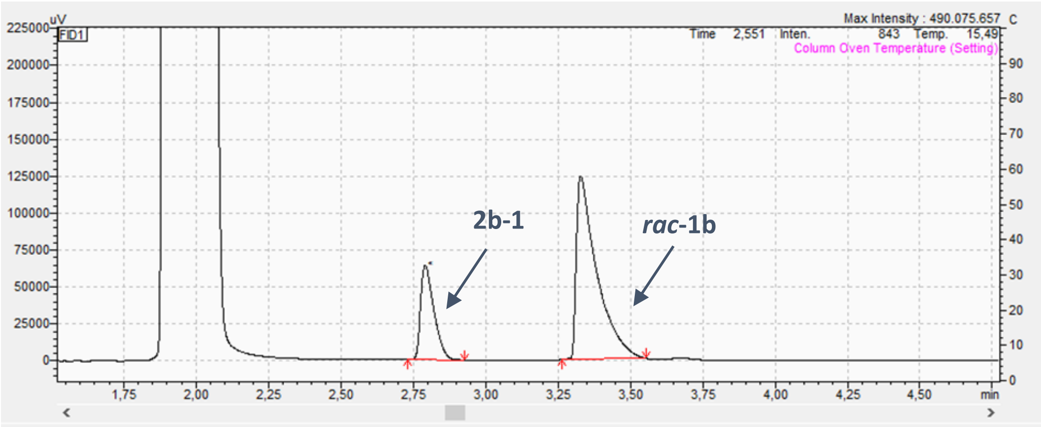

Peak Table | Compound | Group | Calibration Curve

Peak#	Ret. Time	Area	Height	Mark	ID#	Name	Area%
1	2,792	204145	63782	M	2	myrcene	25,060
2	3,330	610480	123800	M	1	linalool	74,940
Total		814625	187581				100,000

4.3 Chiral-GC-Chromatogram

The chiral GC-analysis of *rac*-linalool was done using on a SHIMADZU GC-2030 with FID-detector on a BGB-174 (25 m x 250 µm x 0.25 µm) chiral column from the BGB Analytik AG company with N_2 as a carrier gas in constant flow of 2.00 mL/min., 50:1 split ration, 1µL injection volume. The oven program for the analysis: starting at 60°C with 1.50 min hold time and then with an immediate gradient of 10°C/min to 70°C and hold 40 min, subsequently to a 30°C/min gradient to 170°C and hold 2min. The reference spectrum of commercially sourced *rac*-**1b**, with an enantiomeric ratio of 50:50, showed retention times of t_R((*R*)-**1b**) = 35.09 min and t_R((*S*)-**1b**) = 36.71 min.

A Reference: *rac*-Linalool (*rac*-**1b**)

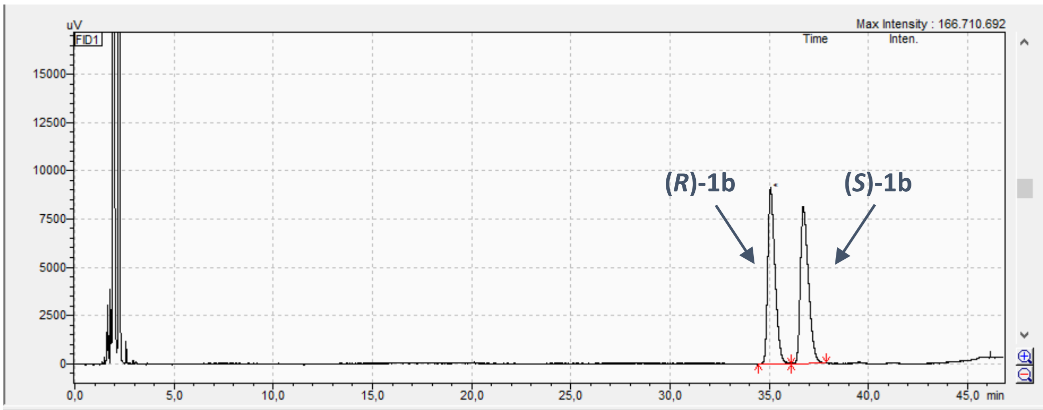

Results View - Peak Table

Peak Table | Compound | Group | Calibration Curve

Peak#	Ret. Time	Area	Height	Mark	Conc.	Unit	ID	N	Area%
1	35,091	230083	9154	M	0,000				50,105
2	36,708	229122	8129	V M	0,000				49,895
Total		459205	17283		0,000				100,000

B Example: Dehydration of *rac*-Linalool (*rac*-**1b**) using whole-cell LinD (0.8 mol%) for 3h

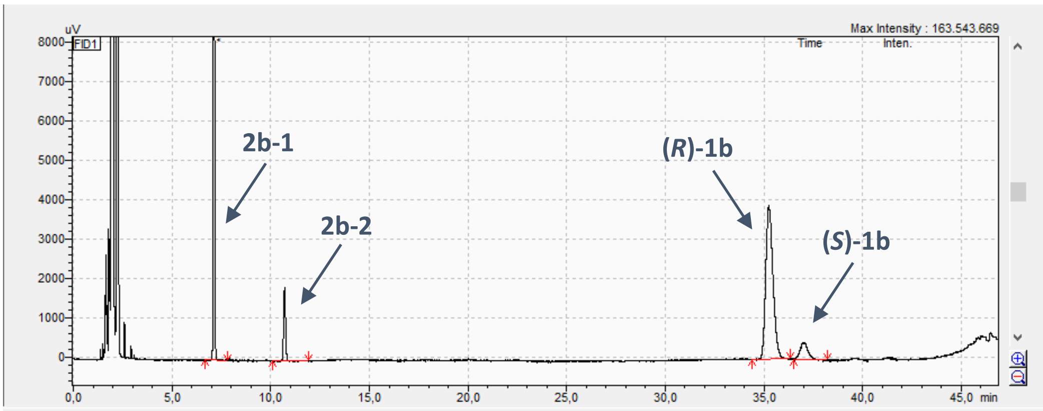

Results View - Peak Table

Peak Table | Compound | Group | Calibration Curve

Peak#	Ret. Time	Area	Height	Mark	Conc.	Unit	ID	N	Area%
1	7,126	64655	14527	M	0,000				34,331
2	10,708	12682	1854	M	0,000				6,734
3	35,245	99393	3897	M	0,000				52,776
4	37,023	11599	435	M	0,000				6,159
Total		188330	20714		0,000				100,000

4.4 GCMS-Chromatogram

The GCMS-analysis was performed on a SHIMADZU single quadrupole GCMS-QP2020 NX gas chromatograph-mass spectormeter using Phenomenex ZB-5MSi (30 m x 250 µm x 0.25 µm) as column with H_2 as a carrier gas in constant flow of 1.03 mL/min., 10:1 split ration, 1µL injection volume. The oven program for the analysis: starting at the oven program for the analysis: starting at 100°C with 1.50 min hold time and then with an immediate gradient of 40°C/min to155°C and hold 1.50 min, subsequently to a 40°C/min gradient to 210°C and hold 2min.

A Example: Dehydration of 10 mM (*R*)-**1b** (purity 95%) using LinD

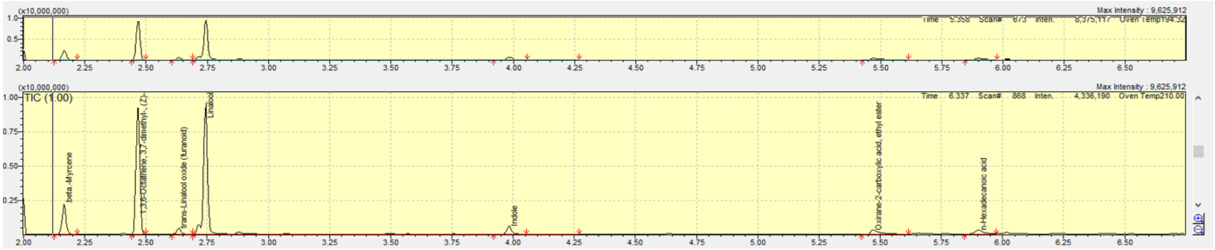

Peak#	Ret.Time	Start Tm	End Tm	m/z	Area	Area%	Height	Height%	A/H	Mark	Name	CAS #	SI
1	2.165	2.125	2.220	TIC	2483948	9.30	2185023	9.64	1.14	V	.beta.-Myrcene	123-35-3	96
2	2.468	2.440	2.500	TIC	10312521	38.59	9199973	40.61	1.12	V	1,3,6-Octatriene, 3,7-dimethyl-, (Z)-	3338-55-4	95
3	2.635	2.605	2.690	TIC	514468	1.93	447001	1.97	1.15	V	trans-Linalool oxide (furanoid)	34995-77-2	96
4	2.744	2.690	4.270	TIC	10983300	41.10	9591211	42.34	1.15	SV	Linalool	78-70-6	97
5	3.984	3.920	4.055	TIC	814948	3.05	587609	2.59	1.39	TV	Indole	120-72-9	98
6	5.472	5.425	5.615	TIC	823132	3.08	324638	1.43	2.54	S	Oxirane-2-carboxylic acid, ethyl ester	0-0-0	69
7	5.901	5.845	5.975	TIC	788027	2.95	319741	1.41	2.46	V	n-Hexadecanoic acid	57-10-3	94

4.5 HPLC-chromatograms

HPLC analysis was performed with column IC-3 from *Daicel* (4.6 mm ID x 250 mm): n-hexane/2-propanol 98:2 flow rate 1 mL/min, λ = 220 nm, Rt_{olefin} = 3.49 min Rt_S = 11.45 min, Rt_R = 12.21 min). Representative chromatograms for the dehydration of rac-1a (500 mM) on a 5 mL scale are shown below.

A Reference: *rac*-**1a**

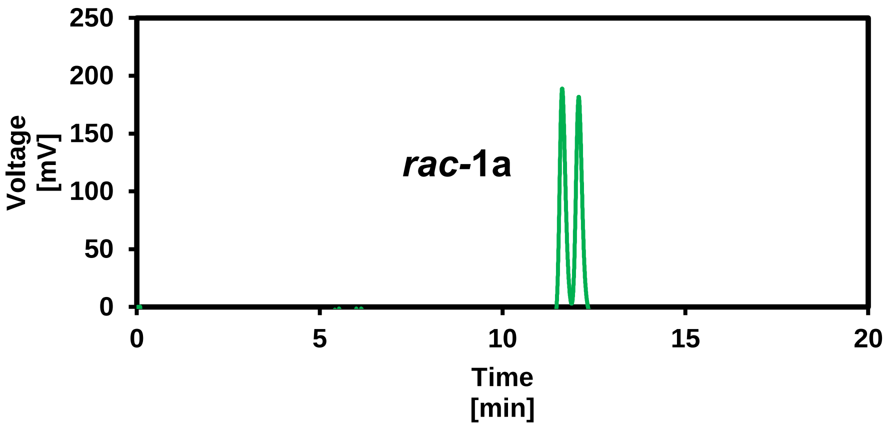

B Dehydration of *rac*-**1a** to **2a-1**

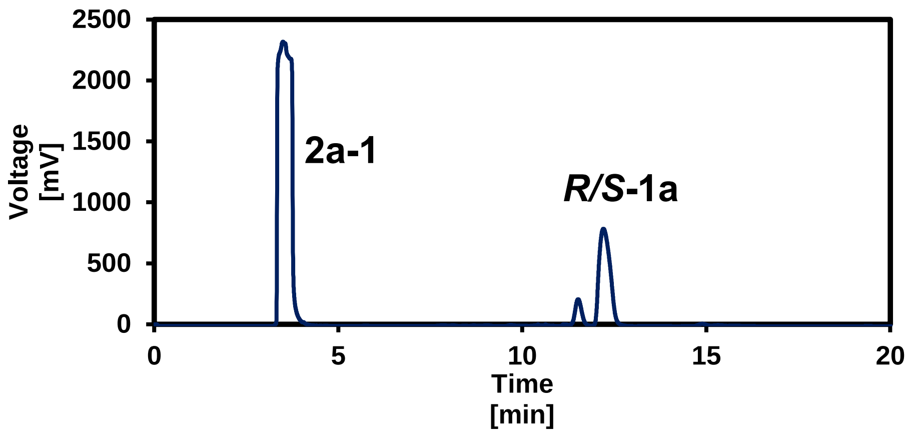

C Reference: **2a-1**

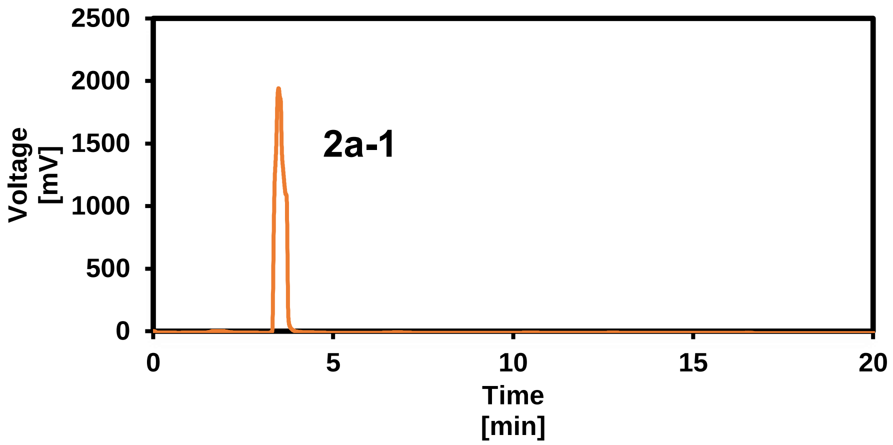

D Reference: (*R*)-**1a**

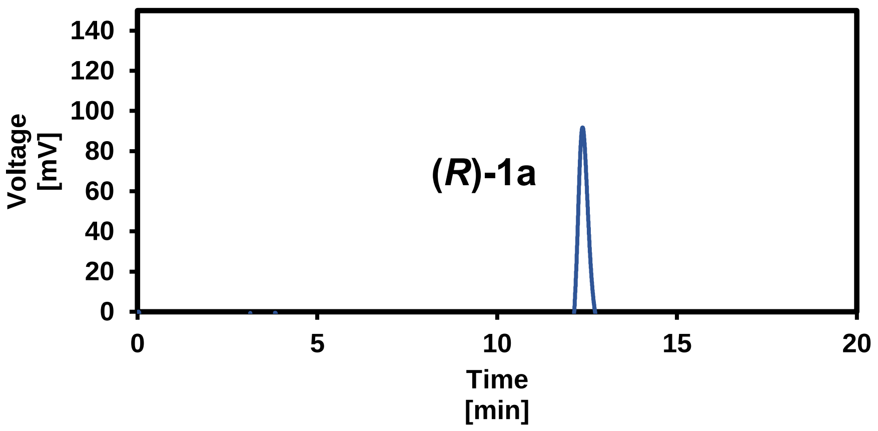

E Isolated *R*/S-**1a** mixture

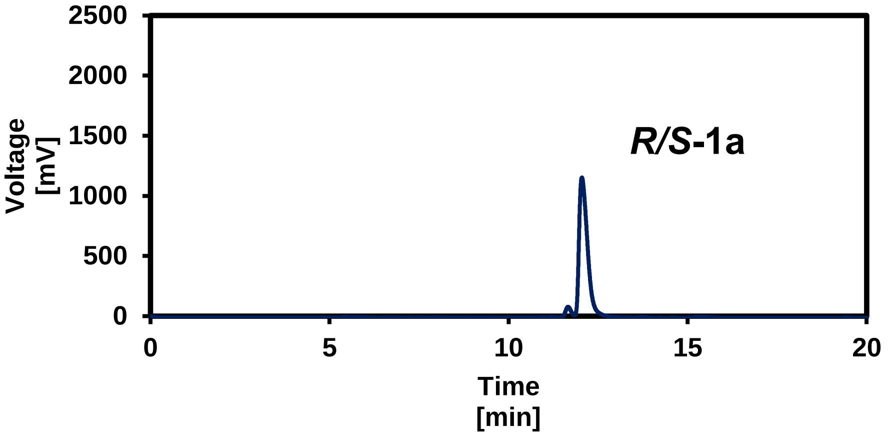

5. SYNTHETIC PROCEDURES

5.1 Synthesis of 3-methyl-5-phenylpent-1-en-3-ol (*rac*-1a)

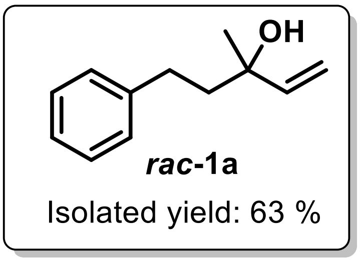

The synthesis was carried out according to GP-F. Vinylmagnesium bromide in THF (20 mmol) and 4-phenyl-2-butanone (19 mmol) were applied for the reaction. Work up and purification yielded the compound (***rac*-1a**, 2.11 g, 12 mmol, 63 %) as yellow liquid. HPLC analysis was performed with column IC-3 from *Daicel* (4.6 mm ID x 250 mm): n-hexane/2-propanol 98:2 flow rate 1 mL/min, λ = 220 nm, Rt_1 = 11.45 min, Rt_2 = 12.21 min).

^{1}H-NMR (500 MHz. $CDCl_3$): δ [ppm] = 7.38-7.03 (m, 5H, -**H**^{aromatic}, 6.05 (dd, 1 H, - C**H**), 5.30 (dd, 1 H, -CHC**H**$_2$), 5.21 (m, 1 H, -CHC**H**$_2$), 5.04 (m, 1 H, -C**H**$_2$), 2.73-2.60 (m, 2H, - C**H**$_2$CH$_2$), 1.90-1.80 (m, 2H, CH$_2$C**H**$_2$), 1.35 (s, 3H, -CC**H**$_3$).

5.2 Synthesis of (3-metyhlenepent-4-en-1-yl)bezene (2a-1)

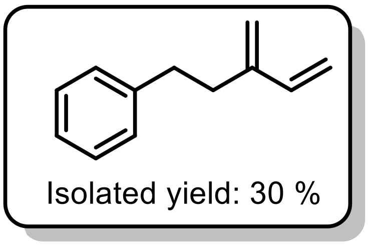

The synthesis was carried out according to the protocol for kinetic resolution according to GP-C. Whole-cell LinD (0.004 mol%) and 3-methyl-5-penylpent-1-en-3-ol (*rac*-**1a**, 500 mM, 2.5 mmol) were performed on a 5 mL reaction scale. The reaction was carried out in the presence of DMSO (5%, v/v) and DTT (2 mM) at 35 °C and 850 rpm for 20 h.

Work up was carried out with ethyl acetate and purification yielded the compound (cyclohexane: DCM=9:1, **2a-1**, 118 mg, 0.75 mmol, 47% reaction yield, 30% isolated yield) as a colorless liquid. HPLC analysis was performed with column IC-3 from *Daicel* (4.6 mm ID x 250 mm): n-hexane/2-propanol 98:2 flow rate 1 mL/min, λ = 220 nm, Rt_1 = 3.49 min).

^{1}H NMR (500 MHz, $CDCl_3$) δ 7.30 (t, *J* = 7.5 Hz, 2H, $\mathbf{H}^{ortho}$-$C^{aromatic}$), 7.24 – 7.19 (m, 3H, $\mathbf{H}^{meta/para}$-$C^{aromatic}$), 6.41 (dd, *J* = 17.6, 10.8 Hz, 1H, C**H**-CH_2), 5.28 (dd, *J* = 17.6 Hz, 1H, CH-C$\mathbf{H}_2$), 5.12 (dd, *J* = 10.8 Hz, 1H, CH-C$\mathbf{H}_2$), 5.07 – 4.97 (m, 2H, C-C$\mathbf{H}_2$), 2.81(m, 2H, $C^{aromatic}$-C$\mathbf{H}_2$), 2.53 (m, *J* = 9.8, 6.5 Hz, 2H, $C^{aromatic}$-CH_2-C$\mathbf{H}_2$).

5.3 Synthesis of (*E*)-(3-methylpenta-2,4-dien-1-yl)benzene (2a-2)

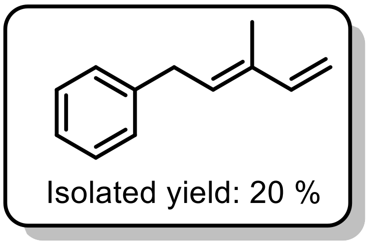

The synthesis was carried out according to the protocol for kinetic resolution according to GP-C. Whole-cell LinD (0.2 mol%) and (*R*)-3-methyl-5-penylpent-1-en-3-ol ((*R*)-**1a**, 10 mM,

2.5 mmol) were performed on a 250 mL reaction scale. The biosynthesis was carried out in the presence of DMSO (5%, v/v) and DTT (2 mM) at 35 °C and 850 rpm for 20 h.

Workup was performed with ethyl acetate, and purification by column chromatography (cyclohexane/DCM = 9:1) yielded **2a-2** as a colorless liquid (80 mg, 0.51 mmol; 33% reaction yield, 20% isolated yield).HPLC analysis was performed with column IC-3 from *Daicel* (4.6 mm ID x 250 mm): n-hexane/2-propanol 98:2 flow rate 1 mL/min, λ = 220 nm, Rt_1 = 3.50 min).

^{1}H NMR (500 MHz, $CDCl_3$): δ 7.29 (m, *J* = 8.3 Hz, 2H, H^{ortho}-$C^{aromatic}$), 7.19 (m, *J* = 8.7 Hz, 3H, $H^{meta/para}$-$C^{aromatic}$), 6.40 (dd, *J* = 17.4 Hz,1H, C**H**-C), 5.68 (t, *J* = 7.6 Hz, 1H, CH_2-C**H**), 5.18 (dd, *J* = 17.4 Hz, 1H, C**H**$_2$-CH), 4.98 (dd, *J* = 10.7 Hz, 1H, C**H**$_2$-CH), 3.50 (d, 2H, C**H**$_2$-Ph), 1.86 (s, 3H, C**H**$_3$).

5.4 Synthesis of 3-methyl-5-(4-(trifluoromethyl)phenyl)-pent-1-en-3-ol (*rac*-1c)

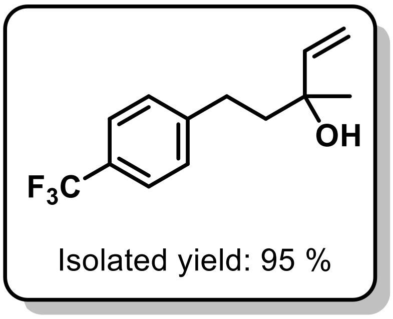

The synthesis was carried out according to GP F. Vinylmagnesium bromide in THF (15 mmol) and 4-(4-Trifluoromethyl)phenyl)butan-2-one (10 mmol) were utilized for the reaction. Work up and purification yielded the compound (***rac*-1c**, 2.33 g, 9.54 mmol, 95% yield) as a colorless liquid. HPLC analysis was performed with column OD-H from *Daicel* (4.6 mm ID x 250 mm): n-hexane/2-propanol 99.5:0.5, flow rate 1.0 mL/min, λ = 210 nm, Rt_1 = 27.9 min, Rt_2 = 23.6 min).

^{1}H NMR (500 MHz, $CDCl_3$) δ 7.52 (d, *J* = 7.9 Hz, 2H, **H**^{aromatic}), 7.29 (d, *J* = 8.0 Hz, 2H, **H**^{aromatic}), 5.97 (dd, *J* = 17.3, 10.8 Hz, 1H, OH-C-C**H**-CH_2), 5.28 (dd, *J* = 17.3 Hz, 1H, OH-C-CH-C**H**$_2$), 5.13 (dd, *J* = 10.8 Hz, 1H, OH-C-CH-C**H**$_2$), 2.73 (m, *J* = 12.7, 5.9 Hz, 2H, $C^{aromatic}$-C**H**$_2$), 1.98 – 1.73 (m, 2H, $C^{aromatic}$-CH_2-**CH**$_2$), 1.36 (s, *J* = 1.2 Hz, 3H, OH-C-C**H**$_3$).

5.5 Synthesis of (5-(4-fluorophenyl)-3-methylpent-1-en-3-ol (*rac*-1d)

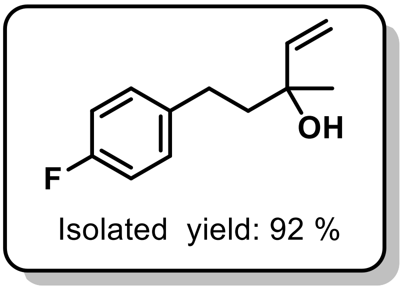

The synthesis was conducted in analogy to GP F. Grignard reagent (10 mmol) and 4-(4-Fluorophenyl)butan-2-one (10 mmol) were applied for the reaction. Work up and purification yielded the compound (***rac*-1d**, 1.79 g, 9.22 mmol, 92% yield) as a colorless liquid. HPLC analysis was performed with column OD-H from *Daicel* (4.6 mm ID x 250 mm): n-hexane/2-propanol 99.5:0.5, flow rate 1.0 mL/min, λ = 210 nm, Rt_1 = 25.5 min, Rt_2 = 29.6 min).

^{1}H NMR (500 MHz, $CDCl_3$) ^{1}H NMR (500 MHz, $CDCl_3$) δ 7.13 (d, *J* = 8.3, 5.5 Hz, 2H, **H**^{aromatic}), 6.95 (d, *J* = 8.8, 2.0 Hz, 2H, **H**^{aromatic}), 5.96 (dd, *J* = 17.4, 10.7 Hz, 1H, OH-C-C**H**-CH_2), 5.41 – 5.19 (dd, 1H, OH-C-CH-C**H$_2$**), 5.12 (dd, *J* = 10.8 Hz, 1H, OH-C-CH-C**H$_2$**), 2.71 – 2.46 (m, 2H, C^{aromatic}-C**H$_2$**), 1.81 (m, *J* = 22.6, 17.5 Hz, 3H, C^{aromatic}-CH_2-**CH$_2$**), 1.34 (s, *J* = 1.2 Hz, 3H, OH-C-C**H$_3$**).

5.6 Synthesis of (5-(4-chlorophenyl)-3-methylpent-1-en-3-ol (*rac*-1e)

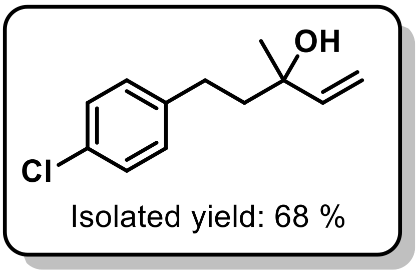

The synthesis was carried out according to GP F. Grignard reagent (5.2 mmol) and 4-(4-clorophenyl) butan-2-one (5 mmol) were applied for the reaction. Work up and purification yielded the compound (***rac*-1e**, 711 mg, 3.38 mmol, 68% yield) as a yellow liquid.

HPLC analysis was performed with column OD-H from *Daicel* (4.6 mm ID x 250 mm): n-hexane/2-propanol 99.5:0.5, flow rate 1.0 mL/min, λ = 210 nm, Rt_1 = 27.7 min, Rt_2 = 32.6 min).

^{1}H NMR (500 MHz, $CDCl_3$) δ 7.34 – 7.19 (m, 2H, -**H**^{aromatic}), 7.15 – 7.02 (m, 2H, -**H**^{aromatic}), 5.96 (dd, *J* = 17.3, 1H, -C**H**CH_2), 5.26 (dd, *J* = 17.4,1H, -CHC**H**$_2$), 5.12 (dd, *J* = 10.8, 1H, CHC**H**$_2$), 2.74 – 2.49 (m, 3H, - C**H**$_2$$CH_2$), 1.92 – 1.65 (m, 2H, CH_2C**H**$_2$), 1.34 (s, 3H, -CC**H**$_3$).

5.7 Synthesis of (5-(4-Bromorophenyl)-3-methylpent-1-en-3-ol (*rac*-1f)

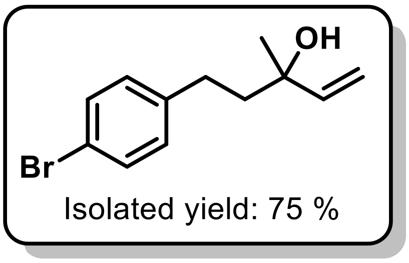

The synthesis was prepared in analogy to GP F. Grignard reagent (4.2 mmol) and 4-(4-bromophenyl)butan-2-one (4 mmol) were applied for the reaction. Work up and purification yielded the compound (***rac*-1f**, 778 mg, 3.05 mmol, 75% yield) as a yellow liquid. HPLC analysis was performed with column OD-H from *Daicel* (4.6 mm ID x 250 mm): n-hexane/2-propanol 99.5:0.5, flow rate 1.0 mL/min, λ = 210 nm, Rt_1 = 30.7 min, Rt_2 = 37.2 min).

^{1}H NMR (500 MHz, $CDCl_3$) δ 7.51 – 7.31 (m, 2H, -**H**^{aromatic}), 7.05 (m, *J* = 7.8, 2H, -**H**^{aromatic}), 5.96 (dd, *J* = 17.3, 1H, -C**H**CH_2), 5.26 (dd, *J* = 17.4,1H, -CHC**H**$_2$), 5.12 (dd, *J* = 10.7, 1H, CHC**H**$_2$), 2.67 – 2.48 (m, 2H, - C**H**$_2$$CH_2$), 1.92 – 1.68 (m, 2H, CH_2C**H**$_2$), 1.34 (s, 3H, -CC**H**$_3$).

5.8 Synthesis of 3-methyl-5-(p-tolyl)pent-1-en-3-ol (*rac*-1g)

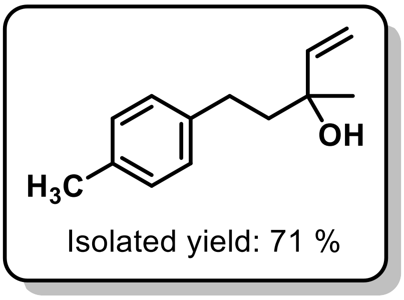

The synthesis was carried out according to GP F. Vinylmagnesium bromide in THF (15 mmol) and 4-(4-methylphenyl)butan-2-ol (15 mmol) were performed for the reaction. Work up and purification yielded the compound (***rac*-1g**, 2 g, 10.59 mmol, 71% yield) as a colorless liquid. HPLC analysis was performed with column IC-3 from *Daicel* (4.6 mm ID x 250 mm): n-hexane/2-propanol 98:2 flow rate 1 mL/min, λ = 220 nm, Rt_1 = 12.61 min, Rt_2 = 14.55 min).

^{1}H NMR (500 MHz, $CDCl_3$) δ 7.09 (m, 4H, **H**^{aromatic}), 5.97 (dd, *J* = 17.4, 10.8 Hz, 1H, OH-C-C**H**-CH_2), 5.27 (dd, *J* = 17.3 Hz, 1H, OH-C-CH-C**H_2**), 5.11 (dd, *J* = 10.6 Hz, 2H, OH-C-CH-C**H_2**), 2.61 (m, *J* = 13.4, 6.6 Hz, 2H, C^{aromatic}-C**H_2**), 2.32 (s, 3H, C^{aromatic}-C**H_3**), 1.83 (m, *J* = 24.6, 2H, C^{aromatic}-CH_2-**CH_2**), 1.34 (s, 3H, OH-C-C**H_3**).

5.9 Synthesis of 5-(4-methoxyphenyl)-3-methylpent-1-en-3-ol (*rac*-1h)

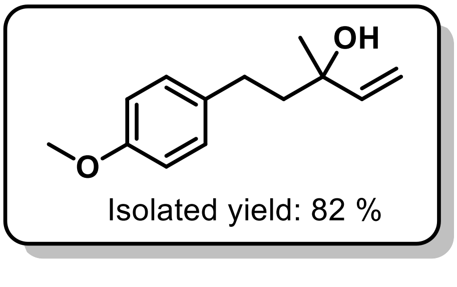

The synthesis was conducted in analogy to GP F. Grignard reagent (35 mmol) and 4-(4-methoxyphenyl)butan-2-one (35 mmol) were applied for the reaction. Work up and purification yielded the compound (***rac*-1h**, 5.91 g, 28.67 mmol, 82% yield) as a yellow liquid. HPLC analysis was performed with column IC-3 from *Daicel* (4.6 mm ID x 250 mm): n-hexane/2-propanol 98:2 flow rate 1 mL/min, λ = 220 nm, Rt_1 = 22.4 min, Rt_2 = 24.3 min).

^{1}**H-NMR** (500 MHz, $CDCl_3$, 25°C) *δ* (ppm) = 7.11 (m, 2H, -**H**^{aromatic}), 6.83 (m, 2H, -**H**^{aromatic}), 5.99 (m, 1H, **H**C(CH_2)), 5.30 (m, 1H, HC(C**H**$_2$)), 5.11 (m, 1H, HC(C**H**$_2$), 3.78 (s, 3H, OC**H**$_3$), 2.84 (m,1 H, O**H**) 2.66 (m, 2H, Ar(C**H**$_2$)CH_2), 1.83 (m, 1H, Ar(CH_2)C**H**$_2$), 1.34 (s, 3H, C**H**$_3$).

5.10 Synthesis of 4-(3-Hydroxy-3-methylpent-4-en-1-yl)phenol (*rac*-1i)

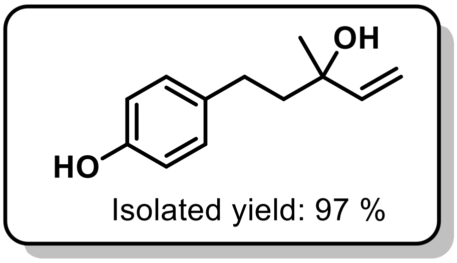

The synthesis was performed according to GP F. Grignard reagent (70 mmol) and 4-hydroxy-phenyl)butan-2-on (31 mmol) were applied for the reaction. Work up and purification yielded the compound (***rac*-1i**, 5,78 g; 30.07 mmol; 97 % yield) as a yellow solid. . HPLC analysis was performed with column OJ-H from *Daicel* (4.6 mm ID x 250 mm): n-hexane/2-propanol 90:10 flow rate 1 mL/min, λ = 230 nm, Rt_1 = 24.2 min, Rt_2 = 26.0 min).

^{1}**H-NMR** (500 MHz, $CDCl_3$, 25°C) *δ* (ppm) = 6.99 (m, 2H, -**H**^{aromatic}), 6.74 (m, 2H, -**H**^{aromatic}), 5.96 (m, *J* = 17.3, 10.8 Hz, 1H, **H**C(CH_2), 5.26 (dd, *J* = 17.4, 1.2 Hz, 1H, HC(C**H**$_2$)), 5.11 (dd, *J* = 10.8, 1.2 Hz, 1H, C(13)H_2), 4.13 (m, 1H, **H**OAr), 2.58 (tt, *J* = 11.4, 7.2 Hz, 2H, Ar(C**H**$_2$)CH_2), 1.82 (m, 2H, Ar(CH_2)C**H**$_2$), 1.34 (s, 2H, C**H**$_3$).

5.11 Synthesis of 2-phenylbut-3-en-2-ol (*rac*-1j)

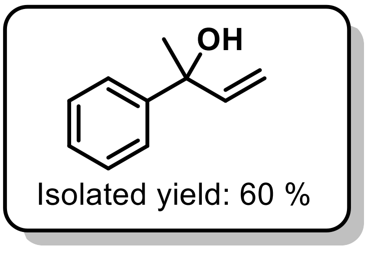

The synthesis was carried out according to GP F. Vinylmagnesium bromide in THF (20 mmol) and acetophenone (18 mmol) were performed for the reaction. Work up and purification yielded the compound (***rac*-1j**, 1.614 g, 10.88 mmol, 60%) as a colorless liquid. HPLC analysis was performed with column IC-3 from *Daicel* (4.6 mm ID x 250 mm): n-hexane/2-propanol 98:2 flow rate 1 mL/min, λ = 220 nm, Rt_1 = 8.1 min, Rt_2 = 9.0 min).

^{1}H-NMR (500 MHz. $CDCl_3$): δ [ppm] = 7.48 (d, 2H, -**H**^{aromatic}), 7.35 (t, 2H, -**H**^{aromatic}), 7.26 (t, 1H, -**H**^{aromatic}), 6.18 (dd, 1 H, - C**H**), 5.30 (dd, 1 H, CHC**H**$_2$), 5.15 (dd, 1 H, CHC**H**$_2$), 1.66 (s, 3H, -CC**H**$_3$).

5.12 Synthesis of 2-cyclohexylbut-3-en-2ol (*rac*-1k)

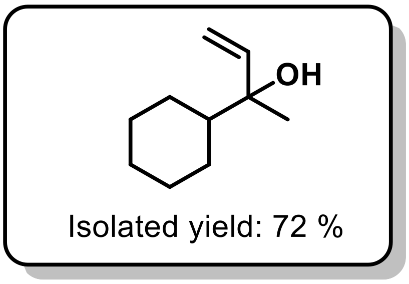

The synthesis was carried out according to GP F. Vinylmagnesium bromide in THF (12.5 mmol) and cyclohexylmethylketon (10 mmol) were utilized for the reaction. Work up and purification yielded the compound (***rac*-1k**, 1113 mg, 7.216 mmol, 72% yield) as a colorless liquid.

^{1}H NMR (500 MHz, $CDCl_3$) δ 5.91 (dd, *J* = 17.4, 10.8 Hz, 1H, OH-C-C**H**-CH$_2$), 5.18 (dd, *J* = 17.4, 1.4 Hz, 1H, OH-C-CH-C**H**$_2$), 5.06 (dd, *J* = 10.8, 1.4 Hz, 1H, OH-C-CH-C**H**$_2$), 1.78 (m, *J* = 13.3, 6.9 Hz, 6H, C^{cyc}-C**H$_2$**), 1.68 – 1.63 (m, 1H, C^{para}-C**H**$_2$), 1.38 (s, 1H, O**H**), 1.33 (m, *J* = 12.1, 2.9 Hz, 1H, C**H**-C-OH), 1.23 (s, 3H, C**H$_3$**), 1.11 (m, *J* = 12.7, 10.2 Hz, 1H, C^{para}-C**H**$_2$), 1.03 – 0.92 (m, 2H, C^{ortho}-C**H**$_2$).

5.13 Synthesis of 5-cyclohexyl-3-methylpent-1-en-3ol (*rac*-1l)

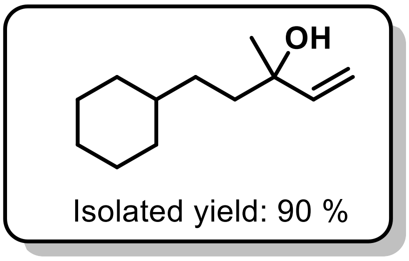

The synthesis was carried out according to GP F. Vinylmagnesium bromide in THF (10 mmol) and 4-cyclohexylbutan-2-on (6.8 mmol) were performed for the reaction. Work up and purification yielded the compound (***rac*-1l**, 1110 mg, 6.09 mmol, 90% yield) as a yellow liquid. The chiral GC-analysis of the generated alcohol ***rac*-1l** was performed using an Agilent CP-Chirasil-Dex CB column 30 m x 250µm x 0.25 µm) and helium as the carrier gas in constant flow of 1.2 mL/min., 15:1 split ration, 1µL injection volume. The oven program for the analysis: starting at 30°C with an immediate gradient of 5°C/min to 100°C and hold 5 min, then to a 0.1°C/min gradient to 103°C and hold 1 min and then to a 30°C/min gradient to 180°C and hold 2 min. Retention times of *R*/*S* enantiomers in minutes: Rt_1 = 42.1 min; Rt_2 = 42.8 min.

^{1}H NMR (500 MHz, $CDCl_3$) δ 5.93 (dd, *J* = 17.3, 1H, -C**H**CH_2), 5.20 (dd, *J* = 17.3, 1H, -CHC**H**$_2$), 5.02 (dd, *J* = 10.8,1H, -CHC**H**$_2$), 1.78 – 1.72 (m, 2H, -**H**^{cyclic}), 1.64 (m, 2H, -**H**^{cyclic}), 1.58 (m, 2H, -**H**^{cyclic}), 1.48 – 1.38 (m, 4H, -**H**^{cyclic}), 1.36 (s, 1H, -**H**^{cyclic}), 1.27 (s, 3H, -CC**H**$_3$), 1.22 (m, *J* = 12.3, 1H, C**H**$_2$$CCH_3$), 1.17 – 1.07 (m, 1H, C**H**$_2$$CCH_3$), 1.00 – 0.89 (m, 2H, - C**H**$_2$$CH_2$).

5.14 Synthesis of 1-cyclopentyl-2-methylbut-3-en-2-ol (*rac*-1m)

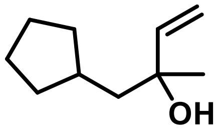

The synthesis was conducted in analogy to GP F. Grignard reagent (12.5 mmol) and 1-cyclopentyl-propan-2-on (10 mmol) were applied for the reaction. Work up and purification yielded the compound (***rac*-1m**, 799 mg, 5.180 mmol, 52% yield) as a colorless liquid. The chiral GC-analysis of the generated alcohol ***rac*-1m** was done using an AGILENT CP-Chirasil-Dex CB column 30 m x 250µm x 0.25 µm) and helium as the carrier gas in constant flow of 1.2 mL/min., 15:1 split ration, 1µL injection volume. The oven program for the analysis: starting at 30°C with an immediate gradient of 5°C/min to 50°C, directly to a 0.1°C/min gradient to 76°C, then to a 30°C/min gradient to 150°C and hold 1 min. Retention times of *R*/*S* enantiomers in minutes: Rt_1 = 250.1 min; Rt_2 = 252.3 min.

^{1}H NMR (500 MHz, $CDCl_3$) δ 5.94 (dd, *J* = 17.3, 10.7 Hz, 1H, OH-C-C**H**-CH_2), 5.21 (dd, *J* = 17.3, 1H, OH-C-CH-C**H_2**), 5.02 (dd, *J* = 10.7, 1H, OH-C-CH-C**H_2**), 1.85 – 1.76 (m, 2H, H-C^{cyc}-C^{cyc}-C**H_2**), 1.64 – 1.60 (d, 2H, CH-C**H_2**), 1.59 – 1.55 (m, 2H, H-C^{cyc}-C^{cyc}-**H_2**), 1.52 – 1.45 (m, 2H, H-C^{cyc}-C^{cyc}-C^{cyc}-**H_2**), 1.40 (s, 1H, O**H**), 1.28 (s, 3H, C**H_3**), 1.17 – 1.01 (m, 2H, H-C^{cyc}-C^{cyc}-**H_2**).

5.15 Synthesis of 3-methylhex-1-en-3-ol (*rac*-C_1)

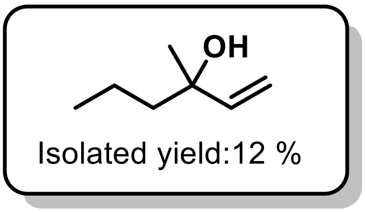

The synthesis was carried out according to GP F. Vinylmagnesium bromide in THF (20 mmol) and 2-pentanone (17 mmol) were applied for the reaction. Work up and purification yielded the compound (***rac*-C₁**, 228 mg, 2 mmol, 12%) as colorless liquid. The chiral GC-analysis of the generated alcohol ***rac*-C_1** was done using on a SHIMADZU GC-2030 with FID-detector on a BGB-174 (25 m x 250 µm x 0.25 µm) chiral column from the BGB Analytik AG company with N_2 as a carrier gas in constant flow of 2.23 mL/min., 50:1 split ration, 1µL injection volume. The oven program for the analysis: starting at 40°C with 3 min hold time and then with an immediate gradient of 15°C/min to 45°C and hold 10 min, subsequently to a 1°C/min gradient to 50°C and hold 10 min, and then with a gradient of 1°C/min to 55°C and hold 5 min and then to a 45°C/min gradient to 180°C and hold 1 min. Retention times of *R*/*S* enantiomers in minutes: Rt_1 = 18.4 min; Rt_2 = 19.3 min.

^{1}H-NMR (500 MHz. $CDCl_3$): δ [ppm] = 5.90 (dd, 1 H, -C**H**), 5.21 (dd, 1 H, -CHCH_2), 5.04 (dd, 1 H, -CH_2), 1.59-1.25 (m, 4H, CH_2CH_2), 1.30 (s, 3H, -CCH_3), 0.92 (t, 3H, - CH_2CH_3).

5.16 Synthesis of 3-methylhept-1-en-3-ol (*rac*-C_2)

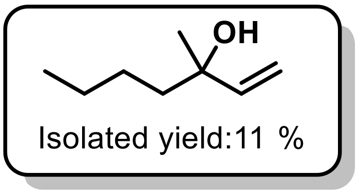

The synthesis conducted in analogy to GP F. Grignard reagent (22 mmol) and 2-hexanone (18 mmol) were applied for the reaction. Work up and purification yielded the compound (***rac*-C_2**, 508 mg, 2 mmol, 11%) as colorless liquid.

The chiral GC-analysis of the generated alcohol ***rac*-C2** was conducted using on a SHIMADZU GC-2030 with FID-detector on a BGB-174 (25 m x 250 µm x 0.25 µm) chiral column from the BGB Analytik AG company with N_2 as a carrier gas in constant flow of 2.23 mL/min., 50:1 split ration, 1µL injection volume. The oven program for the analysis: starting at 40°C with 3 min hold time and then with an immediate gradient of 15°C/min to 45°C and hold 10 min, subsequently to a 1°C/min gradient to 50°C and hold 10 min, and then with a gradient of 1°C/min to 55°C and hold 5 min and then to a 45°C/min gradient to 180°C and hold 1 min. Retention times of *R*/*S* enantiomers in minutes: Rt_1 = 29.7 min; Rt_2 = 30.9 min.

^{1}H-NMR (500 MHz. $CDCl_3$): δ [ppm] = 5.90 (dd, 1 H, -C**H**), 5.19 (dd, 1 H, -CHC**H**$_2$), 5.03 (dd, 1 H, -C**H**$_2$), 1.60-1.45 (m, 4H, C**H**$_2$C**H**$_2$), 1.45-1.23 (m, 6H, -C**H**$_2$C**H**$_2$, -CC**H**$_3$), 0.90 (t, 3H, - CH_2C**H**$_3$).

5.17 Synthesis of 3-methyloct-1-en-3-ol (*rac*-C3)

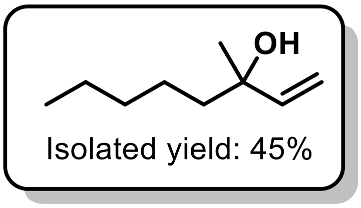

The synthesis was carried out according to GP F. Vinylmagnesium bromide in THF (20 mmol) and 2-heptanone (18 mmol) were applied for the reaction. Work up and purification yielded the compound (***rac*-C3**, 1.16 g, 8.1 mmol, 45%) as colorless liquid. The chiral GC-analysis of the generated alcohol ***rac*-C3** was done using on a SHIMADZU GC-2030 with FID-detector on a BGB-174 (25 m x 250 µm x 0.25 µm) chiral column from the BGB Analytik AG company with N_2 as a carrier gas in constant flow of 2.23 mL/min., 10:1 split ration, 1µL injection volume. The oven program for the analysis: starting at 40°C with 3 min hold time and then with an immediate gradient of 15°C/min to 60°C and hold 10 min, subsequently to a 5°C/min gradient to 75°C and hold 5 min and then to a 45°C/min gradient to 180°C and hold 1 min. Retention times of *R*/*S* enantiomers in minutes: Rt_1 = 16.1. min; Rt_2 = 16.4 min.

^{1}H-NMR (500 MHz. $CDCl_3$): δ [ppm] = 5.90 (dd, 1 H, -C**H**), 5.20 (dd, 1 H, -CHC**H**$_2$), 5.03 (dd, 1 H, -C**H**$_2$), 1.60-1.40 (m, 4H, C**H**$_2$C**H**$_2$), 1.40-1.19 (m, 8H, -C**H**$_2$C**H**$_2$C**H**$_2$, -CC**H**$_3$), 0.95 (t, 3H, - CH_2C**H**$_3$).

5.18 Synthesis of 3-methyltridec-1-en-3-ol (*rac*-C_8)

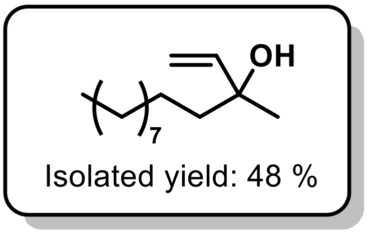

The synthesis was conducted in analogy to GP F. Vinylmagnesium bromide in THF (10 mmol) and 2-dodecanone (8.6 mmol) were applied for the reaction. Work up and purification yielded the compound (*rac*-$\mathbf{C_7}$, 821 mg, 4.1 mmol, 48%) as a colorless liquid. The chiral GC-analysis of the generated alcohol *rac*-$\mathbf{C_7}$ was done using an AGILENT CP-Chirasil-Dex CB column 30 m x 250µm x 0.25 µm) and helium as the carrier gas in constant flow of 1.2 mL/min., 15:1 split ration, 1µL injection volume. The oven program for the analysis: starting at 30°C with an immediate gradient of 5°C/min to 100°C and hold 5 min, then to a 0.1°C/min gradient to 110°C and hold 1 min and then to a 30°C/min gradient to 180°C and hold 2 min. Retention times of *R*/*S* enantiomers in minutes: Rt_1 = 122.0 min; Rt_2 = 122.2 min.

^{1}H-NMR (500 MHz. $CDCl_3$): δ [ppm] = 5.84 (dd, 1 H, C**H**), 5.13 (dd, 1 H, CHC$\mathbf{H_2}$), 4.96 (dd, 1 H, C$\mathbf{H_2}$), 2.02 (s, 1H, O**H**), 1.45 (m, 2H, COHC$\mathbf{H_2}$), 1.24 (m, 17H, (C$\mathbf{H_2}$)$_6$C$\mathbf{H_2}$, COHC$\mathbf{H_3}$), 0.83 (t, 3H, $CH_2C\mathbf{H_3}$).

5.19 Synthesis of 3-methylhexadec-1-en-3ol (*rac*-C_{11})

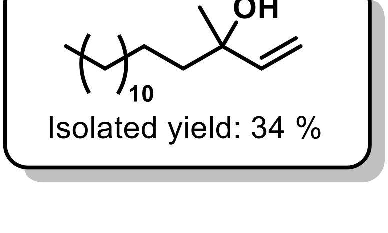

The synthesis was carried out according to GP F. Vinylmagnesium bromide in THF (10 mmol) and 2-pentadecanone (7 mmol) were utilized for the reaction. Work up and purification yielded the compound (*rac*-$\mathbf{C_{11}}$, 0.612 g, 2.41 mmol, 34%) as a colorless liquid.

The chiral GC-analysis of the generated alcohol *rac*-**C_{11}** was done using an AGILENT CP-Chirasil-Dex CB column 30 m x 250µm x 0.25 µm) and helium as the carrier gas in constant flow of 1.2 mL/min., 15:1 split ration, 1µL injection volume. The oven program for the analysis: starting at 100°C with an immediate gradient of 5°C/min to 140°C and directly to a 0.1°C/min gradient to 146°C and then to a 30°C/min gradient to 180°C and hold 2 min. Retention times of *R*/*S* enantiomers in minutes: Rt_1 = 58.6.0 min; Rt_2 = 59.5 min.

^{1}H-NMR (500 MHz. $CDCl_3$): δ [ppm] = 5.91 (dd, 1 H, -C**H**), 5.19 (dd, 1 H, -CHC**H_2**), 5.04 (dd, 1 H, -C**H_2**), 1.51 (m, 3H, C**H_3**CCOH), 1.26 (m, 24H, (C**H_2**)$_{12}$CH3), 0.88 (t, 3H, - CH_2C**H_3**).

5.20 Synthesis of 3,5-dimethylhex-1-en-3ol (*rac*-1o)

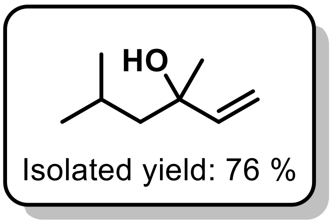

The synthesis was carried out according to GP F. Vinylmagnesium bromide in THF (10 mmol) and 4-methyl-2-pentanone (7 mmol) were performed for the reaction. Work up and purification yielded the compound (*rac*-**1o**, 0.685 g, 5.34 mmol, 76%) as a colorless liquid.

^{1}H NMR (500 MHz, $CDCl_3$) δ 5.93 (dd, *J* = 17.4, 10.7 Hz, 1H, OH-C-C**H**-CH_2), 5.20 (dd, *J* = 17.4, 1.3 Hz, 1H, OH-C-CH-C**H_2**), 5.02 (dd, *J* = 10.7, 1.3 Hz, 1H, OH-C-CH-C**H_2**), 1.74 (m, *J* = 12.9, 6.5 Hz, 1H, C**H**-CH_2), 1.45 (m, 2H, C**H_2**), 1.27 (s, 3H, C**H_3**), 0.93 (dd, 6H, CC**H_3**).

5.21 Synthesis of 3-isopropylhex-1-en-3-ol (*rac*-p)

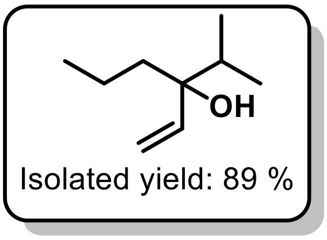

The synthesis was carried out according to GP F. Vinylmagnesium bromide in THF (10 mmol) and 2,4-dimethyl-3-pentanone (7 mmol) were performed for the reaction. Work up and purification yielded the compound (*rac*-**1p**, 0.892 g, 6.27 mmol, 89%) as a colorless liquid.

^{1}H NMR (500 MHz, $CDCl_3$) δ 5.81 (dd, *J* = 17.4, 10.9 Hz, 1H, OH-C-C**H**-CH_2), 5.19 (dd, *J* = 17.4, 1.5 Hz, 1H, OH-C-CH-C$\mathbf{H_2}$), 5.13 (dd, *J* = 10.9, 1.5 Hz, 1H, OH-C-CH-C$\mathbf{H_2}$), 1.72 (m, 1H, C**H**CH_3), 1.48 (m, 2H, OH-C-C$\mathbf{H_2}$), 1.27 (m, 2H, C$\mathbf{H_2}$-CH_3), 0.88 (m, 9H, CH_2C$\mathbf{H_3}$, C$\mathbf{H_3}$CC$\mathbf{H_3}$).

5.22 Synthesis of 5-isopropoxy-3-methylpent-1-en-3-ol (*rac*-1q)

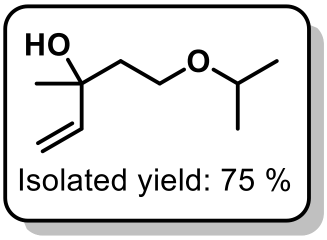

The synthesis was performed in analogy to GP F. Grignard reagent (10 mmol) and 3-decen-2-on (7 mmol) were applied for the reaction. Work up and purification yielded the compound (*rac*-**1q**, 828 mg, 5.23 mmol, 75%) as a colorless liquid.

^{1}H NMR (500 MHz, $CDCl_3$) δ 5.85 (dd, *J* = 17.1, 10.6 Hz, 1H, C**H**-CH_2), 5.31 (dd, *J* = 17.2, 1.7 Hz, 1H, CH-C$\mathbf{H_2}$), 5.05 (dd, 1H, CH-C$\mathbf{H_2}$), 3.59 (m, 2H, O-C$\mathbf{H_2}$), 3.52 (p, *J* = 6.1 Hz, 1H, O-C**H**-CH_3), 1.90 (dt, *J* = 14.3, 6.9 Hz, 1H, O-CH_2-C$\mathbf{H_2}$), 1.65 (dt, *J* = 14.6, 4.2 Hz, 1H, O-CH_2-C$\mathbf{H_2}$), 1.25 (s, 3H, C-C$\mathbf{H_3}$), 1.13 (dd, *J* = 6.1, 1.4 Hz, 6H, C-(C$\mathbf{H_3}$)$_2$).

5.23 Synthesis of *1S, 2R, 4R*-2-vinylbicyclo [2.2.1]-heptan-2-ol (*rac*-1r)

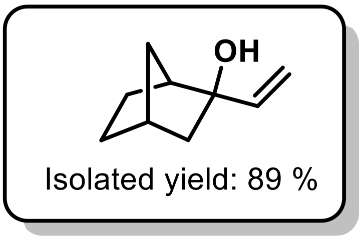

The synthesis was carried out according to GP F. Vinylmagnesium bromide in THF (10 mmol) and norcamphor (8 mmol) were applied for the reaction. Work up and purification yielded the compound (*rac*-**1r**, 1.006 g, 7.27 mmol, 89%) as a colorless liquid.

^{1}H-NMR (500 MHz. $CDCl_3$): *δ* [ppm] = 5.95 (dd, 1H, C**H**CH_2), 5.08 (dd, 1H, CHC**H**$_2$), 4.89 (dd, 1H, CHC**H**$_2$), 2.22 (s, 1H, O**H**), 2.14 (m, 1H, CH_2C**H**CH_2), 1.98 (m, 2H, C**H$_2$**CHCH_2), 1.74 (m, 1H, C**H**COH), 1.48 (m, 2H, C**H$_2$**CHCOH), 1.28 (m, 2H, CHC**H$_2$**CH), 1.20 (m, 1H, C**H**$_2$COH), 1.11 (m, 1H, C**H**$_2$COH).

5.24 Synthesis of 1-vinylcyclooctan-1-ol ((*rac*-1s)

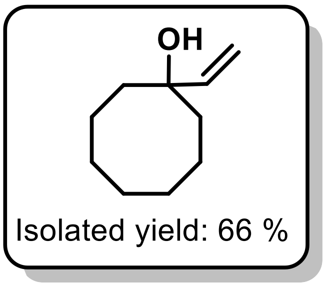

The synthesis was conducted in analogy to GP F. Vinylmagnesium bromide in THF (10 mmol) and cyclooctanone (8 mmol) were applied for the reaction. Work up and purification yielded the compound (*rac*-**1s**, 811 mg, 5.26 mmol, 66%) as a colorless liquid.

^{1}H-NMR (500 MHz. $CDCl_3$): *δ* [ppm] = 6.01 (dd, 1H, C**H**CH_2), 5.22 (dd, 1H, CHC**H**$_2$), 5.02 (dd, 1H, CHC**H**$_2$), 1.77 (m, 2H, C**H$_2$**$(CH_2)_3$COH), 1.66 (m, 8H, (C**H$_2$**)$_2$$CH_2$COH), 1.50 (m, 4H, C**H$_2$**COH).

6. NMR SPECTRA

6.1 ^{1}H-NMR: *rac*-1a

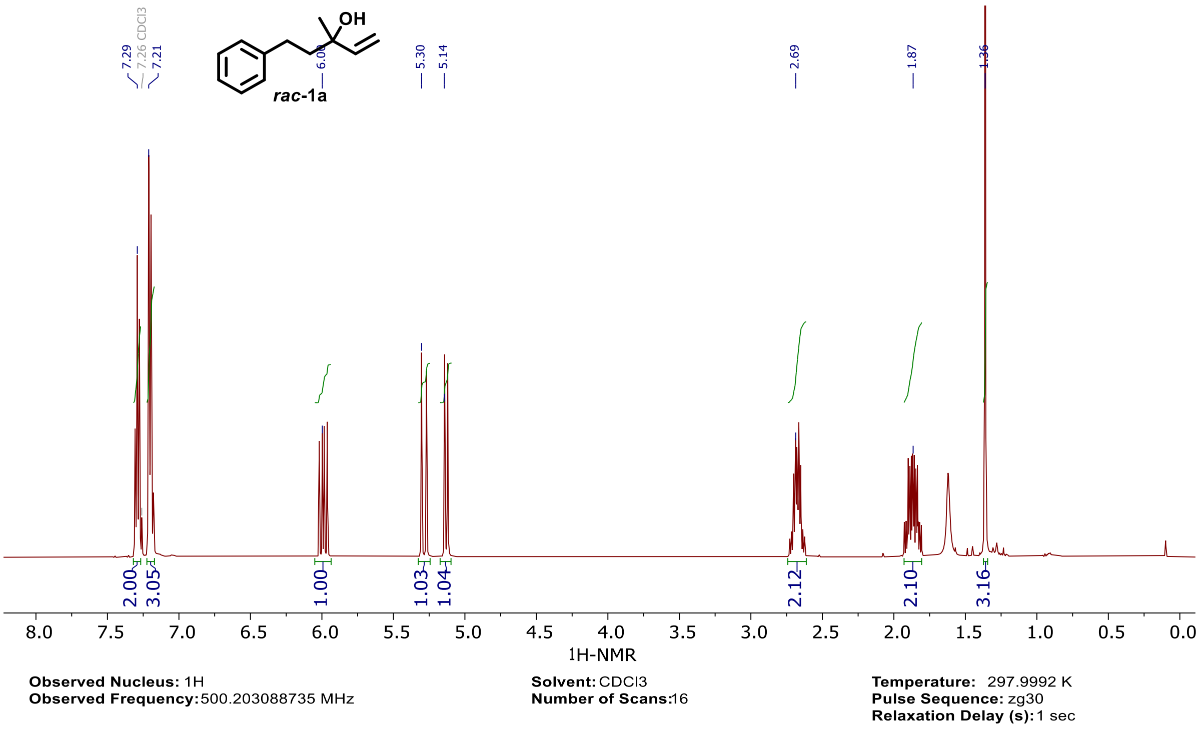

6.2 ^{1}H-NMR: Example-Biocatalytic kinetic resolution-1. Round (500 mM reaction, 5 mL)

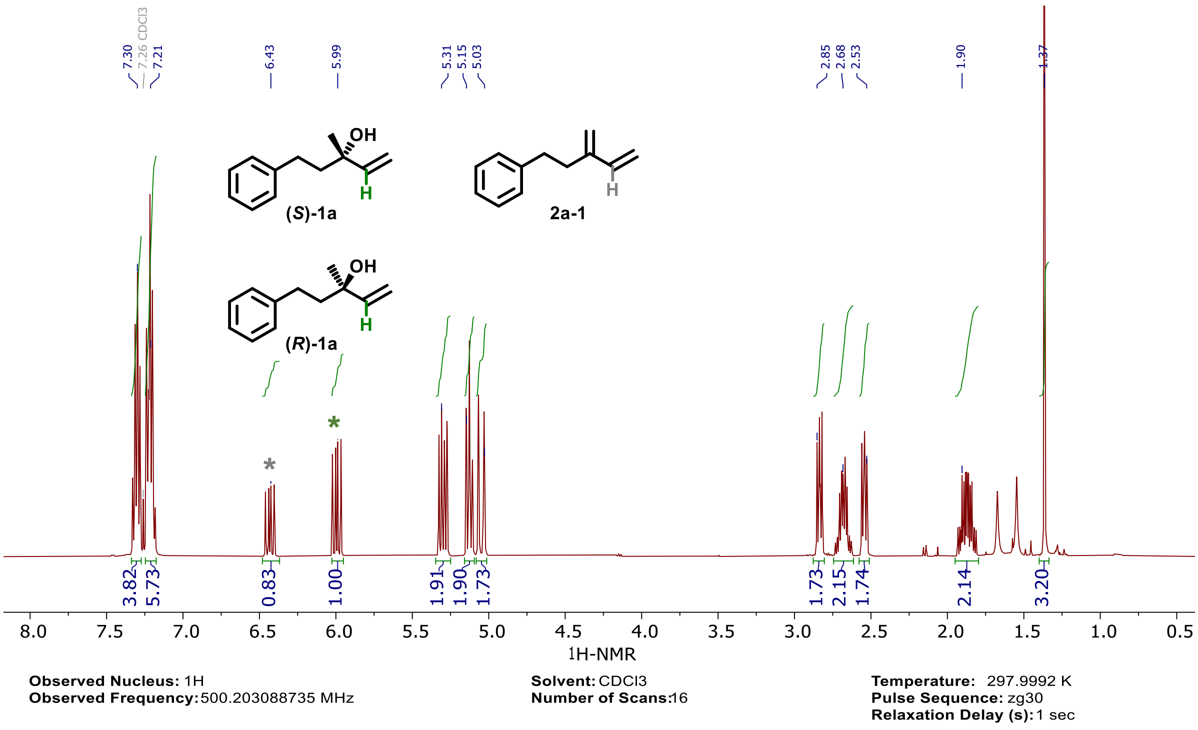

6.3 ^{1}H-NMR: Example-Biocatalytic kinetic resolution-1. Round (500 mM reaction, 50mL)

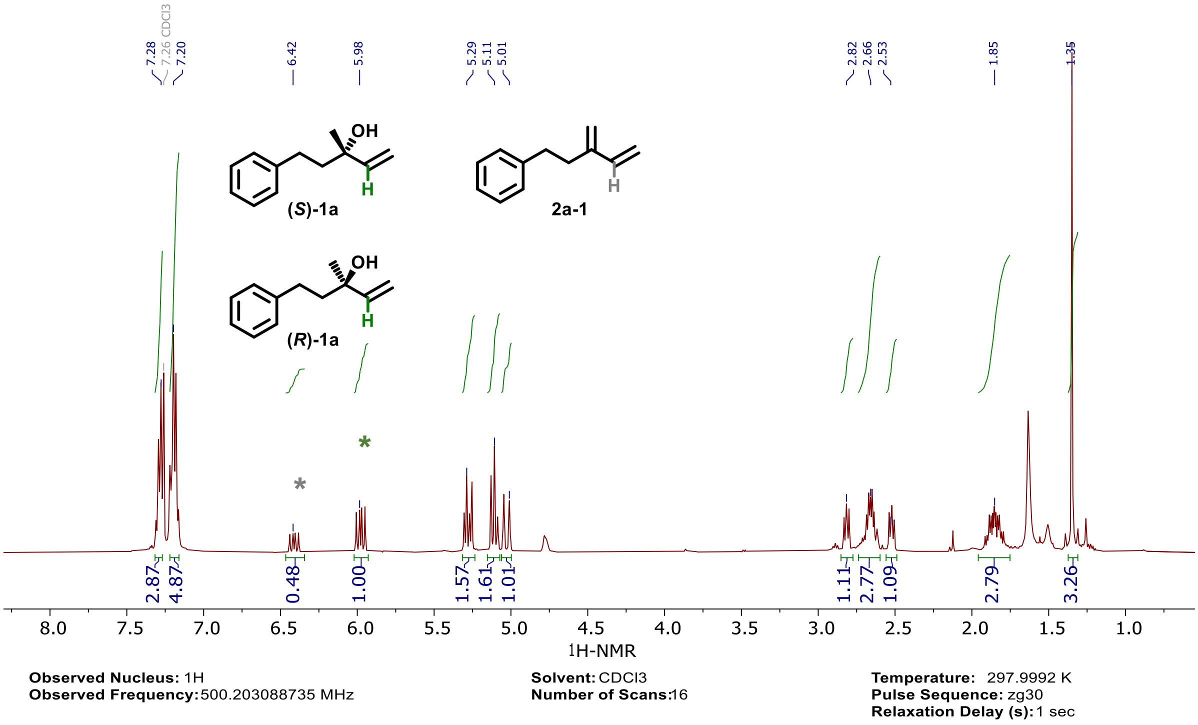

6.4 ^{1}H-NMR: Example-Biocatalytic kinetic resolution-2. Round (500 mM reaction, 50mL)

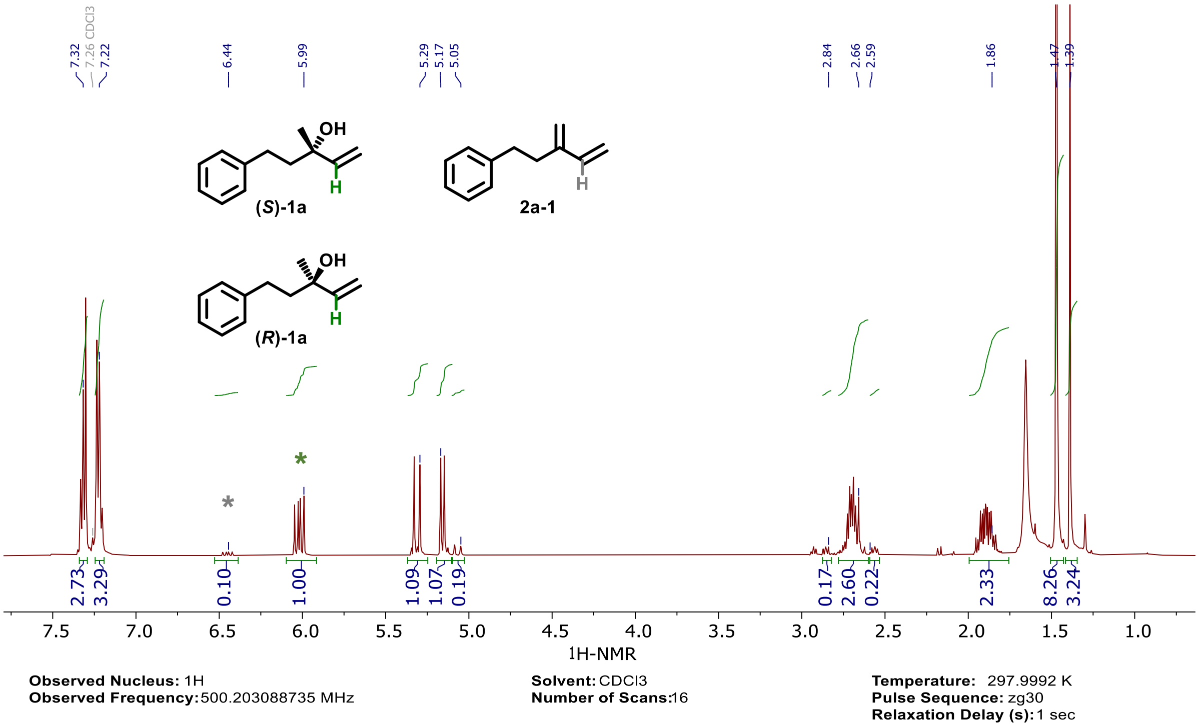

6.5.1 ^{1}H-NMR: Isolated Hofmann product 2a-1

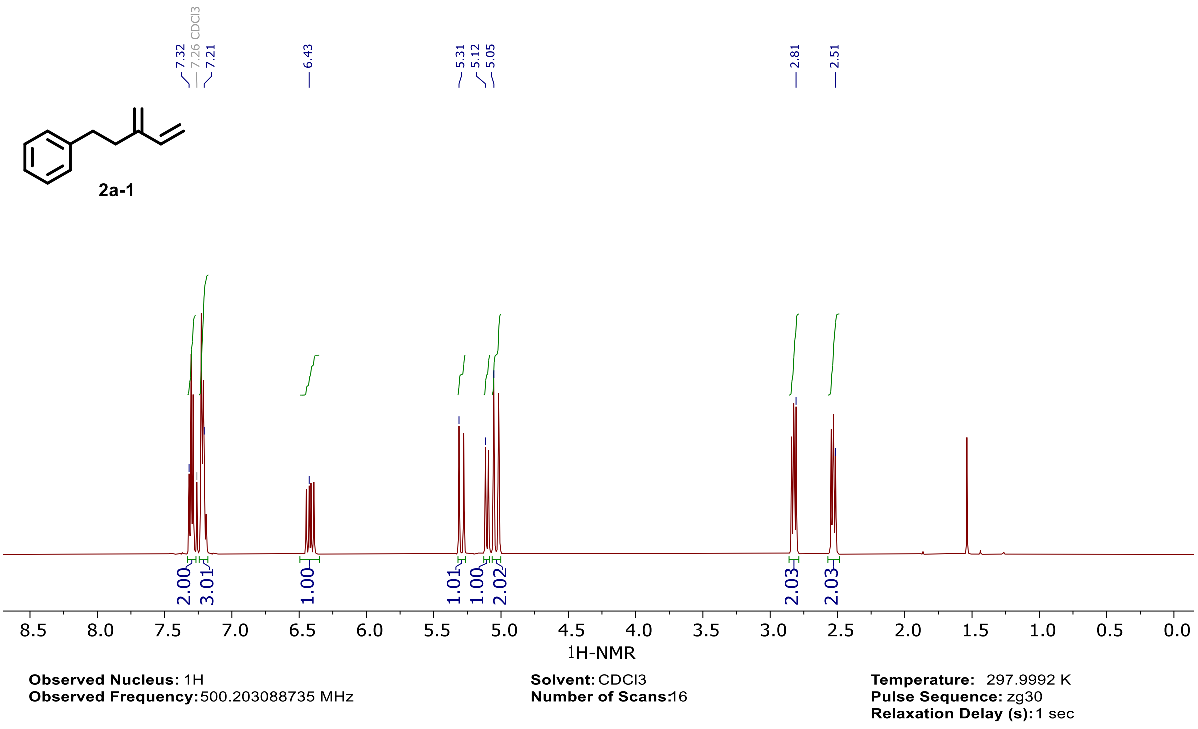

6.5.2 ^{13}C-NMR: Isolated Hofmann product 2a-1

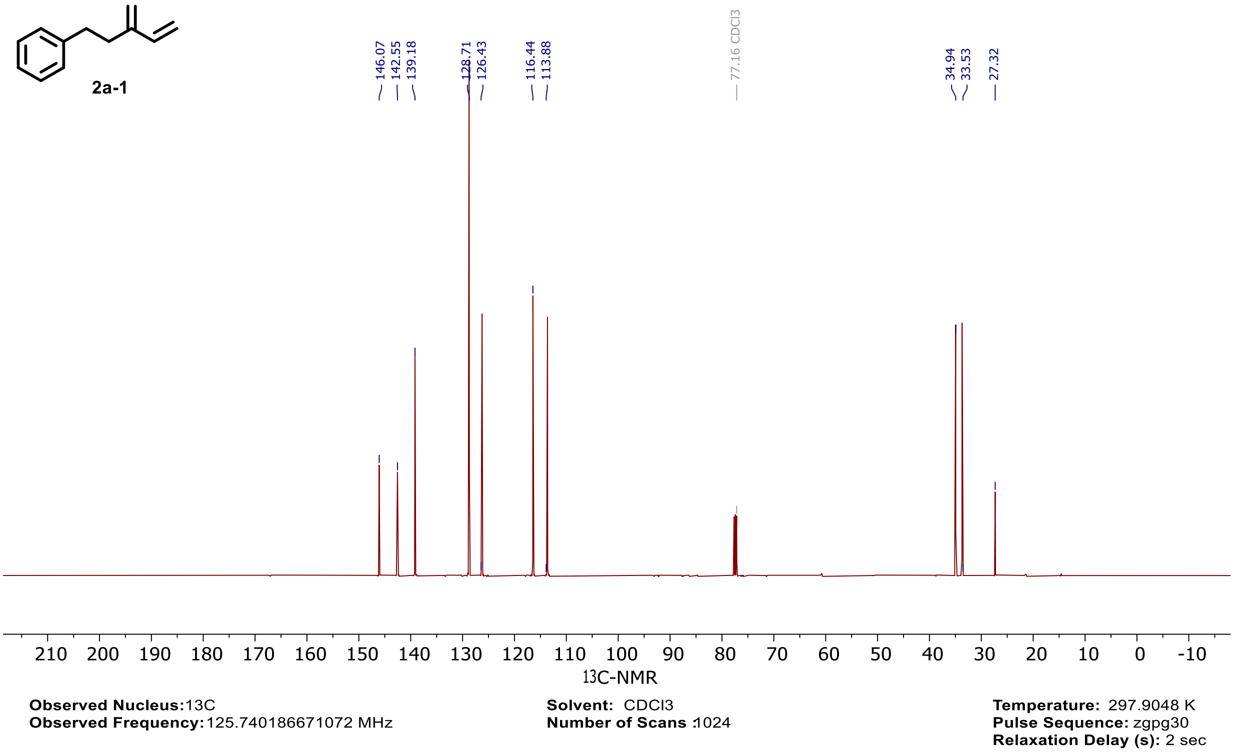

6.5.3 ^{13}C DEPT 135 NMR: Isolated Hofmann product 2a-1

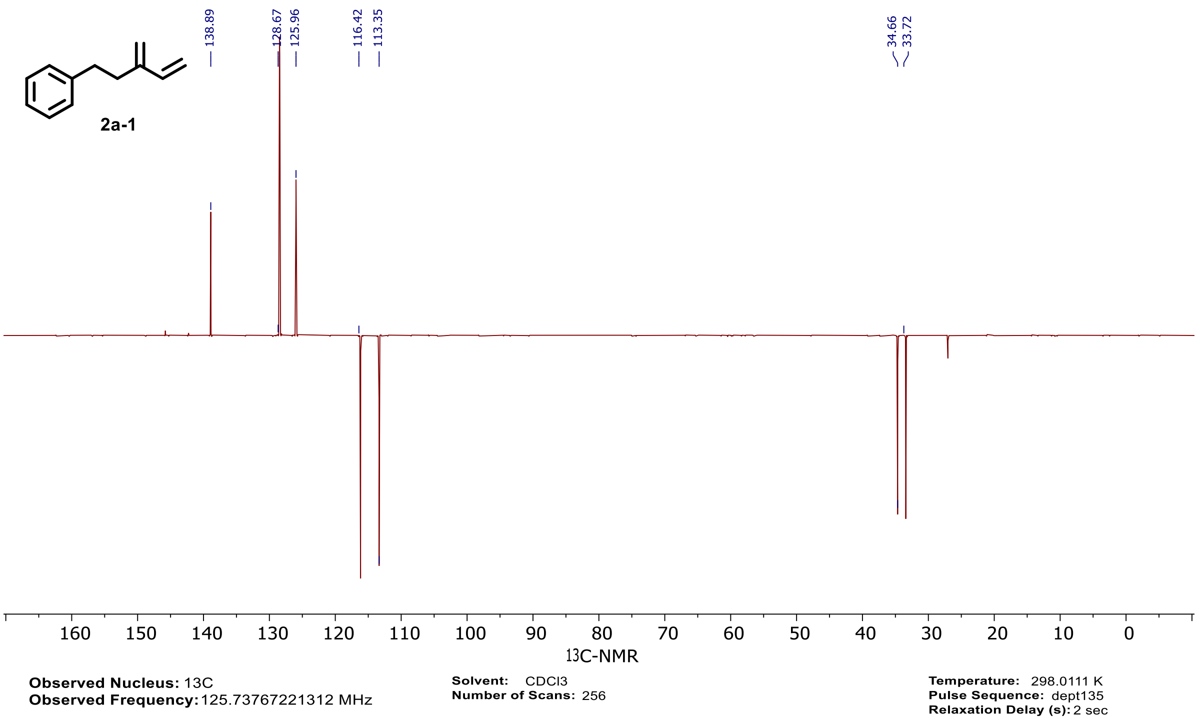

6.5.4 COSY: Isolated Hofmann product 2a-1

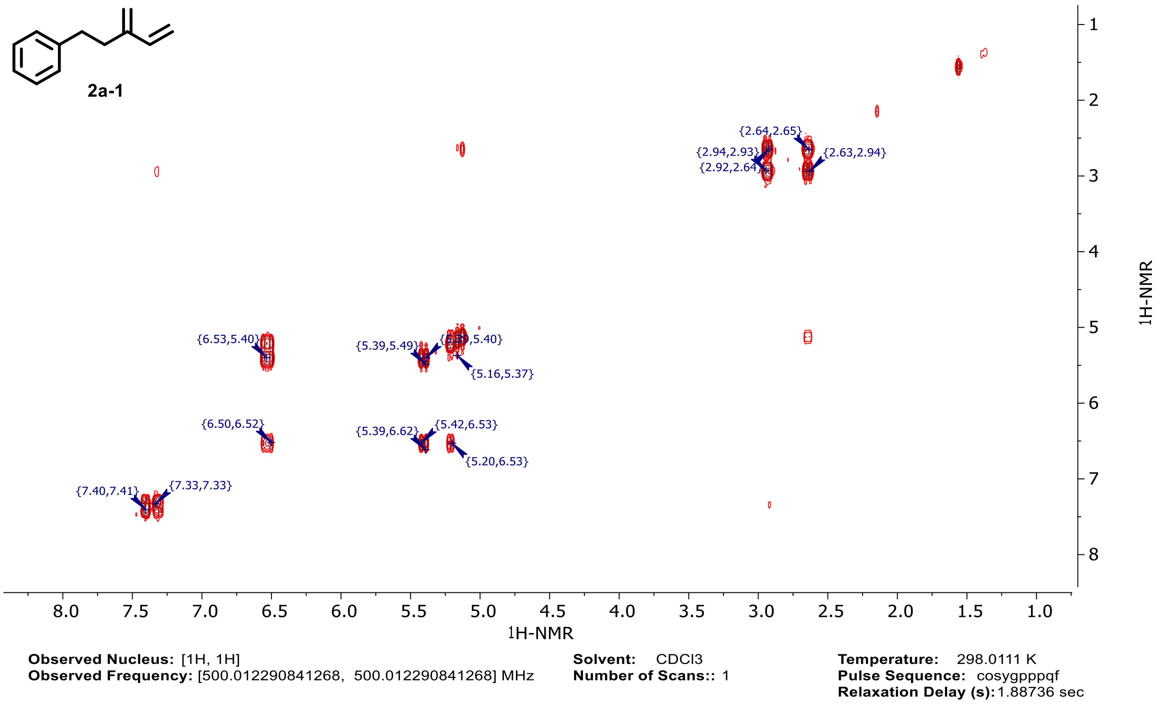

6.5.5 HMBC: Isolated Hofmann product 2a-1

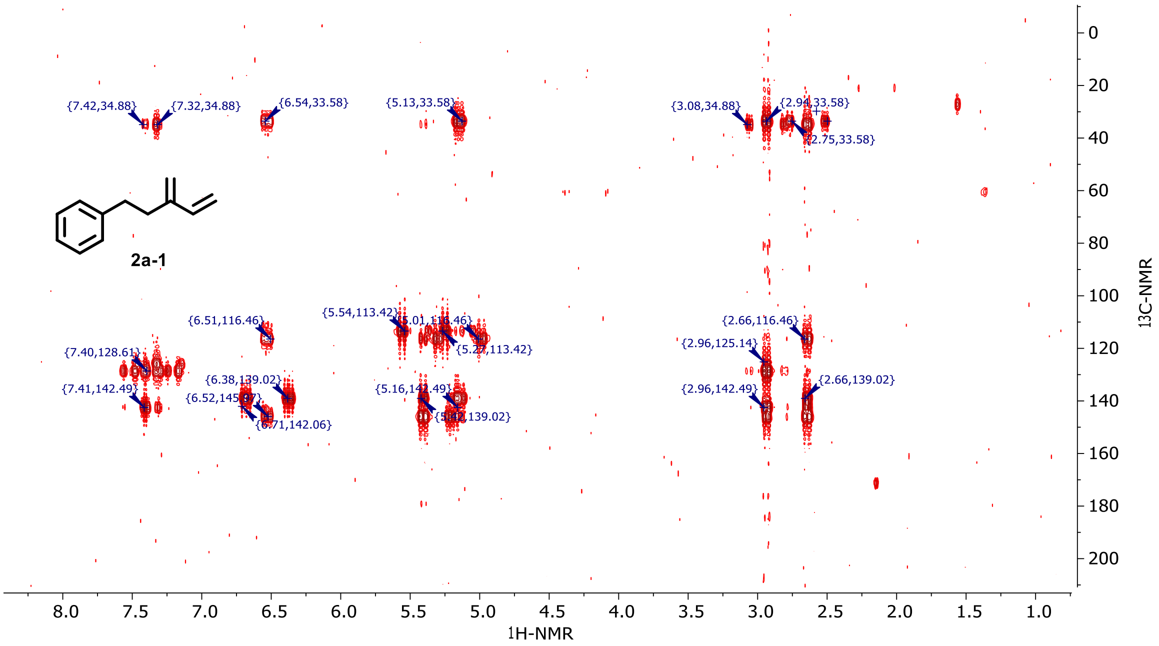

Observed Nucleus: [13C, 1H]
Observed Frequency: [125.740167244107, 500.012290841268] MHz
Solvent: CDCl3
Number of Scans: 8
Temperature: 297.9048 K
Pulse Sequence: hmbcgpndqf
Relaxation Delay (s): 1.27472 sec

6.5.6 HMQC: Isolated Hofmann product 2a-1

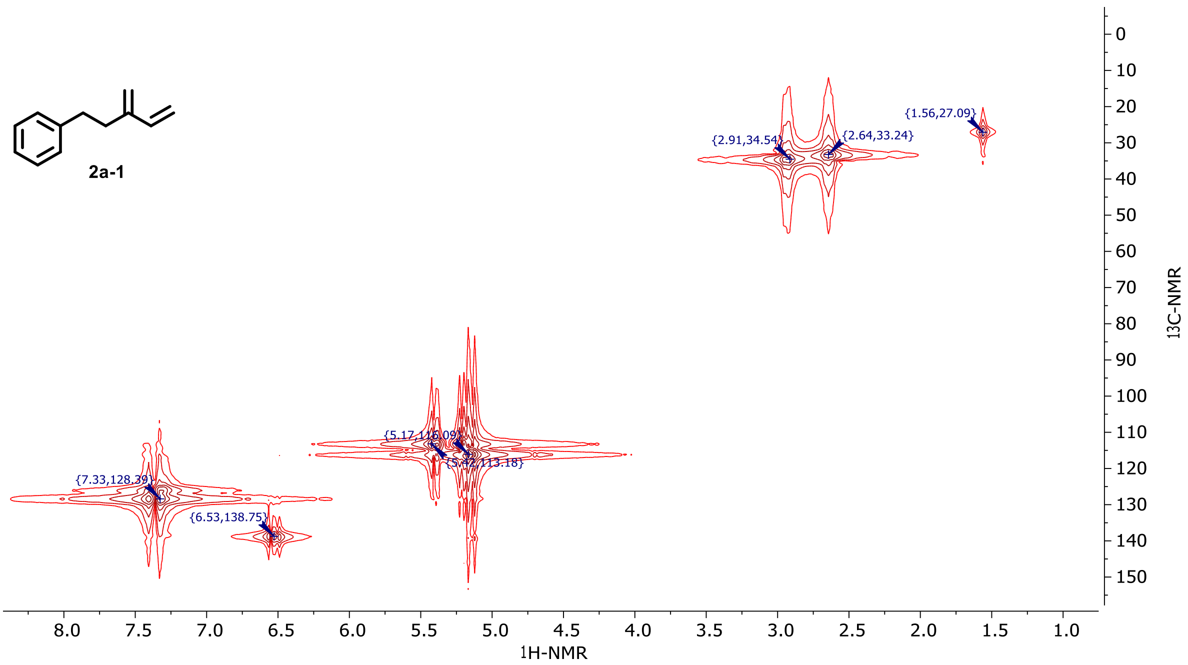

Observed Nucleus: [13C, 1H]
Observed Frequency: [125.737043480723, 500.012290841268] MHz
Solvent: CDCl3
Number of Scans: 4
Temperature: 298.0111 K
Pulse Sequence: hmqcgpqf
Relaxation Delay (s): 1.46928 sec

6.6 ^{1}H-NMR: Isolated alcohol (*R*/*S* = 94:6), 5 mL-Scale up

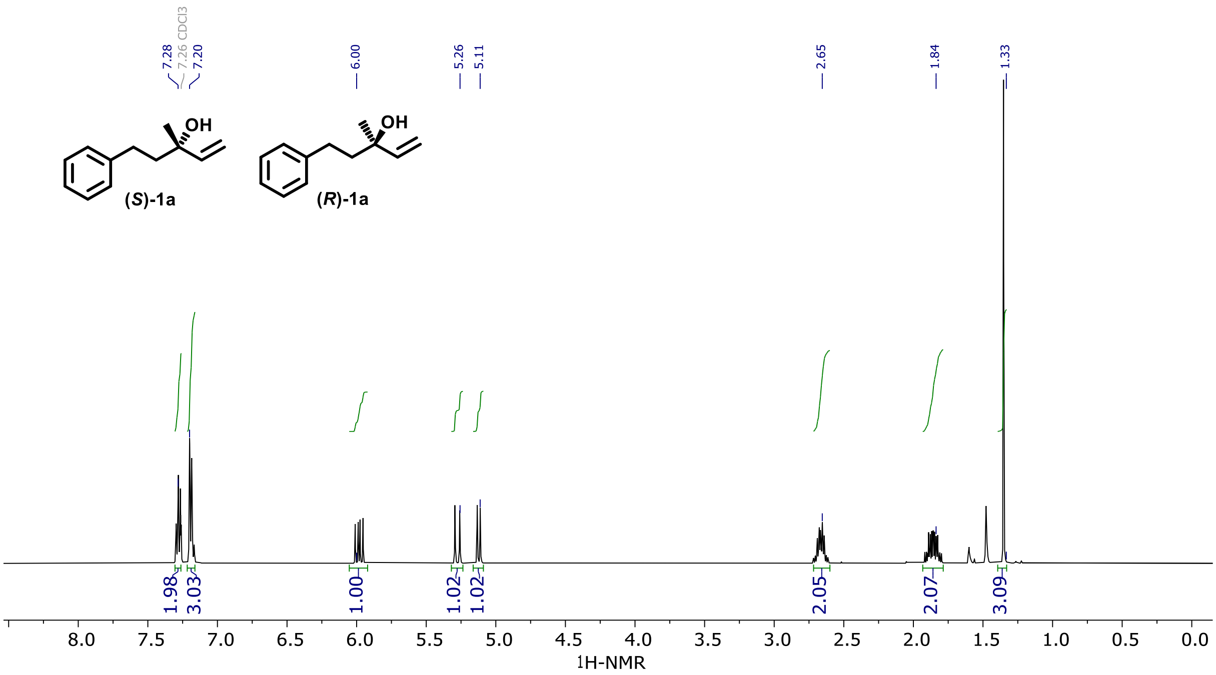

Observed Nucleus: 1H
Observed Frequency: 500.203088735 MHz

Solvent: CDCl3
Number of Scans: 16

Temperature: 298.0003 K
Pulse Sequence: zg30
Relaxation Delay (s): 1 sec

6.7 Kinetic resolution-2.Round purified (*R*)-1a

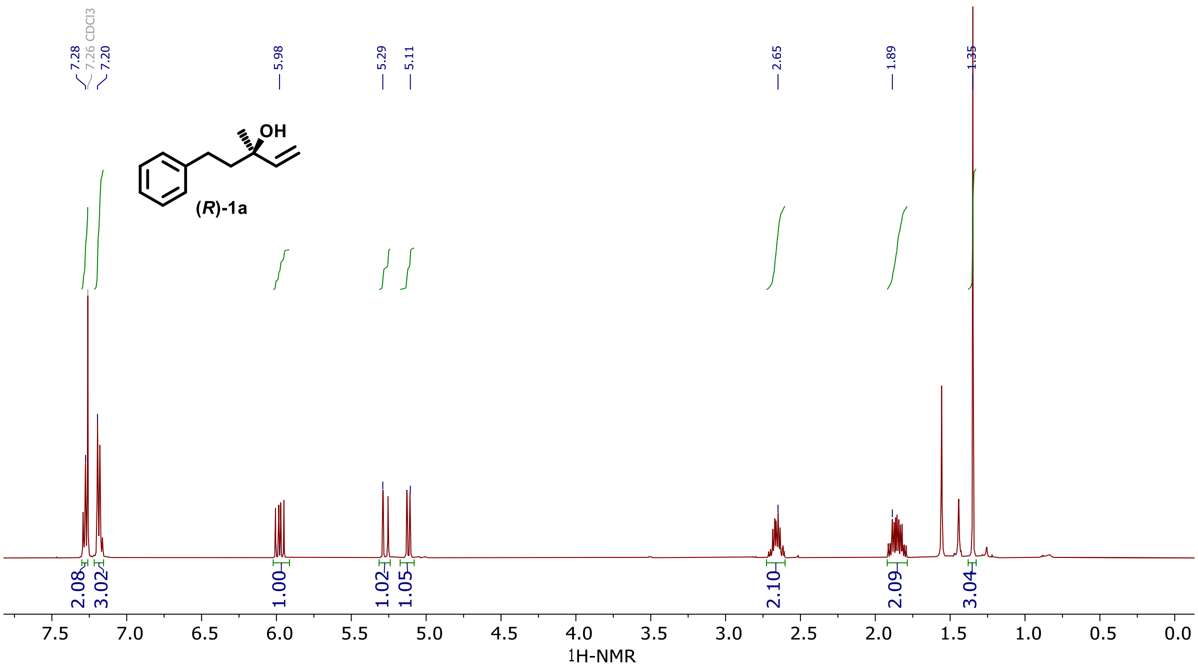

Observed Nucleus: 1H
Observed Frequency: 500.203088735 MHz

Solvent: CDCl3
Number of Scans: 128

Temperature: 298.0025 K
Pulse Sequence: zg30
Relaxation Delay (s): 1 sec

6.8 ^{1}H-NMR: Dehydration of (*R*)-1a, 250 mL, 10 mM

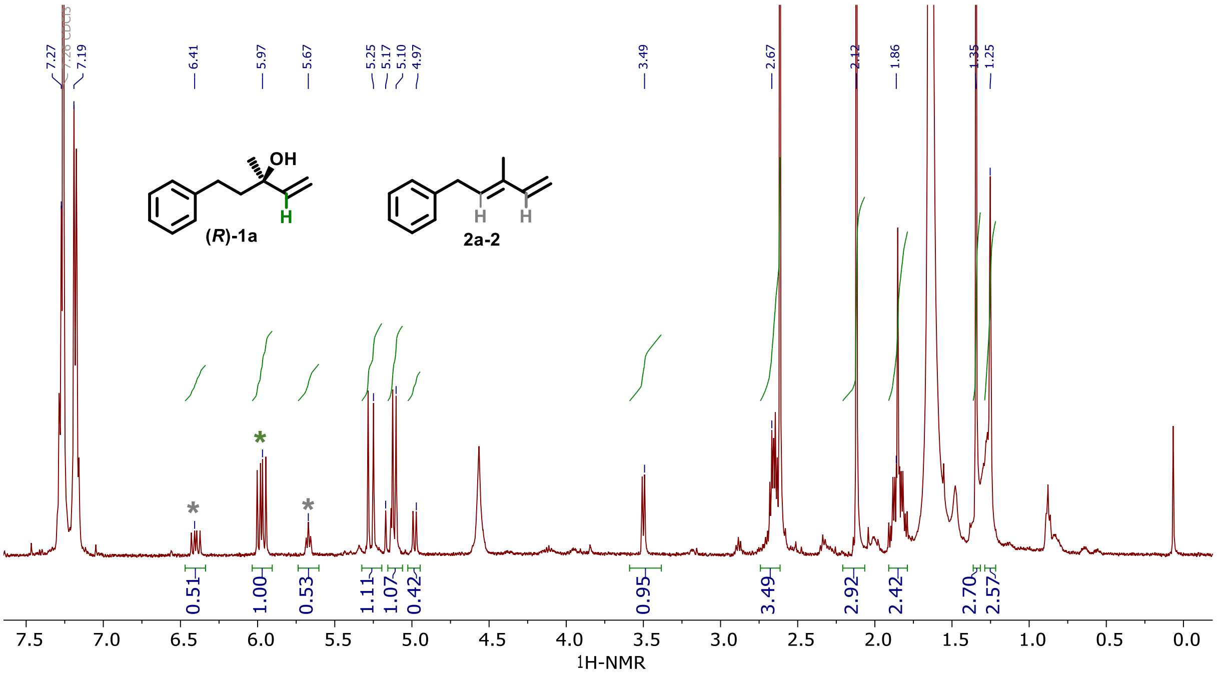

Observed Nucleus: 1H
Observed Frequency: 500.203088735 MHz
Solvent: CDCl3
Number of Scans: 16
Temperature: 298.0003 K
Pulse Sequence: zg30
Relaxation Delay (s): 1 sec

6.9.1 ^{1}H-NMR: Isolated Saytzeff product 2a-2

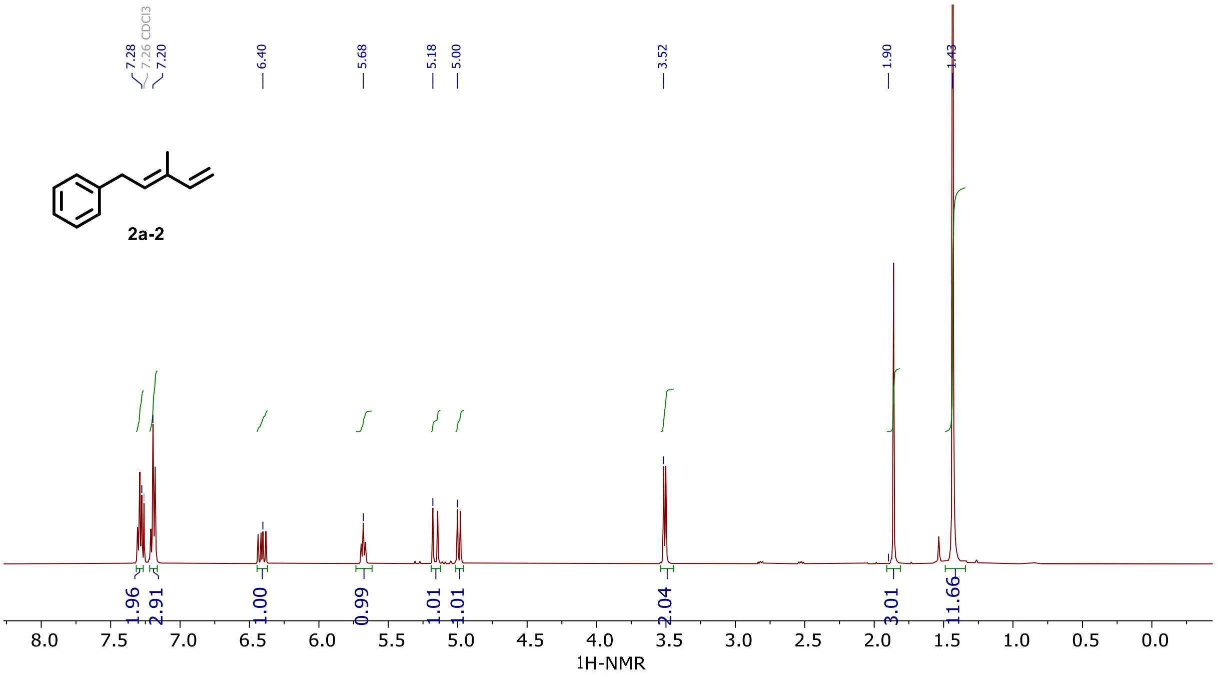

Observed Nucleus: 1H
Observed Frequency: 500.203088735 MHz
Solvent: CDCl3
Number of Scans: 16
Temperature: 298.0003 K
Pulse Sequence: zg30
Relaxation Delay (s): 1 sec

6.9.2 ^{13}C-NMR: Isolated Saytzeff product 2a-2

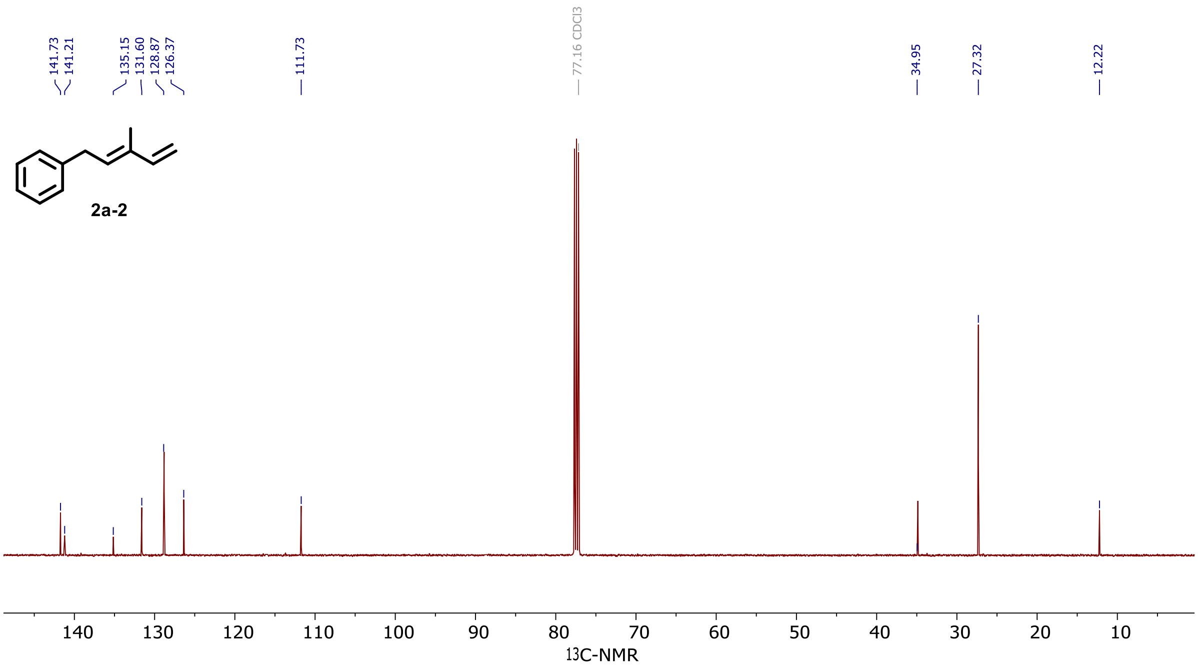

Observed Nucleus: 13C
Observed Frequency: 125.740186671072 MHz
Solvent: CDCl3
Number of Scans: 2048
Temperature: 297.9048 K
Pulse Sequence: zgpg30
Relaxation Delay (s): 2 sec

6.9.3 ^{13}C DEPT 135 NMR:

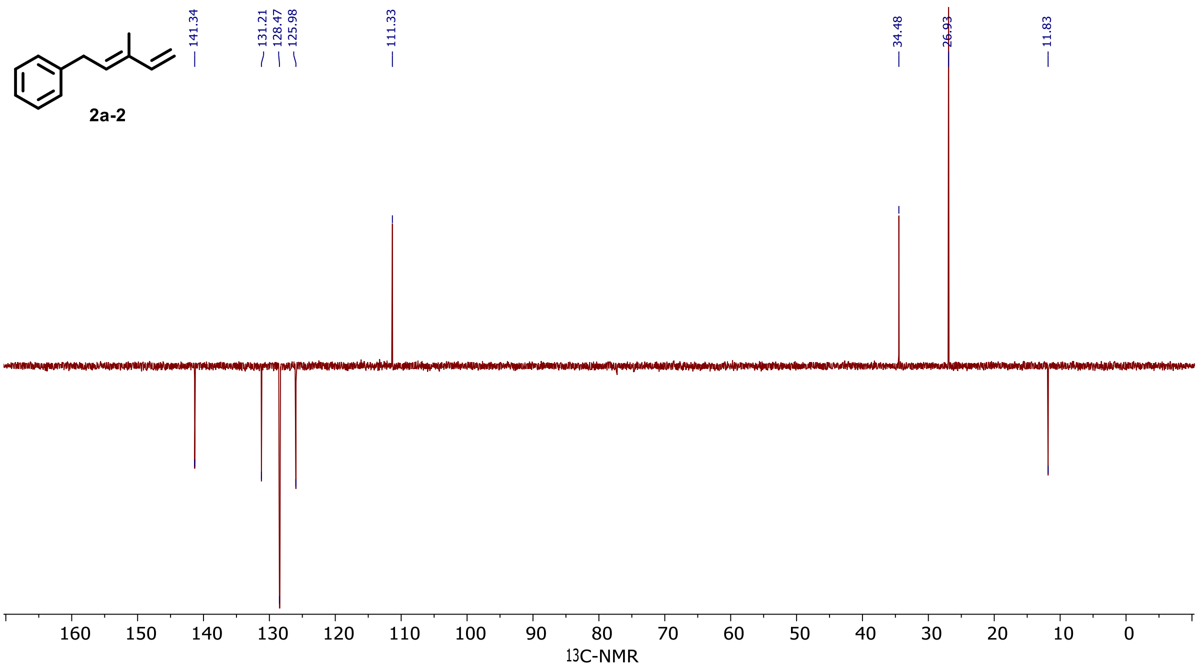

Observed Nucleus: 13C
Observed Frequency: 125.73767221312 MHz
Solvent: CDCl3
Number of Scans: 512
Temperature: 297.9048 K
Pulse Sequence: dept135
Relaxation Delay (s): 2 sec

6.9.4 COSY: Isolated Saytzeff product 2a-2

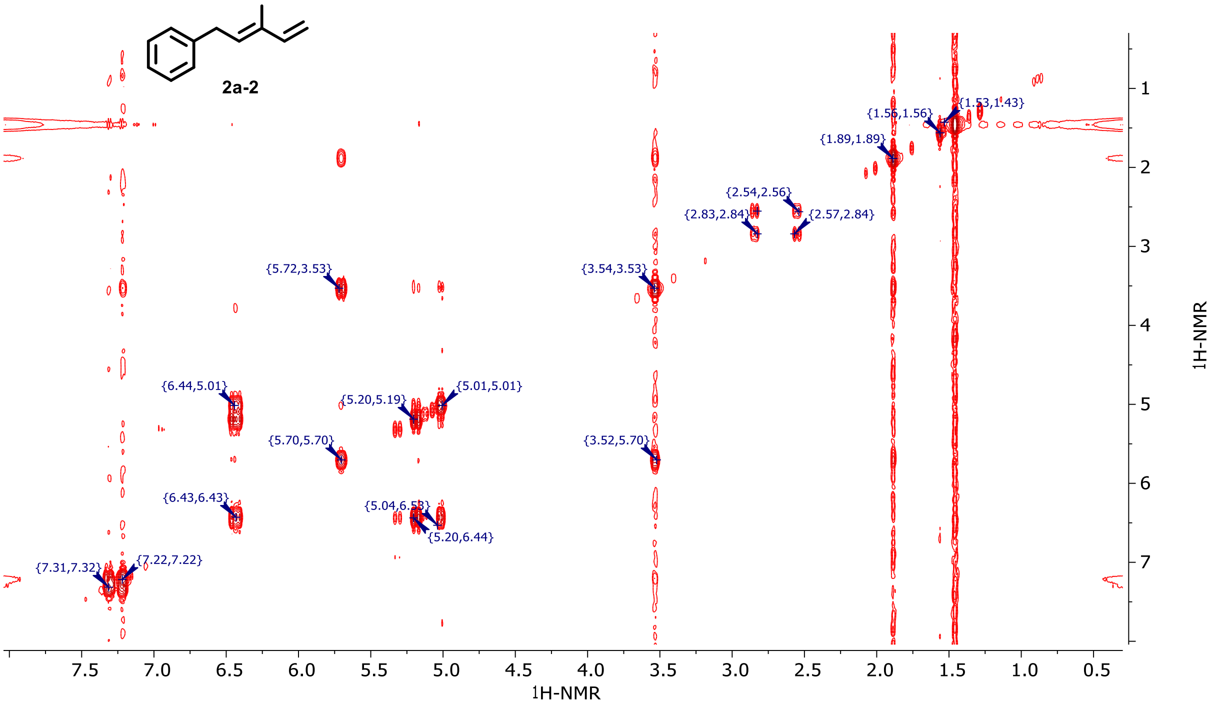

Observed Nucleus: [1H, 1H]
Observed Frequency: [500.012083015079, 500.012083015079] MHz
Solvent: CDCl3
Number of Scans: 1
Temperature: 297.9048 K
Pulse Sequence: cosygpppqf
Relaxation Delay (s): 1.889408 sec

6.9.5 HMBC: Isolated Saytzeff product 2a-2

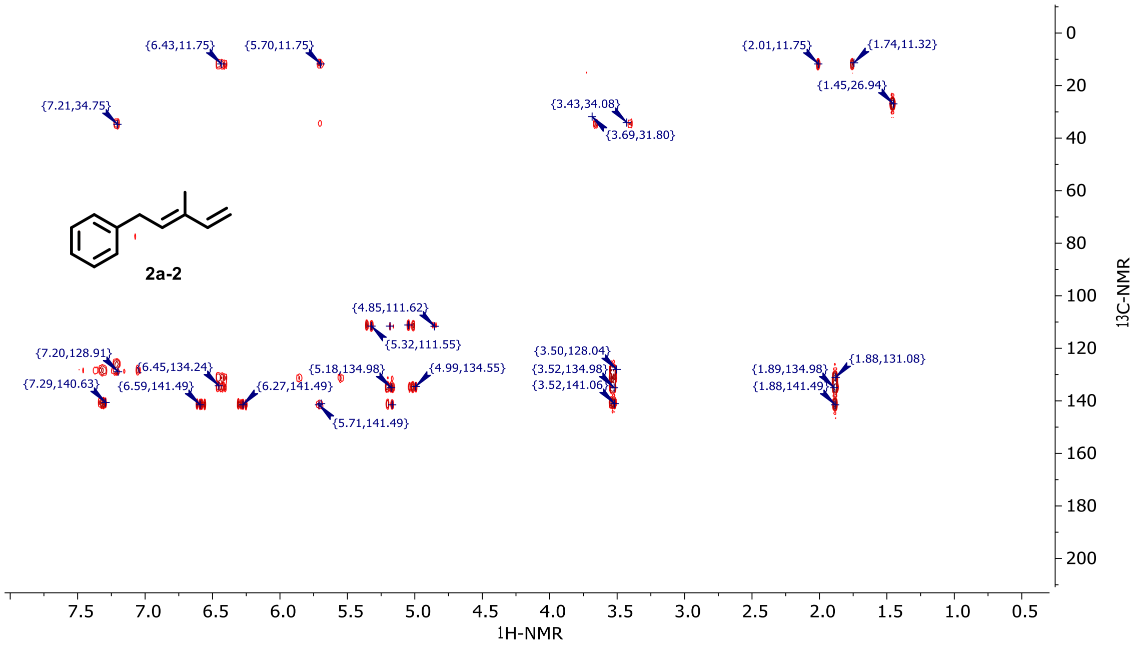

Observed Nucleus: [13C, 1H]
Observed Frequency: [125.740167244107, 500.012083015079] MHz
Solvent: CDCl3
Number of Scans: 8
Temperature: 297.9048 K
Pulse Sequence: hmbcgpndqf
Relaxation Delay (s): 1.278816 sec

6.9.6 HMQC: Isolated Saytzeff product 2a-2

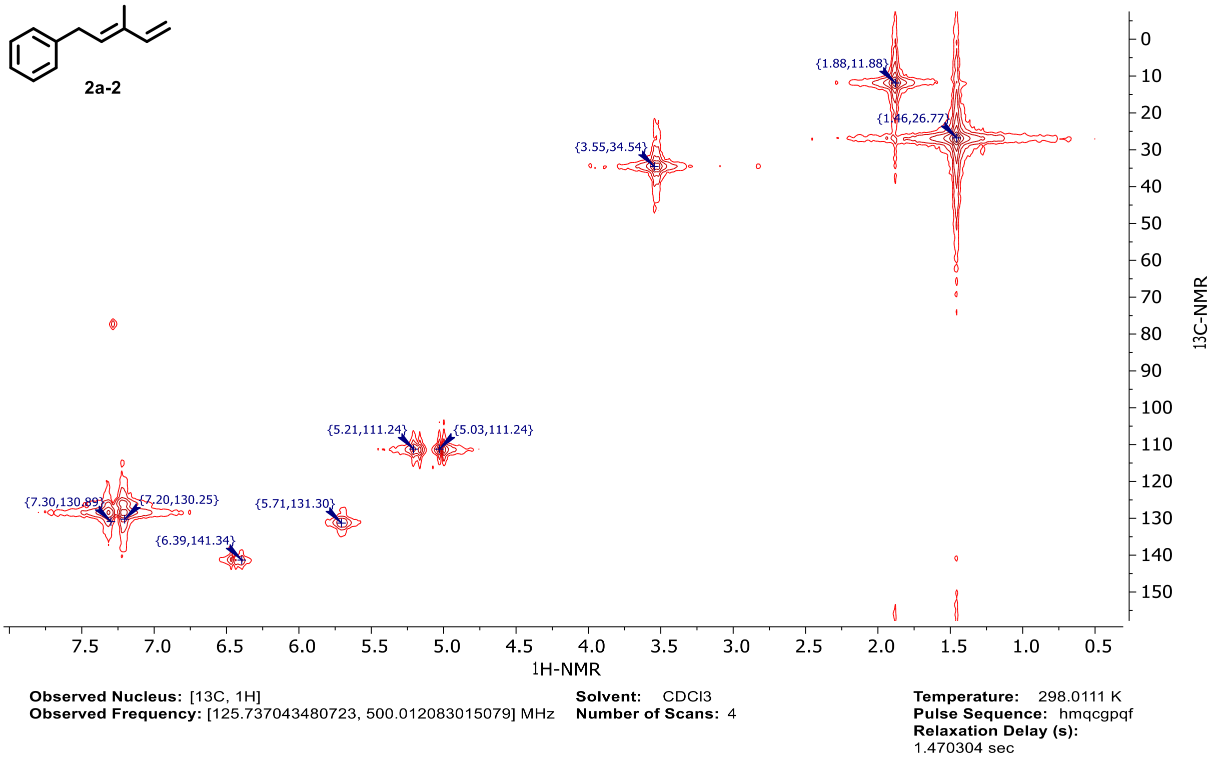

6.10 ^{1}H-NMR: Reference-4-phenyl-2-butanone

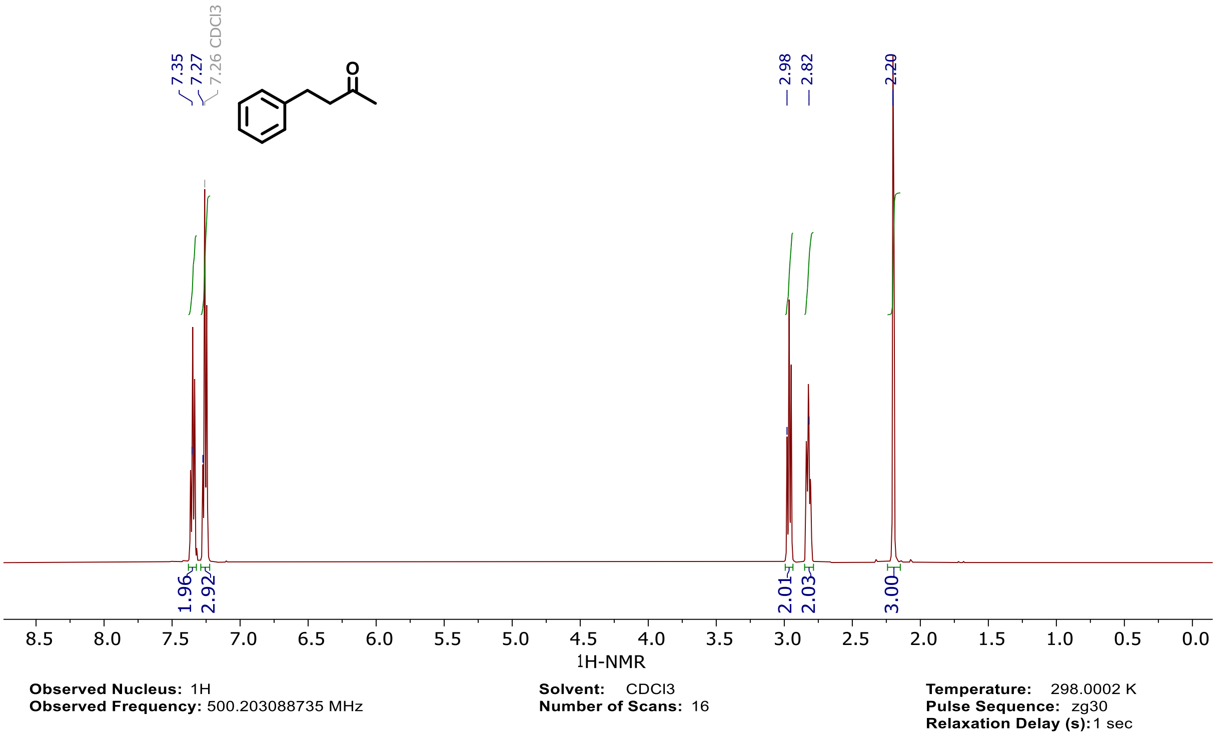

6.11 ^{1}H-NMR: Reference-*rac*-linalool (*rac*-1b)

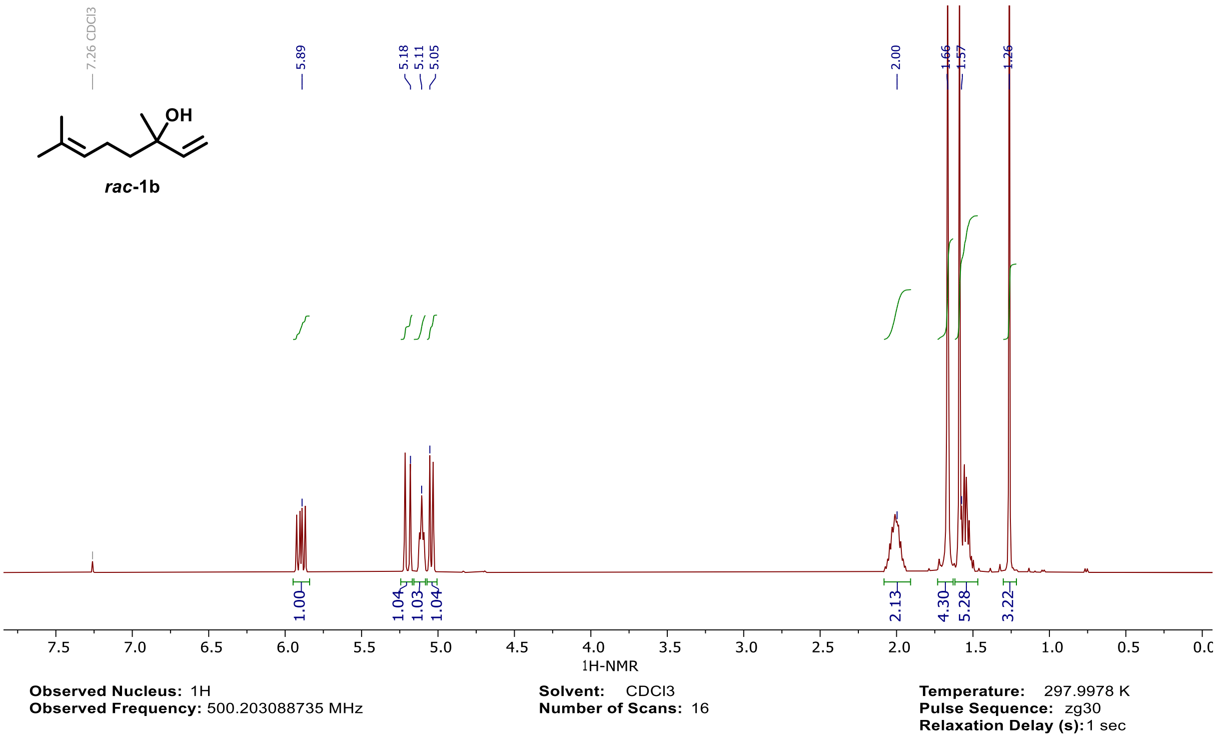

6.12 ^{1}H-NMR: Reference-β-myrcene (2b-1)

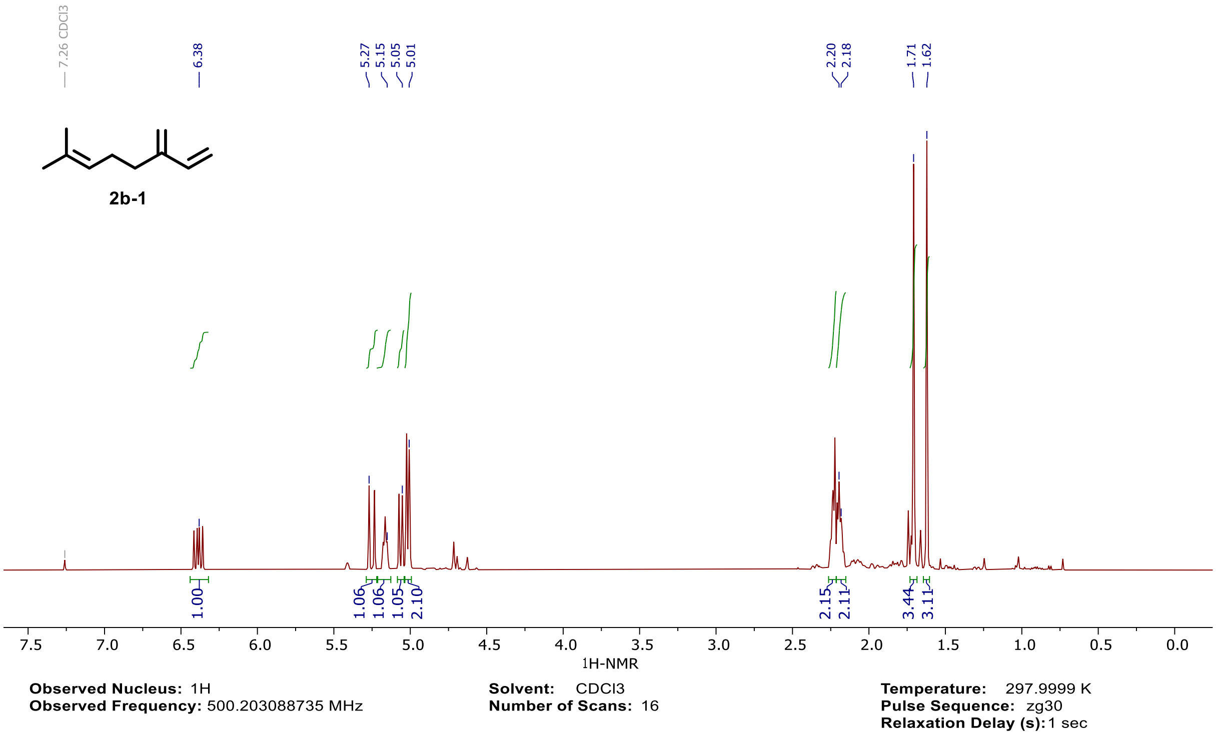

6.13.1 ^{1}H-NMR: Reference- β-ocimene (*Z*-isomer)

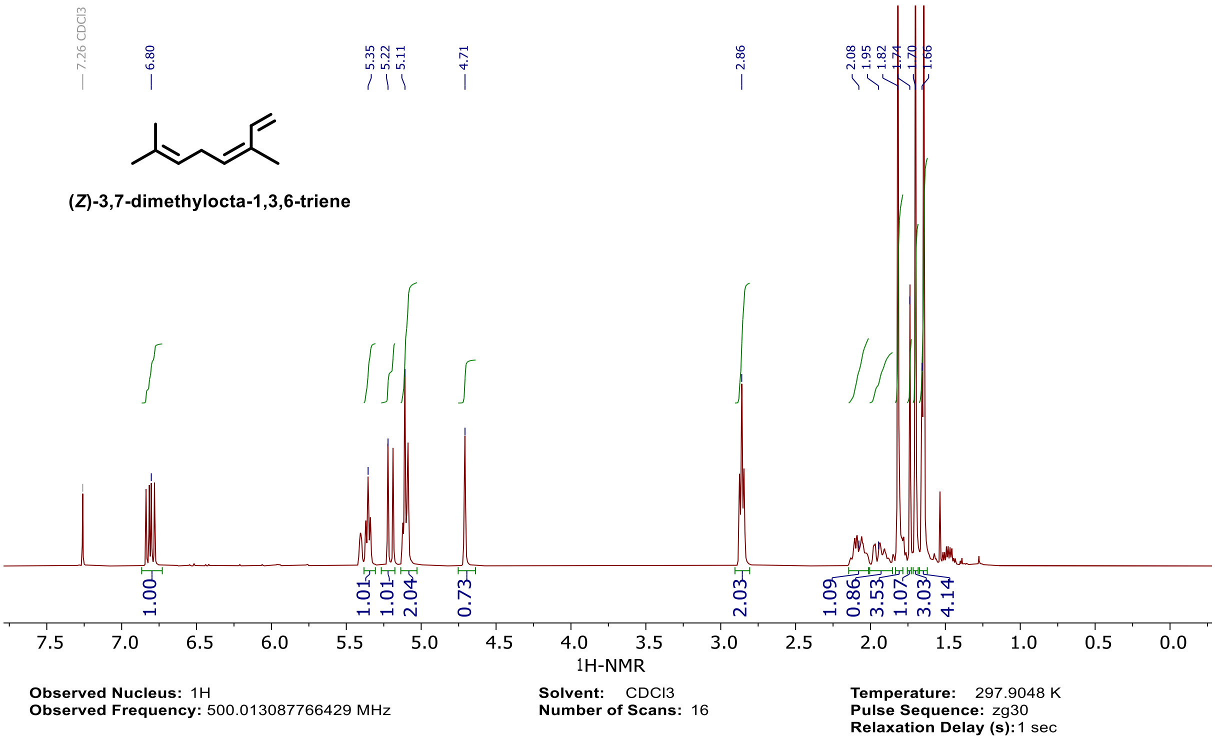

6.13.2 ^{13}C-NMR: Reference- β-ocimene (*Z*-isomer)

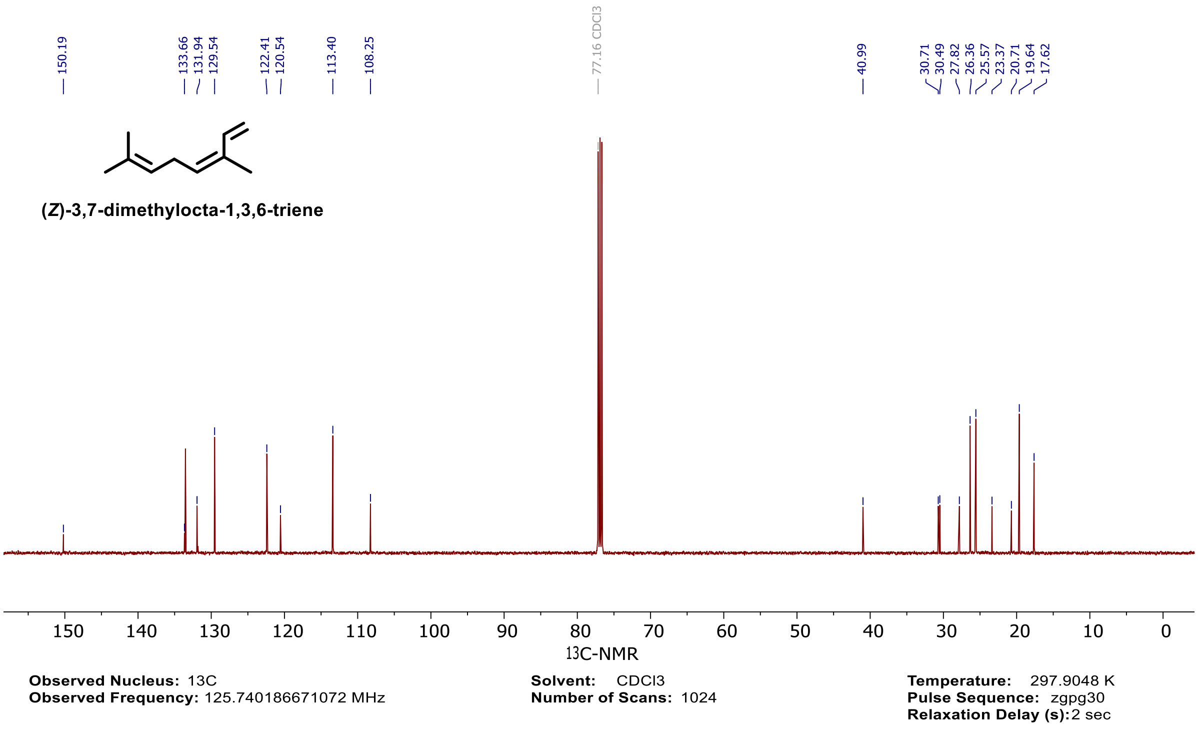

6.14 ^{1}H-NMR: Reference-geraniol

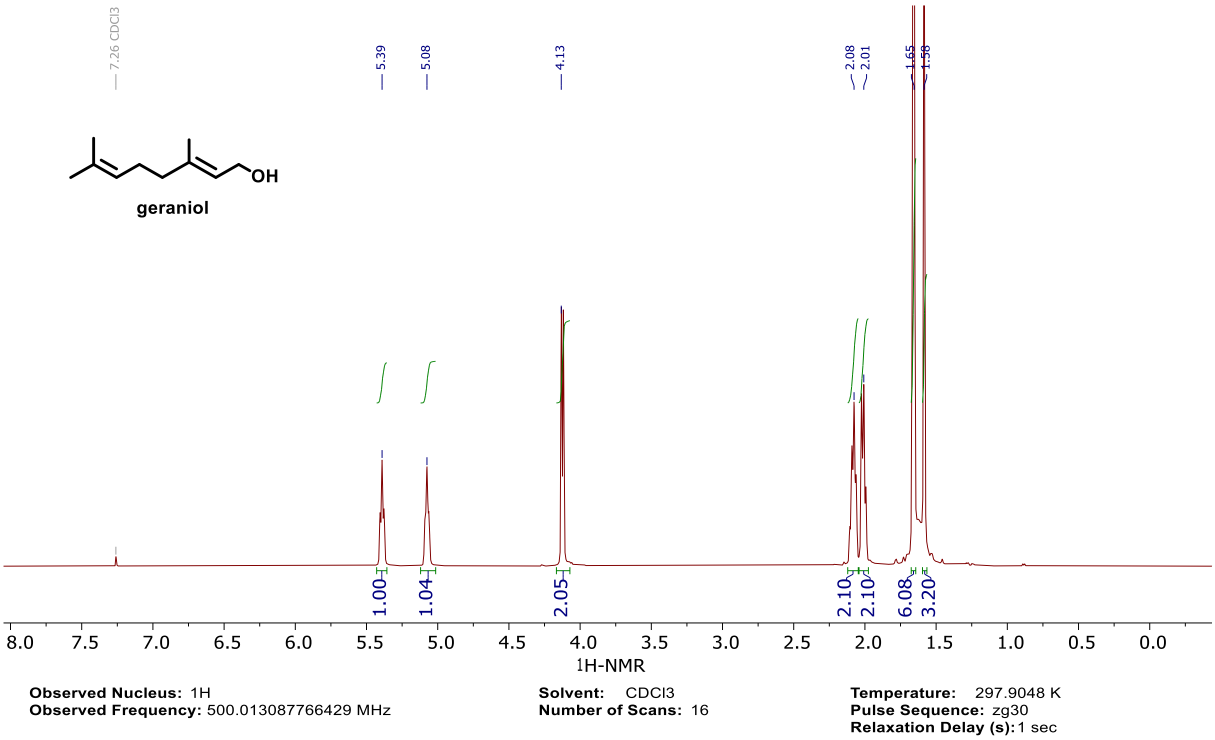

6.15 ^{1}H-NMR: Reference-(*E*)-3-methyl-5-phenylpent-2-en-1-ol

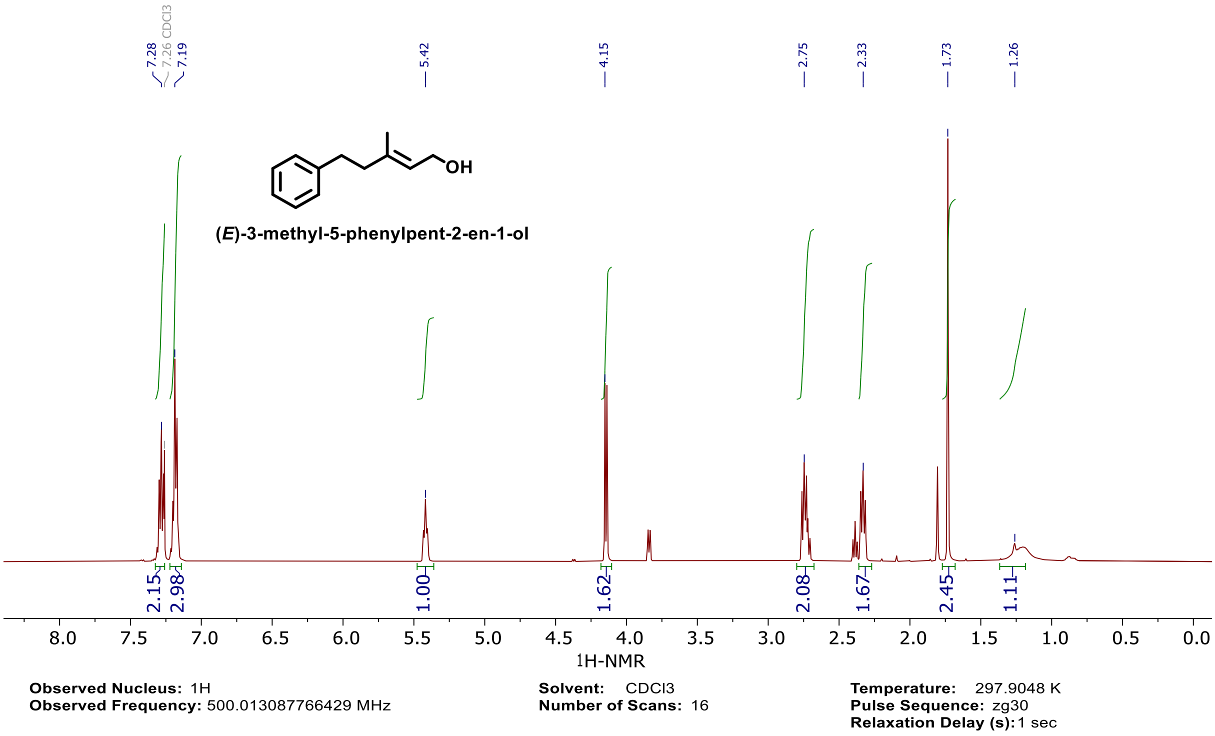

6.16 ^{1}H-NMR: Reference-4-(4-(trifluoromethyl)phenyl)butan-2-one

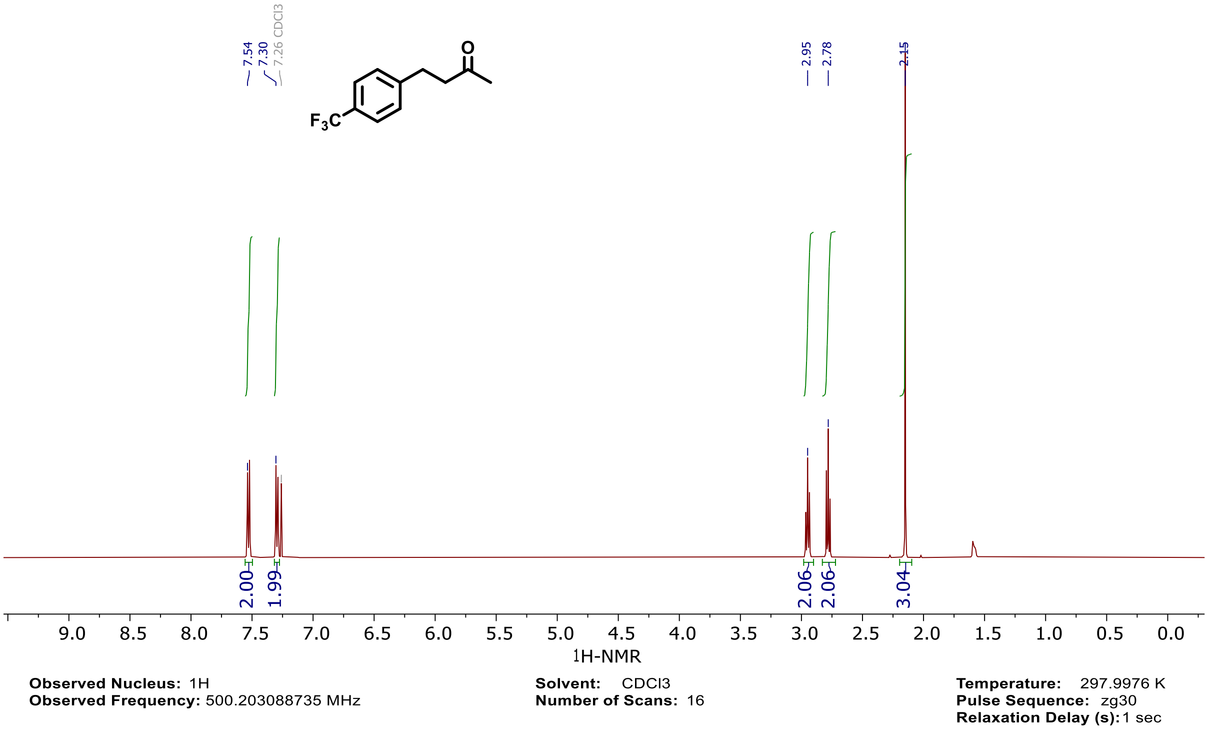

6.17 ^{1}H-NMR: *rac*-1c

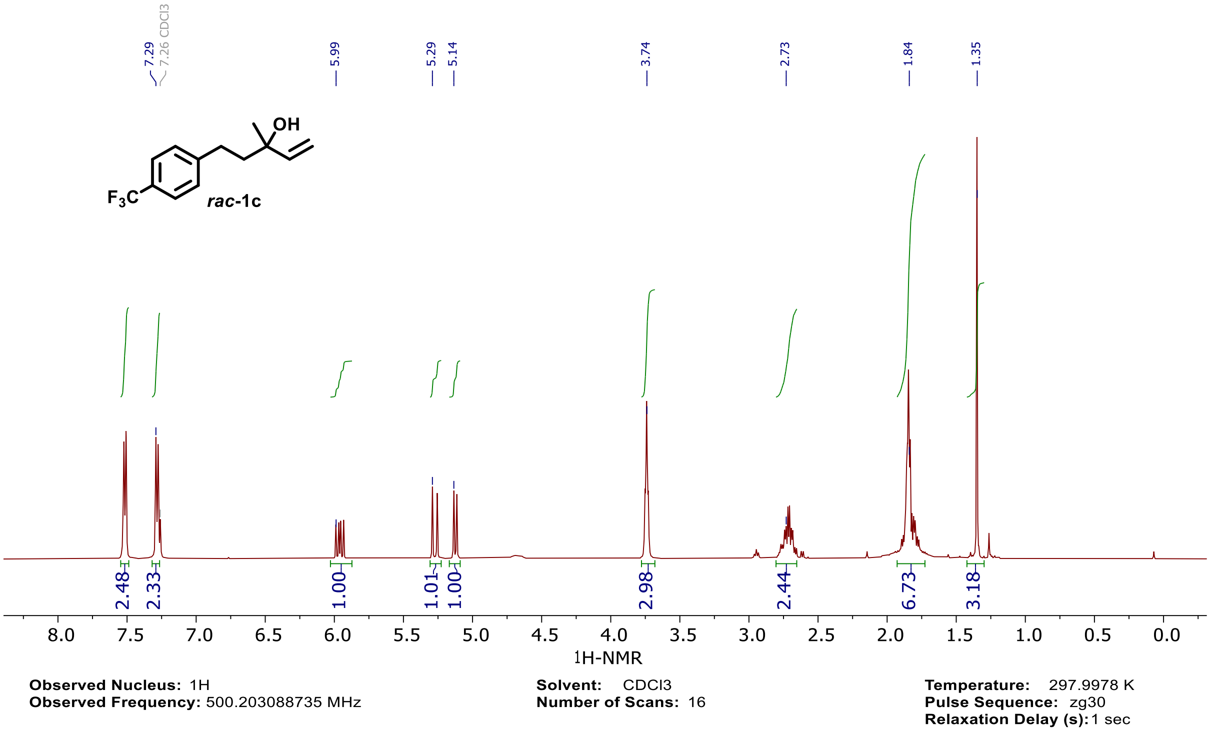

6.18 ^{1}H-NMR: Dehydration of *rac*-1c

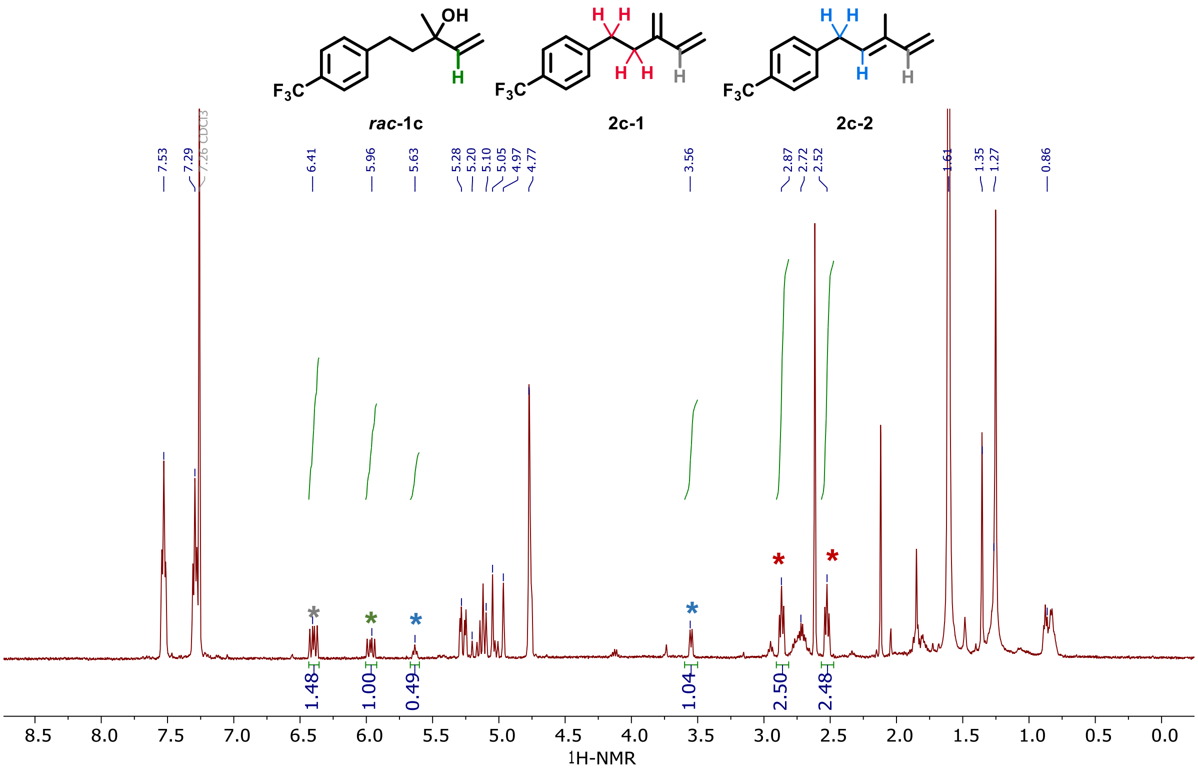

Observed Nucleus: 1H
Observed Frequency: 500.203088735 MHz
Solvent: CDCl3
Number of Scans: 64
Temperature: 298.0012 K
Pulse Sequence: zg30
Relaxation Delay (s): 1 sec

6.19 ^{1}H-NMR: Reference-4-(4-fluorophenyl)butan-2-one

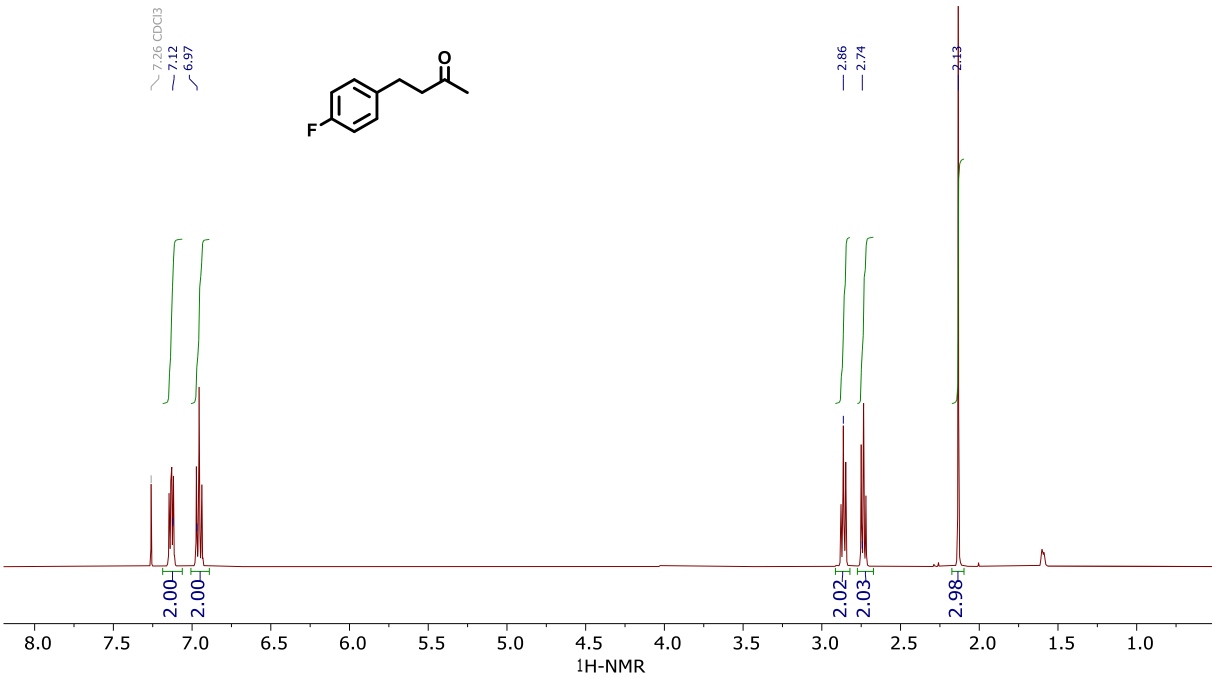

Observed Nucleus: 1H
Observed Frequency: 500.203088735 MHz
Solvent: CDCl3
Number of Scans: 16
Temperature: 297.9963 K
Pulse Sequence: zg30
Relaxation Delay (s): 1 sec

6.20 ^{1}H-NMR: *rac*-1d

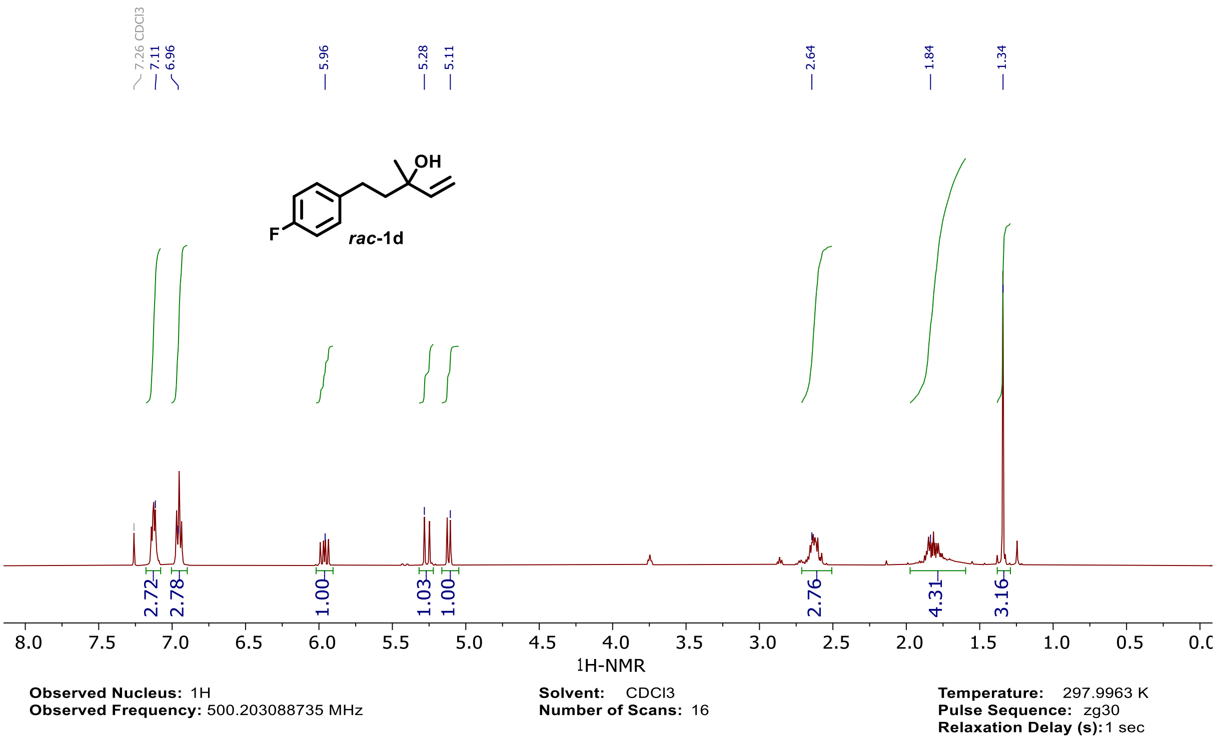

6.21 ^{1}H-NMR:Dehydration of *rac*-1d

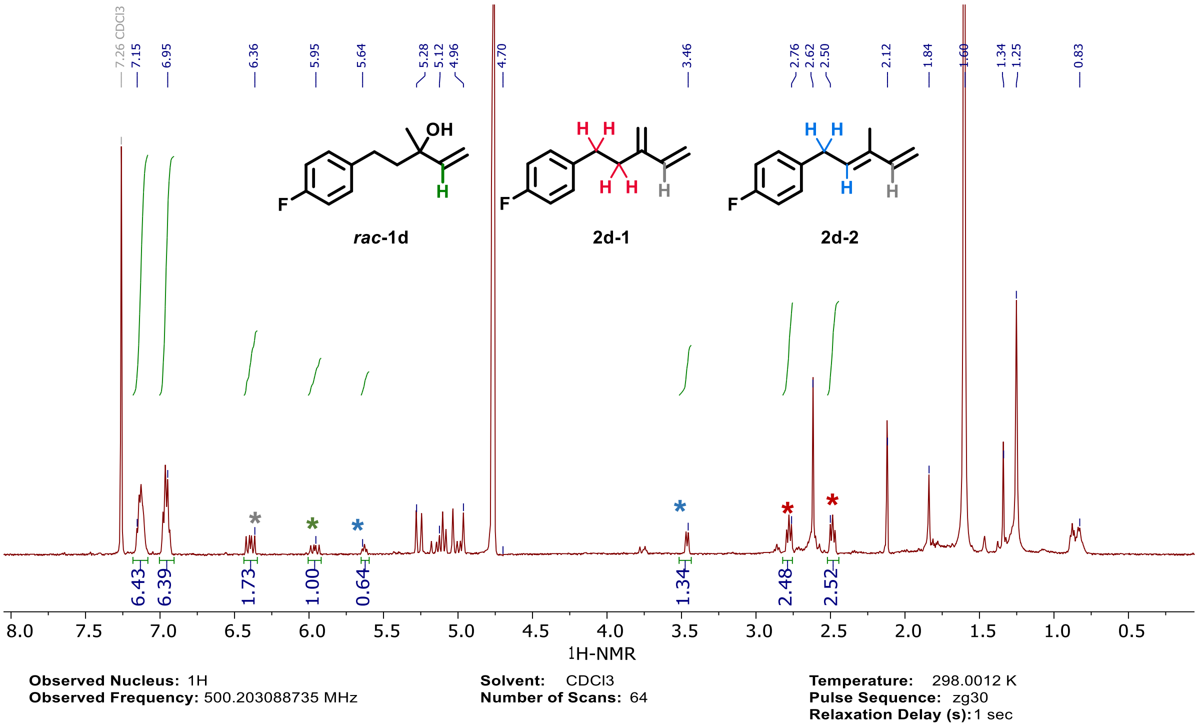

6.22 ^{1}H-NMR: Reference-4-(4-clorophenyl) butan-2-one

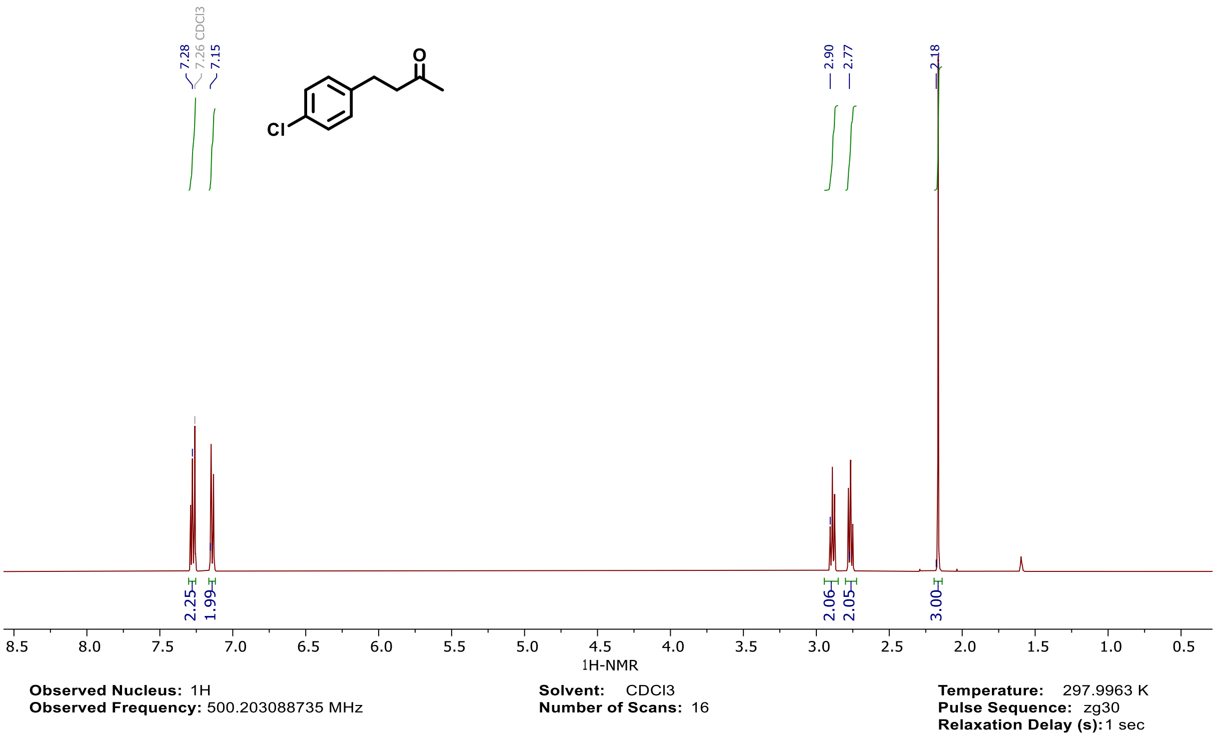

6.23 ^{1}H-NMR: Synthesis of (5-(4-chlorophenyl)-3-methylpent-1-en-3-ol (*rac*-1e)

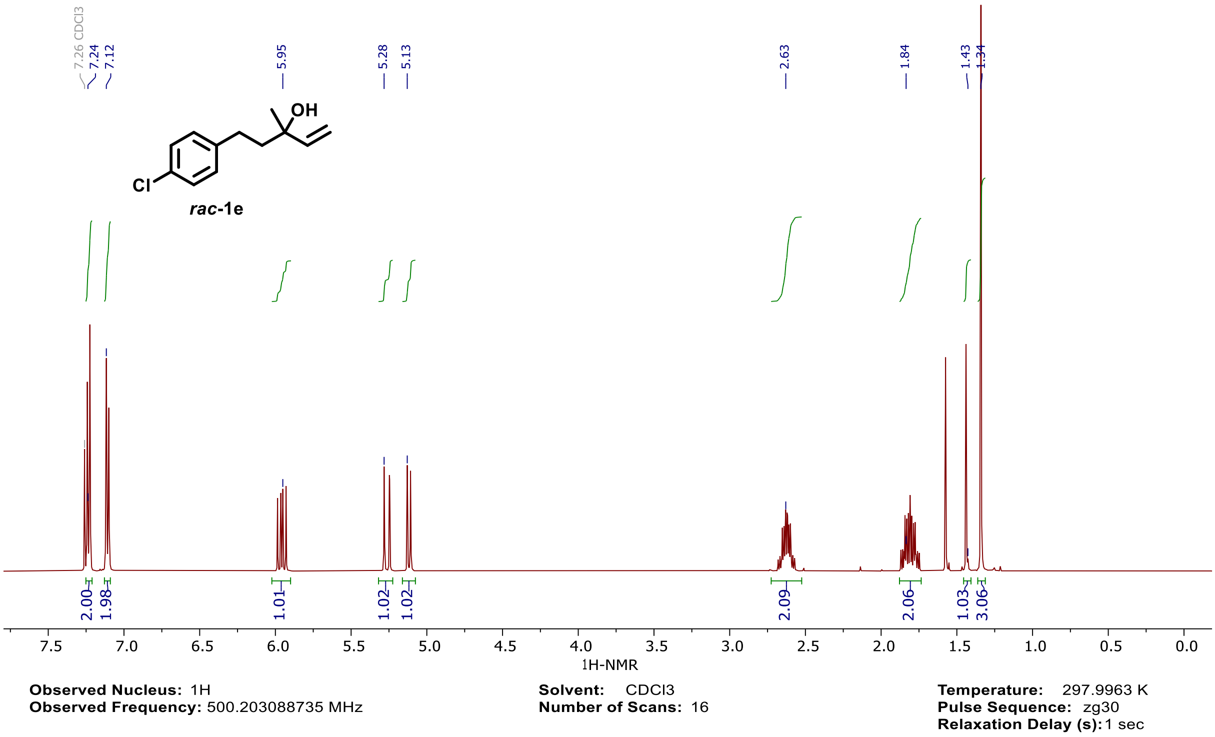

6.24 ^{1}H-NMR: Dehydration of *rac*-1e

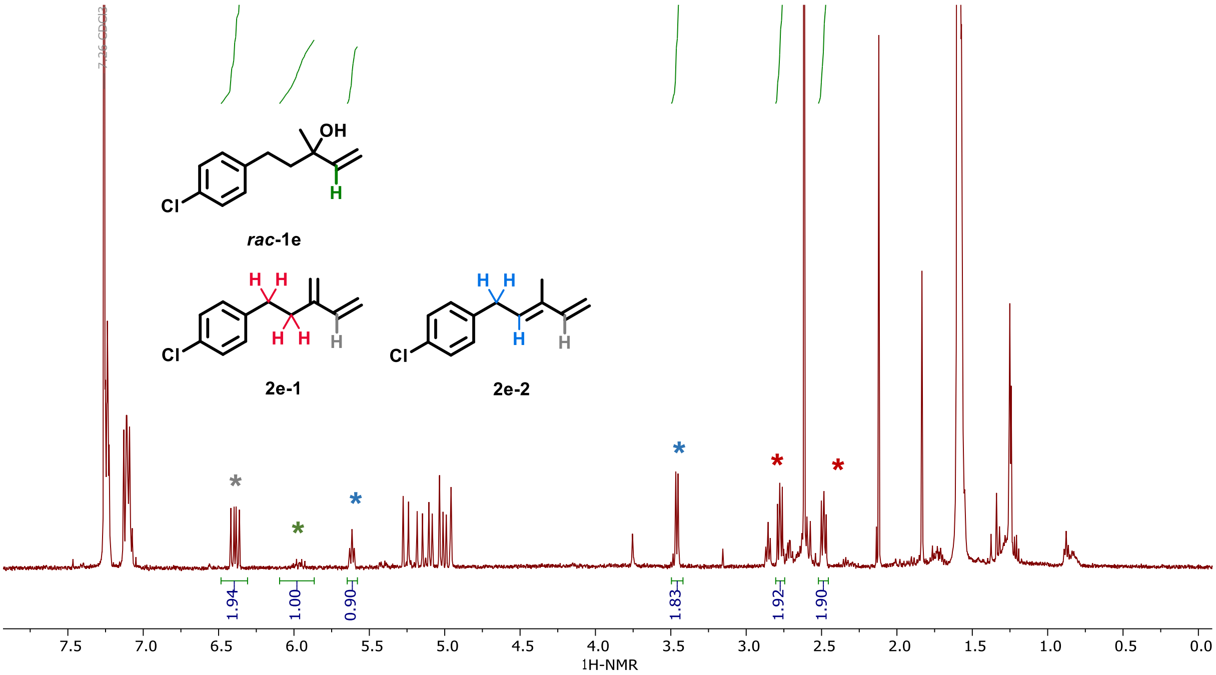

Observed Nucleus: 1H
Observed Frequency: 500.203088735 MHz
Solvent: CDCl3
Number of Scans: 64
Temperature: 298.0012 K
Pulse Sequence: zg30
Relaxation Delay (s): 1 sec

6.25 ^{1}H-NMR: Reference-4-(4-bromophenyl)butan-2-one

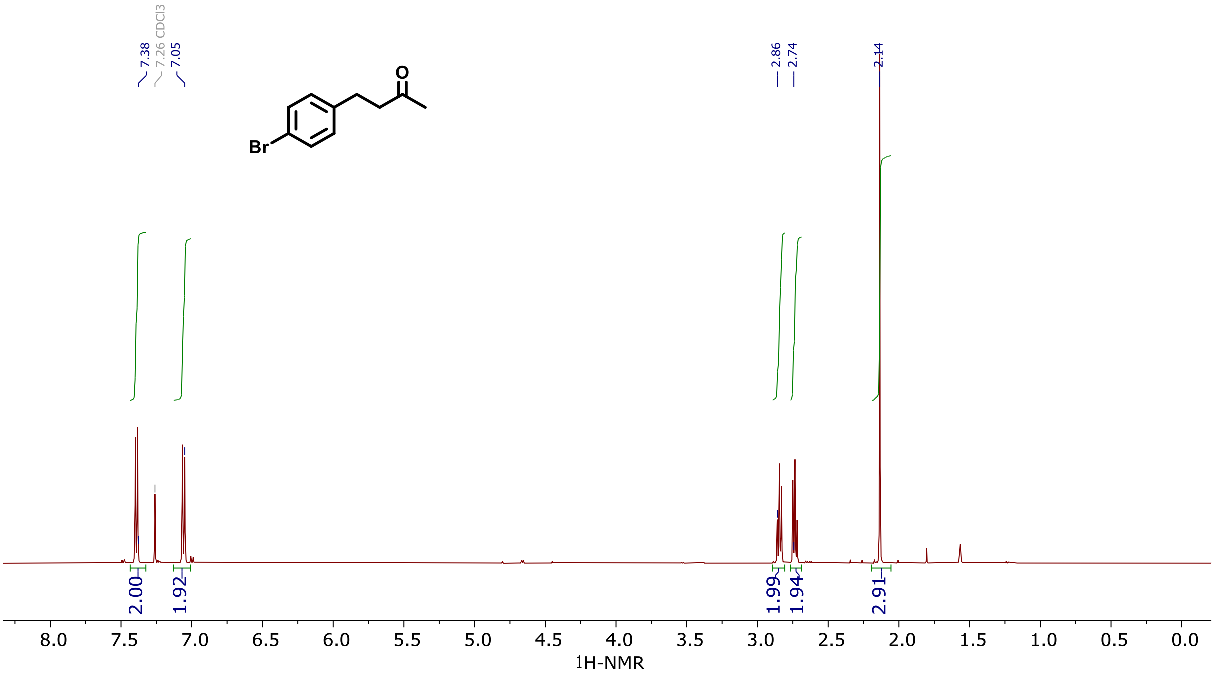

Observed Nucleus: 1H
Observed Frequency: 500.203088735 MHz
Solvent: CDCl3
Number of Scans: 16
Temperature: 298.0001 K
Pulse Sequence: zg30
Relaxation Delay (s): 1 sec

6.26 ^{1}H-NMR: *rac*-1f

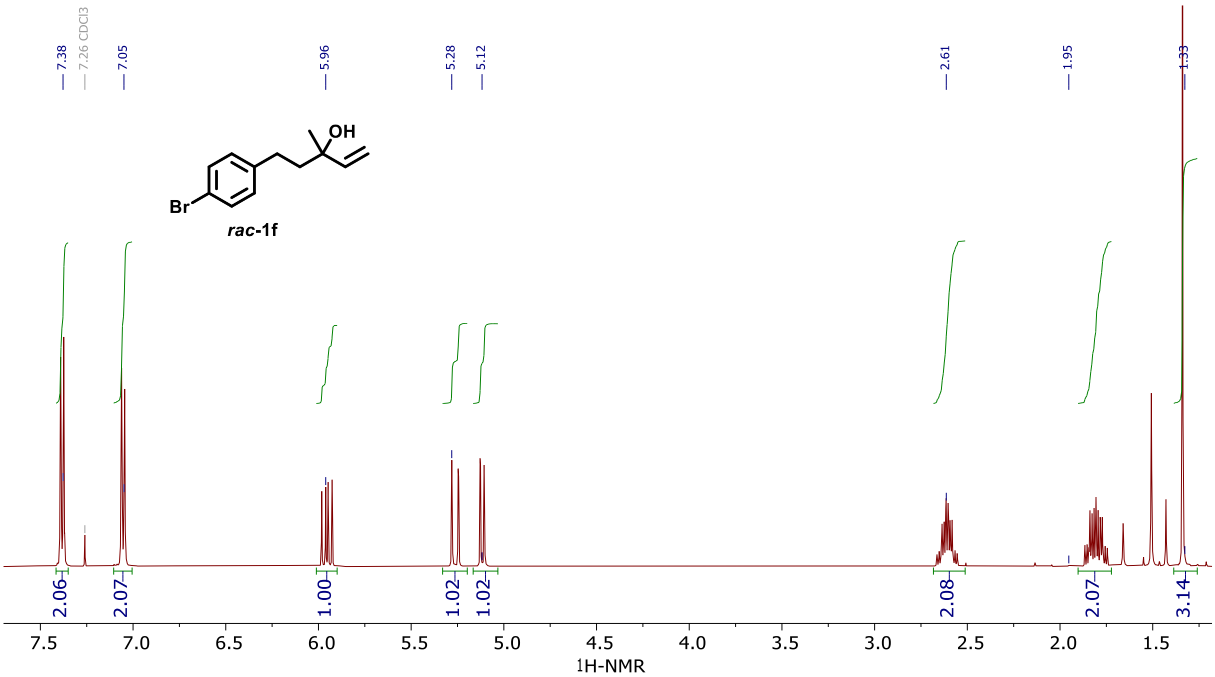

Observed Nucleus: 1H
Observed Frequency: 500.203088735 MHz
Solvent: CDCl3
Number of Scans: 16
Temperature: 298.0001 K
Pulse Sequence: zg30
Relaxation Delay (s): 1 sec

6.27 ^{1}H-NMR: Dehydration of *rac*-1f

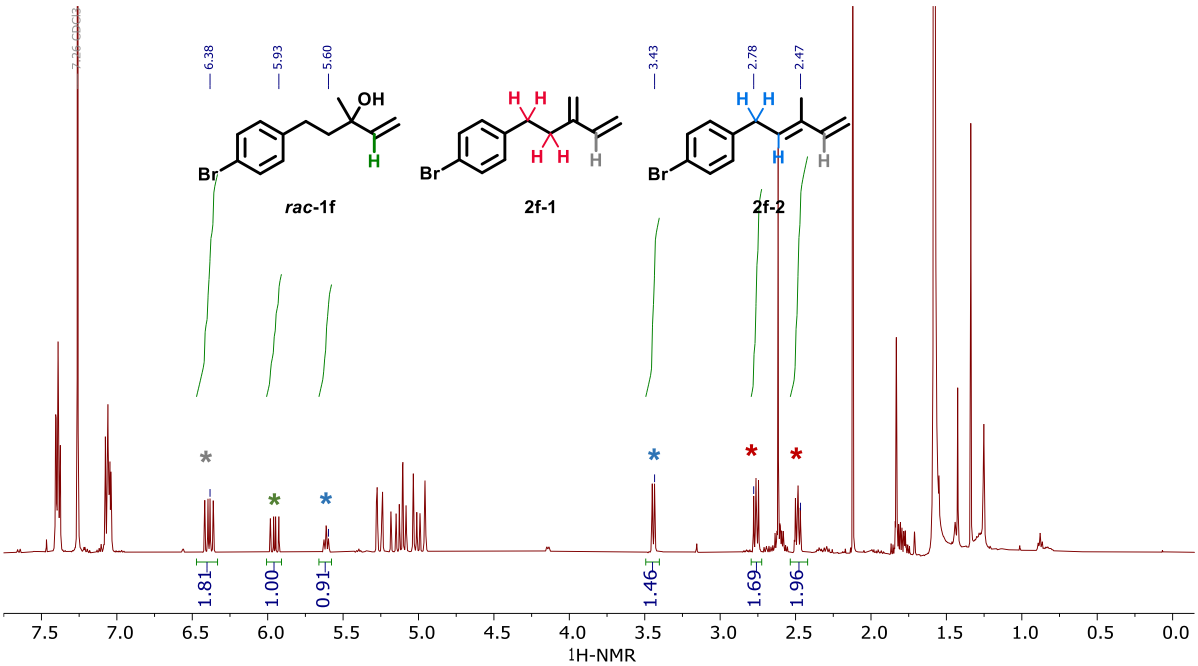

Observed Nucleus: 1H
Observed Frequency: 500.203088735 MHz
Solvent: CDCl3
Number of Scans: 64
Temperature: 298.0012 K
Pulse Sequence: zg30
Relaxation Delay (s): 1 sec

6.28 ¹H-NMR: Reference-4-(p-tolyl)butan-2-one

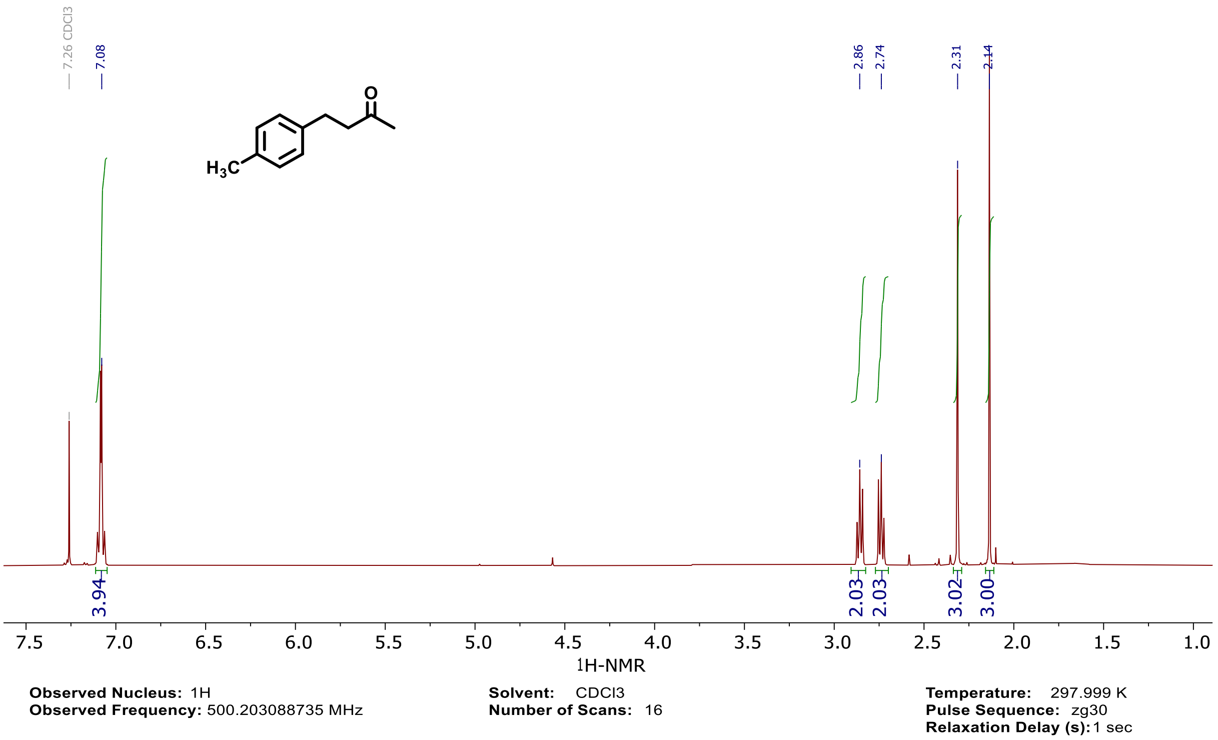

6.29 ¹H-NMR: *rac*-1g

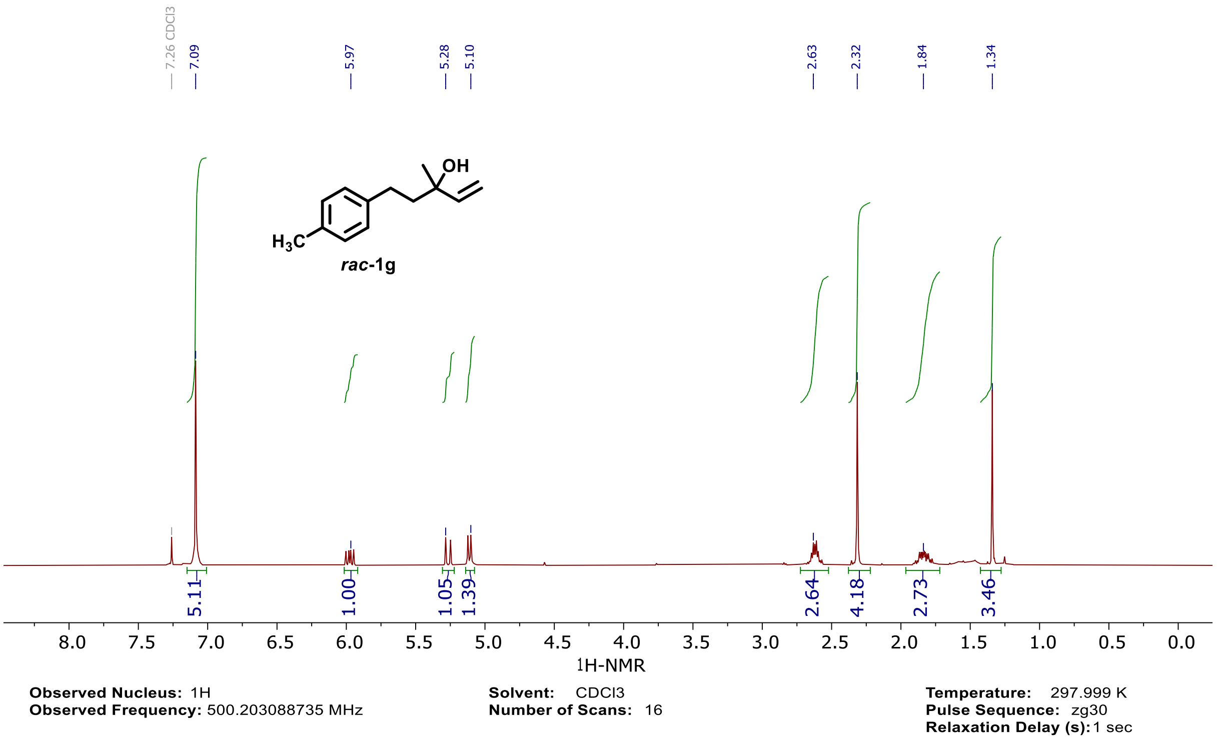

6.30 ^{1}H-NMR: Dehydration of *rac*-1g

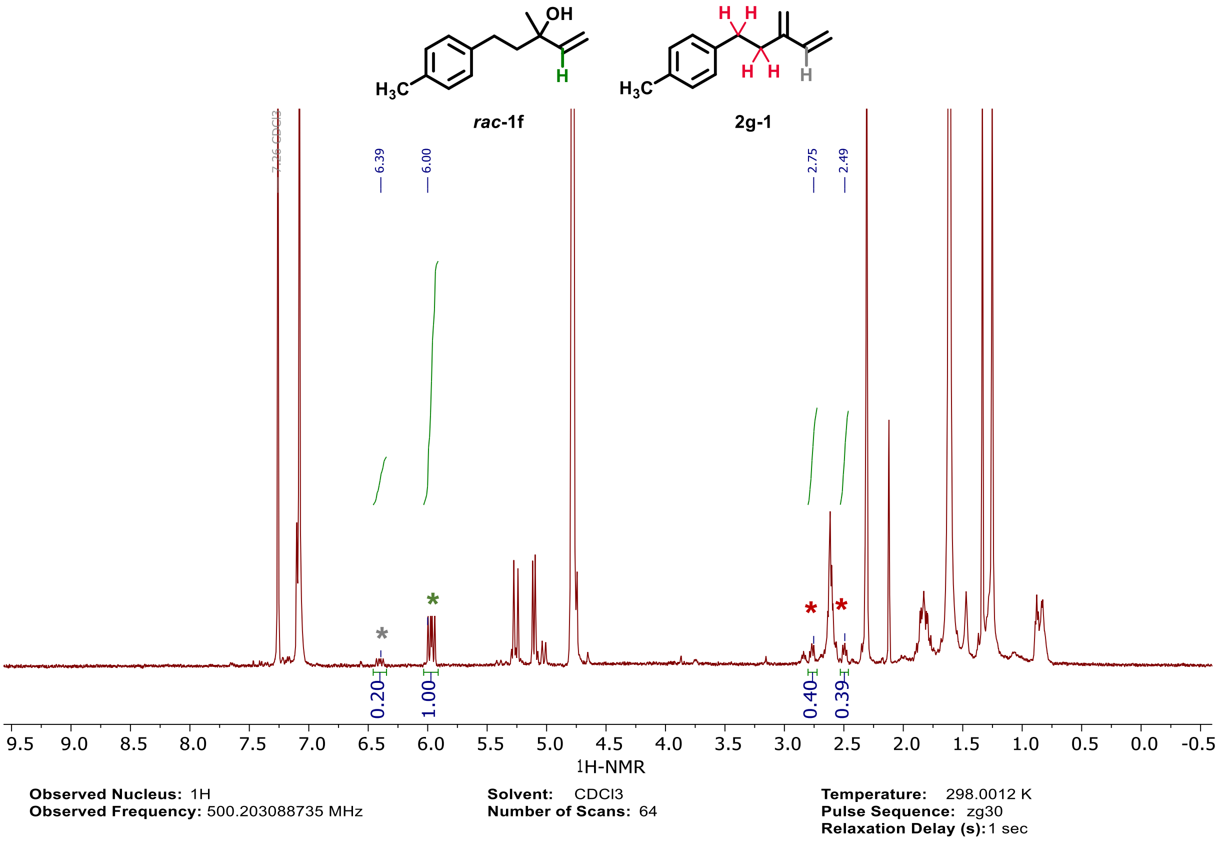

6.31 ^{1}H-NMR: Reference-4-(4-methoxyphenyl)butan-2-one

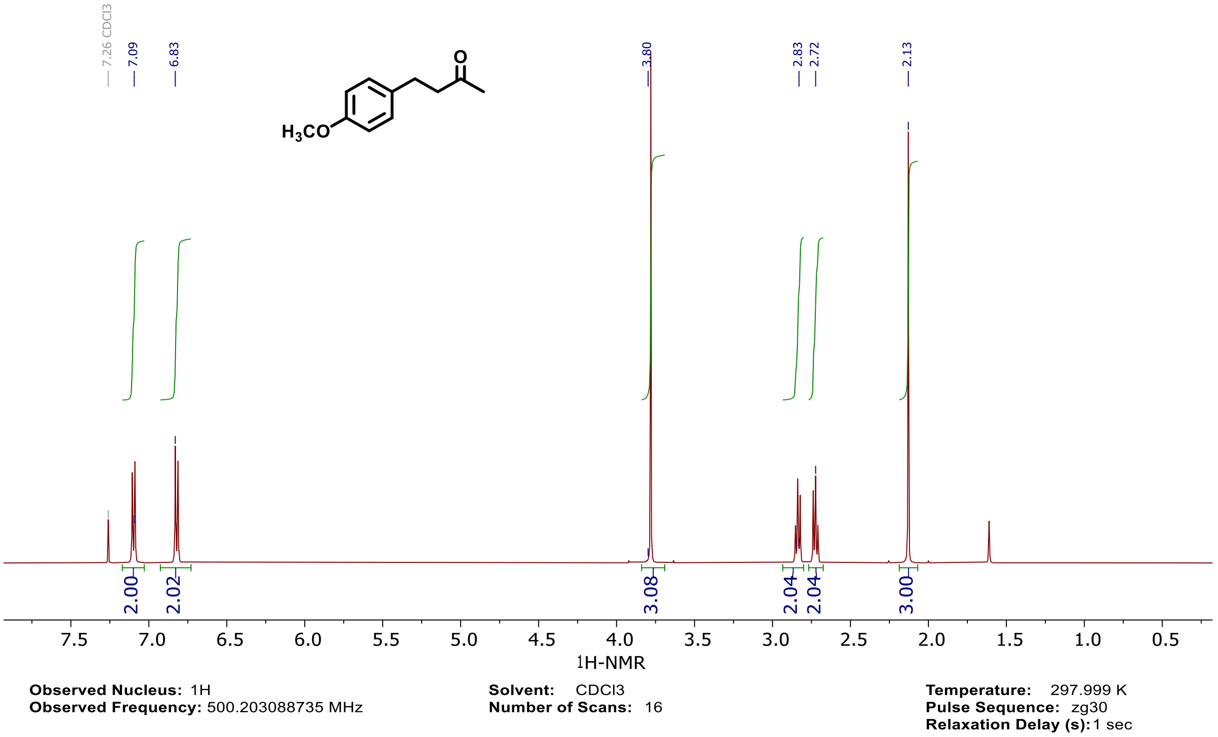

6.32 ^{1}H-NMR: *rac*-1h

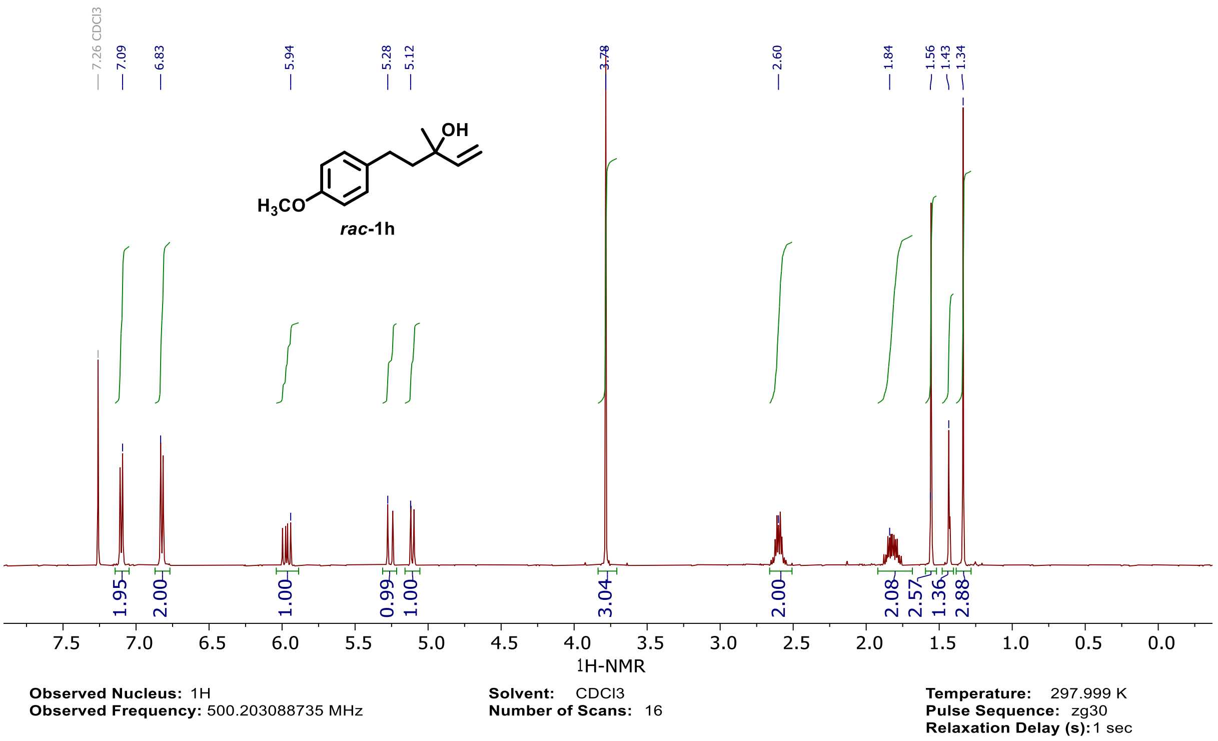

6.33 ^{1}H-NMR: Dehydration of *rac*-1h

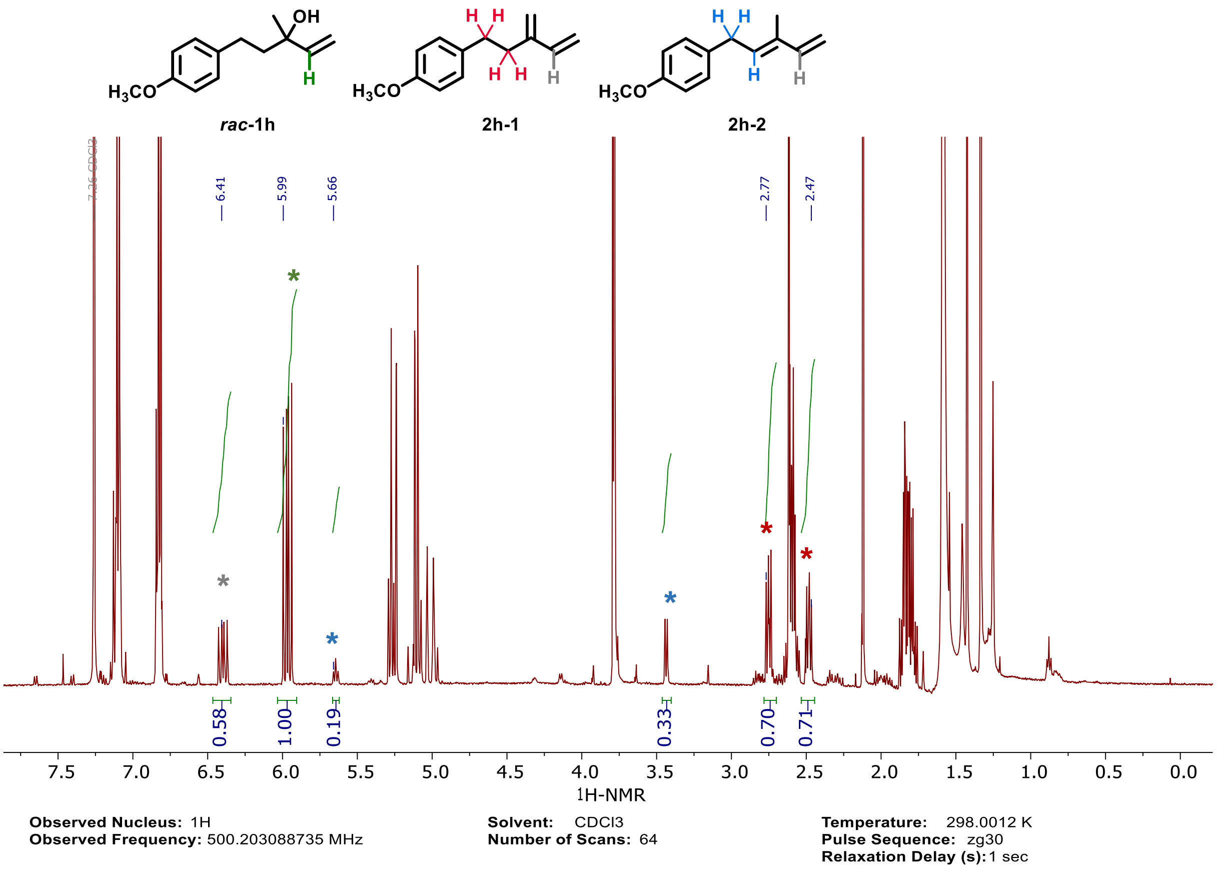

6.34 ^{1}H-NMR: Reference-4-(4-hydroxyphenyl)butan-2-one

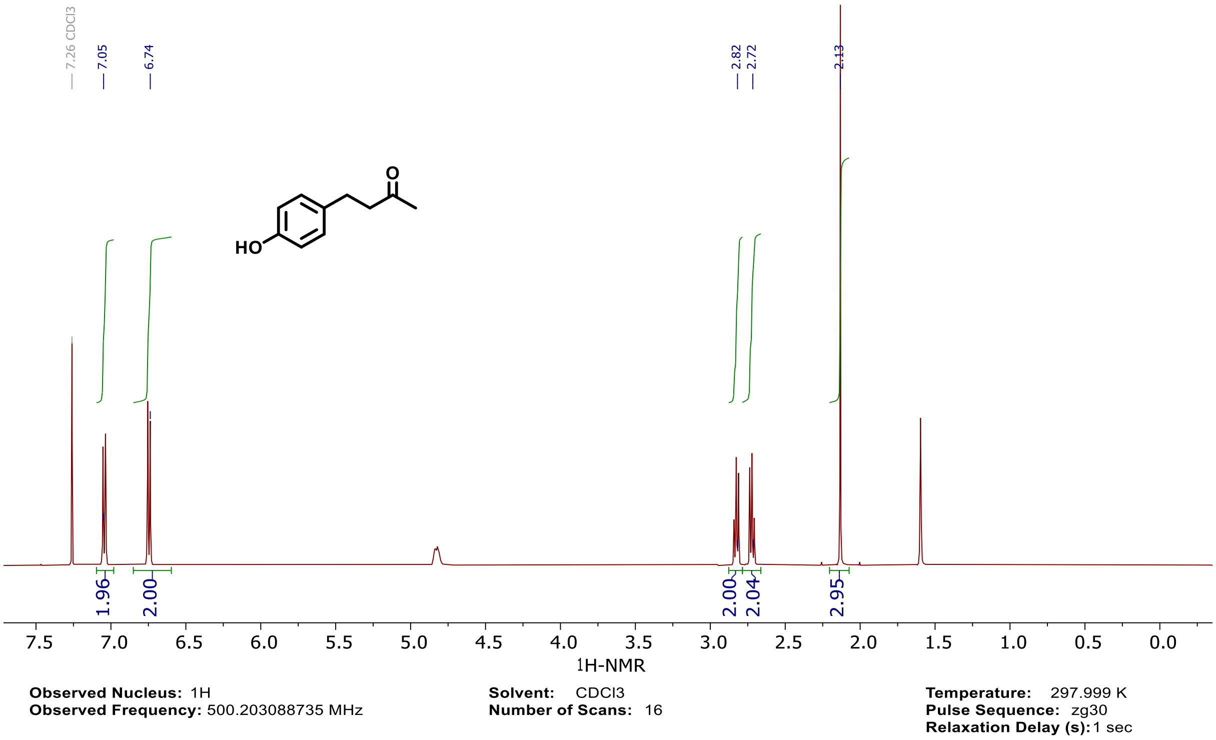

6.35 ^{1}H-NMR: *rac*-1i

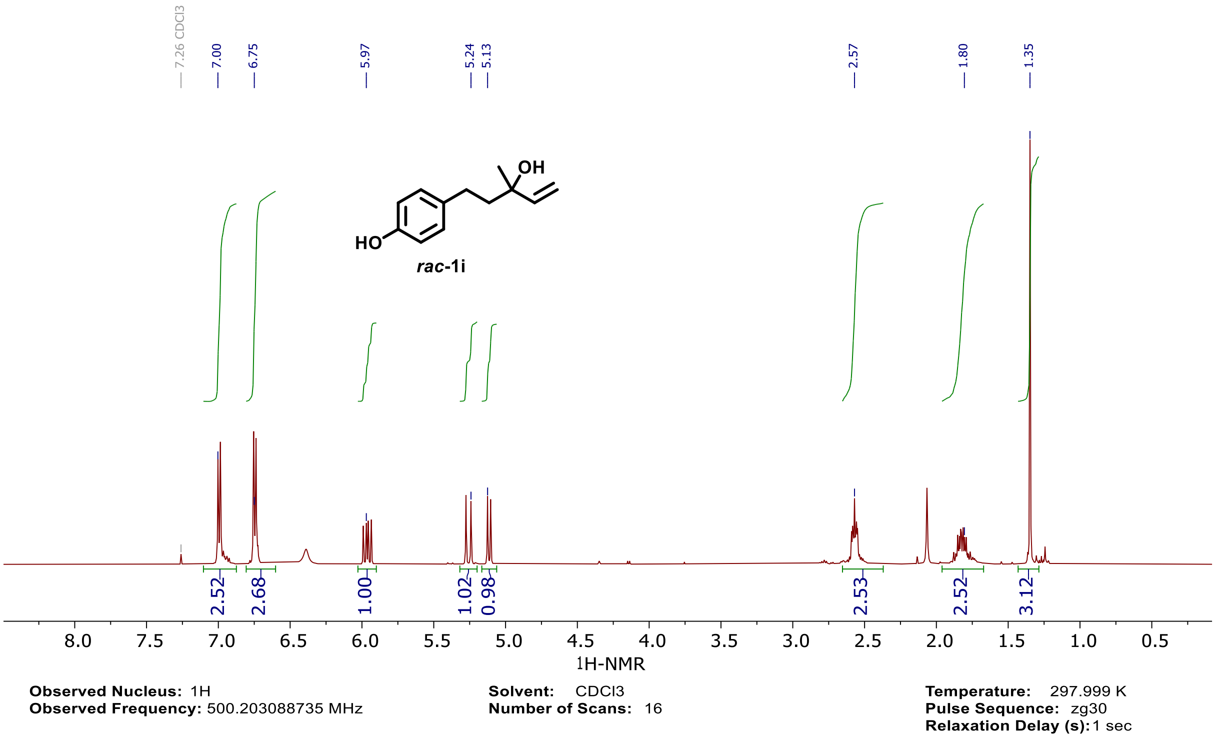

6.36 ^{1}H-NMR: Dehydration of *rac*-1i

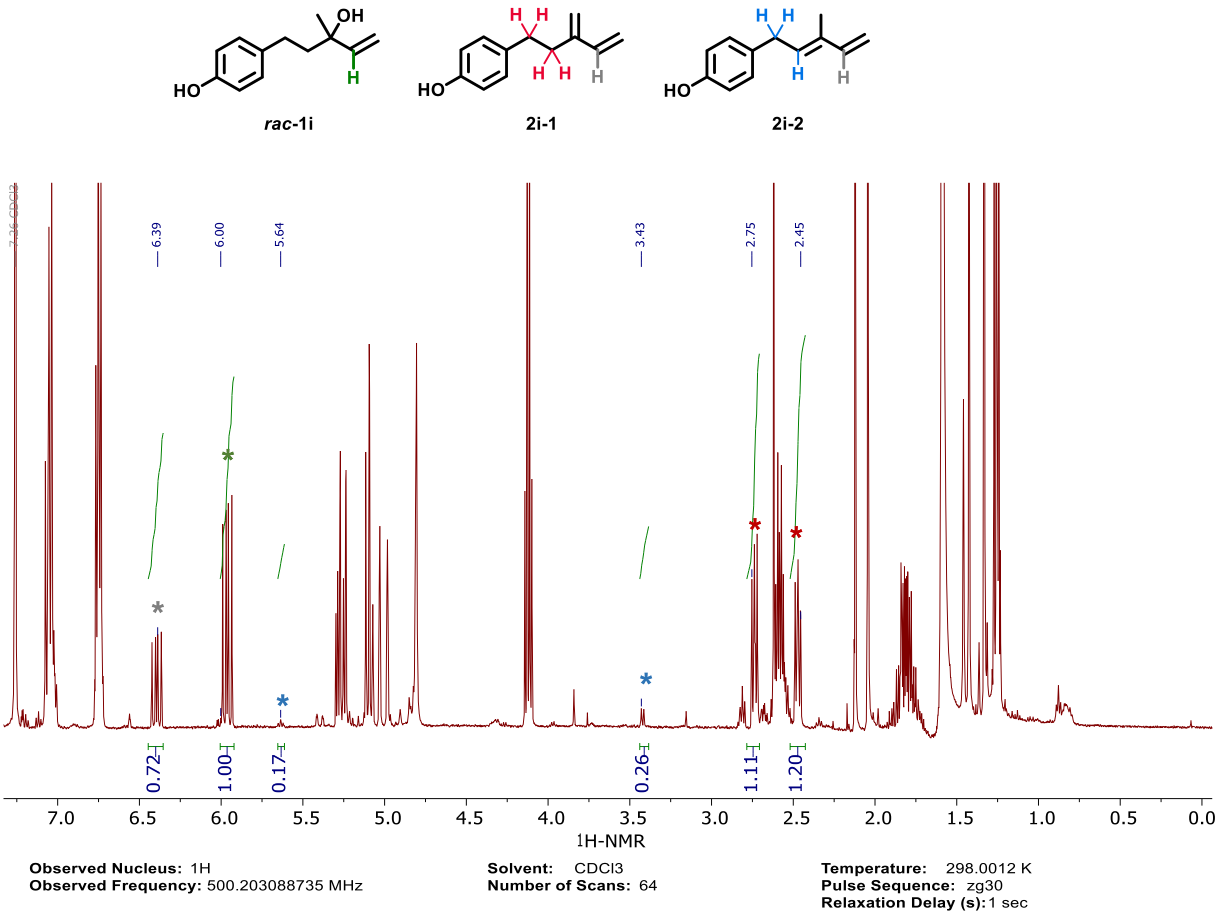

6.37 ^{1}H-NMR: Reference-acetophenone

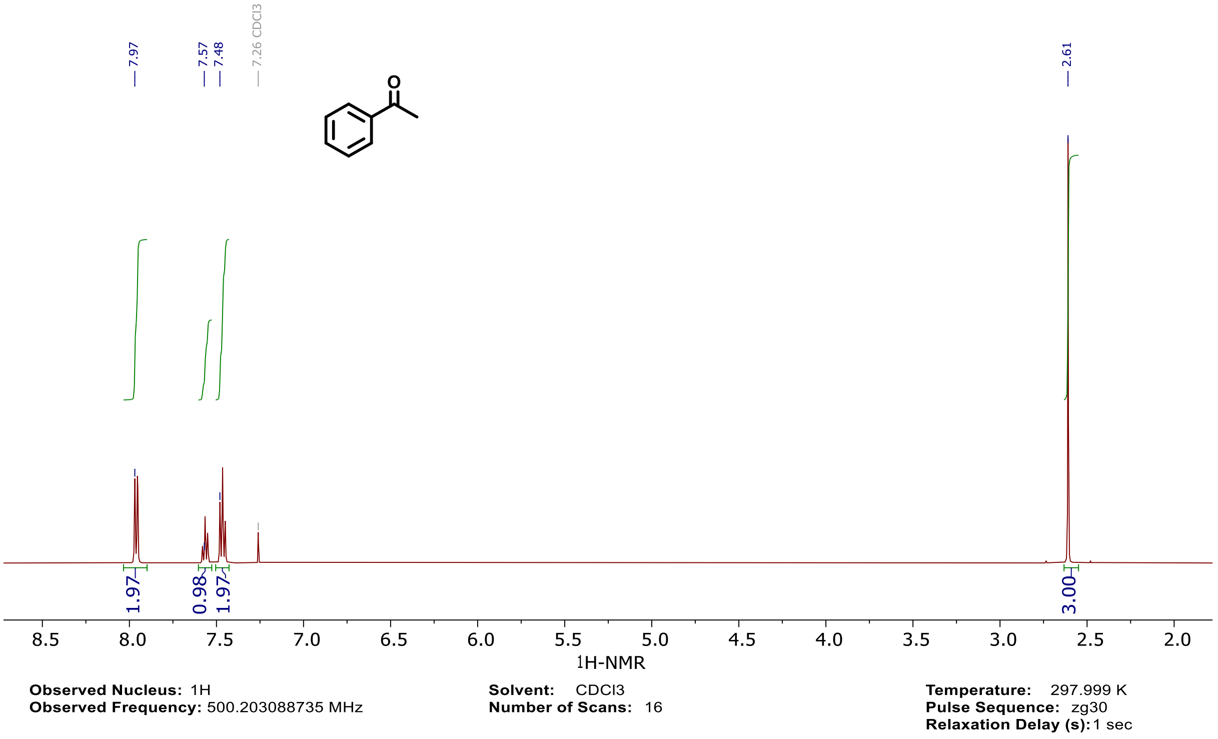

6.38 ^{1}H-NMR: *rac*-1j

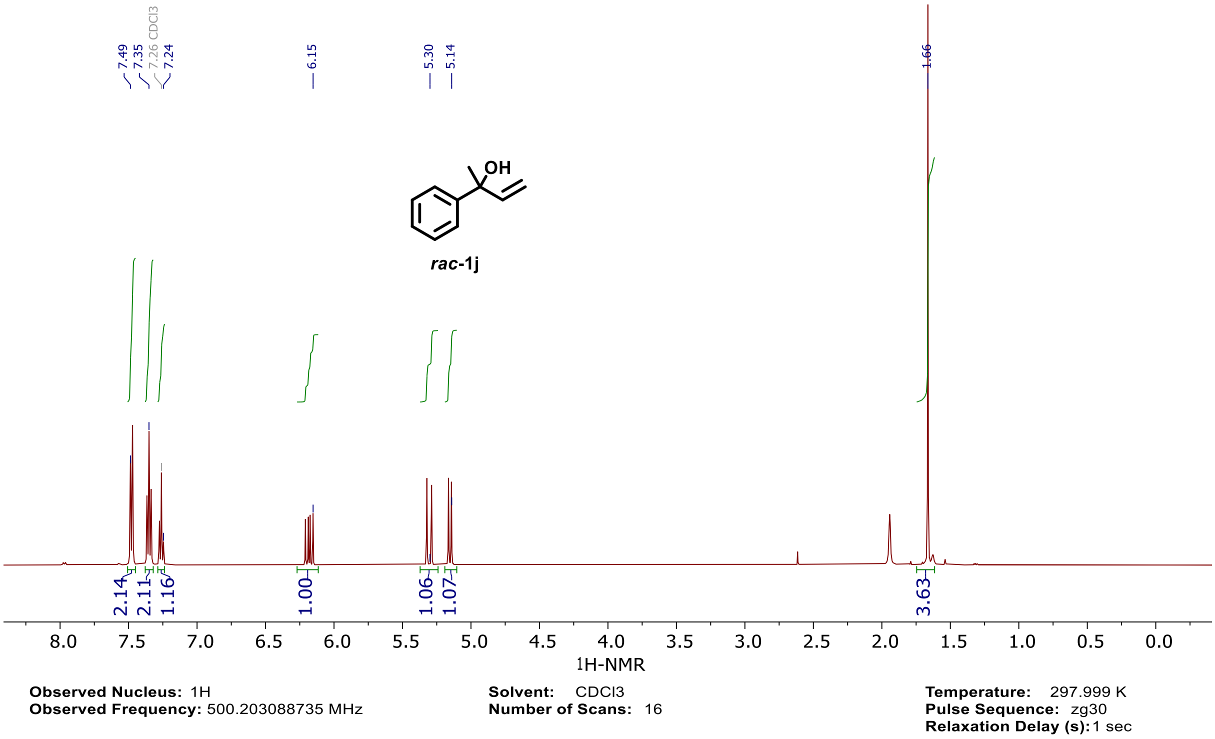

6.39 ^{1}H-NMR: Dehydration of *rac*-1j

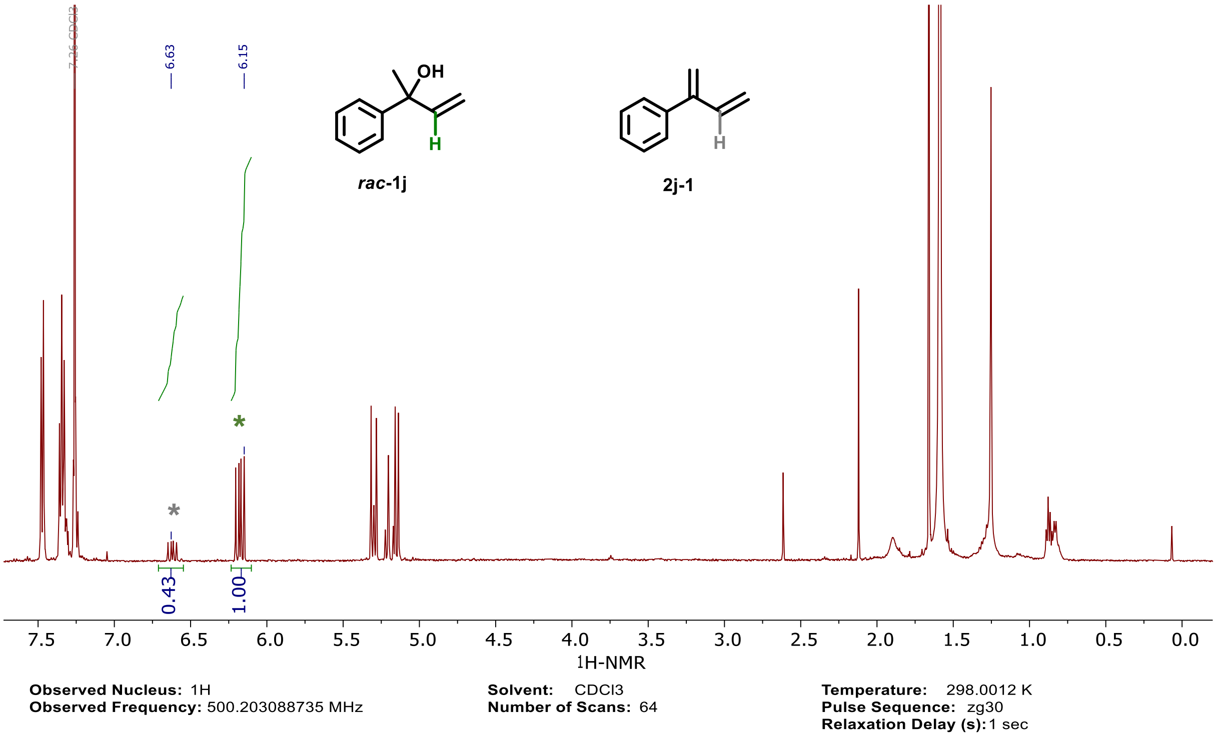

6.40 ^{1}H-NMR: Reference-1-cyclohexylethan-1-one

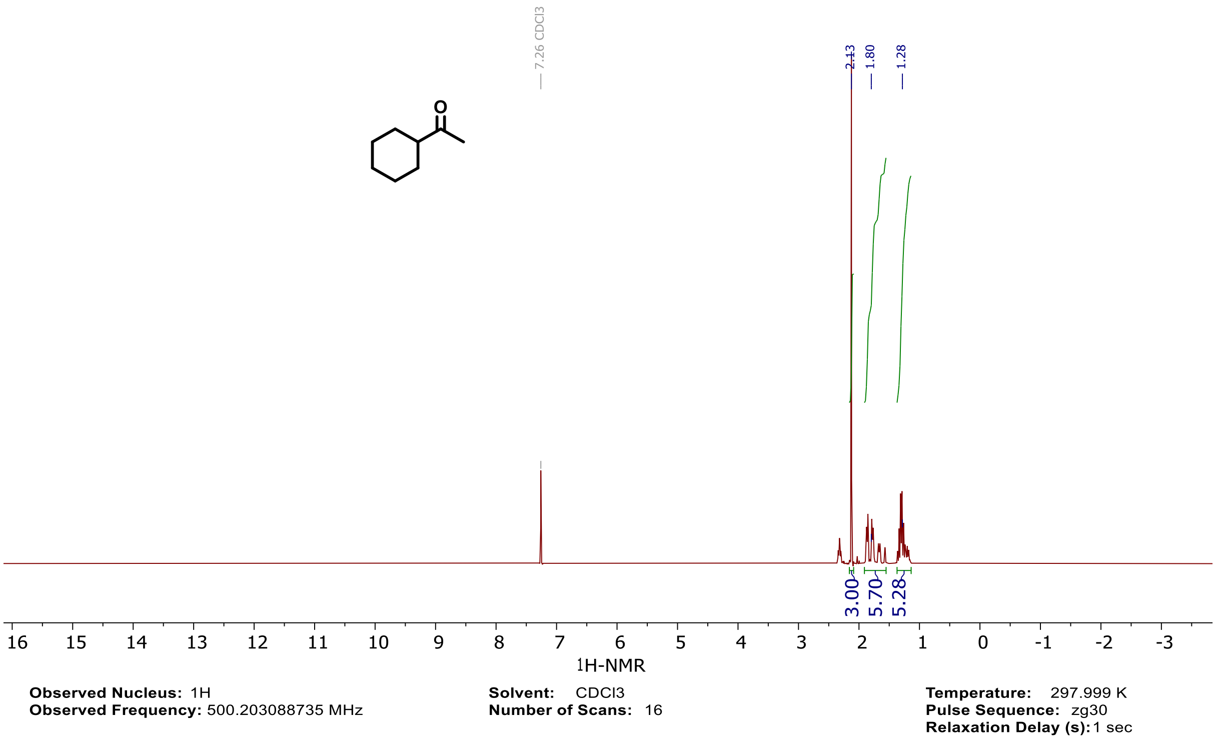

6.41 ^{1}H-NMR: *rac*-1k

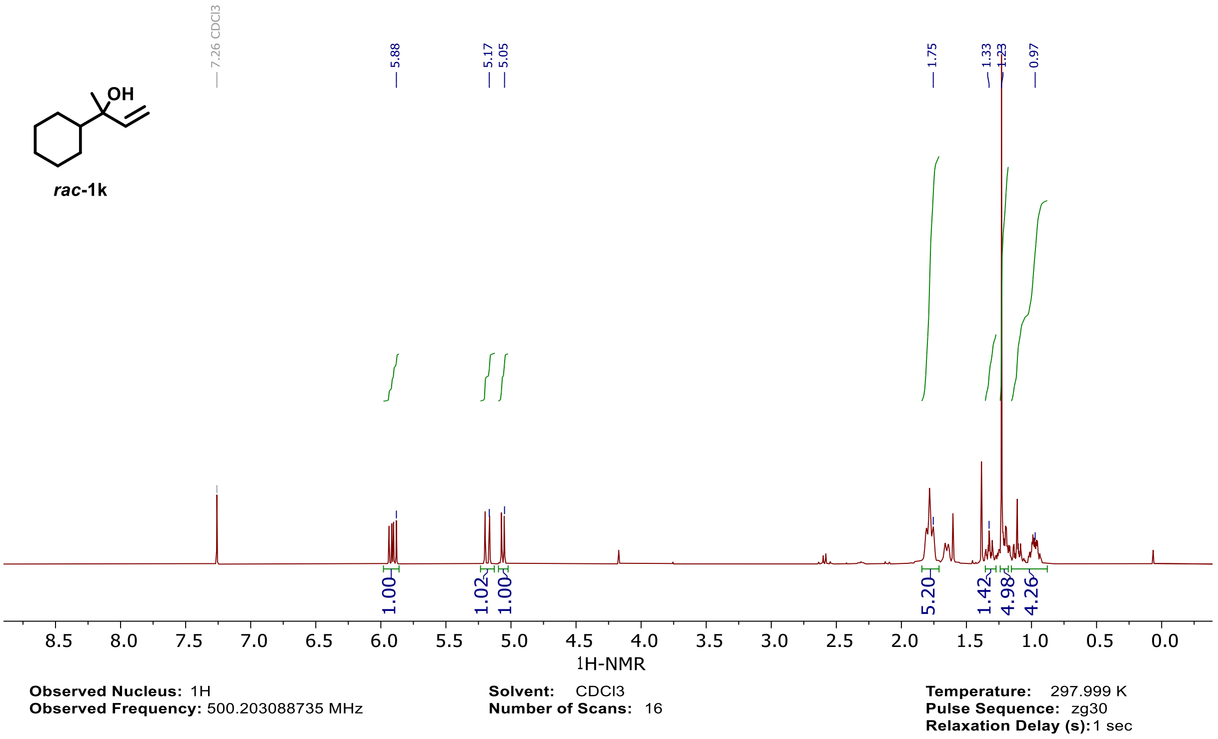

6.42 ^{1}H-NMR: Attempted reaction with *rac*-1k (No detectable product formation)

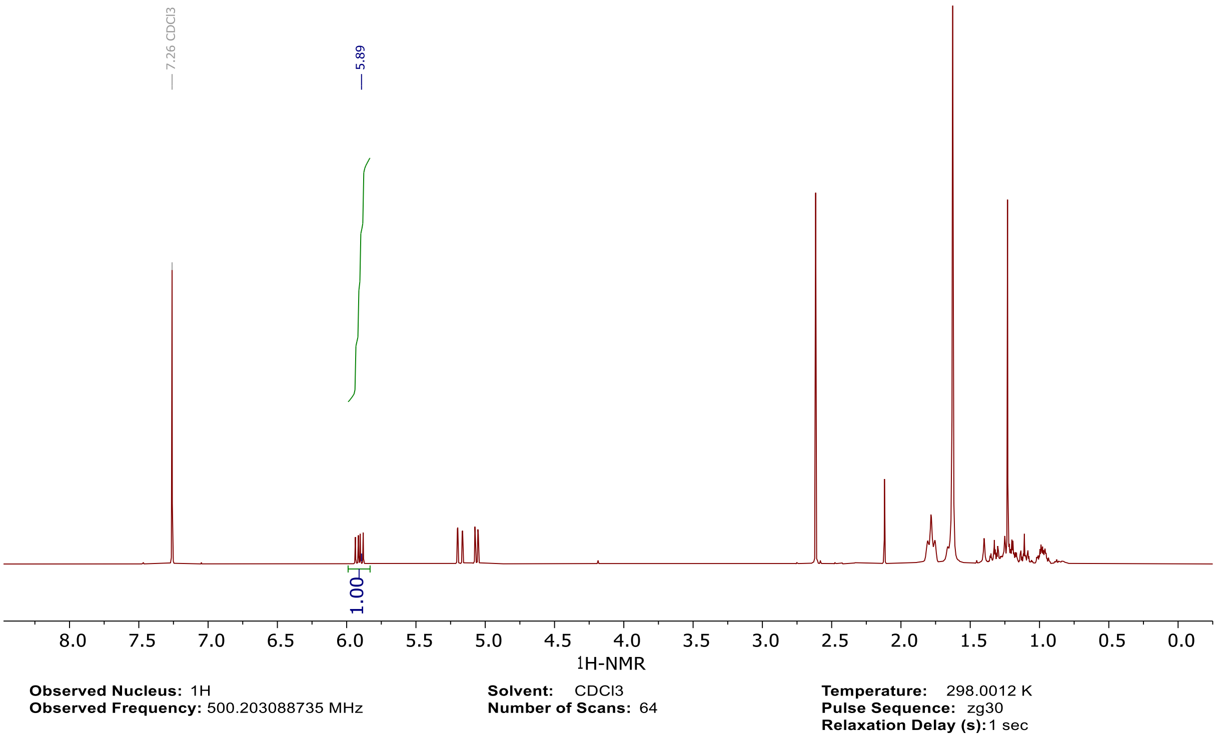

6.43 ^{1}H-NMR: Reference-4-cyclohexylbutan-2-one

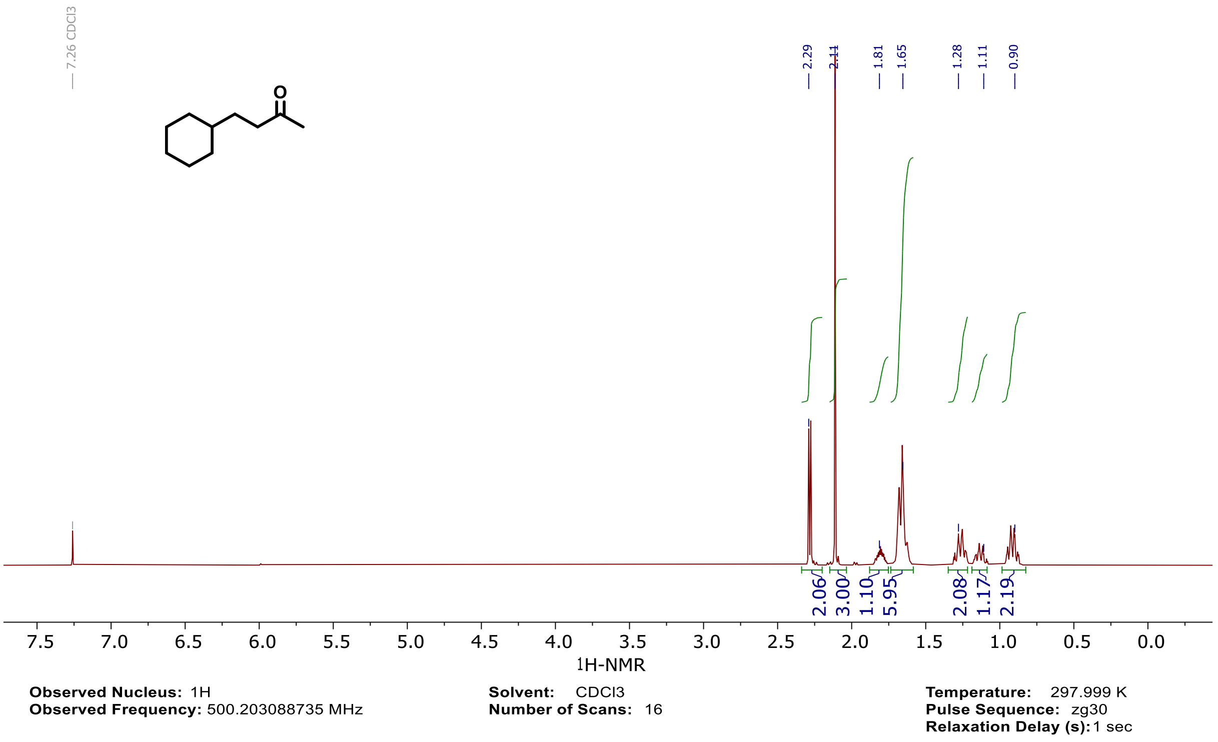

6.44 ^{1}H-NMR: *rac*-1l alcohol

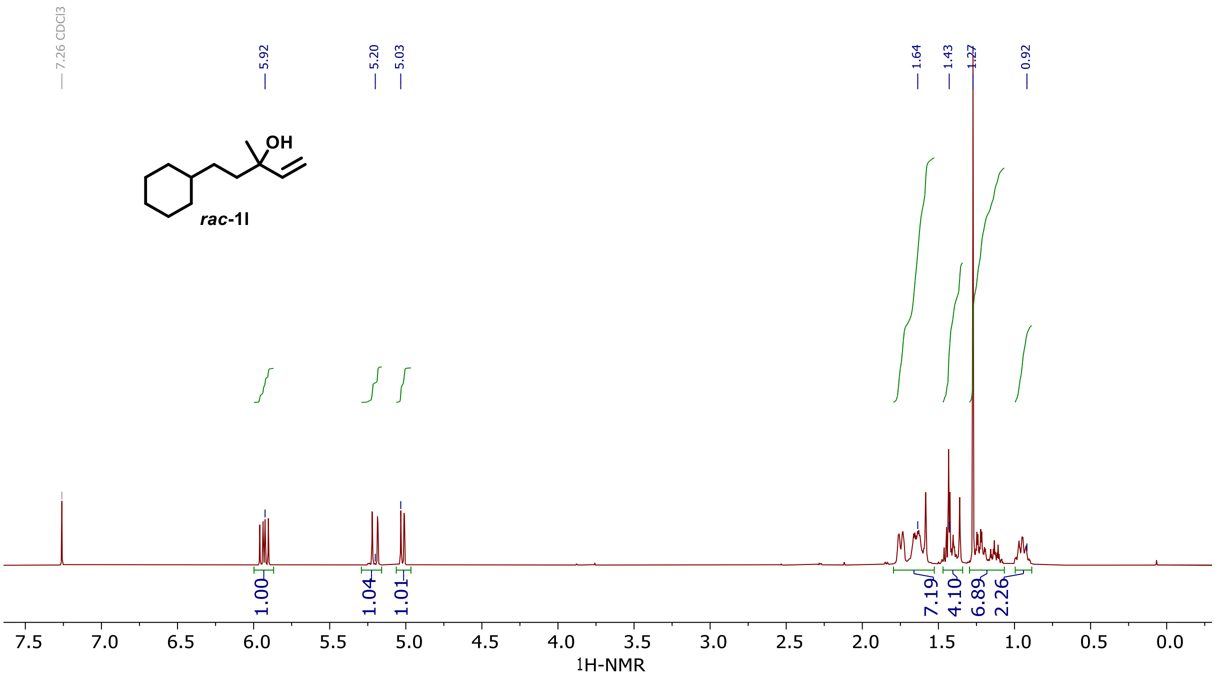

Observed Nucleus: 1H
Observed Frequency: 500.203088735 MHz
Solvent: CDCl3
Number of Scans: 16
Temperature: 297.999 K
Pulse Sequence: zg30
Relaxation Delay (s): 1 sec

6.45 ^{1}H-NMR: Dehydration with *rac*-1l

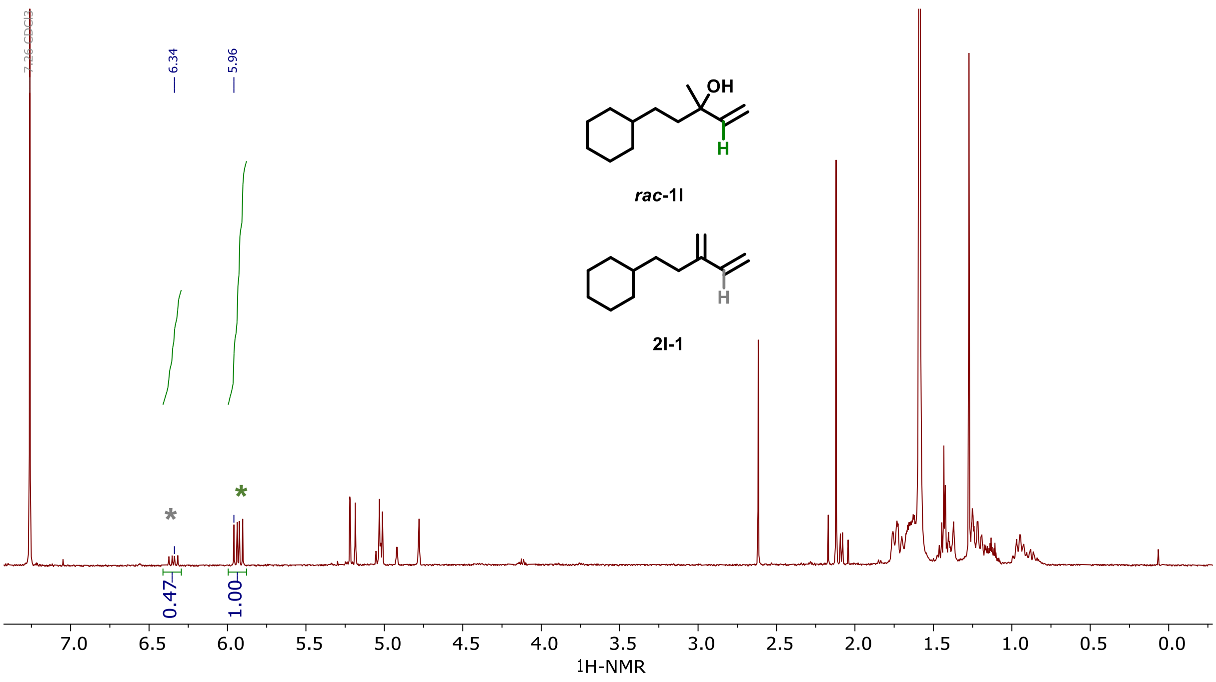

Observed Nucleus: 1H
Observed Frequency: 500.203088735 MHz
Solvent: CDCl3
Number of Scans: 64
Temperature: 298.0012 K
Pulse Sequence: zg30
Relaxation Delay (s): 1 sec

6.46 ^{1}H-NMR: Reference-4-cyclopentylbutan-2-one

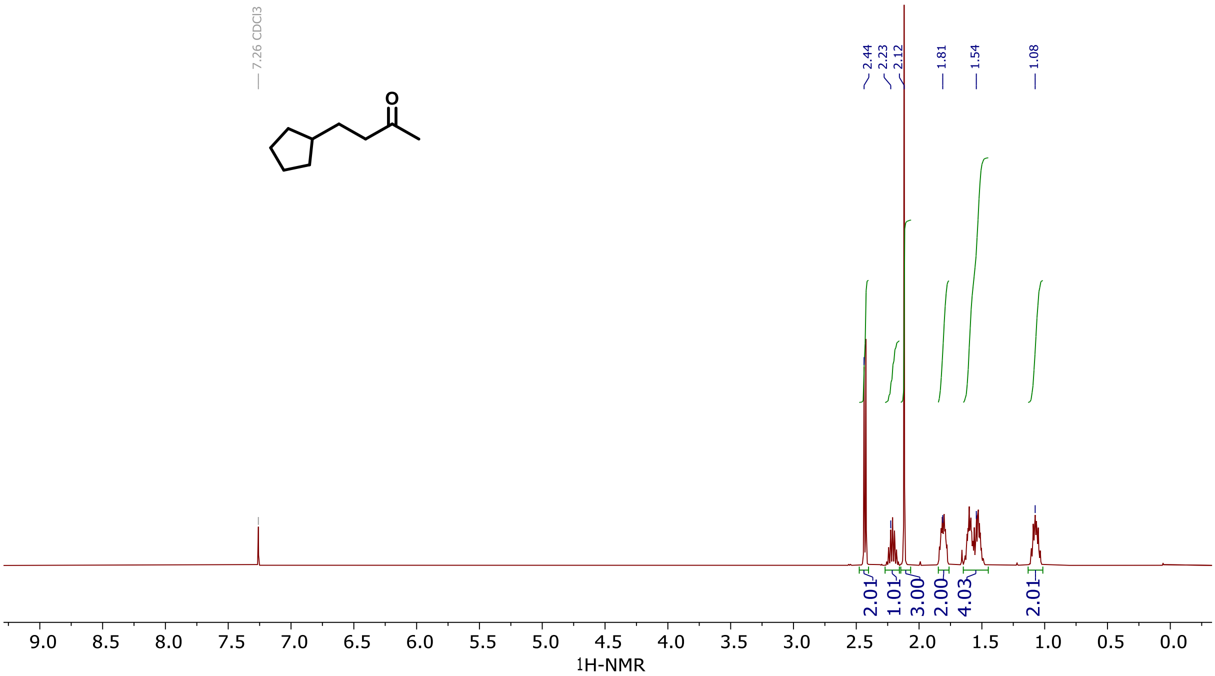

Observed Nucleus: 1H
Observed Frequency: 500.203088735 MHz
Solvent: CDCl3
Number of Scans: 16
Temperature: 297.999 K
Pulse Sequence: zg30
Relaxation Delay (s): 1 sec

6.47 ^{1}H-NMR: *rac*-1m

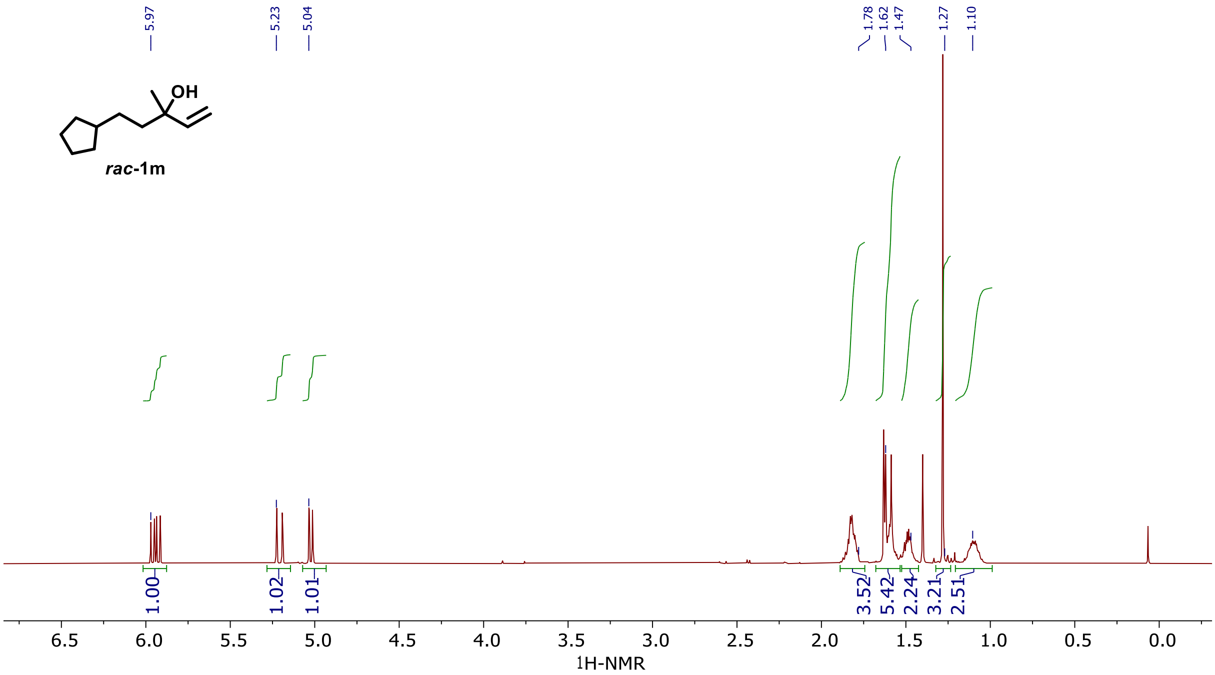

Observed Nucleus: 1H
Observed Frequency: 500.203088735 MHz
Solvent: CDCl3
Number of Scans: 16
Temperature: 297.999 K
Pulse Sequence: zg30
Relaxation Delay (s): 1 sec

6.48 ^{1}H-NMR: Dehydration with *rac*-1m

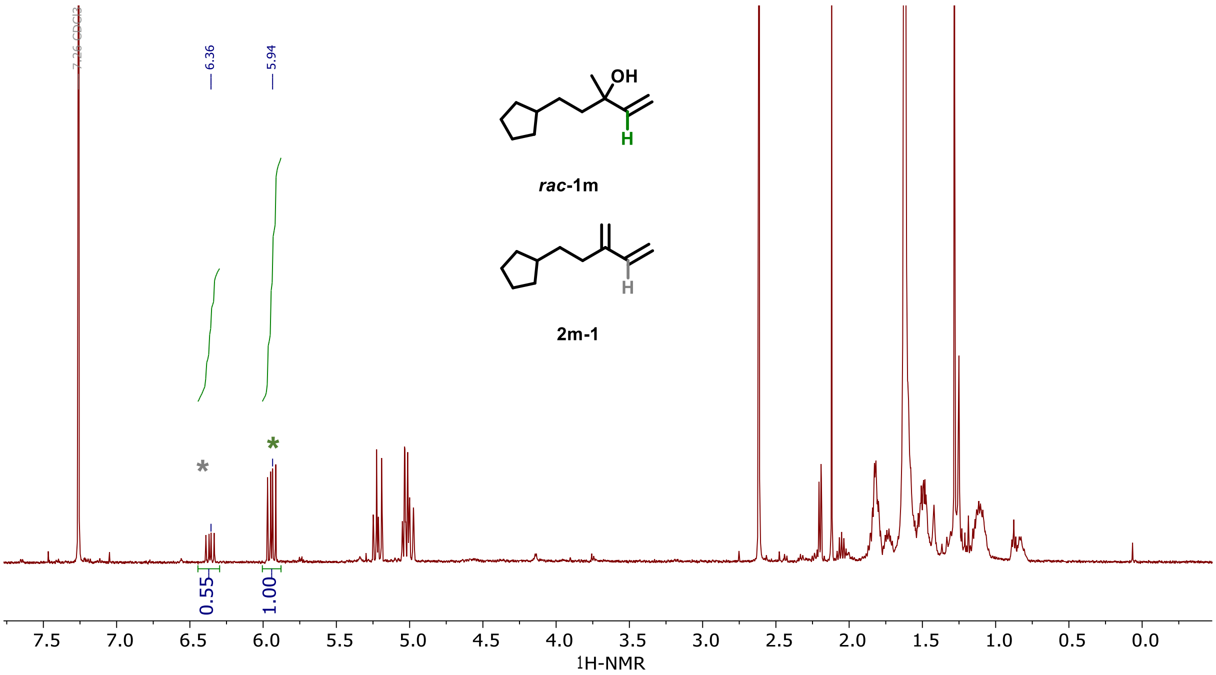

Observed Nucleus: 1H
Observed Frequency: 500.203088735 MHz

Solvent: CDCl3
Number of Scans: 64

Temperature: 298.0012 K
Pulse Sequence: zg30
Relaxation Delay (s): 1 sec

6.49 ^{1}H-NMR: Ref-pentan-2-one

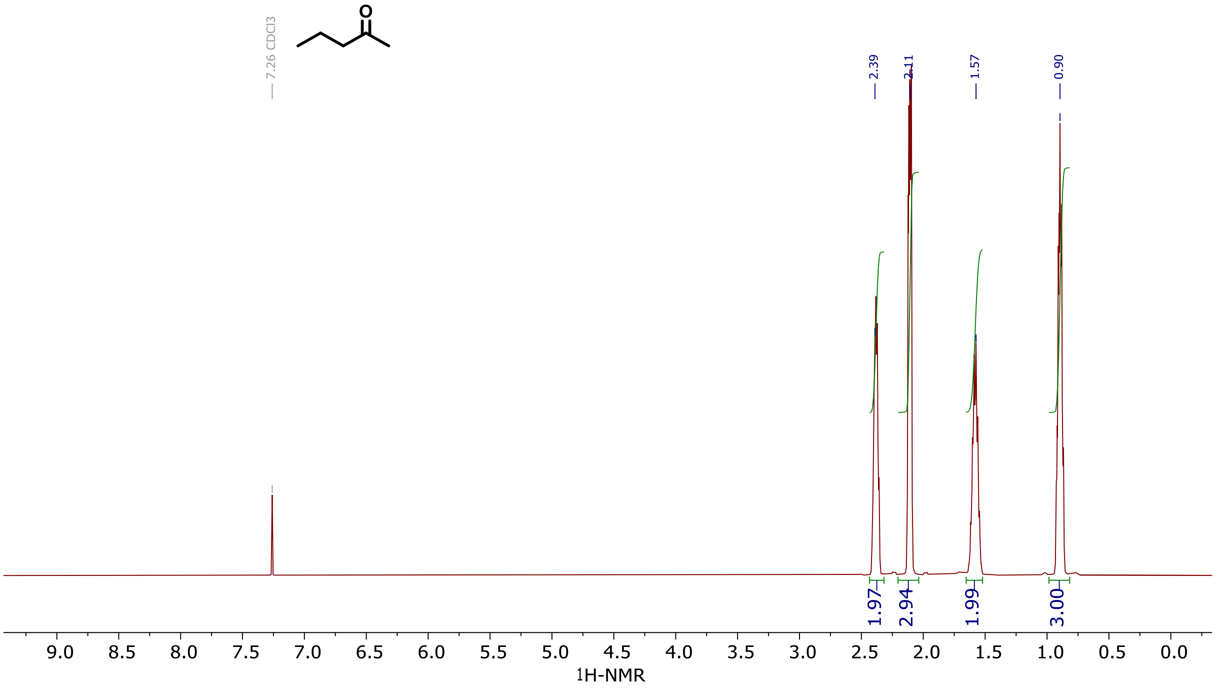

Observed Nucleus: 1H
Observed Frequency: 500.203088735 MHz

Solvent: CDCl3
Number of Scans: 16

Temperature: 297.999 K
Pulse Sequence: zg30
Relaxation Delay (s): 1 sec

6.50 ^{1}H-NMR: *rac*-C_1 alcohol

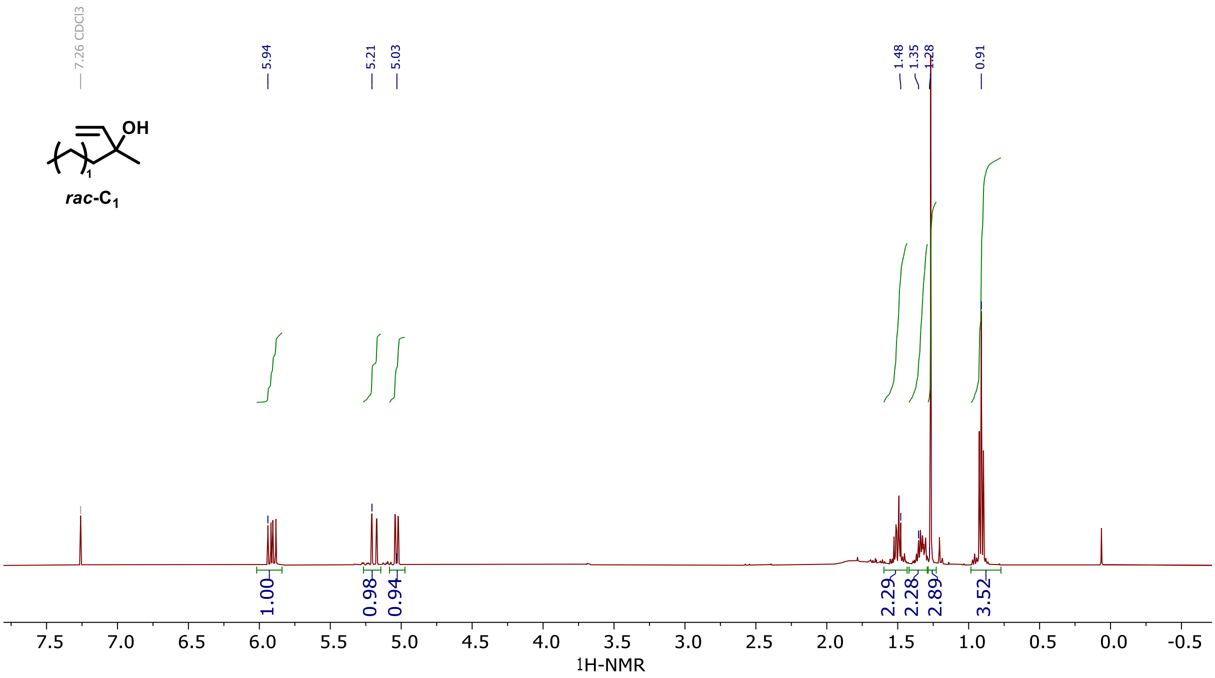

Observed Nucleus: 1H
Observed Frequency: 500.203088735 MHz

Solvent: CDCl3
Number of Scans: 16

Temperature: 297.999 K
Pulse Sequence: zg30
Relaxation Delay (s): 1 sec

6.51 ^{1}H-NMR: Dehydration with *rac*-C_1

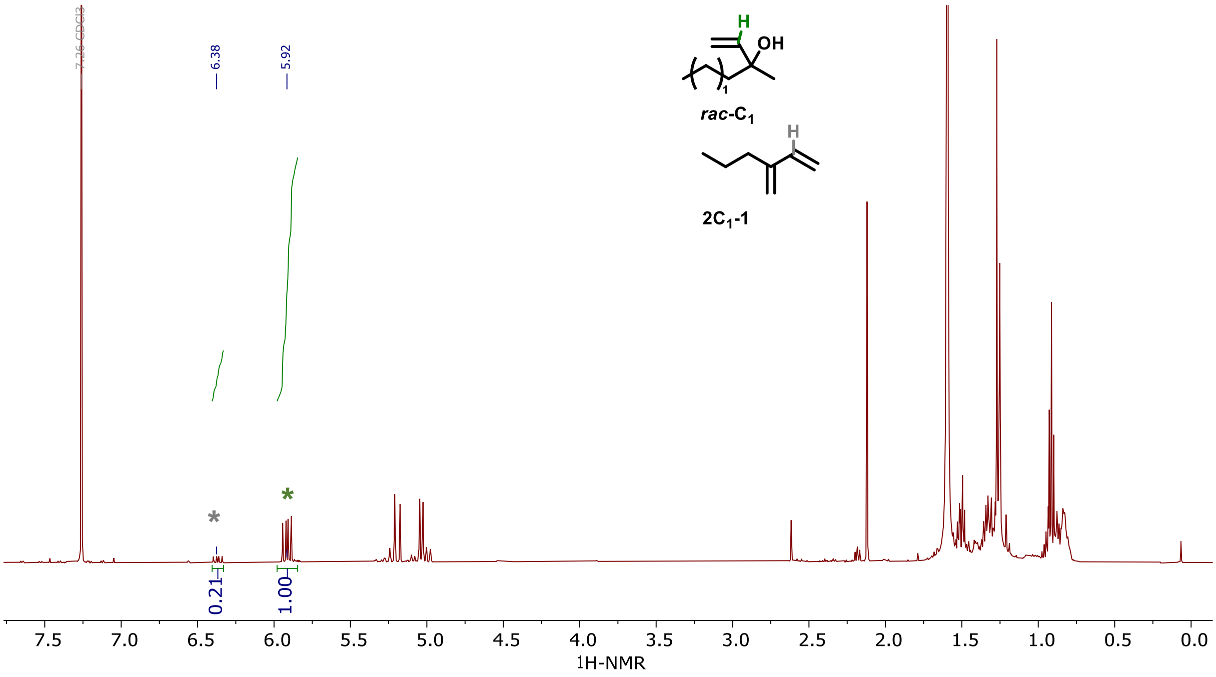

Observed Nucleus: 1H
Observed Frequency: 500.203088735 MHz

Solvent: CDCl3
Number of Scans: 64

Temperature: 298.0012 K
Pulse Sequence: zg30
Relaxation Delay (s): 1 sec

6.52 ^{1}H-NMR: Ref-hexan-2-one

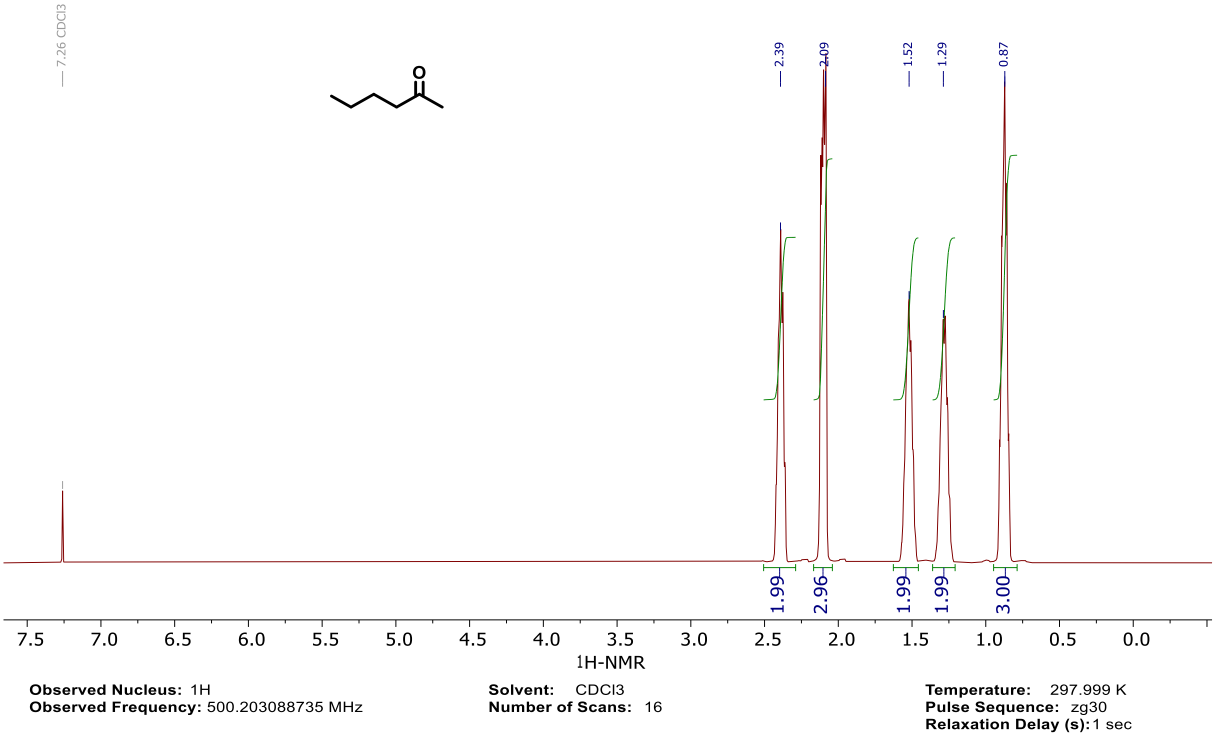

6.53 ^{1}H-NMR: *rac*-C_2

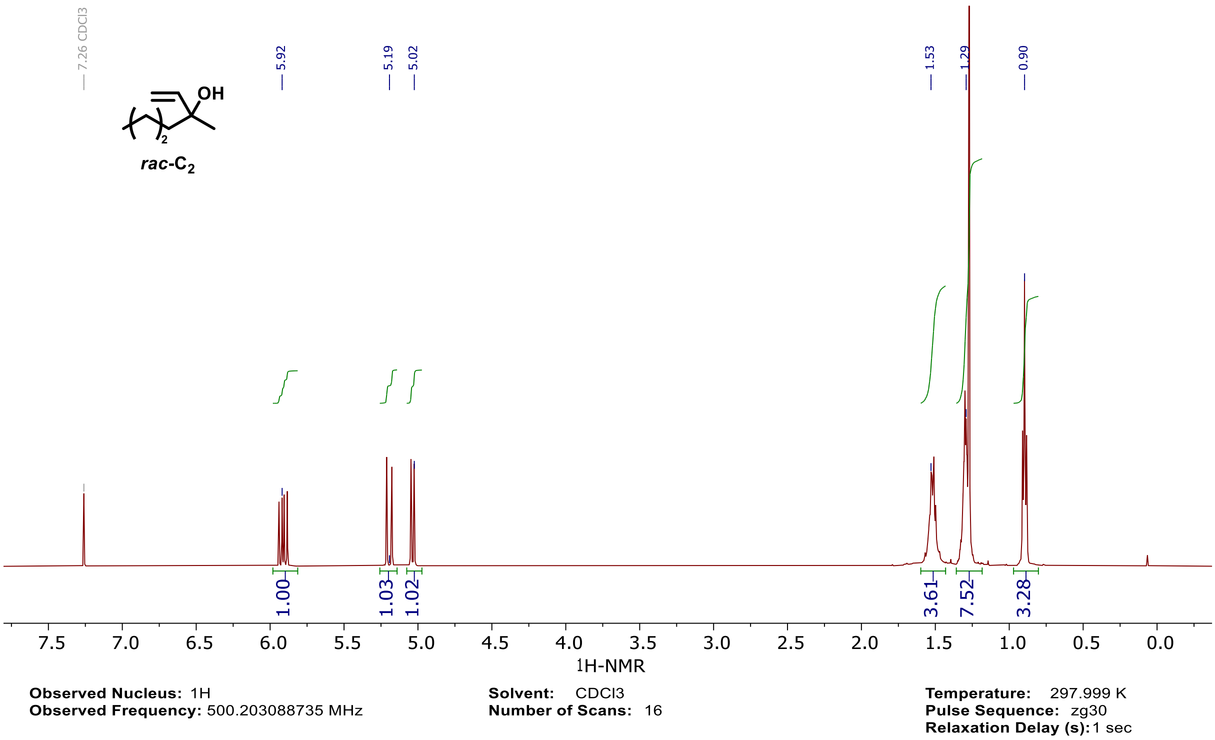

6.54 ¹H-NMR: Dehydration with *rac*-C_2

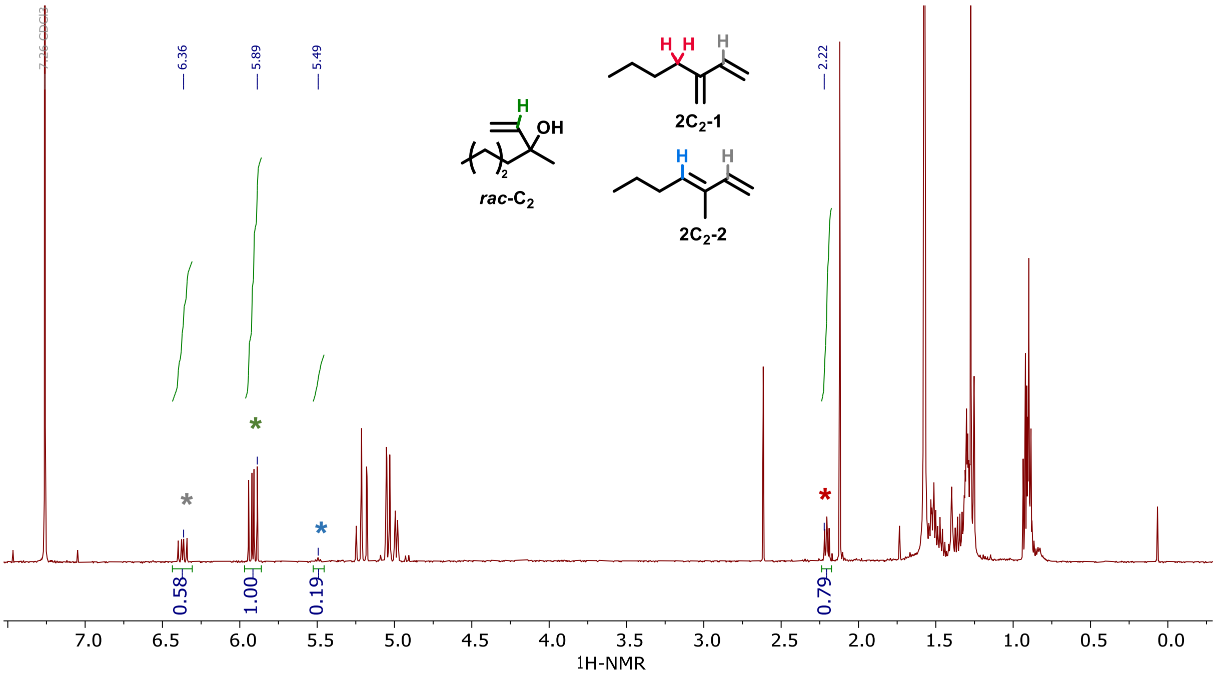

Observed Nucleus: 1H
Observed Frequency: 500.203088735 MHz
Solvent: CDCl3
Number of Scans: 64
Temperature: 298.0012 K
Pulse Sequence: zg30
Relaxation Delay (s): 1 sec

6.55 ¹H-NMR: Ref-heptan-2-one

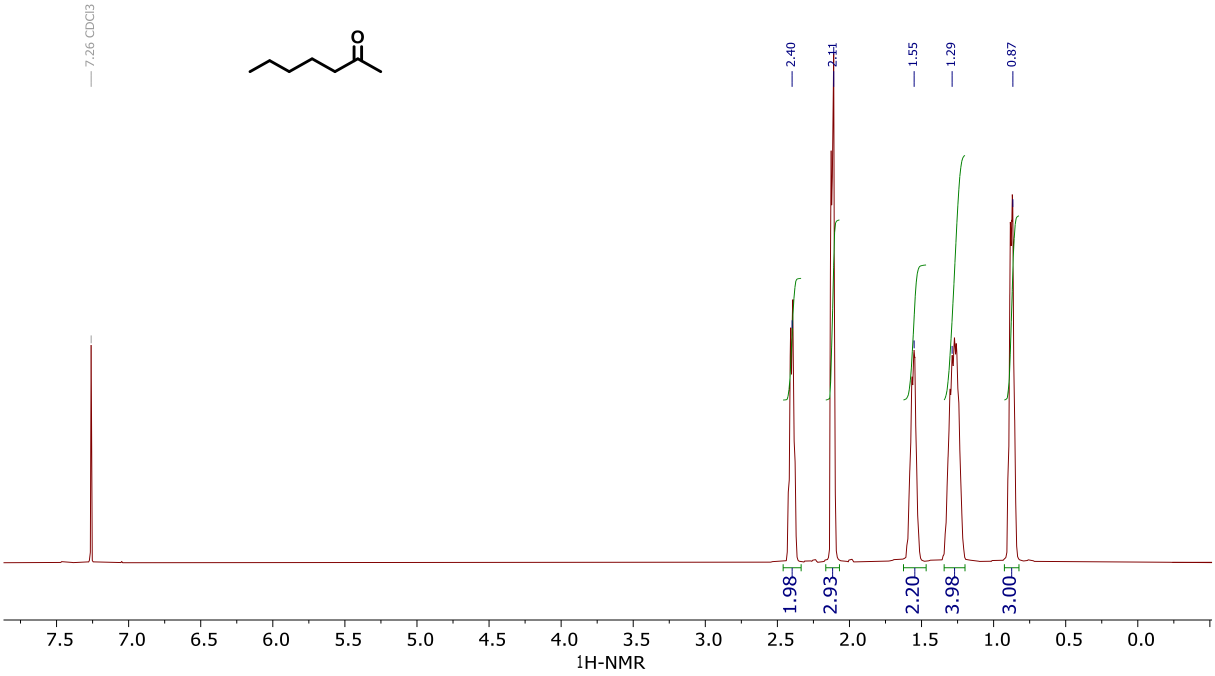

Observed Nucleus: 1H
Observed Frequency: 500.203088735 MHz
Solvent: CDCl3
Number of Scans: 16
Temperature: 297.999 K
Pulse Sequence: zg30
Relaxation Delay (s): 1 sec

6.56 ^{1}H-NMR: *rac*-C$_3$

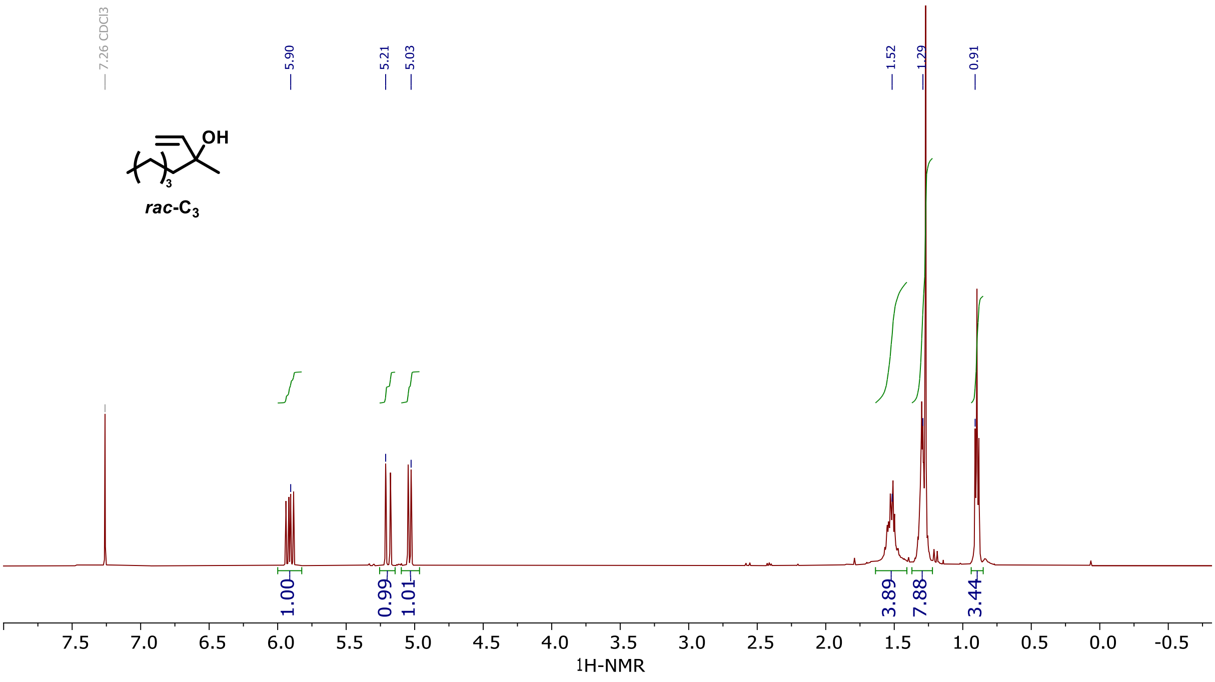

Observed Nucleus: 1H
Observed Frequency: 500.203088735 MHz
Solvent: CDCl3
Number of Scans: 16
Temperature: 297.999 K
Pulse Sequence: zg30
Relaxation Delay (s): 1 sec

6.57 ^{1}H-NMR: Dehydration with *rac*-C$_3$

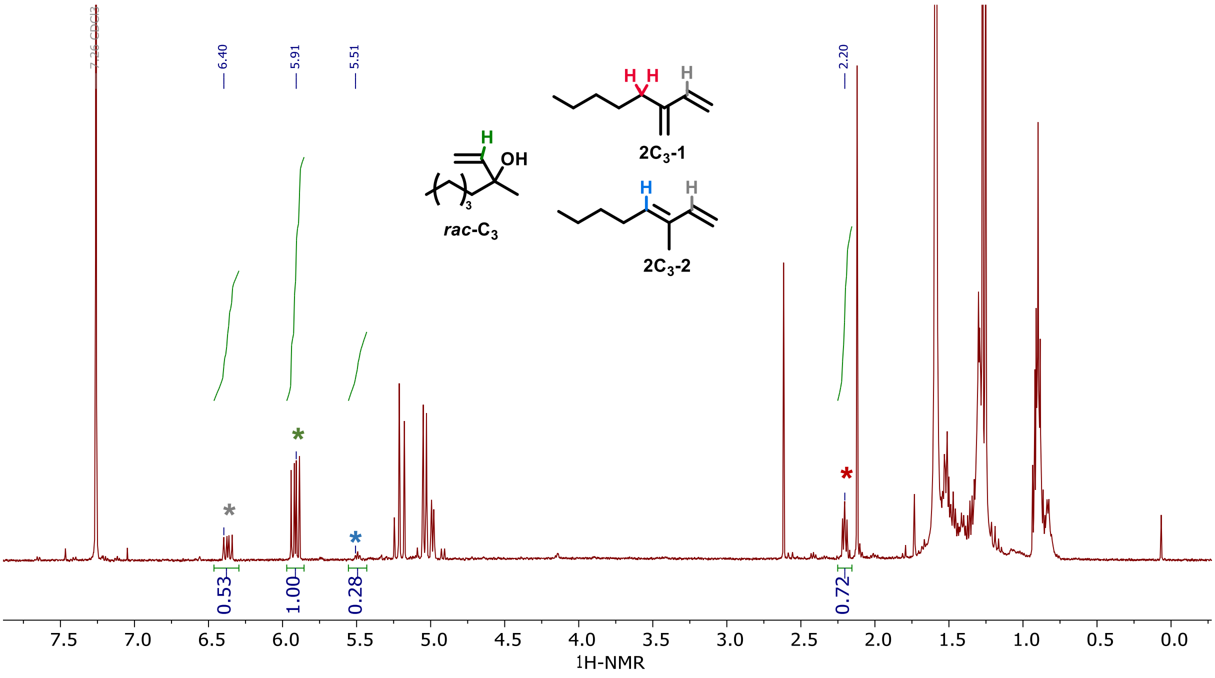

Observed Nucleus: 1H
Observed Frequency: 500.203088735 MHz
Solvent: CDCl3
Number of Scans: 64
Temperature: 298.0012 K
Pulse Sequence: zg30
Relaxation Delay (s): 1 sec

6.58 ^{1}H-NMR: Reference-2-dodecanone

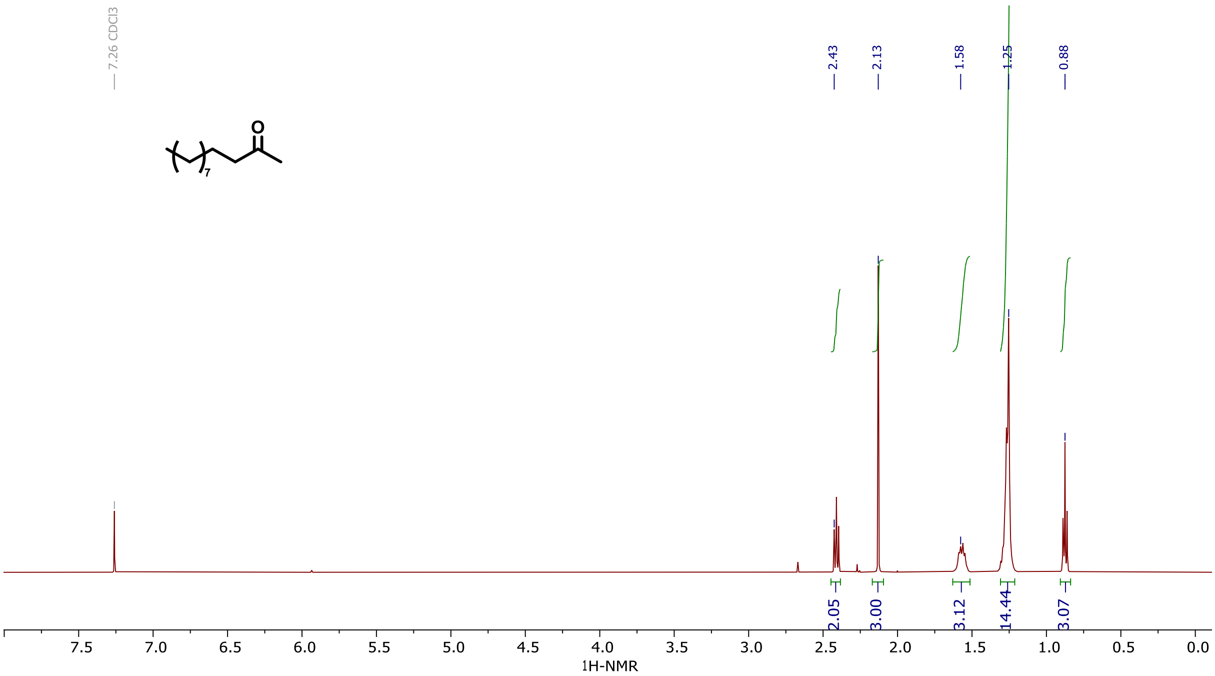

Observed Nucleus: 1H
Observed Frequency: 500.203088735 MHz
Solvent: CDCl3
Number of Scans: 16
Temperature: 297.999 K
Pulse Sequence: zg30
Relaxation Delay (s): 1 sec

6.59 ^{1}H-NMR:Synthesis of 3-methyltridec-1-en-3-ol (*rac*-C_8)

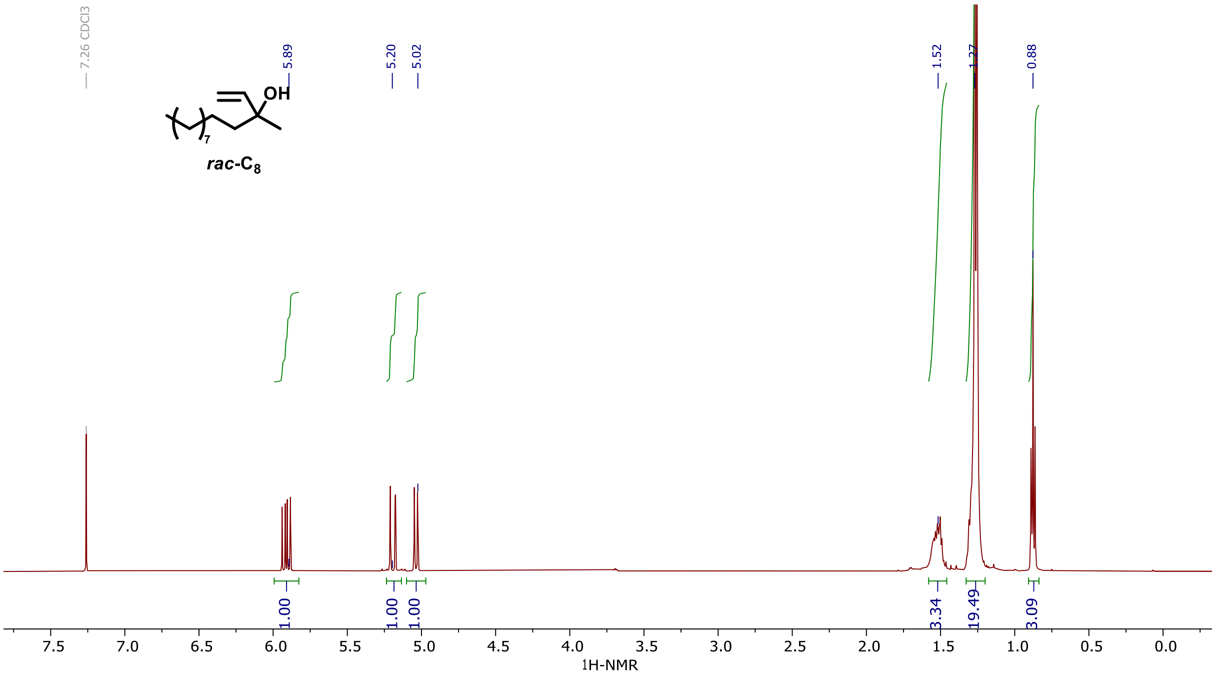

Observed Nucleus: 1H
Observed Frequency: 500.203088735 MHz
Solvent: CDCl3
Number of Scans: 16
Temperature: 297.999 K
Pulse Sequence: zg30
Relaxation Delay (s): 1 sec

6.60 ^{1}H-NMR: Example-Biocatalytic dehydration of *rac*- C_8

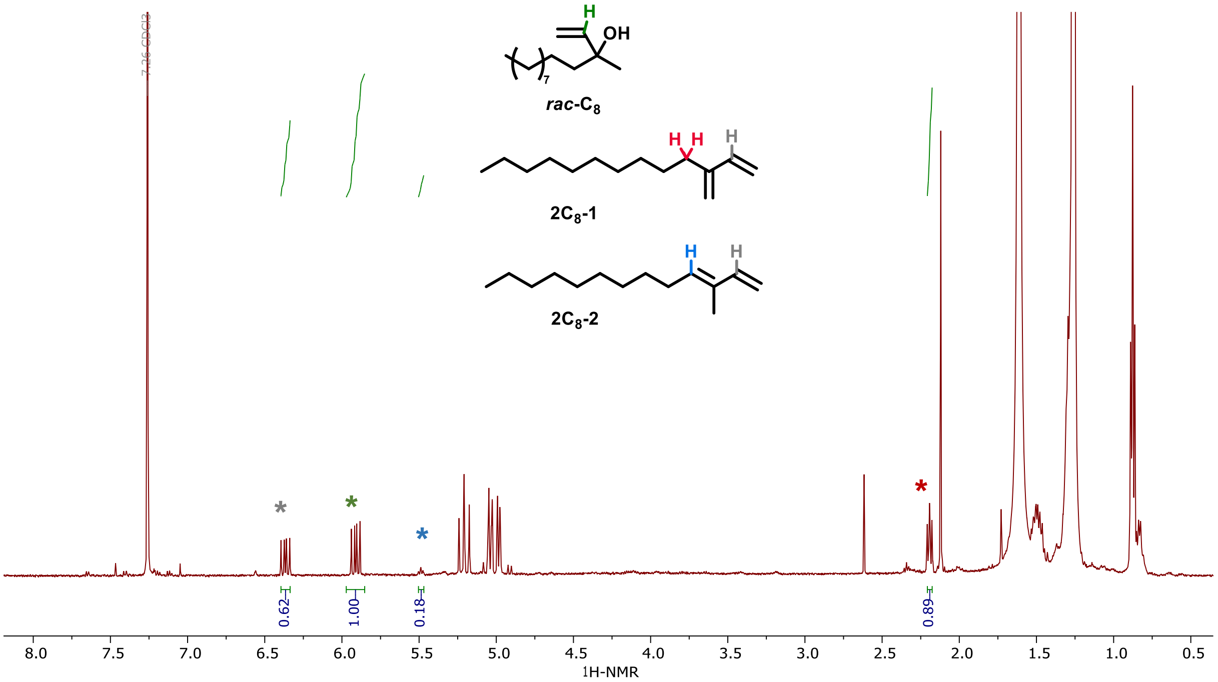

Observed Nucleus: 1H
Observed Frequency: 500.203088735 MHz
Solvent: CDCl3
Number of Scans: 64
Temperature: 297.9984 K
Pulse Sequence: zg30
Relaxation Delay (s): 10 sec

6.61 ^{1}H-NMR: Ref-2-pentadecanone

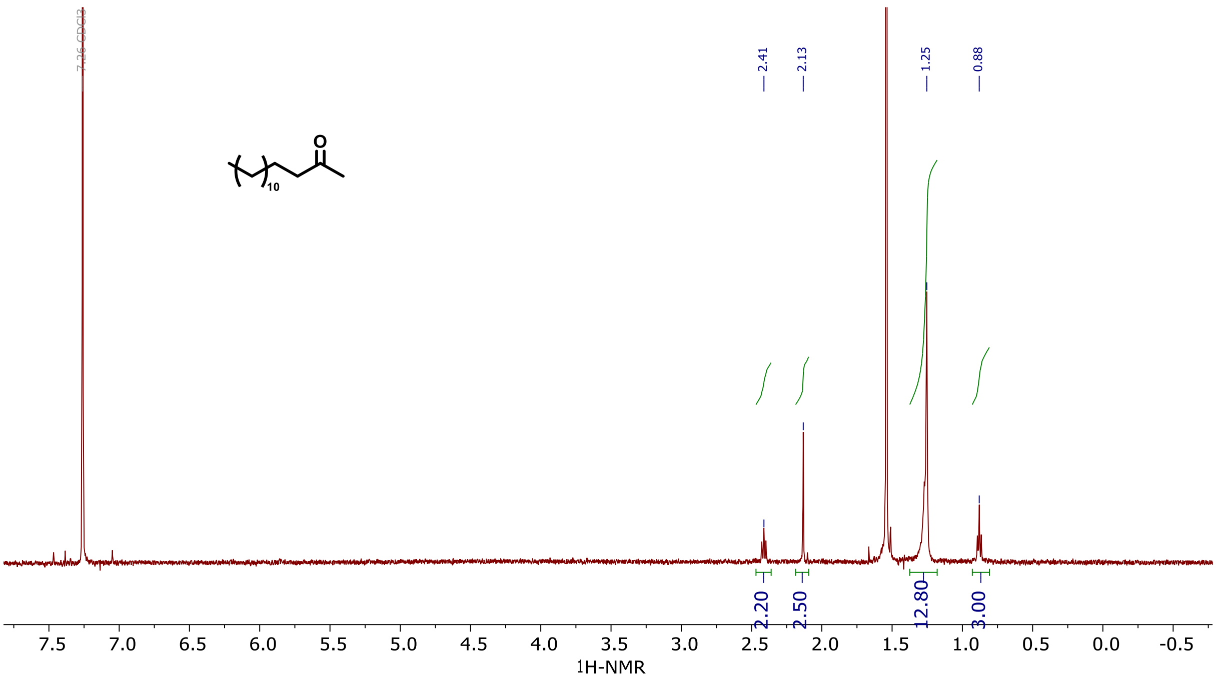

Observed Nucleus: 1H
Observed Frequency: 500.203088735 MHz
Solvent: CDCl3
Number of Scans: 16
Temperature: 297.999 K
Pulse Sequence: zg30
Relaxation Delay (s): 1 sec

6.62 ^{1}H-NMR: *rac*-C_{11}

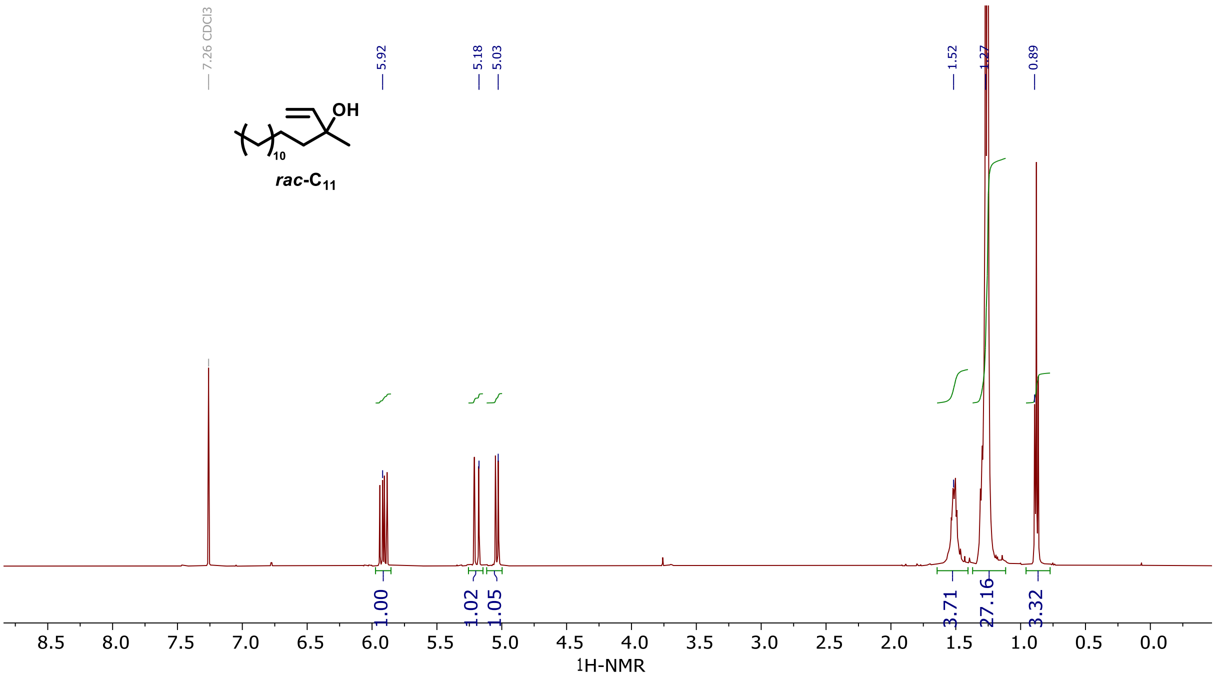

Observed Nucleus: 1H
Observed Frequency: 500.203088735 MHz
Solvent: CDCl3
Number of Scans: 16
Temperature: 297.999 K
Pulse Sequence: zg30
Relaxation Delay (s): 1 sec

6.63 ^{1}H-NMR: Dehydration with *rac*-C_{11}

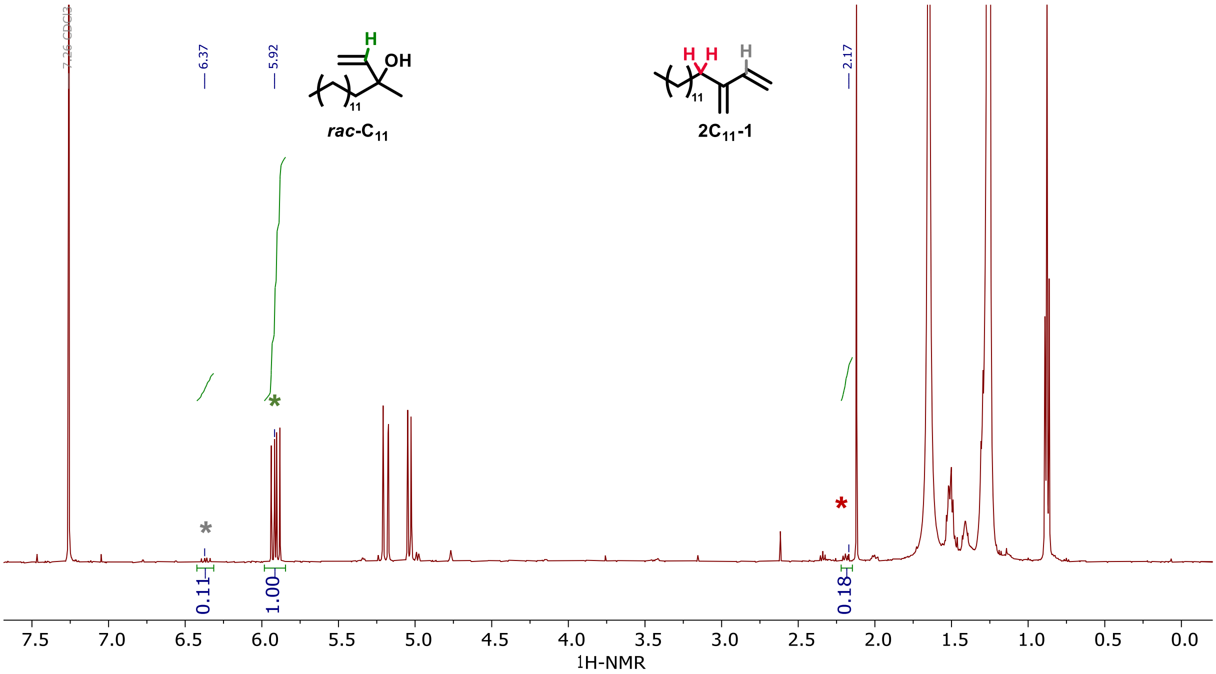

Observed Nucleus: 1H
Observed Frequency: 500.203088735 MHz
Solvent: CDCl3
Number of Scans: 64
Temperature: 297.9984 K
Pulse Sequence: zg30
Relaxation Delay (s): 10 sec

6.64 ^{1}H-NMR: Ref-1-hexen-3-ol

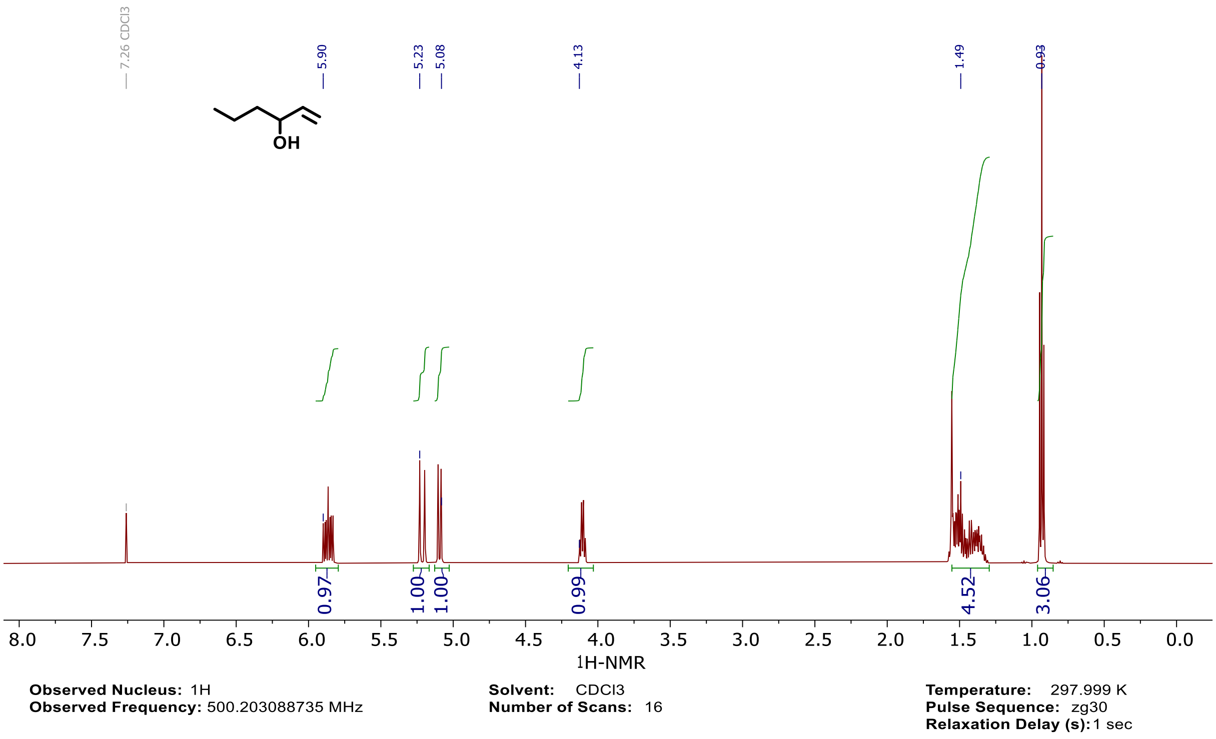

6.65 ^{1}H-NMR: Attempted reaction with *rac*-1n (No detectable product formation)

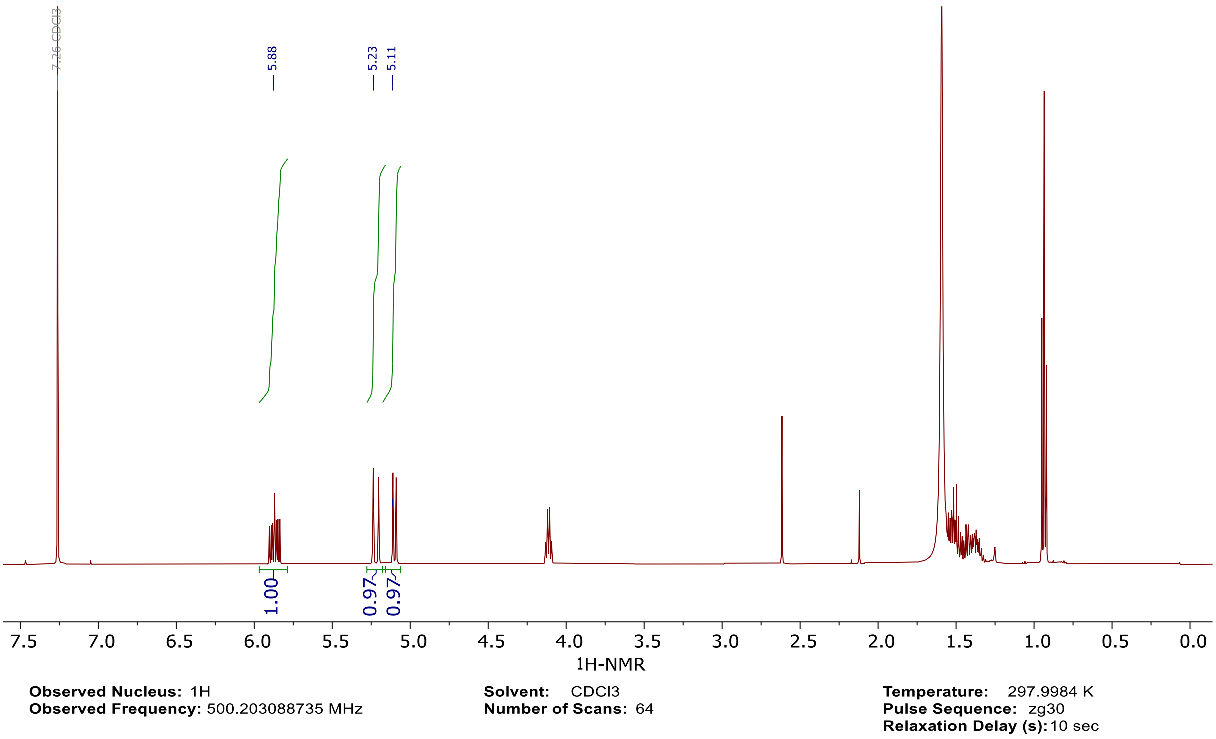

6.66 ^{1}H-NMR: Ref-4-methylpentan-2-one

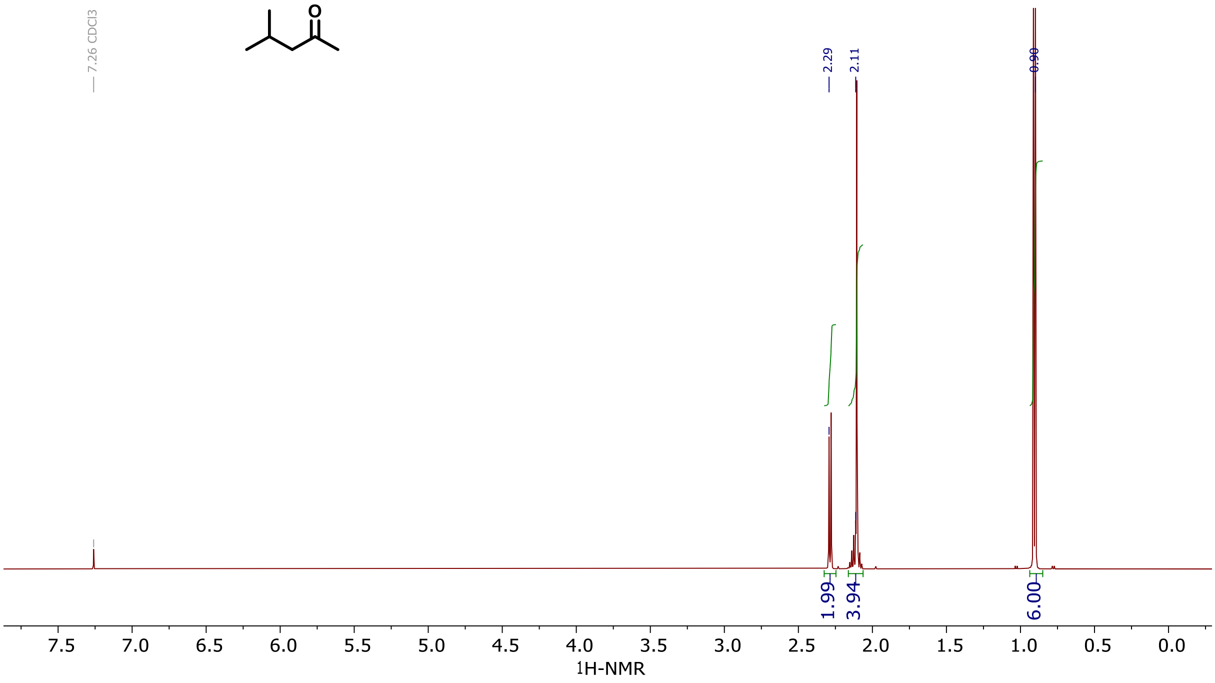

Observed Nucleus: 1H
Observed Frequency: 500.203088735 MHz
Solvent: CDCl3
Number of Scans: 16
Temperature: 297.999 K
Pulse Sequence: zg30
Relaxation Delay (s): 1 sec

6.67 ^{1}H-NMR: *rac*-1o

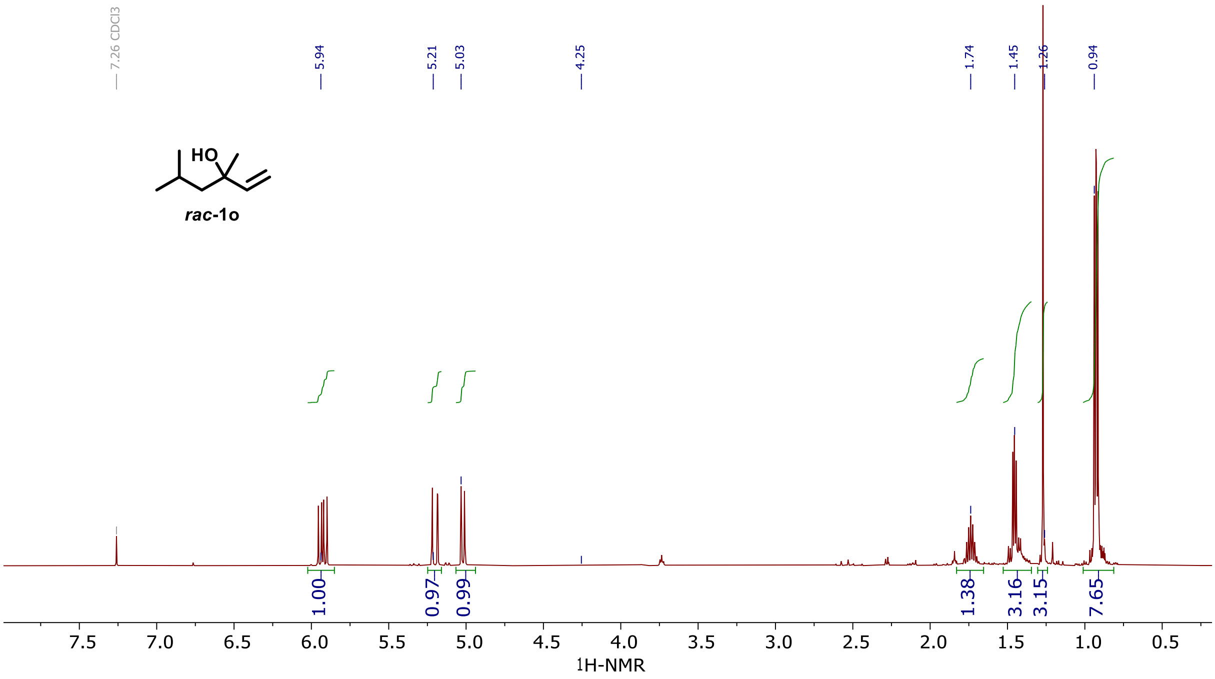

Observed Nucleus: 1H
Observed Frequency: 500.203088735 MHz
Solvent: CDCl3
Number of Scans: 16
Temperature: 297.999 K
Pulse Sequence: zg30
Relaxation Delay (s): 1 sec

6.68 ^{1}H-NMR: Dehydration with *rac*-1o

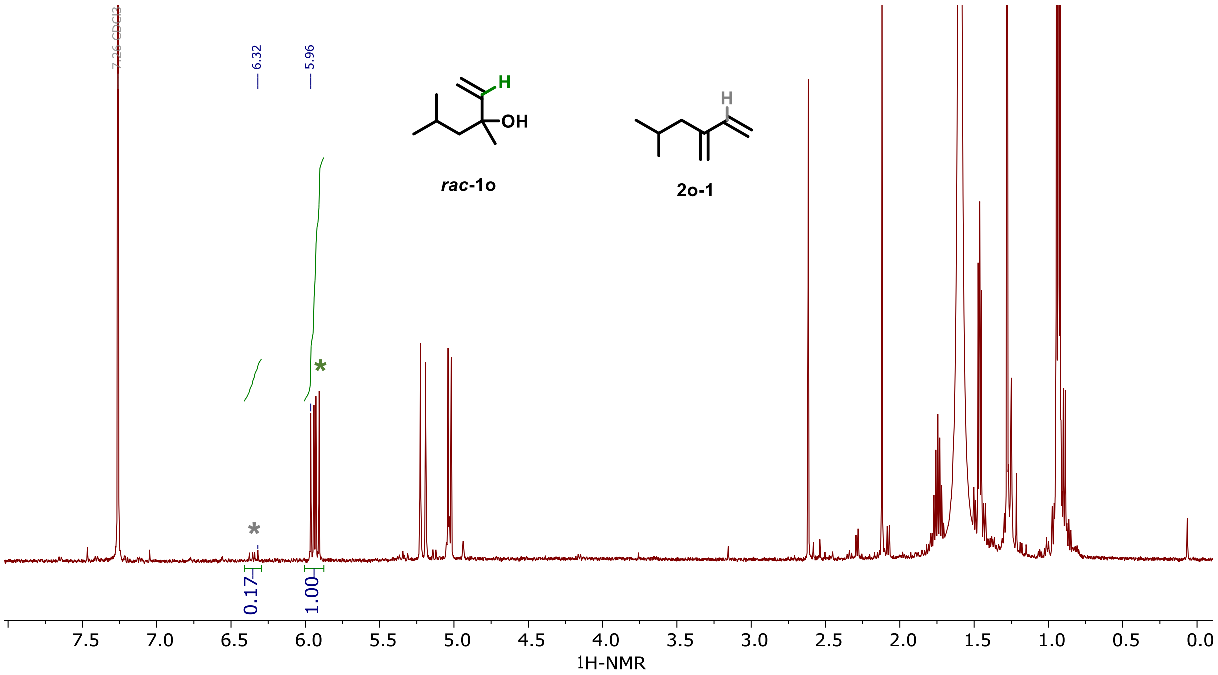

Observed Nucleus: 1H
Observed Frequency: 500.203088735 MHz
Solvent: CDCl3
Number of Scans: 64
Temperature: 297.9984 K
Pulse Sequence: zg30
Relaxation Delay (s): 10 sec

6.69 ^{1}H-NMR: Ref-2-methylhexan-3-one

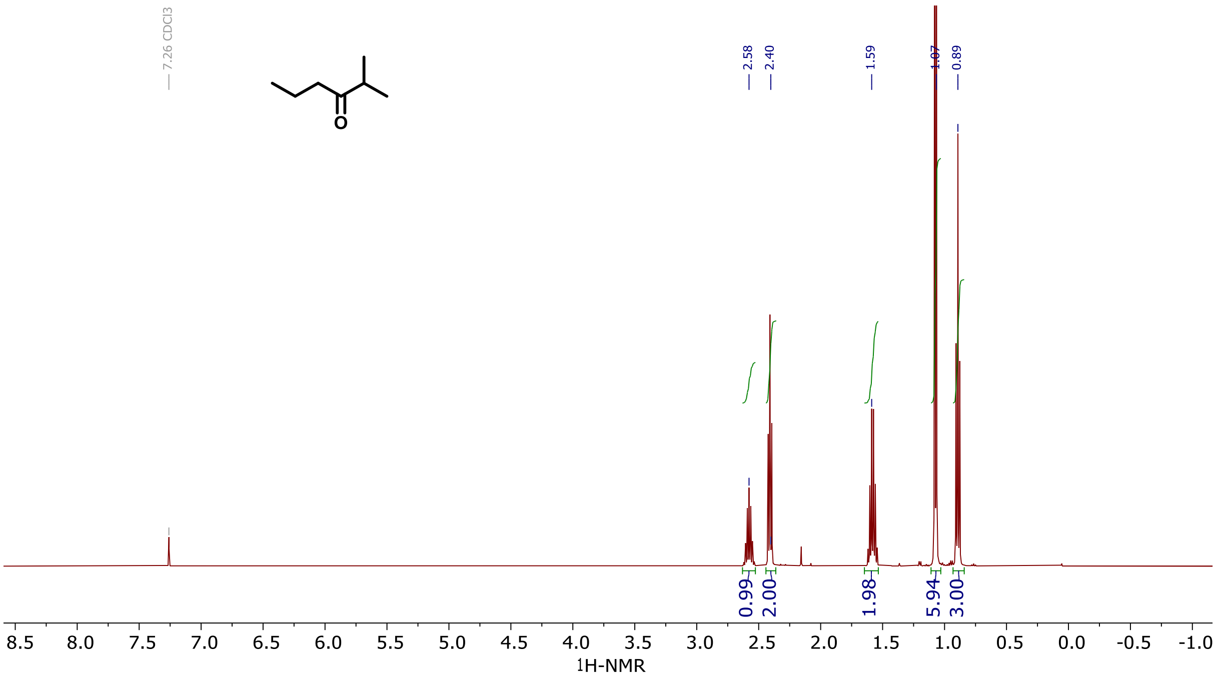

Observed Nucleus: 1H
Observed Frequency: 500.203088735 MHz
Solvent: CDCl3
Number of Scans: 16
Temperature: 297.999 K
Pulse Sequence: zg30
Relaxation Delay (s): 1 sec

6.70 ^{1}H-NMR: *rac*-1p

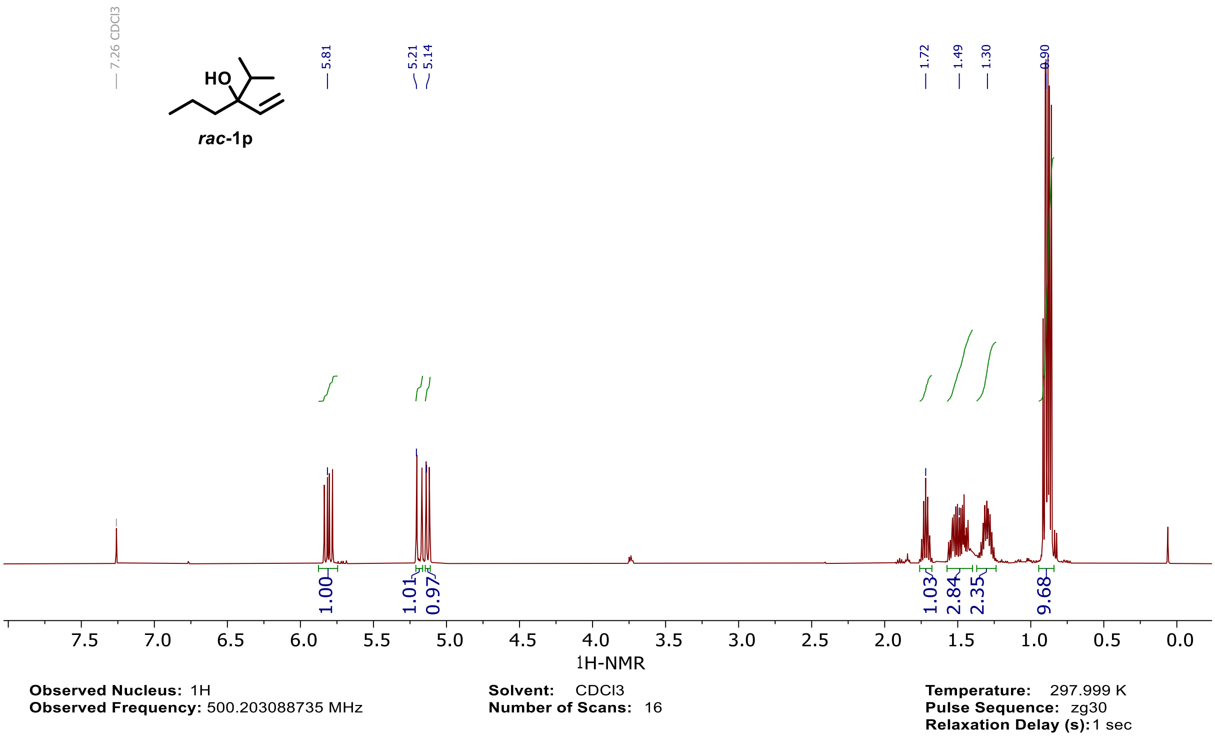

6.71 ^{1}H-NMR: Attempted reaction with *rac*-1p (No detectable product formation)

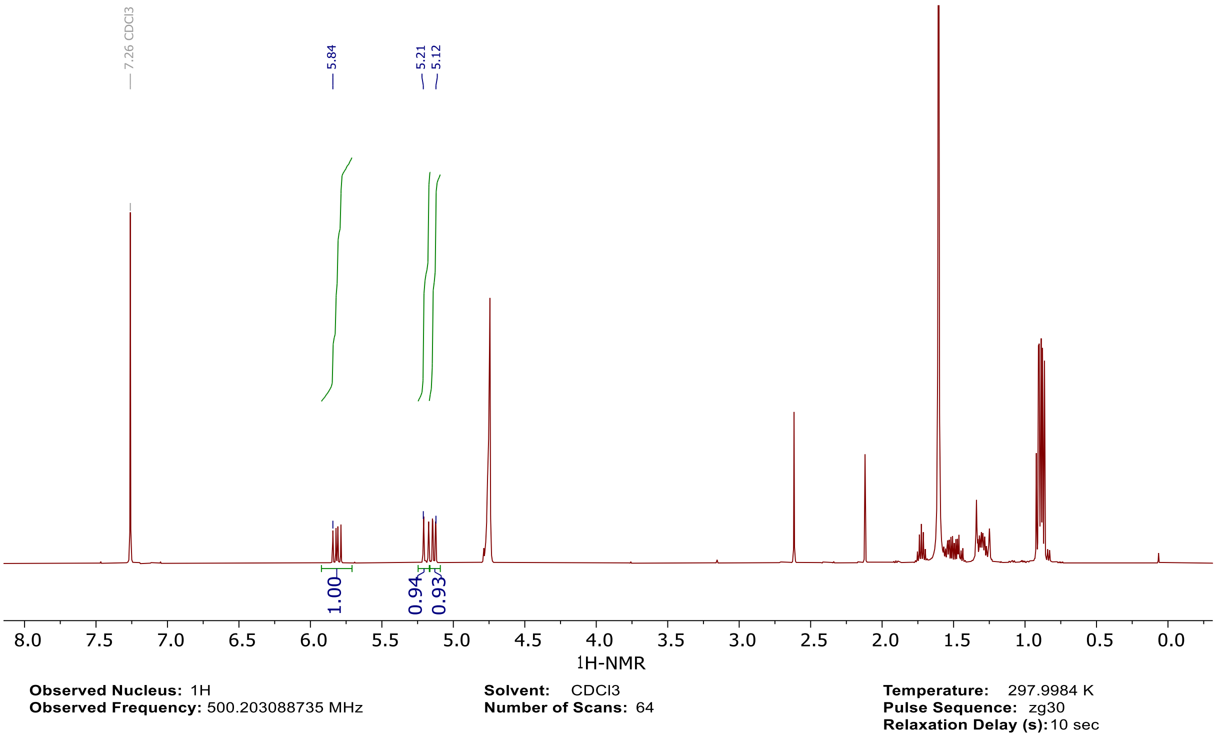

6.72 ^{1}H-NMR: Ref- 4-isopropoxybutan-2-one

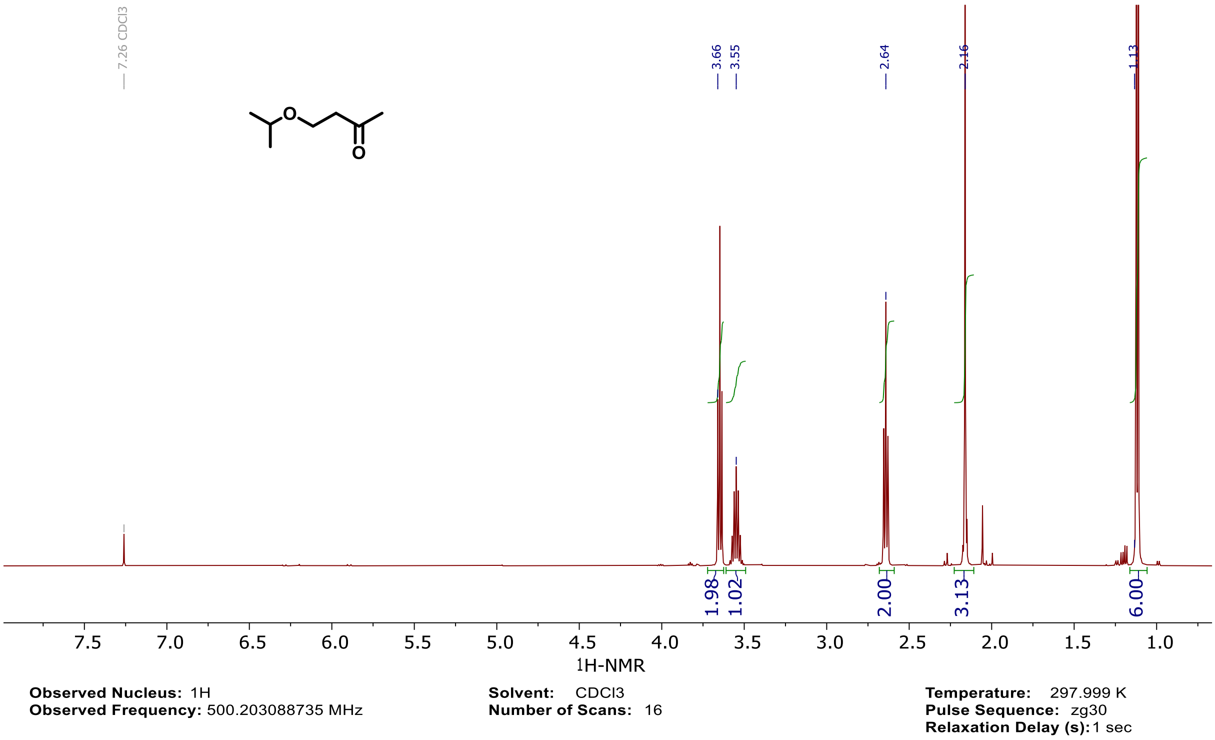

6.73 ^{1}H-NMR: *rac*-1q

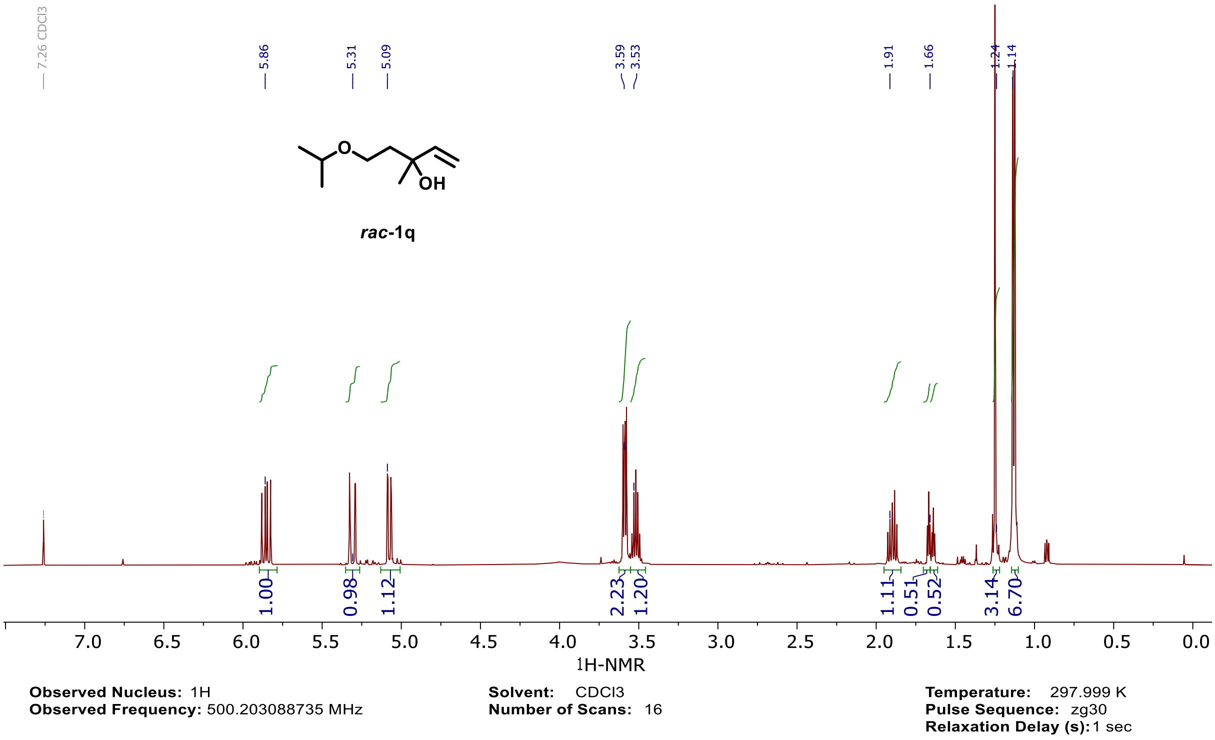

6.74 ^{1}H-NMR: Attempted reaction with *rac*-1q (No detectable product formation)

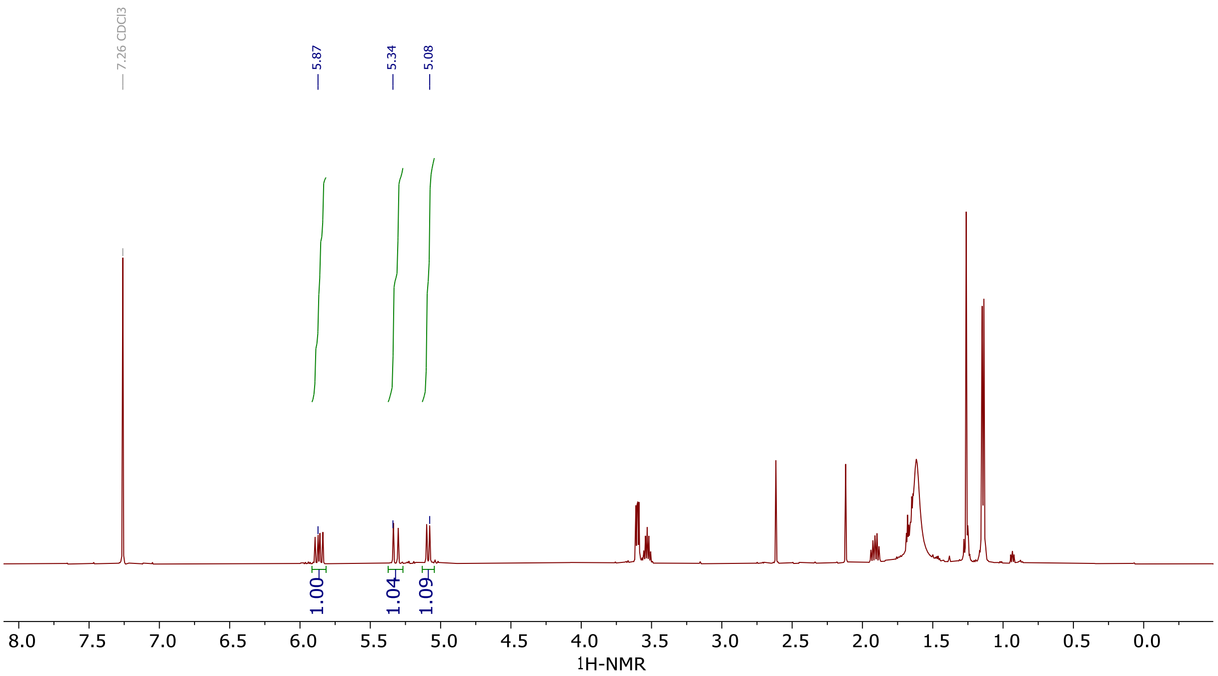

Observed Nucleus: 1H
Observed Frequency: 500.203088735 MHz
Solvent: CDCl3
Number of Scans: 64
Temperature: 297.9984 K
Pulse Sequence: zg30
Relaxation Delay (s): 10 sec

6.75 ^{1}H-NMR: Ref-(1S,4R)-bicyclo[2.2.1]heptan-2-one

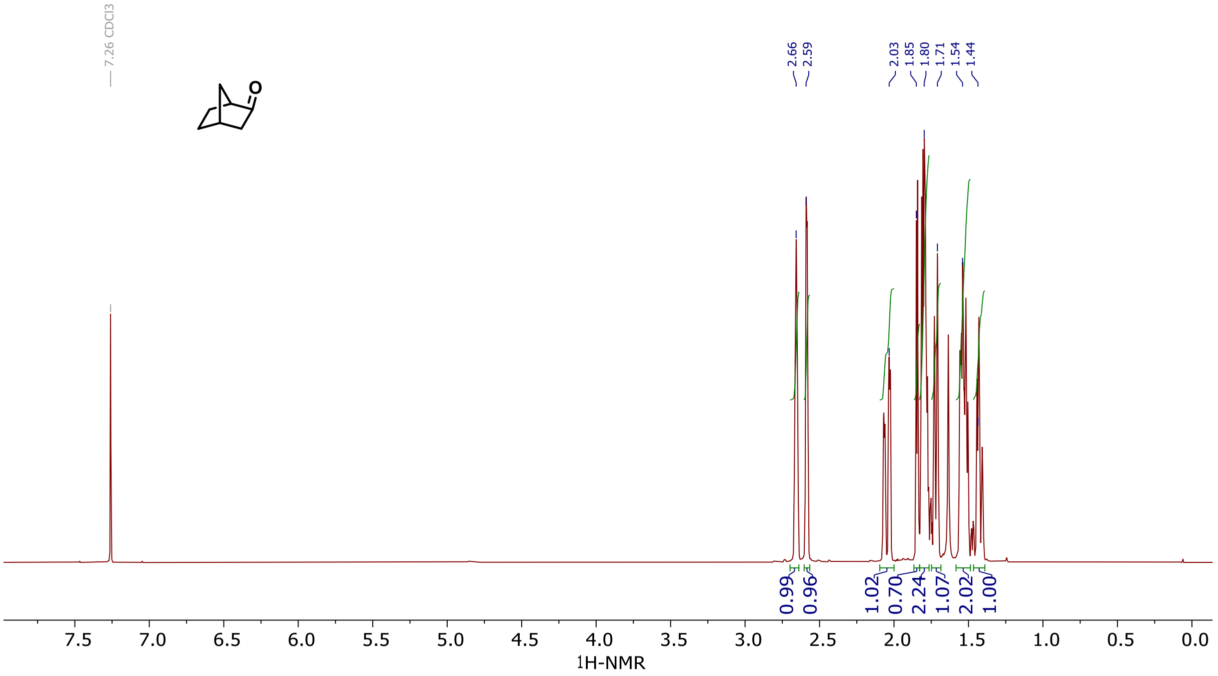

Observed Nucleus: 1H
Observed Frequency: 500.203088735 MHz
Solvent: CDCl3
Number of Scans: 16
Temperature: 297.999 K
Pulse Sequence: zg30
Relaxation Delay (s): 1 sec

6.76 ^{1}H-NMR: *rac*-1r

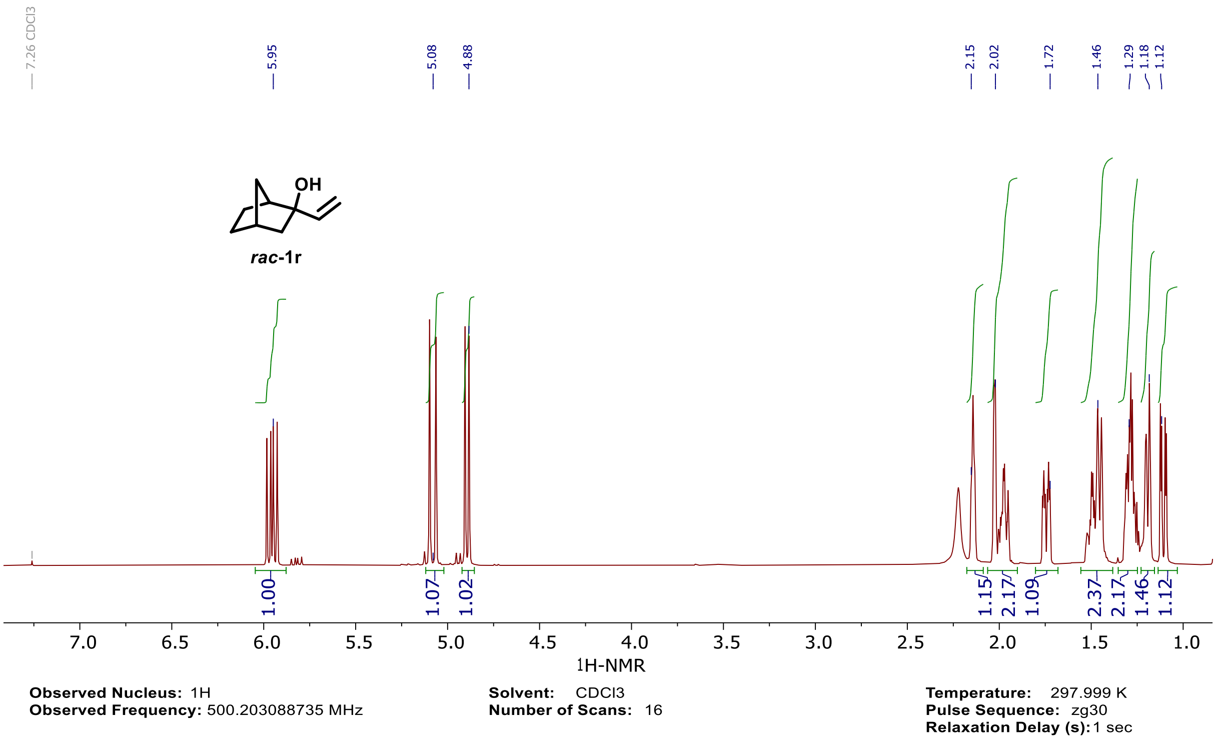

6.77 ^{1}H-NMR: Attempted reaction with *rac*-1r (No detectable product formation)

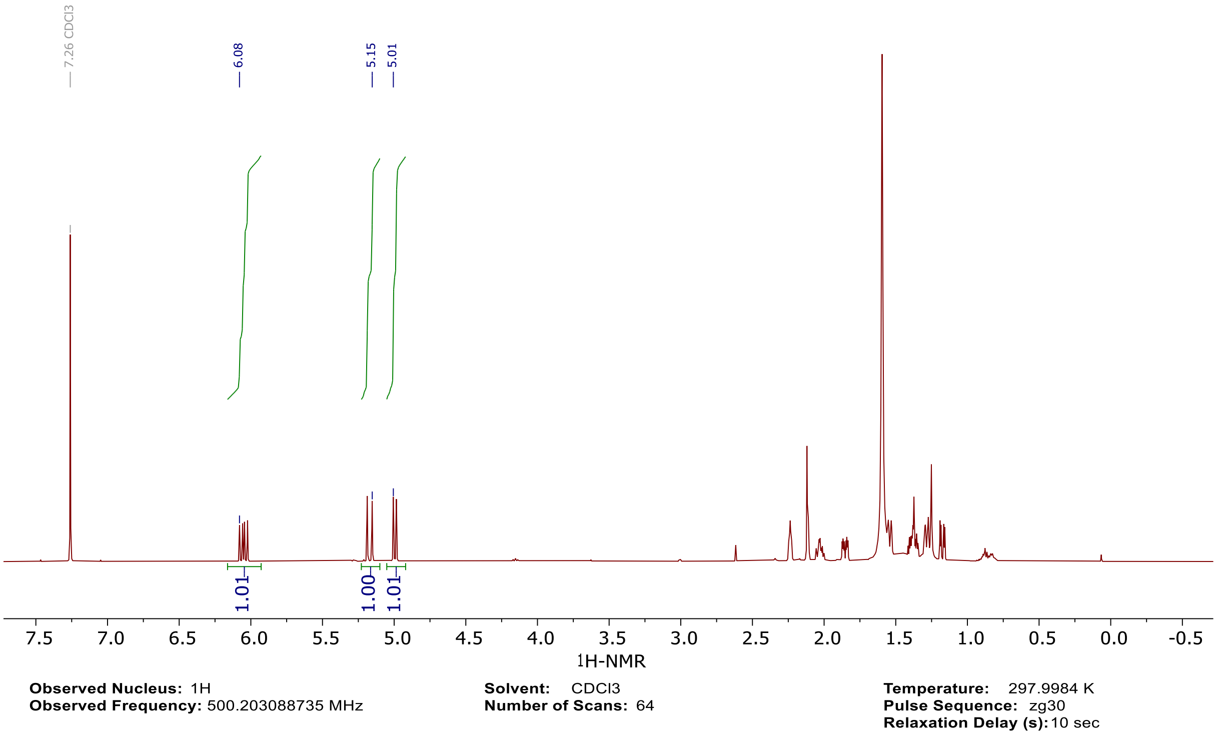

6.78 ¹H-NMR: Ref-cyclooctanone

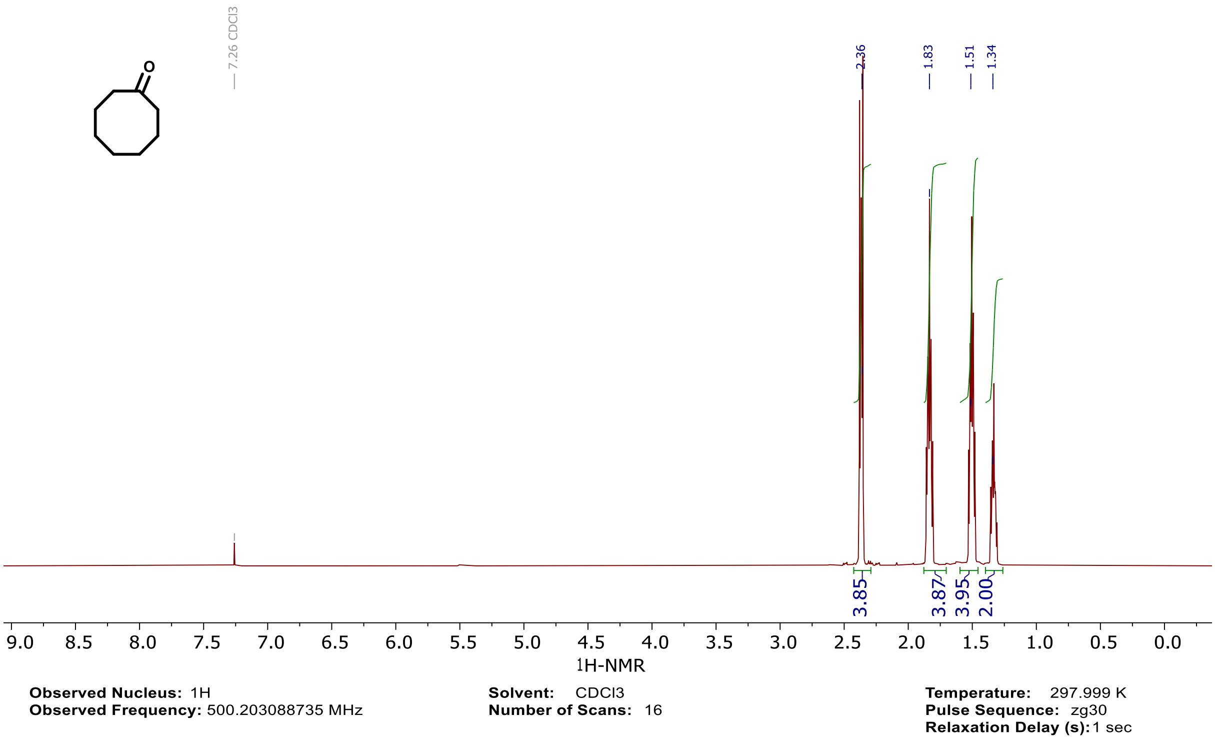

6.79 ¹H-NMR: *rac*-1s

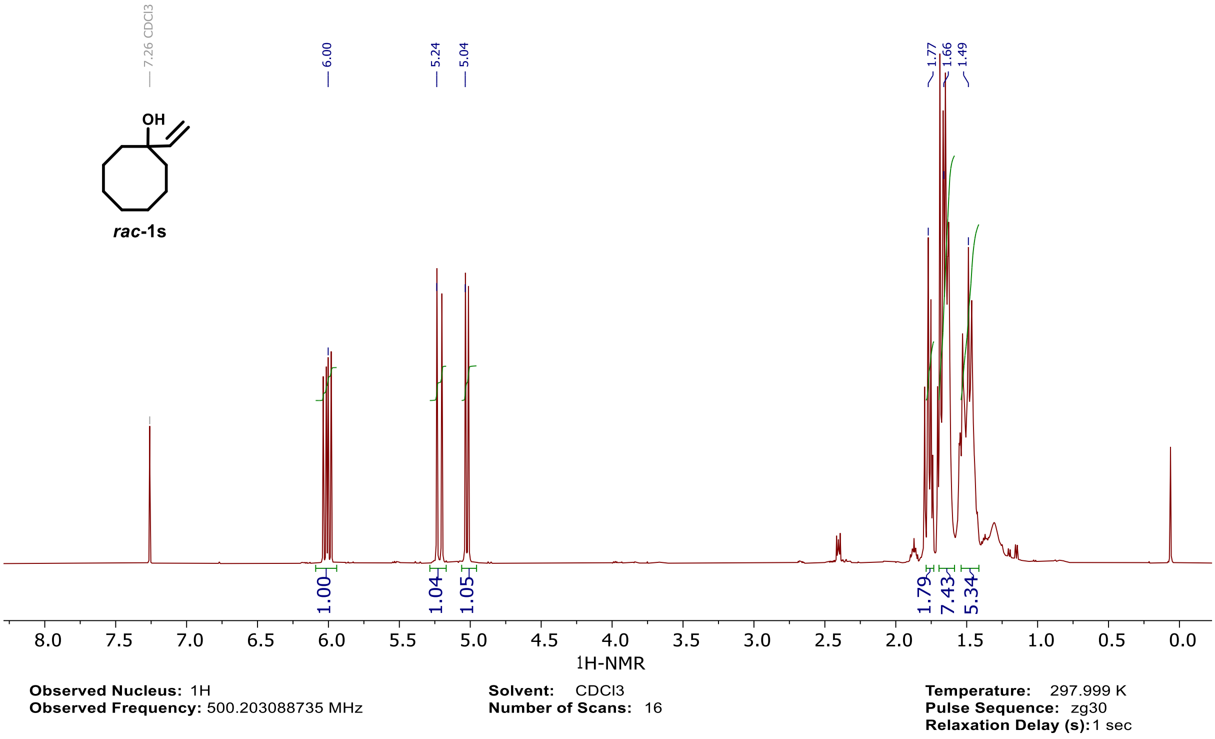

6.80 ^{1}H-NMR: Attempted reaction with *rac*-1s (No detectable product formation)

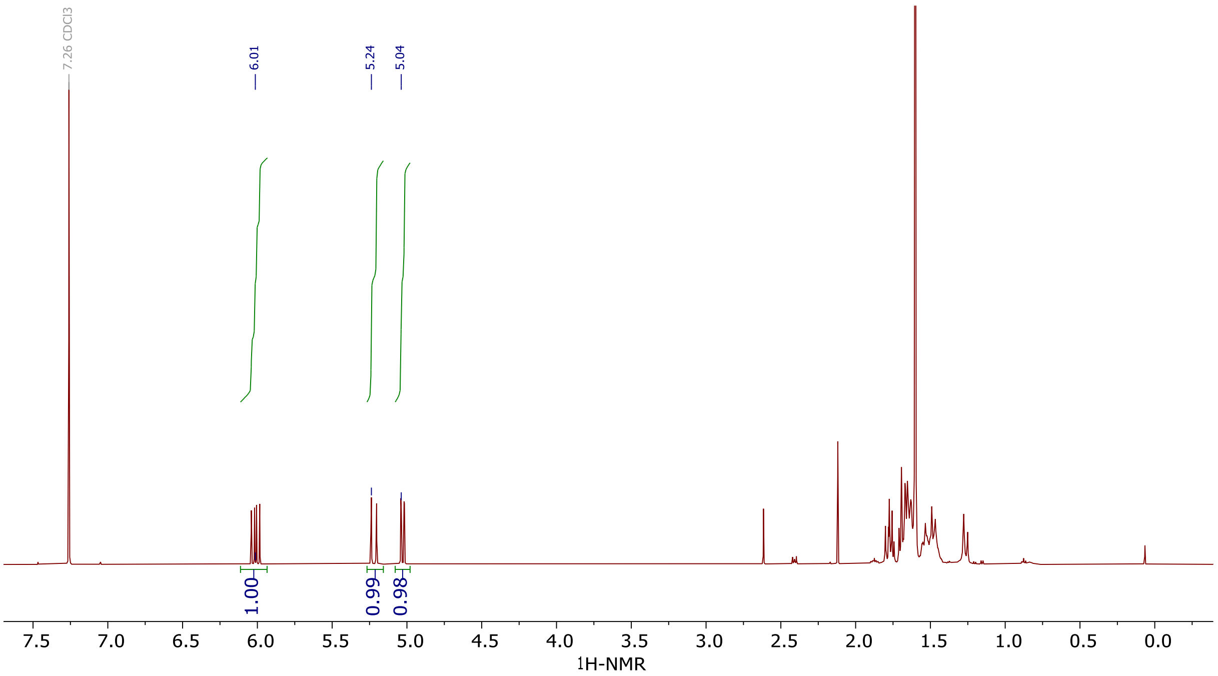

Observed Nucleus: 1H
Observed Frequency: 500.203088735 MHz
Solvent: CDCl3
Number of Scans: 64
Temperature: 297.9984 K
Pulse Sequence: zg30
Relaxation Delay (s): 10 sec

6.81 ^{1}H-NMR: *rac*-1t

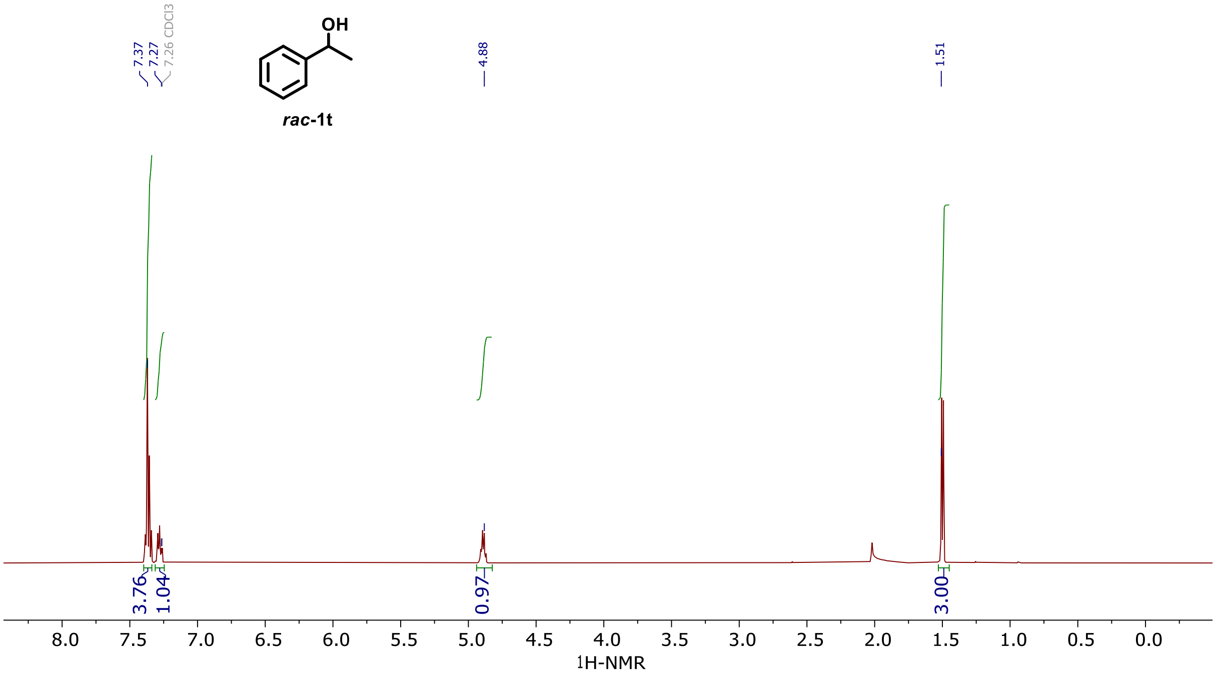

Observed Nucleus: 1H
Observed Frequency: 500.203088735 MHz
Solvent: CDCl3
Number of Scans: 16
Temperature: 297.999 K
Pulse Sequence: zg30
Relaxation Delay (s): 1 sec

6.82 ^{1}H-NMR: Attempted reaction with *rac*-1s (No detectable product formation)

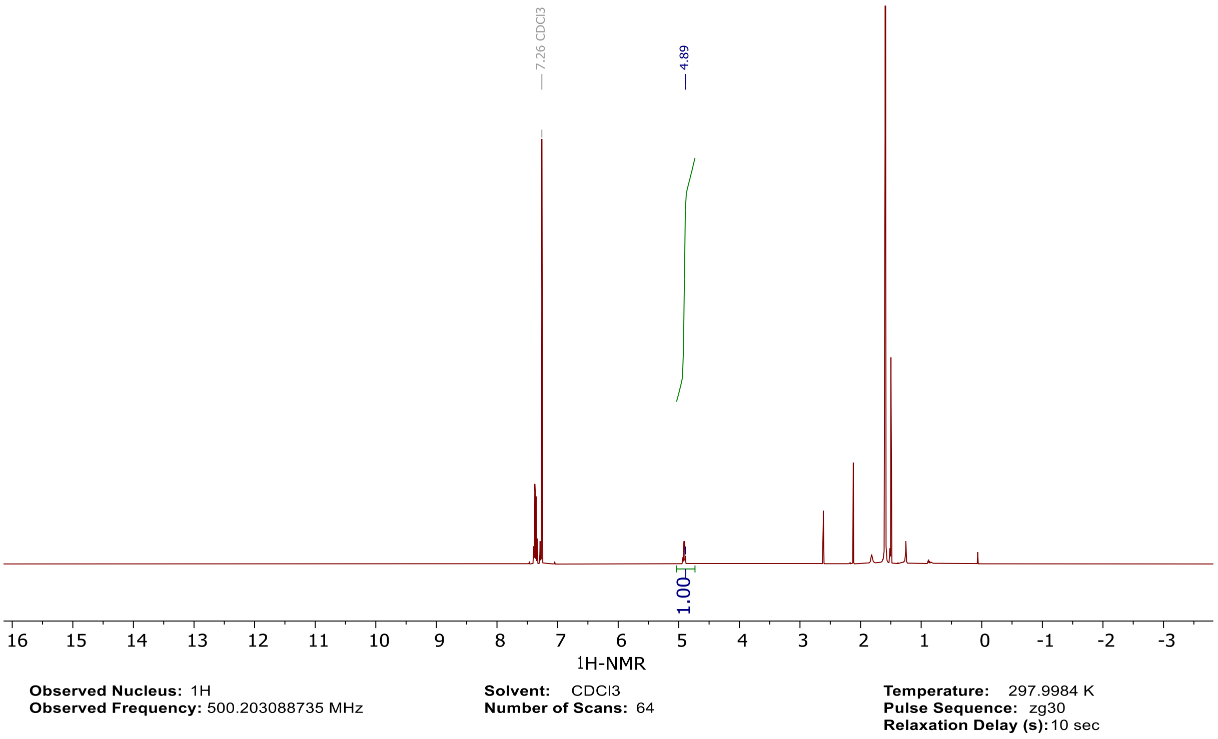

6.83 ^{1}H-NMR: *rac*-1u

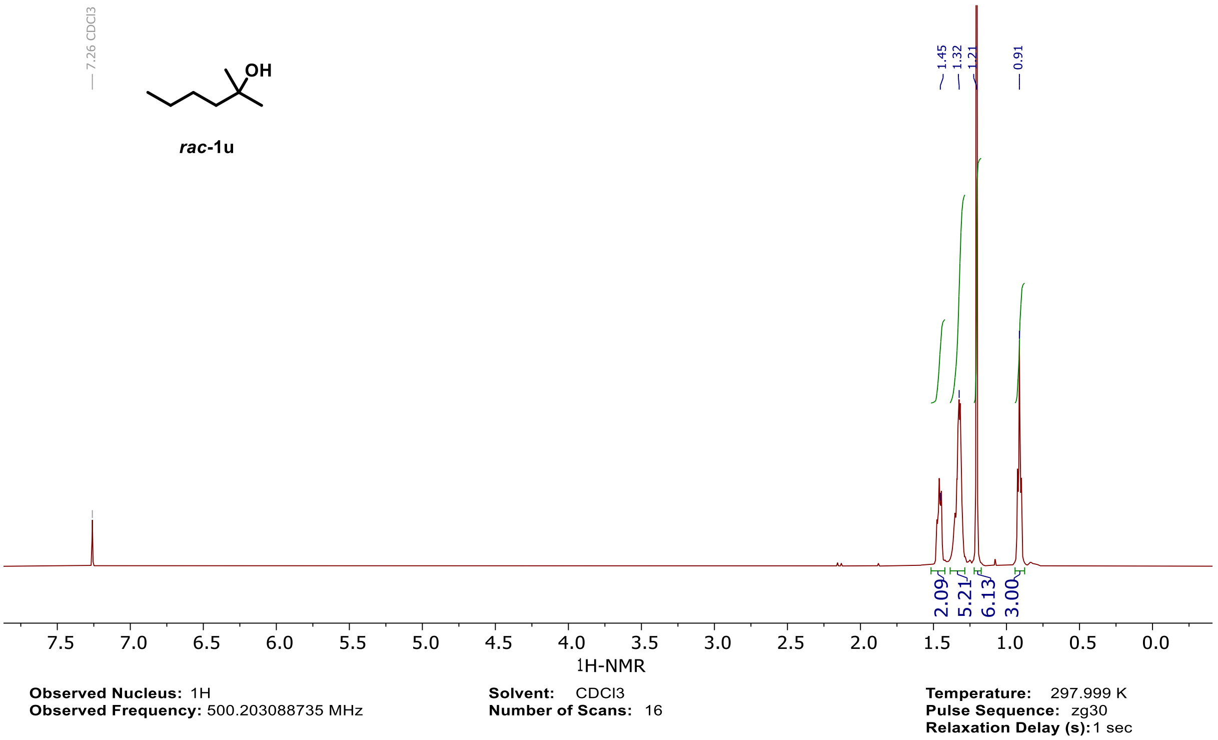

6.84 ¹H-NMR: Attempted reaction with *rac*-1u (No detectable product formation)

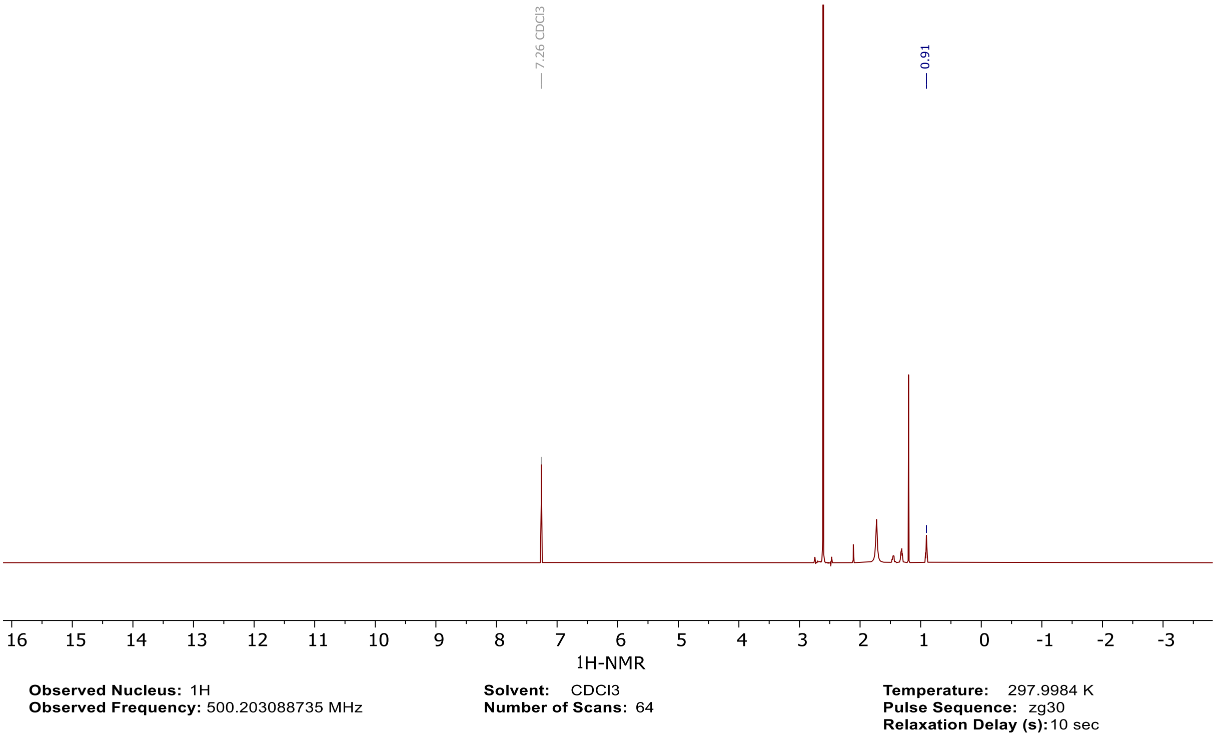

7. DNA/AA sequences

The codon-optimized protein sequences of the linalool dehydratase used, which have a C-terminal His_6 tag and are cloned into the vector pET-28(+), are given below.

LinD from *Castellaniella defragrans* (C-terminaler His_6-Tag) 6735 bp

DNA sequence

GGCGGTTTACGTTGAAAACGACCGCAATTGTTTCCGCCGCCGCCTTGCTCGCTGG
TTTCGGCCCACCGCCTCGTGCCGCGGAACTGCCACCGGGACGTTTAGCCACCACT
GAAGACTACTTCGCTCAGCAAGCAAAACAAGCTGTTACCCCTGACGTGATGGCGC
AACTTGCGTATATGAATTATATCGATTTTATTAGCCCATTTTACTCACGTGGGTGT
TCATTTGAAGCATGGGAGTTAAAGCACACGCCGCAGCGTGTCATCAAATATAGCA
TAGCCTTTTATGCCTATGGGTTAGCTTCCGTTGCTCTCATCGATCCTAAATTGCGT
GCCTTAGCGGGTCATGATCTTGATATTGCGGTGTCTAAAATGAAATGCAAACGGG
TGTGGGGGGATTGGGAGGAGGACGGCTTCGGTACAGATCCCATAGAGAAGGAGA
ATATTATGTATAAGGGTCATCTCAATTTAATGTATGGTCTTTACCAGCTCGTGACG
GGTTCGCGCCGTTATGAAGCTGAGCATGCGCACCTGACCCGGATTATACACGACG
AAATTGCTGCAAACCCCTTCGCTGGTATCGTATGTGAACCGGATAACTACTTTGTT
CAGTGTAATAGCGTGGCGTATCTGTCACTGTGGGTTTATGACCGTTTACATGGCA
CAGATTACCGCGCCGCGACGCGTGCCTGGCTGGATTTCATACAGAAGGACCTGAT
CGACCCAGAACGGGGGGCTTTTTATCTTTCCTACCACCCGGAAAGTGGCGCGGTC
AAACCGTGGATAAGCGCATACACAACCGCATGGACTTTGGCTATGGTCCACGGTA
TGGATCCGGCGTTTAGTGAGCGTTATTATCCGCGTTTCAAGCAAACGTTCGTTGA
AGTATACGACGAAGGTAGAAAAGCCCGCGTGCGTGAAACCGCCGGCACTGATGA
TGCGGACGGCGGTGTAGGATTGGCCAGCGCTTTTACGCTGCTGCTGGCAAGAGAG
ATGGGTGATCAGCAGCTTTTTGATCAGCTGTTGAATCACTTAGAACCACCAGCGA
AACCGTCAATTGTTTCGGCGAGTCTTCGATATGAACATCCGGGCAGTCTTCTCTTT
GACGAGCTGCTCTTCTTAGCAAAAGTCCATGCAGGCTTTGGCGCATTGCTTCGTAT
GCCTCCGCCGGCCGCTAAACTGGCAGGGAAACTCGAGCACCACCACCACCACCA
CTGAGATCCGGCTGCTAACAAAGCCCGAAAGGAAGCTGAGTTGGCTGCTGCCAC
CGCTGAGCAATAACTAGCATAACCCCTTGGGGCCTCTAAACGGGTCTTGAGGGGT
TTTTTGCTGAAAGGAGGAACTATATCCGGATTGGCGAATGGGACGCGCCCTGTAG
CGGCGCATTAAGCGCGGCGGGTGTGGTGGTTACGCGCAGCGTGACCGCTACACTT
GCCAGCGCCCTAGCGCCCGCTCCTTTCGCTTTCTTCCCTTCCTTTCTCGCCACGTTC

GCCGGCTTTCCCCGTCAAGCTCTAAATCGGGGGCTCCCTTTAGGGTTCCGATTTAG
TGCTTTACGGCACCTCGACCCCAAAAAACTTGATTAGGGTGATGGTTCACGTAGT
GGGCCATCGCCCTGATAGACGGTTTTTCGCCCTTTGACGTTGGAGTCCACGTTCTT
TAATAGTGGACTCTTGTTCCAAACTGGAACAACACTCAACCCTATCTCGGTCTATT
CTTTTGATTTATAAGGGATTTTGCCGATTTCGGCCTATTGGTTAAAAAATGAGCTG
ATTTAACAAAAATTTAACGCGAATTTTAACAAAATATTAACGCTTACAATTTAGG
TGGCACTTTTCGGGGAAATGTGCGCGGAACCCCTATTTGTTTATTTTTCTAAATAC
ATTCAAATATGTATCCGCTCATGAATTAATTCTTAGAAAAACTCATCGAGCATCA
AATGAAACTGCAATTTATTCATATCAGGATTATCAATACCATATTTTTGAAAAAG
CCGTTTCTGTAATGAAGGAGAAAACTCACCGAGGCAGTTCCATAGGATGGCAAG
ATCCTGGTATCGGTCTGCGATTCCGACTCGTCCAACATCAATACAACCTATTAATT
TCCCCTCGTCAAAAATAAGGTTATCAAGTGAGAAATCACCATGAGTGACGACTGA
ATCCGGTGAGAATGGCAAAAGTTTATGCATTTCTTTCCAGACTTGTTCAACAGGC
CAGCCATTACGCTCGTCATCAAAATCACTCGCATCAACCAAACCGTTATTCATTC
GTGATTGCGCCTGAGCGAGACGAAATACGCGATCGCTGTTAAAAGGACAATTAC
AAACAGGAATCGAATGCAACCGGCGCAGGAACACTGCCAGCGCATCAACAATAT
TTTCACCTGAATCAGGATATTCTTCTAATACCTGGAATGCTGTTTTCCCGGGGATC
GCAGTGGTGAGTAACCATGCATCATCAGGAGTACGGATAAAATGCTTGATGGTCG
GAAGAGGCATAAATTCCGTCAGCCAGTTTAGTCTGACCATCTCATCTGTAACATC
ATTGGCAACGCTACCTTTGCCATGTTTCAGAAACAACTCTGGCGCATCGGGCTTC
CCATACAATCGATAGATTGTCGCACCTGATTGCCCGACATTATCGCGAGCCCATT
TATACCCATATAAATCAGCATCCATGTTGGAATTTAATCGCGGCCTAGAGCAAGA
CGTTTCCCGTTGAATATGGCTCATAACACCCCTTGTATTACTGTTTATGTAAGCAG
ACAGTTTTATTGTTCATGACCAAAATCCCTTAACGTGAGTTTTCGTTCCACTGAGC
GTCAGACCCCGTAGAAAAGATCAAAGGATCTTCTTGAGATCCTTTTTTTCTGCGC
GTAATCTGCTGCTTGCAAACAAAAAAACCACCGCTACCAGCGGTGGTTTGTTTGC
CGGATCAAGAGCTACCAACTCTTTTTCCGAAGGTAACTGGCTTCAGCAGAGCGCA
GATACCAAATACTGTCCTTCTAGTGTAGCCGTAGTTAGGCCACCACTTCAAGAAC
TCTGTAGCACCGCCTACATACCTCGCTCTGCTAATCCTGTTACCAGTGGCTGCTGC
CAGTGGCGATAAGTCGTGTCTTACCGGGTTGGACTCAAGACGATAGTTACCGGAT
AAGGCGCAGCGGTCGGGCTGAACGGGGGGTTCGTGCACACAGCCCAGCTTGGAG
CGAACGACCTACACCGAACTGAGATACCTACAGCGTGAGCTATGAGAAAGCGCC
ACGCTTCCCGAAGGGAGAAAGGCGGACAGGTATCCGGTAAGCGGCAGGGTCGGA
ACAGGAGAGCGCACGAGGGAGCTTCCAGGGGGAAACGCCTGGTATCTTTATAGT

CCTGTCGGGTTTCGCCACCTCTGACTTGAGCGTCGATTTTTGTGATGCTCGTCAGG
GGGGCGGAGCCTATGGAAAAACGCCAGCAACGCGGCCTTTTTACGGTTCCTGGCC
TTTTGCTGGCCTTTTGCTCACATGTTCTTTCCTGCGTTATCCCCTGATTCTGTGGAT
AACCGTATTACCGCCTTTGAGTGAGCTGATACCGCTCGCCGCAGCCGAACGACCG
AGCGCAGCGAGTCAGTGAGCGAGGAAGCGGAAGAGCGCCTGATGCGGTATTTTC
TCCTTACGCATCTGTGCGGTATTTCACACCGCAATGGTGCACTCTCAGTACAATCT
GCTCTGATGCCGCATAGTTAAGCCAGTATACACTCCGCTATCGCTACGTGACTGG
GTCATGGCTGCGCCCCGACACCCGCCAACACCCGCTGACGCGCCCTGACGGGCTT
GTCTGCTCCCGGCATCCGCTTACAGACAAGCTGTGACCGTCTCCGGGAGCTGCAT
GTGTCAGAGGTTTTCACCGTCATCACCGAAACGCGCGAGGCAGCTGCGGTAAAGC
TCATCAGCGTGGTCGTGAAGCGATTCACAGATGTCTGCCTGTTCATCCGCGTCCA
GCTCGTTGAGTTTCTCCAGAAGCGTTAATGTCTGGCTTCTGATAAAGCGGGCCAT
GTTAAGGGCGGTTTTTTCCTGTTTGGTCACTGATGCCTCCGTGTAAGGGGGATTTC
TGTTCATGGGGGTAATGATACCGATGAAACGAGAGAGGATGCTCACGATACGGG
TTACTGATGATGAACATGCCCGGTTACTGGAACGTTGTGAGGGTAAACAACTGGC
GGTATGGATGCGGCGGGACCAGAGAAAAATCACTCAGGGTCAATGCCAGCGCTT
CGTTAATACAGATGTAGGTGTTCCACAGGGTAGCCAGCAGCATCCTGCGATGCAG
ATCCGGAACATAATGGTGCAGGGCGCTGACTTCCGCGTTTCCAGACTTTACGAAA
CACGGAAACCGAAGACCATTCATGTTGTTGCTCAGGTCGCAGACGTTTTGCAGCA
GCAGTCGCTTCACGTTCGCTCGCGTATCGGTGATTCATTCTGCTAACCAGTAAGGC
AACCCCGCCAGCCTAGCCGGGTCCTCAACGACAGGAGCACGATCATGCGCACCC
GTGGGGCCGCCATGCCGGCGATAATGGCCTGCTTCTCGCCGAAACGTTTGGTGGC
GGGACCAGTGACGAAGGCTTGAGCGAGGGCGTGCAAGATTCCGAATACCGCAAG
CGACAGGCCGATCATCGTCGCGCTCCAGCGAAAGCGGTCCTCGCCGAAAATGAC
CCAGAGCGCTGCCGGCACCTGTCCTACGAGTTGCATGATAAAGAAGACAGTCATA
AGTGCGGCGACGATAGTCATGCCCCGCGCCCACCGGAAGGAGCTGACTGGGTTG
AAGGCTCTCAAGGGCATCGGTCGAGATCCCGGTGCCTAATGAGTGAGCTAACTTA
CATTAATTGCGTTGCGCTCACTGCCCGCTTTCCAGTCGGGAAACCTGTCGTGCCAG
CTGCATTAATGAATCGGCCAACGCGCGGGGAGAGGCGGTTTGCGTATTGGGCGCC
AGGGTGGTTTTTCTTTTCACCAGTGAGACGGGCAACAGCTGATTGCCCTTCACCG
CCTGGCCCTGAGAGAGTTGCAGCAAGCGGTCCACGCTGGTTTGCCCCAGCAGGCG
AAAATCCTGTTTGATGGTGGTTAACGGCGGGATATAACATGAGCTGTCTTCGGTA
TCGTCGTATCCCACTACCGAGATATCCGCACCAACGCGCAGCCCGGACTCGGTAA
TGGCGCGCATTGCGCCCAGCGCCATCTGATCGTTGGCAACCAGCATCGCAGTGGG

AACGATGCCCTCATTCAGCATTTGCATGGTTTGTTGAAAACCGGACATGGCACTC
CAGTCGCCTTCCCGTTCCGCTATCGGCTGAATTTGATTGCGAGTGAGATATTTATG
CCAGCCAGCCAGACGCAGACGCGCCGAGACAGAACTTAATGGGCCCGCTAACAG
CGCGATTTGCTGGTGACCCAATGCGACCAGATGCTCCACGCCCAGTCGCGTACCG
TCTTCATGGGAGAAAATAATACTGTTGATGGGTGTCTGGTCAGAGACATCAAGAA
ATAACGCCGGAACATTAGTGCAGGCAGCTTCCACAGCAATGGCATCCTGGTCATC
CAGCGGATAGTTAATGATCAGCCCACTGACGCGTTGCGCGAGAAGATTGTGCACC
GCCGCTTTACAGGCTTCGACGCCGCTTCGTTCTACCATCGACACCACCACGCTGG
CACCCAGTTGATCGGCGCGAGATTTAATCGCCGCGACAATTTGCGACGGCGCGTG
CAGGGCCAGACTGGAGGTGGCAACGCCAATCAGCAACGACTGTTTGCCCGCCAG
TTGTTGTGCCACGCGGTTGGGAATGTAATTCAGCTCCGCCATCGCCGCTTCCACTT
TTTCCCGCGTTTTCGCAGAAACGTGGCTGGCCTGGTTCACCACGCGGGAAACGGT
CTGATAAGAGACACCGGCATACTCTGCGACATCGTATAACGTTACTGGTTTCACA
TTCACCACCCTGAATTGACTCTCTTCCGGGCGCTATCATGCCATACCGCGAAAGG
TTTTGCGCCATTCGATGGTGTCCGGGATCTCGACGCTCTCCCTTATGCGACTCCTG
CATTAGGAAGCAGCCCAGTAGTAGGTTGAGGCCGTTGAGCACCGCCGCCGCAAG
GAATGGTGCATGCAAGGAGATGGCGCCCAACAGTCCCCCGGCCACGGGGCCTGC
CACCATACCCACGCCGAAACAAGCGCTCATGAGCCCGAAGTGGCGAGCCCGATC
TTCCCCATCGGTGATGTCGGCGATATAGGCGCCAGCAACCGCACCTGTGGCGCCG
GTGATGCCGGCCACGATGCGTCCGGCGTAGAGGATCGAGATCTCGATCCCGCGA
AATTAATACGACTCACTATAGGGGAATTGTGAGCGGATAACAATTCCCCTCTAGA
AATAATTTTGTTTAACTTTAAGAAGGAGATATACCATGG

397 Amino acid sequence (44,454 Da)

MRFTLKTTAIVSAAALLAGFGPPPRAAELPPGRLATTEDYFAQQAKQAVTPDVMAQL
AYMNYIDFISPFYSRGCSFEAWELKHTPQRVIKYSIAFYAYGLASVALIDPKLRALAG
HDLDIAVSKMKCKRVWGDWEEDGFGTDPIEKENIMYKGHLNLMYGLYQLVTGSRR
YEAEHAHLTRIIHDEIAANPFAGIVCEPDNYFVQCNSVAYLSLWVYDRLHGTDYRAA
TRAWLDFIQKDLIDPERGAFYLSYHPESGAVKPWISAYTTAWTLAMVHGMDPAFSER
YYPRFKQTFVEVYDEGRKARVRETAGTDDADGGVGLASAFTLLLAREMGDQQLFD
QLLNHLEPPAKPSIVSASLRYEHPGSLLFDELLFLAKVHAGFGALLRMPPPAAKLAGK

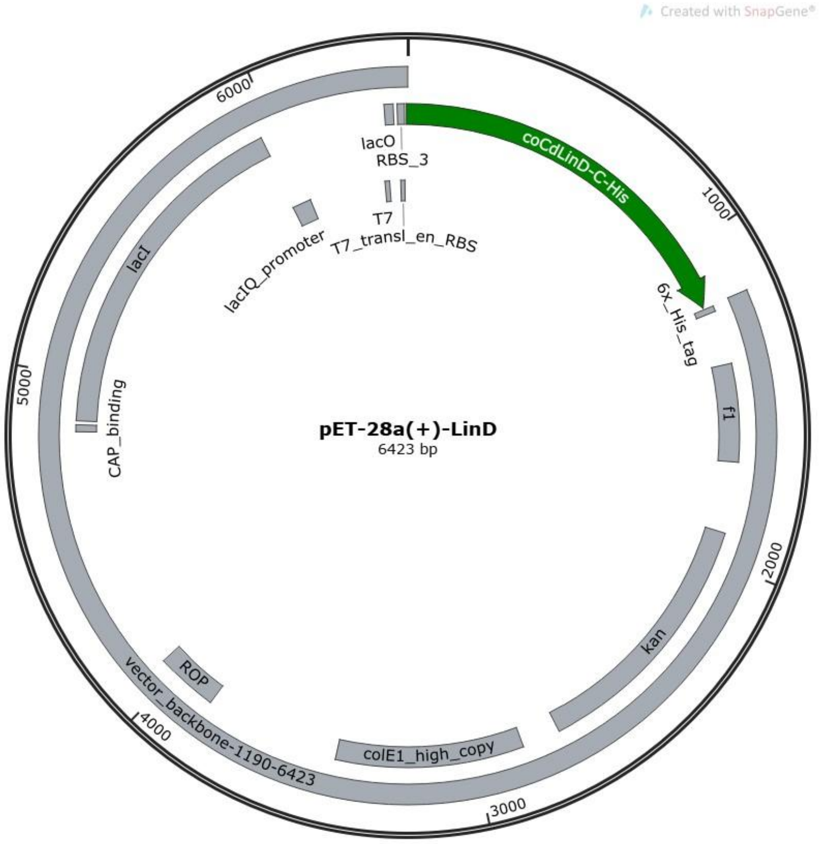

8. Computations

Geometry optimizations of **(*R*)-1a** and **(*S*)-1a** were performed through DFT calculations with ORCA 5.0.3[5–7] software. All minima were characterized at the PBE0 D3BJ def2-TZVP def2/J level of theory[8,9] in the gas phase. Visualization was performed using Avogadro[10] and Open-Source PyMOL[11].

Docking experiments with substrate **(*R*)-1a** and **(*S*)-1a** in the active site of crystal structure of LinD (PDB ID: 5HLR) as dimer were conducted using AutoDock Vina 1.2.0[12,13] software. Molecular dynamics (MD) simulations and analyses were performed using the GROMACS 2023.3-foss-2022a-CUDA-11.7.0 simulation package[14]. The Amber99sb[15] force field and SPC water model[16] were employed for the simulations. Each structure was solvated in a cubic box, and the simulation systems contained approximately 5,700 water molecules. Electrostatic interactions were calculated by using the particle mesh Ewald (PME)[17] method. Sodium (Na^+) and chloride (Cl^-) ions were added to neutralize the total net charge, ensuring all systems had a net charge of zero.

Before running the MD simulations, energy minimization was conducted using the steepest descent method to eliminate unfavorable interactions. A 100 ps NVT ensemble was carried out at a temperature close to 308 K, followed by 100 ps equilibration in the NPT ensemble. The production simulation was carried out for 100 ns at 308 K and 1 bar with a time step of 2 fs. Restrained MD simulations for each isomer were performed in triplicate. The resulting MD trajectories were analyzed using GROMACS[14] analysis tools. Free energy landscape (FEL) analysis was performed using the gmx-sham utilities of GROMACS[14].

8.1 Optimization of geometry using DFT-calculation

Optimized Cartesian coordinates for (*R*)-1a

29

Coordinates from ORCA-job NEWgeometrie

O	-0.47738785501110	0.35687693913463	3.29193483759989
C	0.09367750885470	0.87131565715961	2.08564865089934
C	-0.43142286923342	-0.07157107757362	1.00300439085115
C	-0.00998529220839	0.29113880356644	-0.40724502713753
C	-0.37899584852314	2.29923349816152	1.87272922390499
C	-0.36173274464445	-0.71815978313406	-1.46813320256196
C	1.58890709270773	0.74125949210665	2.19537424282082
C	-1.22735451455431	-1.78231834703331	-1.24116166987747
C	0.18871353557664	-0.58059962531178	-2.74192988476510
C	-1.53468578814327	-2.68086583038253	-2.25488697440176
C	-0.11516442756028	-1.47238115380261	-3.75562776308819
C	2.48903667789212	1.70453341811014	2.06128668042271
C	-0.98113298385443	-2.53085235210248	-3.51510937764951
H	-0.08423345940622	-1.07887204549731	1.25276254691913
H	-1.52188183347113	-0.08477852823292	1.09321699829480

H	-0.45925336563695	1.25010452185439	-0.68893925571937
H	1.07286006773374	0.45517991991775	-0.43046458541674
H	0.02191705035586	2.73437365385951	0.95602592532315
H	-0.07366515993095	2.93471070616249	2.70777229894536
H	-1.46861053553290	2.31208096219611	1.81508235294755
H	1.91630883995998	-0.26999357360255	2.43042731002514
H	-0.10268906345910	0.85264795155813	4.02603617839204
H	-1.67031283018172	-1.92049385422937	-0.26232816283297
H	0.87104475349138	0.24134737727605	-2.93639357669739
H	0.32791149121879	-1.34462382375847	-4.73682220319849
H	3.54710788740646	1.50127268957611	2.17851824665577
H	2.21172911973311	2.72638597773922	1.82769395446253
H	-1.21935794090808	-3.23338168381833	-4.30513843591084

Optimized Cartesian coordinates for (*S*)-1a

29

Coordinates from ORCA-job NEWgeometrie

C	-0.50074128581712	0.48726555877309	3.42576153349274
C	0.10596890714810	0.84558329918525	2.07713717760882
C	-0.43955484227456	-0.10191357610472	0.99820670397771
C	0.01070334400686	0.24826486493542	-0.40741374557925
O	-0.29434452509422	2.18107496596405	1.82477601673512
C	-0.35841713430770	-0.73848704474670	-1.48413166183576
C	1.60475551836114	0.71605747488462	2.15587850334142
C	-1.21255021545293	-1.81311792249691	-1.26482134294586
C	0.17029401912057	-0.57104754351939	-2.76377818646501
C	-1.52845484353567	-2.69471320024350	-2.29099370017367

C	-0.14246984701887	-1.44540284758251	-3.78963520685855
C	2.46544824137649	1.72230458705512	2.16043442509345
C	-0.99551812480636	-2.51600742814582	-3.55616737749749
H	-0.12098132382140	-1.11996827366793	1.24622330213895
H	-1.53169254232240	-0.08291641423907	1.06161842848583
H	-0.41068452989261	1.22157977614992	-0.68799879732842
H	1.09910641517185	0.37756789930125	-0.41810823326192
H	1.97308235768381	-0.30578506964177	2.22332721025896
H	-1.64108057024495	-1.97273278164575	-0.28291213549135
H	0. 84211072894604	0.26063784841732	-2.95340695952765
H	-2.19588244881851	-3.52641058700301	-2.09534326763269
H	0.28300009942936	-1.29435025570569	-4.77526745624322
H	3.53267629990891	1.54896763811297	2.22757325250597
H	2.13024504222294	2.75297708745498	2.11914290506720
H	-1.24037696961738	-3.20493788784321	-4.35605136093085
H	0.13815116286216	2.47297618409227	1.01691706534956
H	-1.58826809011795	0.56892575874991	3.37279971345620
H	-0.13262145185706	1.17123207915474	4.19105229299567
H	-0.23824339123853	-0.53315418964493	3.71102090126409

8.2 Most relevant interaction in the active site explored during MD-trajectories

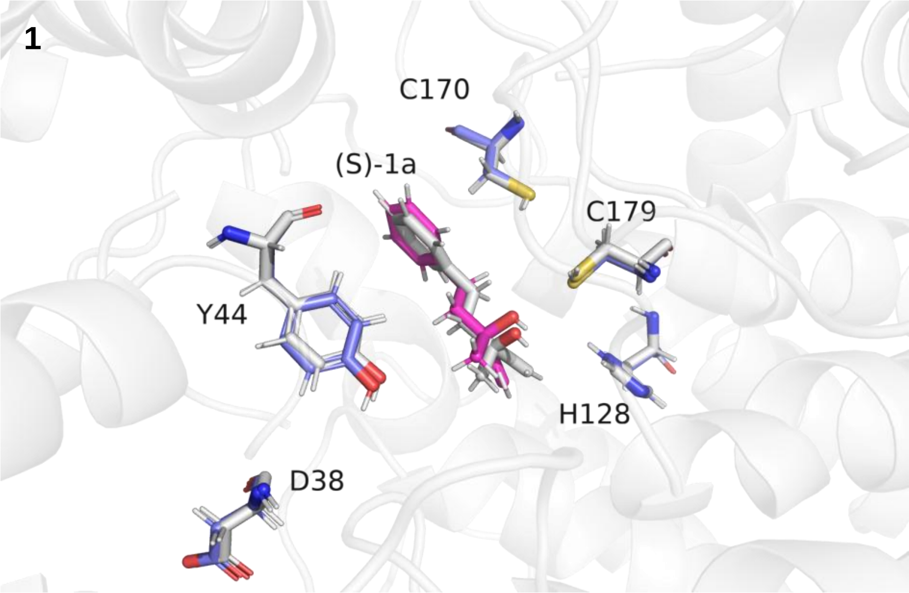

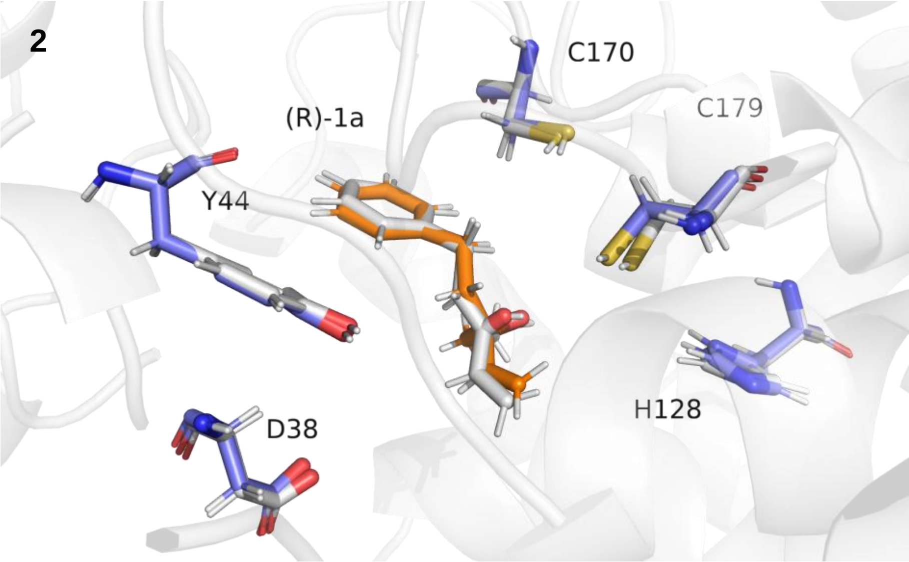

8.3 Root-mean-square deviation (RMSD) analysis of LinD with *S*- and *R*-isomers over 100 ns

Three independent MD simulations were performed to evaluate the RMSD of the LinD dimer complexed with **(*S*)-1a** and **(*R*)-1a** isomers over 100 ns. The RMSD of the protein backbone provides insight into the conformational changes and stability of the LinD enzyme-ligand complex. In the case of the **(*S*)-1a** isomer, the RMSD-value stabilized after approximately 30 ns, indicating that the LinD reaches a stable conformation relatively early in the simulation and remains stable for the duration of the run. For the **(*R*)-1a** isomer, it shows a similar average RMSD value with fluctuations around 0.1 to 0.3 nm.

1) (*S*)-1a:

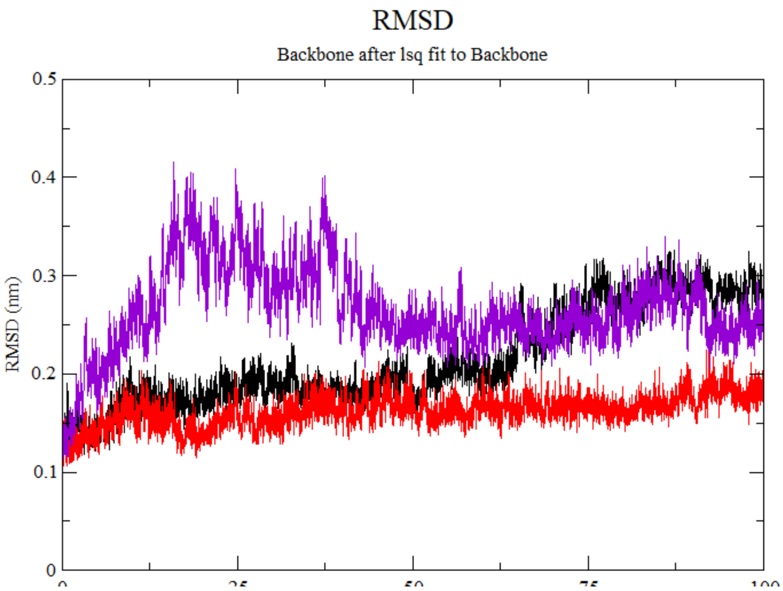

2) (*R*)-1a:

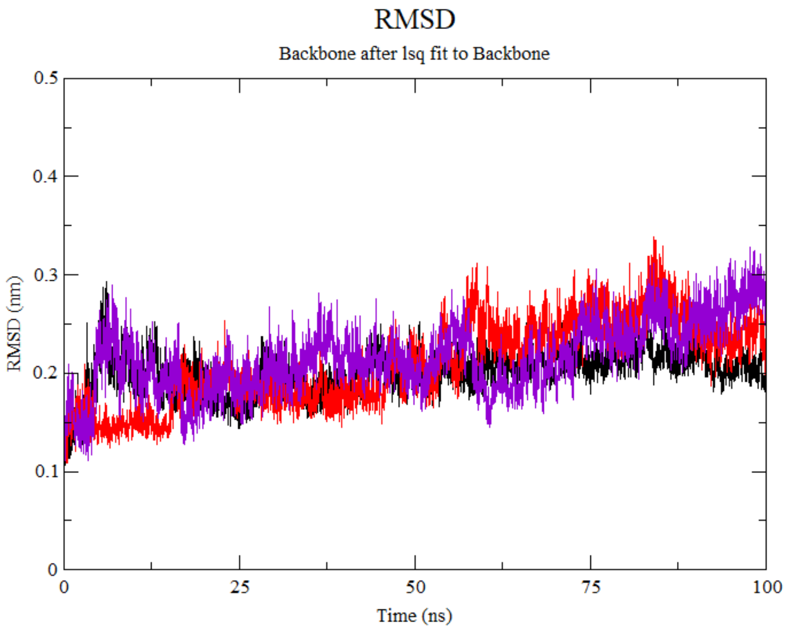

8.4 The root-mean-square fluctuation (RMSF) analysis of LinD with *S*- and *R*-isomers over 100 ns

RMSF analysis reveals similar flexibility of LinD when bound to the **(*S*)-1a** and **(*R*)-1a** isomers. The LinD dimer complexed with the *S*-isomer exhibits fluctuations with RMSF values mostly around 0.2 nm, indicating a relatively stable protein structure. Notable peaks around residues 120, 170, 250, and 300 reflect moderate flexibility in specific regions. For the **(*R*)-1a** complex, it shows comparable fluctuation patterns, with peak values likewise reaching approximately 0.2 nm.

1) (*S*)-1a:

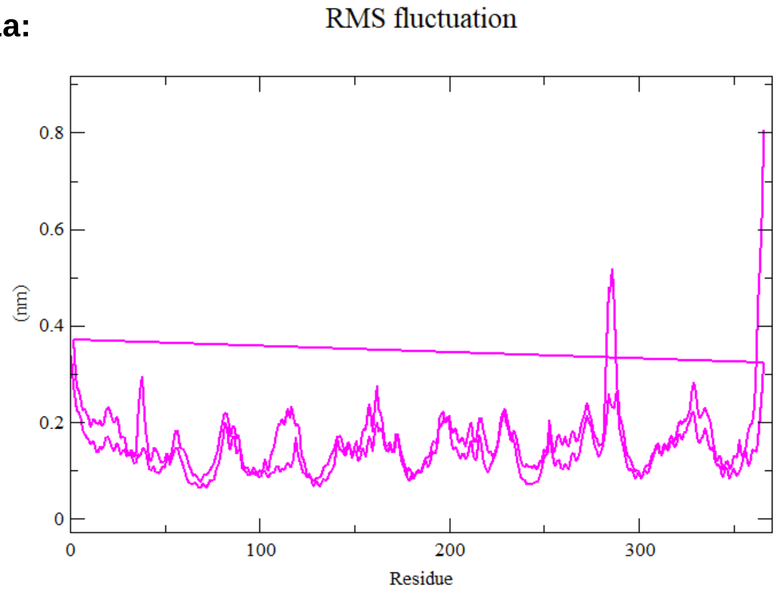

2) (*R*)-1a:

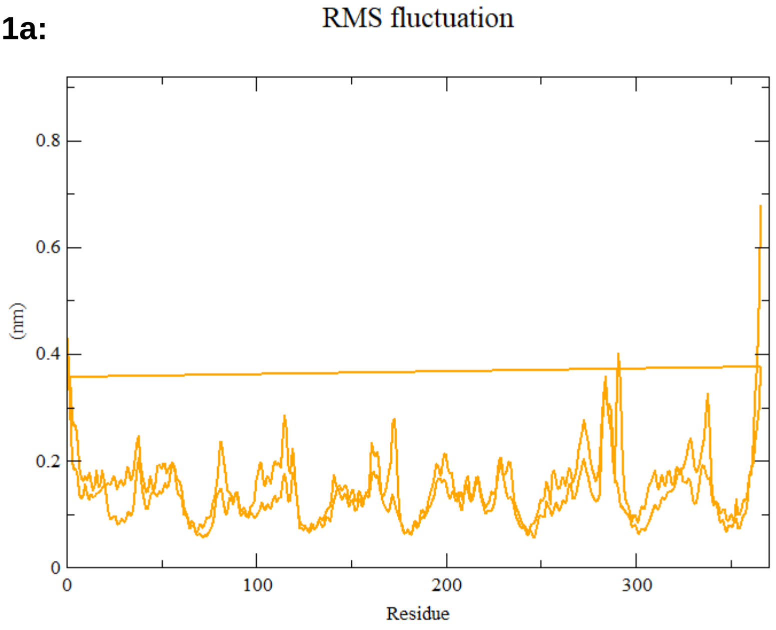

9. References

(1) Kolsi, L. E.; Yli-Kauhaluoma, J.; Moreira, V. M. Catalytic, Tunable, One-Step Bismuth(III) Triflate Reaction with Alcohols: Dehydration Versus Dimerization. *ACS Omega* **2018**, *3* (8), 8836–8842. DOI: 10.1021/acsomega.8b01401.

(2) Gajdoš, M.; Wagner, J.; Ospina, F.; Köhler, A.; Engqvist, M. K. M.; Hammer, S. C. Chiral Alcohols from Alkenes and Water: Directed Evolution of a Styrene Hydratase. *Angew. Chem. Int. Ed. Engl.* **2023**, *62* (7), e202215093. DOI: 10.1002/anie.202215093.

(3) Demming, R. M.; Hammer, S. C.; Nestl, B. M.; Gergel, S.; Fademrecht, S.; Pleiss, J.; Hauer, B. Asymmetric Enzymatic Hydration of Unactivated, Aliphatic Alkenes. *Angew. Chem. Int. Ed. Engl.* **2019**, *58* (1), 173–177. DOI: 10.1002/anie.201810005.

(4) Hinzmann, A.; Adebar, N.; Betke, T.; Leppin, M.; Gröger, H. Biotransformations in Pure Organic Medium: Organic Solvent-Labile Enzymes in the Batch and Flow Synthesis of Nitriles. *Eur. J. Org. Chem.* **2019**, *2019* (41), 6911–6916. DOI: 10.1002/ejoc.201901168.

(5) Neese, F.; Wennmohs, F.; Becker, U.; Riplinger, C. The ORCA quantum chemistry program package. *J. Chem. Phys.* **2020**, *152* (22), 224108. DOI: 10.1063/5.0004608.

(6) Neese, F. The ORCA program system. *WIREs Comput. Mol. Sci.* **2012**, *2* (1), 73–78. DOI: 10.1002/wcms.81.

(7) Neese, F. Software update: The ORCA program system—Version 5.0. *WIREs Comput. Mol. Sci.* **2022**, *12* (5). DOI: 10.1002/wcms.1606.

(8) Perdew, J. P.; Ernzerhof, M.; Burke, K. Rationale for mixing exact exchange with density functional approximations. *J. Chem. Phys.* **1996**, *105* (22), 9982–9985. DOI: 10.1063/1.472933.

(9) Adamo, C.; Barone, V. Toward reliable density functional methods without adjustable parameters: The PBE0 model. *J. Chem. Phys.* **1999**, *110* (13), 6158–6170. DOI: 10.1063/1.478522.

(10) M. D. Hanwell, D. E. Curtis, D. C. Lonie, T. Vandermeersch, E. Zurek and G. R. Hutchison. Avogadro: an advanced semantic chemical editor, visualization, and analysis platform. *J. Cheminform.*, **2012**, *4,*17.

(11) Schrodinger, LLC. 2010. The PyMOL Molecular Graphics System, Version 4.1.

(12) Eberhardt, J.; Santos-Martins, D.; Tillack, A. F.; Forli, S. AutoDock Vina 1.2.0: New Docking Methods, Expanded Force Field, and Python Bindings. *J. Chem. Inf. Model.* **2021**, *61* (8), 3891–3898. DOI: 10.1021/acs.jcim.1c00203.

(13) Trott, O.; Olson, A. J. AutoDock Vina: improving the speed and accuracy of docking with a new scoring function, efficient optimization, and multithreading. *J. Comput. Chem.* **2010**, *31* (2), 455–461. DOI: 10.1002/jcc.21334.
(14) Abraham, M. J.; Murtola, T.; Schulz, R.; Páll, S.; Smith, J. C.; Hess, B.; Lindahl, E. GROMACS: High performance molecular simulations through multi-level parallelism from laptops to supercomputers. *SoftwareX* **2015**, *1-2*, 19–25. DOI: 10.1016/j.softx.2015.06.001.
(15) Showalter, S. A.; Brüschweiler, R. Validation of Molecular Dynamics Simulations of Biomolecules Using NMR Spin Relaxation as Benchmarks: Application to the AMBER99SB Force Field. *J. Chem. Theory Comput.* **2007**, *3* (3), 961–975. DOI: 10.1021/ct7000045.
(16) Mark, P.; Nilsson, L. Structure and Dynamics of the TIP3P, SPC, and SPC/E Water Models at 298 K. *J. Phys. Chem. A* **2001**, *105* (43), 9954–9960. DOI: 10.1021/jp003020w.
(17) Shamshirgar, D. S.; Hess, B.; Tornberg, A.-K. A comparison of the Spectral Ewald and Smooth Particle Mesh Ewald methods in GROMACS, *arXiv.* **2017**, p.arXiv:1712.04718. https://ui.adsabs.harvard.edu/abs/2017arXiv171204718S (accessed January 10, 2025).
(18) Narasaka, K.; Kusama, H.; Hayashi, Y. Rearrangement of Allylic and Propargylic Alcohols Catalyzed by the Combined Use of Tetrabutylammonium Perrhenate(VII) and *p*-Toluenesulfonic Acid. *Tetrahedron* **1992**, *48* (11), 2059–2068. DOI: 10.1016/S0040-4020(01)88874-8.
(19) Pasfield, L. A.; de la Cruz, L.; Ho, J.; Coote, M. L.; Otting, G.; McLeod, M. D. Synthesis of (±)-Panduratin A and Related Natural Products Using the High-Pressure Diels–Alder Reaction. *Asian J. Org. Chem.* **2013**, *2*, 60–63. DOI: 10.1002/ajoc.201200171.
(20) Chukicheva, I. Yu.; Fedorova, I. V.; Koroleva, A. A.; Kuchin, A. V. Alkylation of Phenol by Nerol in the Presence of Organoaluminum Compounds. *Chem. Nat. Compd.* **2012**, *48* (4), 535–538. DOI:10.1007/s10600-012-0303-4.
